# Nitration of 5,11-dihydroindolo[3,2-*b*]carbazoles and synthetic applications of their nitro-substituted derivatives

**DOI:** 10.3762/bjoc.13.136

**Published:** 2017-07-14

**Authors:** Roman A Irgashev, Nikita A Kazin, Gennady L Rusinov, Valery N Charushin

**Affiliations:** 1 Postovsky Institute of Organic Synthesis, Ural Division of the Russian Academy of Sciences, Ekaterinburg, 620990, Russia; 2 Ural Federal University named after the First President of Russia, B. N. Yeltsin, Ekaterinburg, 620002, Russia

**Keywords:** electrophilic aromatic substitution, indolo[3,2-*b*]carbazole, *N*-heteroacenes, nitration, nucleophilic aromatic substitution

## Abstract

A new general approach to double nitration of 6,12-di(hetero)aryl-substituted and 6,12-unsubstituted 5,11-dialkyl-5,11-dihydroindolo[3,2-*b*]carbazoles by acetyl nitrate has been developed to obtain their 2,8-dinitro and 6,12-dinitro derivatives, respectively. A formation of mono-nitro derivatives (at C-2 or C-6) from the same indolo[3,2-*b*]carbazoles has also been observed in several cases. Reduction of 2-nitro and 2,8-dinitro derivatives with zinc powder and hydrochloric acid has afforded 2-amino- and 2,8-diamino-substituted indolo[3,2-*b*]carbazoles, while reduction of 6,12-dinitro derivatives under similar reaction conditions has been accompanied by denitrohydrogenation of the latter compounds into 6,12-unsubstituted indolo[3,2-*b*]carbazoles. Formylation of 6,12-dinitro derivatives has proved to occur only at C-2, while bromination of these compounds has taken place at both C-2 and C-8 of indolo[3,2-*b*]carbazole scaffold. Moreover, 6,12-dinitro-substituted indolo[3,2-*b*]carbazoles have been modified by the reactions with *S*- and *N*-nucleophiles. Notably, the treatment of 6,12-dinitro compounds with potassium thiolates has resulted in the displacement of both nitro groups, unlike potassium salts of indole or carbazole, which have caused substitution of only one nitro group.

## Introduction

**Figure 1:**
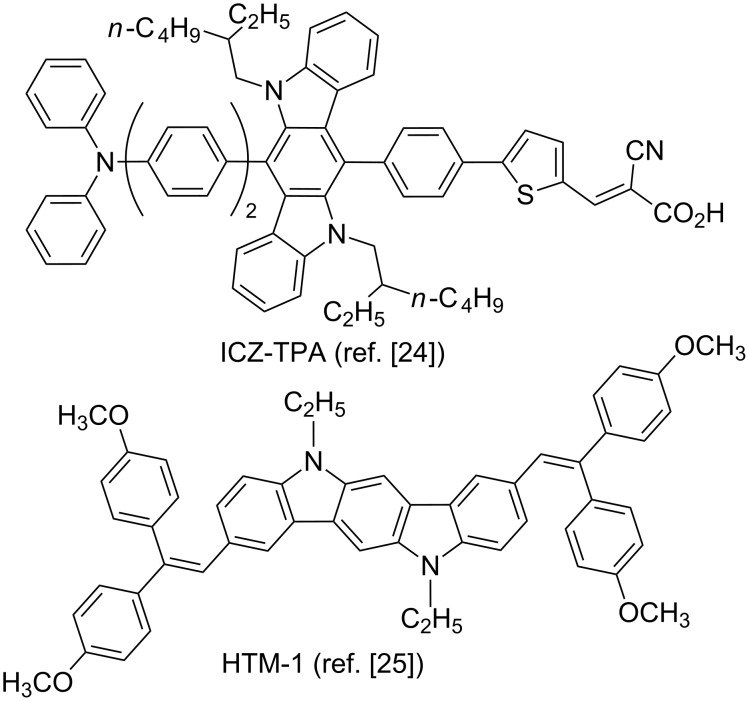
ICZ-cored materials for organic electronic devices.

Organic π-conjugated compounds, based on a ring-fused molecular architecture, have attracted great attention of researchers in the last two decades, because of their plausible use as promising materials for thin film electronic and photonic devices [1–6]. In this context, the fused π-conjugated backbone of 5,11-dihydroindolo[3,2-*b*]carbazole (indolo[3,2-*b*]carbazole, ICZ) has been successfully used as a basic structural fragment of perspective electroluminescent, hole-transporting materials and light-harvesting dyes for organic light emitting diodes (OLEDs) [7–13], organic field-effect transistors (OFETs) [14–18], and a variety of organic photovoltaics [19–25]. For instance, two functional ICZ-cored compounds, that have recently been described as effective materials for organic electronic devices, are shown in Figure 1.

It should be noted that the indolo[3,2-*b*]carbazole system can also be interesting for medicinal chemistry, because some derivatives of this family have proved to exhibit various types of biological activity [26–32].

Sustainable development of the indolo[3,2-*b*]carbazole chemistry has been observed in the last years [33], including elaboration of convenient synthetic ways to construction and modification of ICZ derivatives [34–41]. Taking into account the electron-donating character of the ICZ system, there is no doubt that electrophilic aromatic substitution (S_E_Ar) reactions are the most attractive chemical methods for modification of ICZ derivatives. We have recently reported several synthetic procedures for regioselective C2,8-formylation and acylation of indolo[3,2-*b*]carbazoles, bearing electron-rich aromatic or heteroaromatic substituents at C-6 and C-12, and also demonstrated the usage of the obtained 2,8-bis(RCO)-substituted derivatives as building blocks for the synthesis of more complicated ICZ-containing compounds [42–44]. In addition, other research groups have previously reported convenient methods for C12-formylation, azo-coupling, bromination and chlorination of 6-mono-substituted ICZs [37], as well as C2- and C2,8-bromination of 6,12-disubstituted ICZs [23,38]. Bromo-containing ICZ derivatives have also been involved in further lithiation and metal-catalyzed cross-coupling reactions.

Herein, we describe an effective protocol for nitration of 5,11-dihydroindolo[3,2-*b*]carbazoles as one more example of electrophilic substitution in this series of scaffolds. In general, nitration of aromatic compounds, one of the well-known reactions, has a great significance for various fields of industrial chemistry, as it has been exploited extensively for large-scale processes, obtaining important nitro aromatic products with a wide range of applications [45]. Moreover, incorporation of the nitro group into aromatic or electron-rich heteroaromatic systems is an efficient synthetic tool for their chemical modifications. In this context, we wish to show the usefulness of the synthesized nitro-substituted ICZ derivatives as chemical intermediates.

## Results and Discussion

### Synthesis of 2,8-dinitro- and 2-nitro-substituted indolo[3,2-*b*]carbazoles and their further reactions

**Figure 2:**
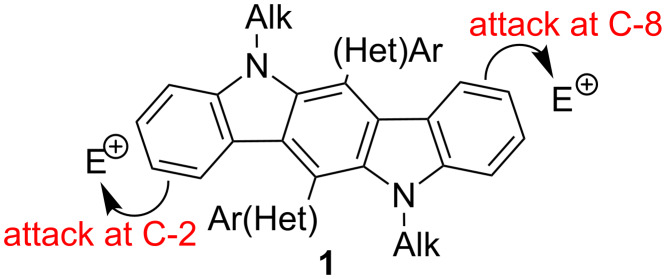
General positions for S_E_Ar in ICZs **1**.

Nitration of 6,12-di(hetero)aryl-substituted indolo[3,2-*b*]carbazoles **1** has been implemented as the first step of our research studies. It has previously been shown that π-excessive system of 6,12-disubstituted indolo[3,2-*b*]carbazoles **1** has a high affinity to electrophilic agents, which react readily with these aromatic substrates, thus affording C2,8-substitution products (Figure 2) [42–43].

**Scheme 1:**
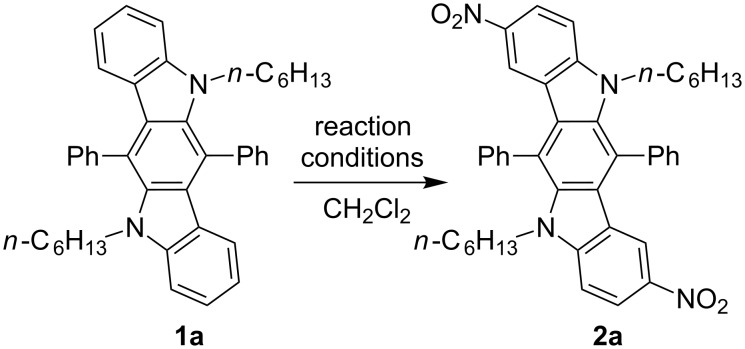
Double nitration of indolo[3,2-*b*]carbazole **1a**.

**Figure 3:**
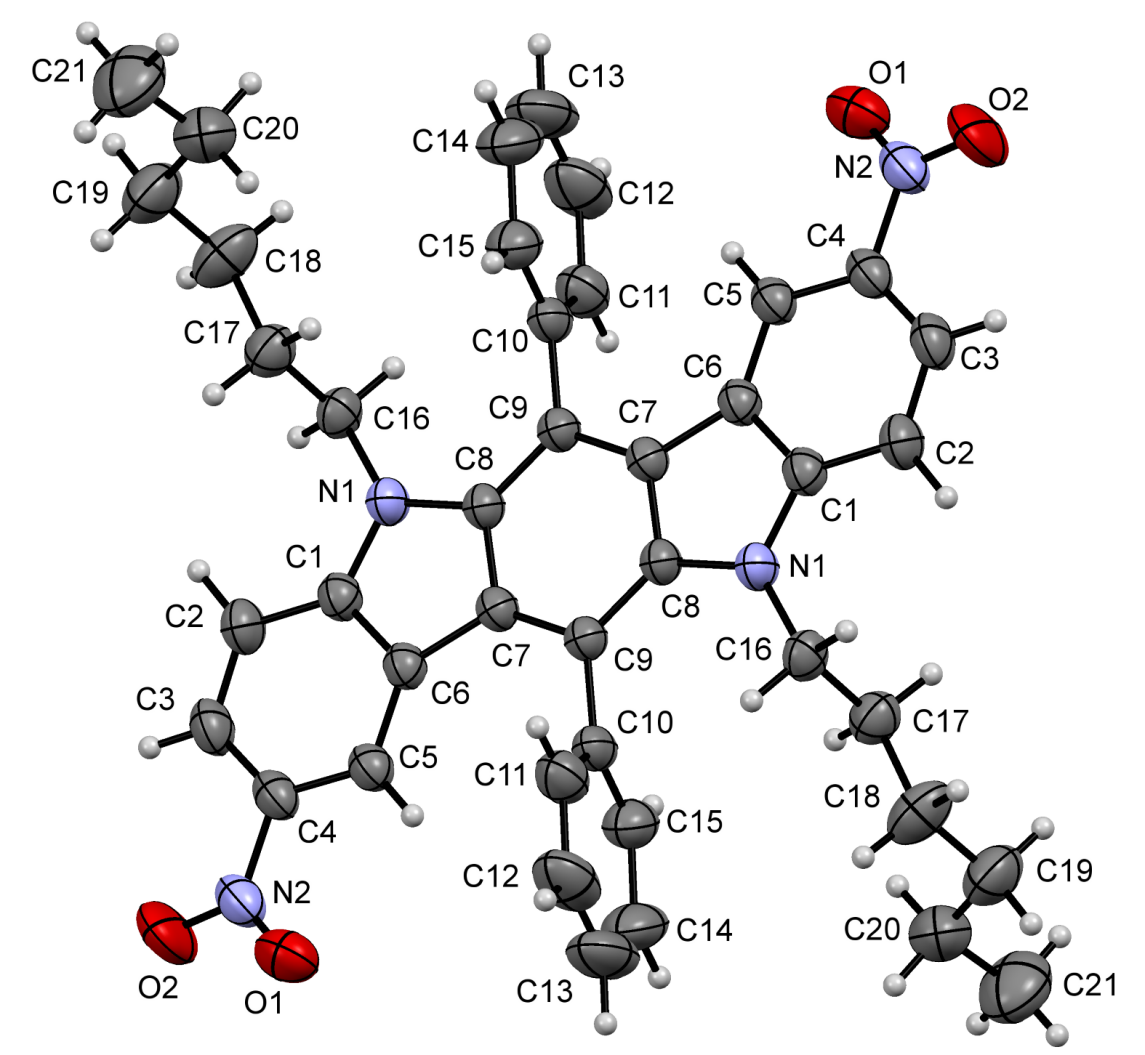
X-ray single crystal structure of compound **2a**. Thermal ellipsoids of 50% probability are presented.

On the other hand, undesired electrophilic substitution can occur in electron-rich (hetero)aromatic substituents of compounds **1** as a side reaction. In this relation, we have focused our efforts on finding suitable conditions for nitration of these compounds in order to incorporate nitro groups exclusively at C-2 and C-8, avoid side nitration of (hetero)aromatic substituents at C-6 and C-12, and exclude over nitration of ICZ scaffolds. It should be noted, that a number of procedures for nitration of carbazoles, formal progenitors of ICZ structures, have been described in the literature, and could be useful for our current research. In many cases 3,6-unsubstituted carbazoles have been nitrated by using fuming or 70% nitric acid with or without addition of acetic anhydride [46]. Two inorganic nitrates, such as copper(II) nitrate [47] or cerium(IV) ammonium nitrate (CAN) [48] have also been used to give 3-mononitro or 3,6-dinitro carbazoles as major products. Taking into account the above mentioned procedures, we have used fuming nitric acid and acetyl nitrate [49] (generated in situ from fuming nitric acid and acetic anhydride) for nitration of indolo[3,2-*b*]carbazole **1a** as a model compound (Scheme 1, Table 1). These experiments have been performed in dry CH_2_Cl_2_, which has proved to be a very effective solvent for compounds **1**, even at a low temperature. We have observed a poor conversion of compound **1a** in experiments with stoichiometric amounts (2 equiv) of nitrating agents, in which have given only trace amounts of the product **2a** (Table 1, entry 1 and 2). However, the starting aromatic substrate is exhausted after 15 min (control by TLC), if nitrating agents are used in excess amounts. Indeed, treatment of ICZ **1a** with 5 equiv of acetyl nitrate at 0 °C has resulted in the formation of compound **2a** with a small impurity of byproducts (Table 1, entry 3), however, all attempts to separate the target product from these impurities have failed. The regioselectivity of this process can be improved by decreasing the reaction temperature to –20 °C, and the use of the same amounts of acetyl nitrate (Table 1, entry 4) has provided the desired product in 88% yield. At the same time, in the experiment with fuming nitric acid (5 equiv), used instead of acetyl nitrate (Table 1, entry 5), we have obtained a mixture of compound **2a** and byproducts again. The molecular structure of compound **2a** has been proved unequivocally by X-ray crystallography analysis, thus supporting the data of ^1^H and ^13^C NMR spectroscopy (Figure 3).

**Table 1:** Nitration of ICZ **1a** under different reaction conditions.

Entry	Reaction conditions	Yield (%) **2a**

1	2 equiv AcONO_2_, 1 h at 0 °C	traces^a^
2	2 equiv HNO_3_, 1 h at 0 °C	traces^a^
3	5 equiv AcONO_2_, 15 min at 0 °C	80^b^
4	5 equiv AcONO_2_, 15 min at −20 °C	88
5	5 equiv HNO_3_, 15 min at −20 °C	78^b^

^a^Poor conversion of starting material **1a** was observed. ^b^Compound **2a** could not be separated from byproducts.

**Scheme 2:**
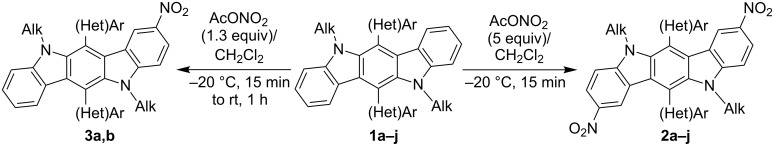
C2- and C2,8-nitration of indolo[3,2-*b*]carbazoles **1**.

We have successfully used the reaction conditions, which have proved to be the most acceptable for the nitration of compound **1a** (Table 1, entry 4), for similar transformations of others ICZ substrates **1b–j** bearing various (hetero)aromatic fragments at C-6 and C-12, including electron-rich alkoxyaryls in **1b**,**h**, thiophen-2-yl in **1f**,**i**,**j** and benzo[*b*]thiophen-2-yl in **1e**. In this manner, a series of 2,8-dinitro-substituted ICZs **2b–j** has been obtained in 64–89% yields (Scheme 2, Table 2). Taking into account a strong electron-withdrawing character of the nitro group, deactivating the ICZ system for the second nitration, we have also suggested that mono C2-nitration of ICZs **1** can be possible, if we apply nearly stoichiometric amounts of acetyl nitrate. To test this version, compounds **1a**,**b** have been treated with a slight excess of acetyl nitrate (1.3 equiv) at −20 °C for 15 min and then kept at ambient temperature for 1 h. Indeed, the expected 2-nitro-substituted ICZs **3a**,**b** have been formed exclusively under these reaction conditions, but with incomplete conversion of ICZs **1a**,**b** (Scheme 2, Table 2). Products **3a**,**b** have been isolated by column chromatography in moderate yields, while nearly one-half of the starting materials **1a**,**b** have been returned during process of separation. It has been mentioned above (see Table 1, entries 1 and 2), that double nitration of the ICZ core of compound **1a** has been observed with two equivalents of acetyl nitrate. Therefore, attempts to enhance yields of compounds **3** by using large amounts of acetyl nitrate have proved to be unsuccessful.

**Table 2:** Scope and yields of nitro-substituted ICZs **2** and **3**.

Entry	ICZ **1**	NO_2_-ICZ **2** or **3**	Het(Ar)	Alk	Yield (%)

1	**1a**	**2a**	Ph	*n*-C_6_H_13_	88
2	**1b**	**2b**	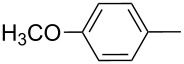	*n*-C_6_H_13_	80
3	**1c**	**2c**	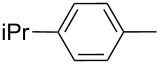	*n*-C_6_H_13_	87
4	**1d**	**2d**	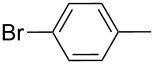	*n*-C_6_H_13_	89
5	**1e**	**2e**	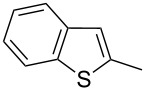	*n*-C_6_H_13_	64
6	**1f**	**2f**	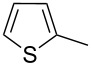	*n*-C_6_H_13_	84
7	**1g**	**2g**	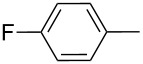	*n*-C_6_H_13_	87
8	**1h**	**2h**	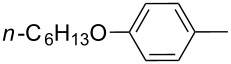	*n*-C_6_H_13_	81
9	**1i**	**2i**	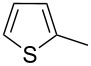	*n*-C_7_H_15_	76
10	**1j**	**2j**	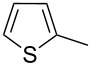	*n*-C_15_H_31_	72
11	**1a**	**3a**	Ph	*n*-C_6_H_13_	54
12	**1b**	**3b**	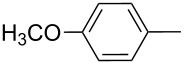	*n*-C_6_H_13_	50

**Scheme 3:**
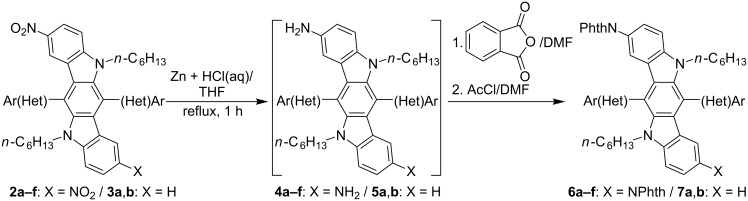
Reduction of nitro-substituted ICZs **2** and **3**.

Notably, 2,8-diamino-substituted ICZ derivatives have previously been prepared by using the Buchwald–Hartwig amination of the corresponding 2,8-dibromo-ICZs [8]. In this study, we have realized a convenient synthesis of amino-containing ICZ derivatives, starting from 2,8-dinitro-ICZs **2** and 2-nitro-ICZ compounds **3** (Scheme 3). Indeed, nitro compounds **2a–f** and **3a**,**b** can easily be reduced on treatment with an excess of zinc powder and hydrochloric acid in THF solution under reflux for 1 h, thus affording amines **4a–f** and **5a**,**b**. The latter amines have been separated in free-base form by extraction of the reaction mixtures after alkaline workup. However, further purification and characterization of compounds **4** and **5** have proved to be rather problematic, because of the poor stability of these amines towards oxidation by air oxygen. In this respect, crude samples of these compounds should be stored under inert atmosphere and better used immediately after preparation for further synthesis. Thus, we have converted crude amines **4** and **5** into the corresponding phthalimides (PhthN-ICZ) **6** and **7** (Scheme 3, Table 3), which have been isolated in 53–89% yields, based on the starting nitro compounds **2** and **3**.

**Table 3:** Scope and yields of phthalimides **6** and **7**.

Entry	NO_2_-ICZ	PhthN-ICZ	Het(Ar)	Yield (%)

1	**2a**	**6a**	Ph	62
2	**2b**	**6b**	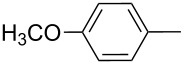	63
3	**2c**	**6c**	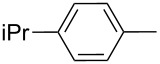	64
4	**2d**	**6d**	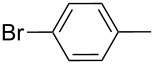	61
5	**2e**	**6e**	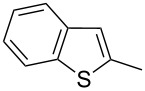	53
6	**2f**	**6f**	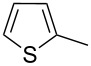	75
7	**3a**	**7a**	Ph	89
8	**3b**	**7b**	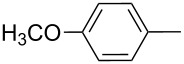	75

### Synthesis of 6,12-dinitro and 6-nitro-substituted indolo[3,2-*b*]carbazoles and their further reactions

**Scheme 4:**
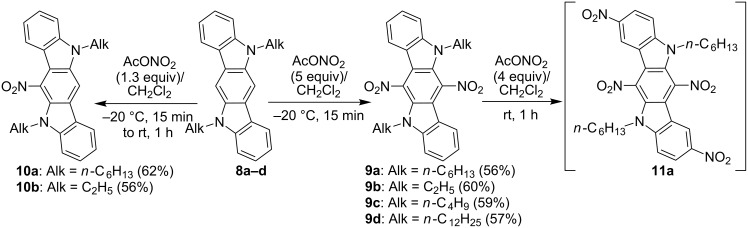
Nitration of 6,12-unsubstituted indolo[3,2-*b*]carbazoles **8**.

In the frames of this research study we have elucidated the nitration of 6,12-unsubstituted indolo[3,2-*b*]carbazoles **8a–d**, by using acetyl nitrate at low temperatures. It has been found that compounds **8** undergo the double nitration under reaction conditions mentioned above, affording products **9a–d** (Scheme 4). However, the nitration of ICZs **8** takes place at the central benzene ring of their fused scaffolds at the vacant C-6 and C-12 positions instead of C-2 and C-8, as it has been observed for ICZs **1**. The structure of **9** have been evidenced by ^1^H NMR spectroscopic data. Indeed, the resonance signal of magnetically equivalent H6 and H12 protons is absent in the ^1^H NMR spectra of nitro derivatives **9**, while this singlet is observed at 8.01–8.04 ppm in the ^1^H NMR spectra of the starting aromatic compounds **8**. We have demonstrated that mono C6-nitration of ICZ compounds **8a**,**b** has afforded 6-nitro-ICZs **10a**,**b** (Scheme 4). It has turned out that further nitration of 6,12-dinitro-ICZ **9a** with an excess of acetyl nitrate at ambient temperature has afforded 2,6,8,12-tetranitro-substituted ICZ **11a**, which has been formed together with a number of unidentified byproducts (Scheme 4). We have failed our attempts to isolate compound **11a** in analytical pure form due to a similar nature of the target product and concomitant impurities.

**Figure 4:**
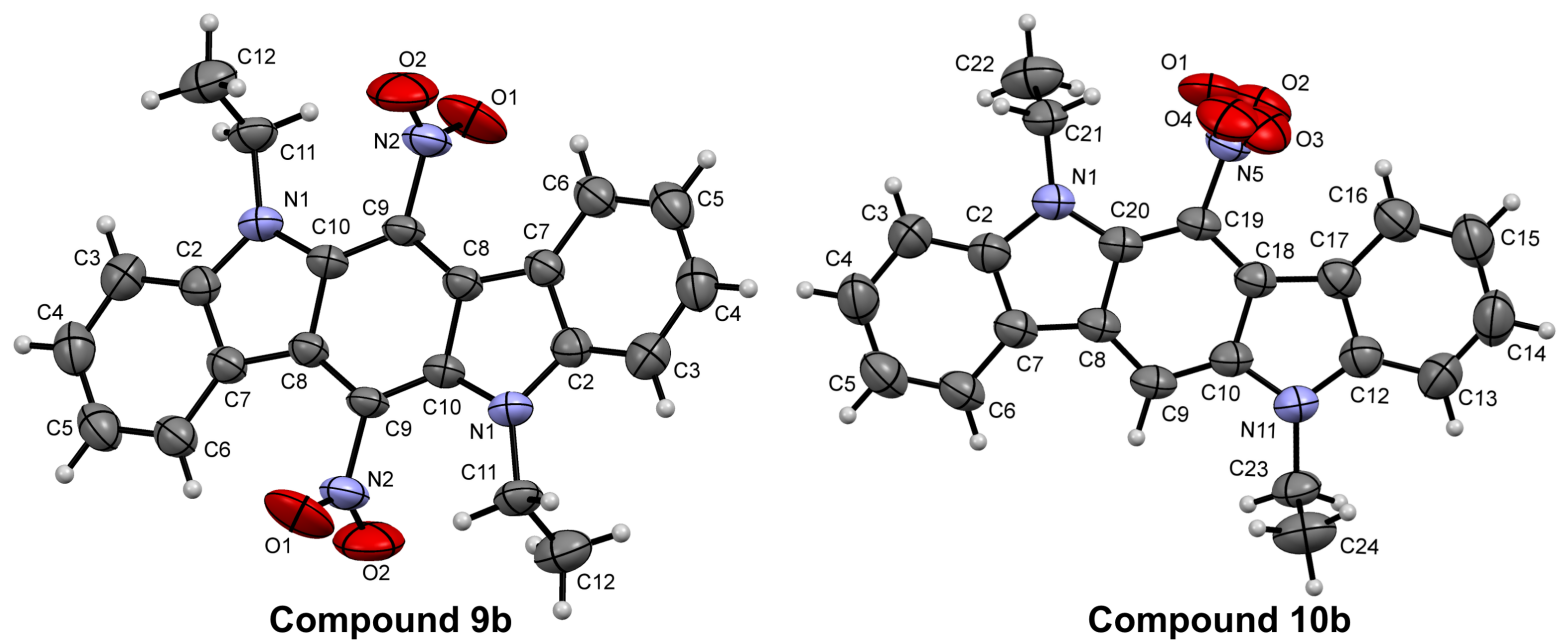
X-ray single crystal structure of compounds **9b** and **10b**. Thermal ellipsoids of 50% probability are presented.

In addition to the data of ^1^H and ^13^C NMR and elemental analysis, the structure of nitro-substituted ICZs **9** and **10** has been confirmed unequivocally by X-ray crystallography analysis of derivatives **9b** and **10b** (Figure 4).

**Scheme 5:**
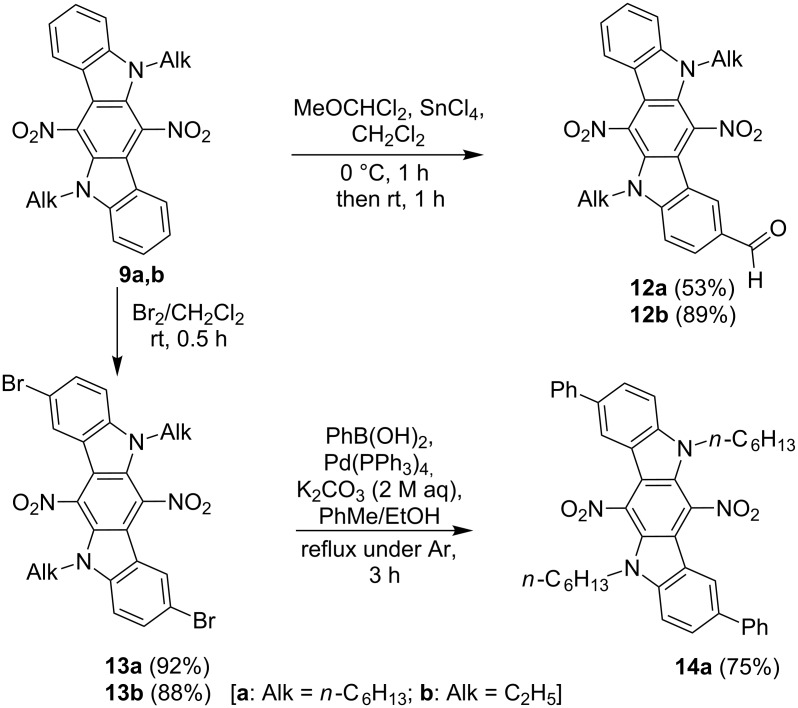
Modification of 6,12-dinitro-ICZs **9a**,**b** by electrophilic substitution.

Taking into account that 6,12-dinitro-ICZs **9** are susceptible to electrophilic substitution at their terminal benzene rings, we have investigated bromination and formylation of derivatives **9a**,**b**, and our hopes for regioselectivity of these reactions have proved to be justified. In particular, aldehydes **12a**,**b** have been obtained on treatment of compounds **9a**,**b** with an excess of dichloromethyl methyl ether in the presence of Sn(IV) chloride (Scheme 5).

**Figure 5:**
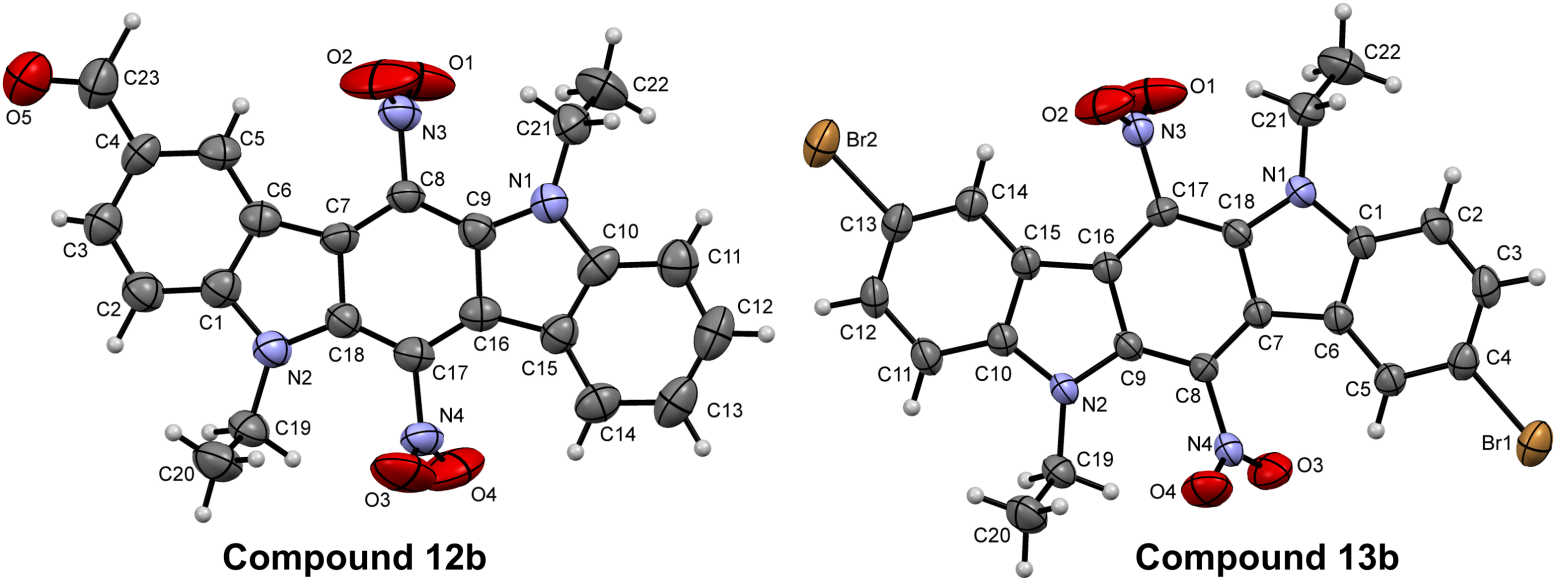
X-ray single crystal structure of compounds **12b** and **13b**. Thermal ellipsoids of 50% probability are presented.

This formylation reaction has proved to occur only at C-2 position, instead of both C-2 and C-8. This fact can be explained by strong negative M-effects of two nitro groups, decreasing the reactivity of ICZs **9** towards mild electrophiles. Unlike the formylation reaction, electrophilic bromination of compounds **9a**,**b** has easily taken place on their treatment with bromine, affording dibromo compounds **13a**,**b**. Further transformation of 2,8-dibromo-ICZ **13a** into derivative **14a** has been performed by using the Suzuki–Miyaura cross-coupling reaction with phenylboronic acid under Pd catalysis (Scheme 5). The location of the formyl group and bromine atoms in ICZ derivatives **12** and **13** has been established by X-ray crystallography analysis, performed for single crystals of **12b** and **13b** (Figure 5).

**Scheme 6:**
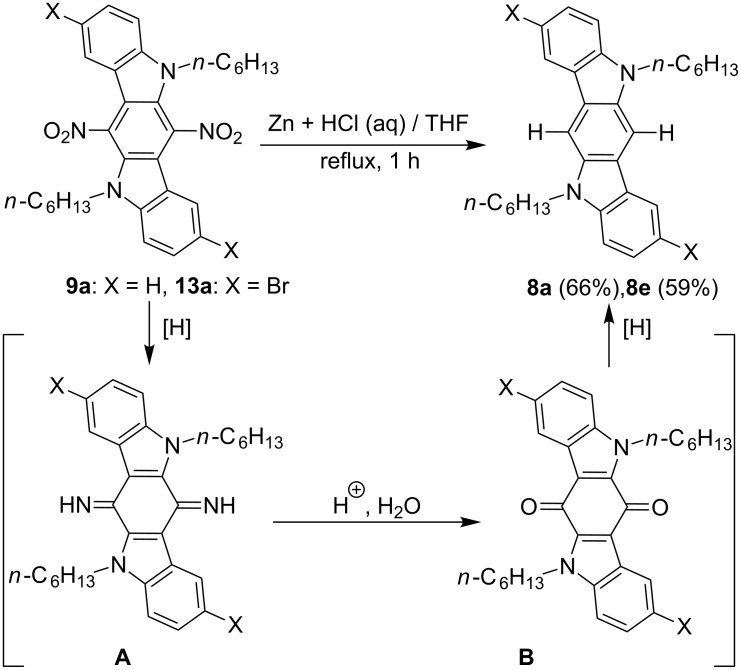
A possible mechanism for the reduction of 6,12-dinitro-ICZs **9a** and **13a**.

We have obtained unexpected results for the reduction of 6,12-dinitro-ICZs with zinc powder and hydrochloric acid in THF solution. Formal exchange of both nitro groups with hydrogen atoms has proved to occur under these reaction conditions, giving 6,12-unsubstituted ICZs **8a** and **8e** from the nitro compounds **9a** and **13a**, respectively (Scheme 6). It is known that 9,10-anthraquinones can be converted into 9,10-unsubstituted anthracenes on reduction with zinc under basic or acidic conditions. A plausible mechanism for the discovered transformation can involve simultaneous reduction of both nitro groups in 6,12-dinitro-ICZs, giving the ICZ-quinone diimine **A**. Then the intermediate **A** is hydrolyzed under acidic conditions into ICZ-quinone **B**, which is reduced into 6,12-unsubstituted ICZ derivatives (Scheme 6). The suggested mechanism is only a hypothesis, which is based on a similar reduction of anthraquinones. Anyway, the discussed transformation is the case of unusual reduction of nitroaromatic compounds. In general, the chemical reactions, leading to direct displacement of the nitro group in nitroarenes with a hydrogen atom, are rare phenomena. For instance, we can mention the reaction of nitrobenzenes with potassium cyanide as the procedure, affording the *ortho*-carboxylation of the benzene ring relative to the leaving nitro group and known as von Richter rearrangement [50]. At the same time, reduction of **9a** with Na_2_S_2_O_4_, Sn(II) chloride or H_2_ and Pd (10 wt %) on charcoal catalyst has proved to be unsuccessful, since we have obtained complicated mixtures of reaction products in those experiments.

**Scheme 7:**
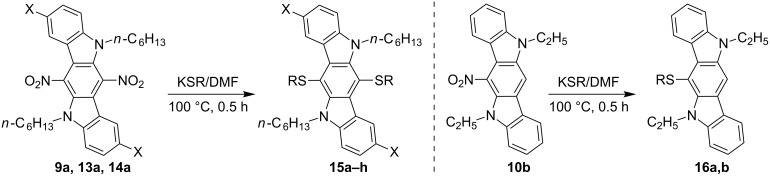
Reactions of 6-nitro- and 6,12-dinitro-ICZs with *S*-nucleophiles.

Further transformation of 6,12-dinitro-substituted ICZs can be realized through nucleophilic aromatic substitution, taking into account a mutual activation of the *para*-disposed nitro groups in these aromatics. Thus, treatment of compounds **9a**, **13a** and **14a** with potassium thiolates in DMF solution has led to substitution of both nitro groups, so that new ICZ compounds **15a–h**, bearing a RS-group at C-6 and C-12, respectively, have been obtained in 65 to 92% yields. In a similar reaction with 6-nitro-ICZ **10b** we have also observed the substitution of the single unactivated nitro group with alkylthio moiety, thus leading to the formation of products **16a**,**b** (Table 4, Scheme 7). Initial displacement of the first nitro group in 6,12-dinitro-ICZs with an alkyl(aryl)thio substituent appears to proceed through a conventional mechanism of nucleophilic aromatic substitution (S_N_Ar). On the other hand, conversion of the second nitro group, as well as a similar transformation of compound **10b**, can possibly be explained in terms of the radical-nucleophilic aromatic substitution (S_RN_1), since no other electron-withdrawing groups, facilitating the S_N_Ar reactions, are present in these aromatics [51]. Such type of nitro–thiolate substitution is a rare phenomenon; only a few reports on this topic are available in the literature [52–54].

**Table 4:** Scope and yields of alkyl(aryl)thio-substituted ICZs **15** and **16**.

Entry	NO_2_-ICZ	X	RS-ICZ	R	Yield (%)

1	**9a**	H	**15a**	CH_3_	69
2	**9a**	H	**15b**	*n*-C_3_H_7_	65
3	**9a**	H	**15c**	iPr	70
4	**9a**	H	**15d**	*n*-C_18_H_37_	72
5	**9a**	H	**15e**	Ph	92
6	**13a**	Br	**15f**	Ph	74
7	**13a**	Br	**15g**	*n*-C_3_H_7_	68
8	**14a**	Ph	**15h**	*n*-C_3_H_7_	90
9	**10b**	H	**16a**	iPr	56
10	**10b**	H	**16b**	*n*-C_3_H_7_	68

**Scheme 8:**
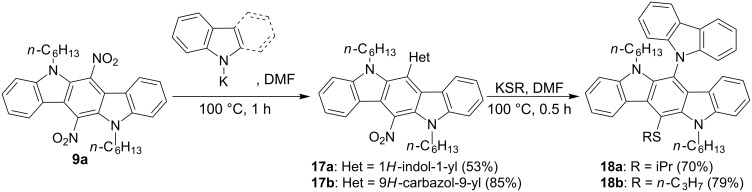
Successive substitution of nitro groups in 6,12-dinitro-ICZ **9a** with *N*- and *S*-nucleophiles.

Some *N*-nucleophiles are also able to react with 6,12-dinitro-substituted ICZs. In particular, treatment of compound **9a** with the potassium salts of indole or carbazole in DMF solution has proceeded smoothly, giving mono-substitution products **17a**,**b**. At the same time, 6,12-dinitro-ICZ **9a** has undergone degradation during the reaction with primary and secondary aliphatic amines, such as *n*-butylamine or pyrrolidine. In addition, substitution of the nitro group in **17b** with an alkylthio residue can be performed easily by the usage of potassium thiolates. It shows that consecutive displacement of both nitro groups in 6,12-dinitro-ICZs can be realized through combination of *N*- and *S*-nucleophiles (Scheme 8).

## Conclusion

We have studied the behavior of 5,11-dihydroindolo[3,2-*b*]carbazole systems, bearing (hetero)aromatic fragments at C-6 and C-12, and 6,12-unsubstituted ones towards electrophilic nitrating agents. A series of new 2,8-dinitro- and 6,12-dinitro-substituted indolo[3,2-*b*]carbazoles has been obtained by using acetyl nitrate at a low temperature. Synthetic usefulness of the obtained nitro derivatives has been demonstrated by several examples. The nitro derivatives of the indolo[3,2-*b*]carbazole family can be considered as promising intermediates for further chemical transformations. In particular, 2,8-diamino-substituted ICZs have been obtained easily from the corresponding 2,8-dinitro derivatives through their reduction with zinc powder and hydrochloric acid. In contrast to 2,8-dinitro-ICZs, 6,12-dinitro-ICZs are able to loose both nitro groups under similar reductive conditions, forming 6,12-unsubstituted derivatives as major products. At the same time, 6,12-dinitro-ICZs demonstrate a remarkable synthetic utility, since both electrophilic and nucleophilic aromatic substitution procedures can successfully be used for their transformations. For instance, we have prepared 6,12-bis(alkyl(aryl)thio)-substituted ICZs, which are difficult to obtain by other ways, by reacting 6,12-dinitro-ICZs with potassium thiolates. In summary, structural modifications of the indolo[3,2-*b*]carbazole system reported herein are of importance as useful synthetic tools and open new opportunities for design of ICZ-cored molecules.

## Supporting Information

File 1 Experimental procedures, characterization data for new compounds and copies of ^1^H and ^13^C NMR spectra.
