# Supplementary material for: Nitration of 5,11-dihydroindolo[3,2-b]carbazoles and synthetic applications of their nitro-substituted derivatives
Source: Beilstein J Org Chem. 2017 Jul 14;13:1396–406. doi: 10.3762/bjoc.13.136 (PMC5530725; doi:10.3762/bjoc.13.136)

Supporting Information

for

Nitration of 5,11-dihydroindolo[3,2-*b*]carbazoles and synthetic applications of their nitro-substituted derivatives

Roman A. Irgashev*[1,2], Nikita A. Kazin[1], Gennady L. Rusinov[1,2], Valery N. Charushin[1,2]

Address: [1]Postovsky Institute of Organic Synthesis, Ural Division of the Russian Academy of Sciences, Ekaterinburg, 620990, Russia and [2]Ural Federal University named after the First President of Russia, B. N. Yeltsin, Ekaterinburg, 620002, Russia

Email: Roman A. Irgashev - irgashev@ios.uran.ru

*Corresponding author

Experimental procedures, characterization data for new compounds and copies of ^{1}H and ^{13}C NMR spectra.

Table of content

1. General information S2
2. General procedure for synthesis of 2,8-dinitro and 6,12-dinitro ICZ derivatives 2a-j and 9a-d S2
3. General procedure for synthesis of 2-nitro and 6-nitro ICZ derivatives 3a,b and 10a,b S6
4. General procedure for reduction of 2,8-dinitro and 2-nitro ICZ derivatives 2a–f and 3a,b S8
5. General procedure for synthesis of phtalimides 6a–f and 7a,b S8
6. General procedure for formylation of 6,12-dinitro ICZ derivatives 9a,b S11
7. General procedure for bromination of 6,12-dinitro ICZ derivatives 9a,b S12
8. Procedure for synthesis of 2,8-diphenyl ICZ derivative 14a S13
9. General procedure for denitrohydrogenation of 6,12-dinitro ICZ derivatives 9a and 13a S13
10. General procedure for synthesis of alkyl(aryl)thyo ICZ derivatives 15a–h and 16a,b S14
11. General procedure for synthesis of ICZ compounds 17a,b S17
12. General procedure for synthesis of ICZ compounds 18a,b S18
13. Crystallographic data and results of refinement for the structure 2a in the XRD experiment S20
14. Crystallographic data and results of refinement for the structure 9b in the XRD experiment S21
15. Crystallographic data and results of refinement for the structure 10b in the XRD experiment S22
16. Crystallographic data and results of refinement for the structure 12b in the XRD experiment S23
17. Crystallographic data and results of refinement for the structure 13b in the XRD experiment S24
18. References S25
19. Copies of ^{1}H and ^{13}C NMR spectra of new compounds S26

1. General information.

^{1}H and ^{13}C NMR spectra were obtained on a Bruker DRX-400 and AVANCE-500 spectrometers at ambient temperature in $CDCl_3/C_6D_6$/DMSO-d_6 solution with TMS as the internal standard. The ^{13}C NMR spectra of compounds **9b**, **9c**, **12b**, **13a** and **13b** were not recorded due to poor solubility of these substances in a majority of deuterated solvents. Elemental analysis was carried on a Eurovector EA 3000 automated analyzer. Mass spectrometry was performed using a Bruker maXis Impact HD spectrometer. Melting points were determined on Boetius combined heating stages and were not corrected. X-ray diffraction analysis was performed on an automated X-ray diffractometer “Xcalibur E” on standard procedure. Column chromatography was performed on silica gel (230−450 mesh). All solvents used were dried and distilled per standard procedures. White fuming HNO_3 (97%, d = 1.50 g/ml) was used for preparation of acetyl nitrate. The indolo[3,2-*b*]carbazoles **1a–j** and **8a–d** were prepared in accordance with the previously described procedures [1-5].

2. General procedure for synthesis of 2,8-dinitro and 6,12-dinitro ICZ derivatives 2a–j and 9a–d.

A solution of acetyl nitrate, prepared in situ from white fuming HNO_3 (0.44 ml, 10 mmol) and acetic anhydride (1.15 ml, 12 mmol), in dry CH_2Cl_2 (10 ml) was added dropwise to the stirring solution of an appropriate indolo[3,2-*b*]carbazole **1** (2 mmol) in dry CH_2Cl_2 (50 ml) at −20 °C and the resulting dark-red mixture was stirred at this temperature for 15 min. The saturated solution of $NaHCO_3$ (50 ml) was added to the reaction mixture and well stirred for 0.5 h. A suspension of the organic solid was obtained after rotary evaporation of CH_2Cl_2 under vacuum from this biphasic mixture. The formed precipitate was filtered and crystallized from DMF (15 ml). The analytically pure product **2** was separated by filtration, washed with EtOH (5 × 5 ml) and dried at 120 °C.

Synthesis of 6,12-dinitro derivatives **9a–d** was carried out in accordance with the above procedure starting from indolo[3,2-*b*]carbazoles **8a–d** (2 mmol).

*2.1. 5,11-Dihexyl-2,8-dinitro-6,12-diphenyl-5,11-dihydroindolo[3,2-b]carbazole (**2a**)*

Orange crystals (1.17 g, 88% yield), mp 337–338 °C.

^{1}H NMR (500 MHz, $CDCl_3$) δ 8.27 (dd, J = 9.0, 2.3 Hz, 2H), 7.82 – 7.72 (m, 6H), 7.69 – 7.63 (m, 4H), 7.36 (d, J = 2.2 Hz, 2H), 7.27 (d, J = 9.1 Hz, 2H), 3.95 – 3.88 (m, 4H), 1.58 – 1.53 (m, 4H), 1.27 – 1.19 (m, 4H), 1.16 – 1.09 (m, 4H), 0.97 – 0.89 (m, 4H), 0.86 (t, J = 7.3 Hz, 6H).

^{13}C NMR (126 MHz, $CDCl_3$) δ 145.5, 134.0, 136.7, 133.6, 129.7, 129.4, 123.5, 122.2, 121.8, 119.7, 119.5, 107.9, 45.0, 31.3, 28.9, 26.2, 22.5, 14.0 (1 signal ($2C_{Ar}$) was not found due to overlapping peaks).

Anal. Calcd for $C_{42}H_{42}N_4O_4$: C, 75.65; H, 6.35; N, 8.40. Found: C, 75.65; H, 6.45; N, 8.71.

*2.2. 5,11-Dihexyl-6,12-bis(4-methoxyphenyl)-2,8-dinitro-5,11-dihydroindolo[3,2-b]carbazole (**2b**)*

Orange crystals (1.16 g, 80% yield), mp 319–320 °C.

^{1}H NMR (500 MHz, $CDCl_3$) δ 8.27 (dd, *J* = 9.0, 2.3 Hz, 2H), 7.57 – 7.52 (m, 4H), 7.45 (d, *J* = 2.2 Hz, 2H), 7.29 – 7.26 (m, 6H), 4.04 (s, 6H), 4.00 – 3.94 (m, 4H), 1.59 – 1.56 (m, 4H), 1.26 – 1.20 (m, 4H), 1.17 – 1.10 (m, 4H), 1.02 – 0.95 (m, 4H), 0.86 (t, *J* = 7.3 Hz, 6H).

^{13}C NMR (126 MHz, $CDCl_3$) δ 160.5, 145.5, 139.9, 133.9, 130.8, 128.4, 124.0, 122.3, 121.6, 119.6, 119.4, 115.3, 107.8, 55.8, 44.9, 31.3, 28.9, 26.4, 22.5, 13.9.

Anal. Calcd for $C_{44}H_{46}N_4O_6$: C,72.71; H, 6.38; N, 7.71. Found: C,72.42; H, 6.36; N, 7.99.

*2.3. 5,11-Dihexyl-6,12-bis(4-isopropylphenyl)-2,8-dinitro-5,11-dihydroindolo[3,2-b]carbazole (**2c**)*

Orange crystals (1.31 g, 87% yield), mp 315–316 °C.

^{1}H NMR (400 MHz, $CDCl_3$) δ 8.26 (dd, *J* = 9.1, 2.2 Hz, 2H), 7.64 – 7.50 (m, 8H), 7.30 (d, *J* = 2.2 Hz, 2H), 7.26 (d, *J* = 9.1 Hz, 2H), 3.99 – 3.94 (m, 4H), 3.24 – 3.12 (m, 2H), 1.64 – 1.54 (m, 4H), 1.49 (d, *J* = 6.9 Hz, 12H), 1.27 – 1.19 (m, 4H), 1.17 – 1.08 (m, 4H), 0.98 – 0.89 (m, 4H), 0.86 (t, *J* = 7.2 Hz, 6H).

^{13}C NMR (126 MHz, $CDCl_3$) δ 150.6, 145.4, 139.9, 133.9, 133.6, 129.6, 127.7, 123.8, 122.2, 121.6, 119.7, 119.7, 107.7, 45.0, 34.4, 31.3, 29.0, 26.3, 24.2, 22.6, 13.9.

Anal. Calcd for $C_{48}H_{54}N_4O_4$: C, 76.77; H, 7.25; N, 7.46. Found: C, 76.52; H, 7.44; N, 7.22.

*2.4. 6,12-Bis(4-bromophenyl)-5,11-dihexyl-2,8-dinitro-5,11-dihydroindolo[3,2-b]carbazole (**2d**)*

Dark yellow crystals (1.47 g, 89% yield), mp > 360 °C.

^{1}H NMR (400 MHz, $CDCl_3$) δ 8.30 (dd, *J* = 9.1, 1.8 Hz, 2H), 7.90 (d, *J* = 8.1 Hz, 4H), 7.55 (d, *J* = 8.0 Hz, 4H), 7.45 (d, *J* = 1.8 Hz, 2H), 7.30 (d, *J* = 9.1 Hz, 2H), 3.97 – 3.86 (m, 4H), 1.56 – 1.50 (m, 4H), 1.30 – 1.22 (m, 4H), 1.18 – 1.09 (m, 4H), 1.03 – 0.94 (m, 4H), 0.89 (t, *J* = 7.2 Hz, 6H).

^{13}C NMR (126 MHz, $CDCl_3$) δ 145.5, 140.2, 135.5, 133.6, 133.0, 131.5, 123.7, 123.4, 122.0, 122.0, 119.3, 118.6, 108.2, 45.1, 31.4, 29.0, 26.4, 22.6, 14.0.

Anal. Calcd for $C_{42}H_{40}Br_2N_4O_4$: C, 61.18; H, 4.89; N, 6.79. Found: C, 61.08; H, 4.61; N, 7.05.

*2.5. 6,12-Bis(benzo[b]thiophen-2-yl)-5,11-dihexyl-2,8-dinitro-5,11-dihydroindolo[3,2-b]carbazole (**2e**)*

Light orange crystals (1.09 g, 64% yield), mp 335–336 °C.

^{1}H NMR (400 MHz, $CDCl_3$) δ 8.29 (dd, *J* = 9.1, 2.2 Hz, 2H), 8.08 – 7.94 (m, 4H), 7.71 (d, *J* = 2.1 Hz, 2H), 7.65 (d, *J* = 6.9 Hz, 2H), 7.61 – 7.53 (m, 4H), 7.33 (d, *J* = 9.1 Hz, 2H), 4.22 – 3.99 (m, 2H), 1.86 – 1.63 (m, 4H), 1.15 – 0.93 (m, 10H), 0.91 – 0.82 (m, 2H), 0.74 (t, *J* = 7.1 Hz, 6H).

^{13}C NMR (126 MHz, $CDCl_3$) δ 145.6, 140.8, 140.6, 139.8, 136.7, 134.8, 125.8, 125.7, 125.4, 124.5, 124.2, 122.7, 122.2, 121.5, 119.8, 112.7, 108.3, 45.3, 31.3, 29.6, 26.4, 22.5, 13.9.

Anal. Calcd for $C_{46}H_{42}N_4S_2O_4$: C, 70.93; H, 5.43; N, 7.19. Found: C, 70.63; H, 5.28; N, 7.23.

*2.6. 5,11-Dihexyl-2,8-dinitro-6,12-di(thiophen-2-yl)-5,11-dihydroindolo[3,2-b]carbazole (**2f**)*

Orange crystals (1.14 g, 84% yield), mp 303–304 °C.

^{1}H NMR (400 MHz, $CDCl_3$) δ 8.33 (dd, *J* = 9.1, 2.2 Hz, 2H), 7.83 (d, *J* = 5.2 Hz, 2H), 7.54 – 7.47 (m, 4H), 7.42 – 7.36 (m, 2H), 7.34 (d, *J* = 9.1 Hz, 2H), 4.23 – 3.88 (m, 4H), 1.76 – 1.61 (m, 4H), 1.32 – 1.17 (m, 8H), 1.15 – 1.05 (m, 4H), 0.89 (t, *J* = 7.0 Hz, 6H).

^{13}C NMR (126 MHz, $CDCl_3$) δ 145.4, 140.4, 136.3, 134.9, 128.6, 128.4, 128.3, 125.1, 122.1, 121.6, 119.6, 112.5, 108.2, 45.0, 31.3, 29.3, 26.4, 22.5, 14.0.

Anal. Calcd for $C_{38}H_{38}N_4O_4S_2$: C, 67.23; H, 5.64; N, 8.25. Found: C, 67.24; H, 5.72; N, 8.21.

*2.7. 6,12-Bis(4-fluorophenyl)-5,11-dihexyl-2,8-dinitro-5,11-dihydroindolo[3,2-b]carbazole (**2g**)*

Yellow powder (1.22 g, 87% yield), mp > 360 °C.

^{1}H NMR (400 MHz, $CDCl_3$) δ 8.30 (dd, *J* = 9.1, 2.3 Hz, 2H), 7.69 – 7.61 (m, 4H), 7.50 – 7.42 (m, 6H), 7.30 (d, *J* = 9.1 Hz, 2H), 4.04 – 3.80 (m, 4H), 1.60 – 1.51 (m, 4H), 1.31 – 1.20 (m, 4H), 1.19 – 1.10 (m, 4H), 1.03 – 0.94 (m, 4H), 0.87 (t, *J* = 7.2 Hz, 6H).

^{13}C NMR (126 MHz, $CDCl_3$) δ 163.43 (d, J_{CF} = 250.6 Hz), 145.6, 140.2, 133.9, 132.5 (d, J_{CF} = 3.7 Hz), 131.6 (d, J_{CF} = 7.9 Hz), 123.8, 122.1, 121.9, 119.3, 118.8, 116.9 (d, J_{CF} = 21.5 Hz), 108.1, 45.0, 31.4, 28.9, 26.4, 22.5, 13.9.

Anal. Calcd for $C_{42}H_{40}F_2N_4O_4$:C, 71.78; H, 5.74; N, 7.97. Found: C, 71.49; H, 5.58; N, 8.27.

*2.8. 5,11-Dihexyl-6,12-bis(4-(hexyloxy)phenyl)-2,8-dinitro-5,11-dihydroindolo[3,2-b]carbazole (**2h**)*

Orange crystals (1.40 g, 81% yield), mp 255–256 °C.

^{1}H NMR (400 MHz, $CDCl_3$) δ 8.27 (dd, *J* = 9.1, 2.2 Hz, 2H), 7.52 (d, *J* = 8.5 Hz, 4H), 7.48 (d, *J* = 2.2 Hz, 2H), 7.31 – 7.21 (m, 6H), 4.19 (t, *J* = 6.6 Hz, 4H), 4.06 – 3.82 (m, 4H), 1.99 – 1.89 (m, 4H), 1.65 – 1.50 (m, 8H), 1.48 – 1.36 (m, 8H), 1.27 – 1.19 (m, 4H), 1.17 – 1.09 (m, 4H), 1.04 – 0.94 (m, 10H), 0.86 (t, *J* = 7.2 Hz, 6H).

^{13}C NMR (126 MHz, $CDCl_3$) δ 160.0, 145.5, 139.9, 133.9, 130.7, 128.2, 124.0, 122.3, 121.6, 119.6, 119.5, 115.8, 107.8, 68.6, 44.9, 31.6, 31.3, 29.3, 28.9, 26.4, 25.8, 22.6, 22.5, 14.1, 13.9.

Anal. Calcd for $C_{54}H_{66}N_4O_6$: C, 74.80; H, 7.67; N, 6.46. Found: C, 74.54; H, 8.05; N, 6.18.

*2.9. 5,11-Diheptyl-2,8-dinitro-6,12-di(thiophen-2-yl)-5,11-dihydroindolo[3,2-b]carbazole (**2i**)*

Orange crystals (1.07 g, 76% yield), mp 278–279 °C.

^{1}H NMR (500 MHz, $CDCl_3$) δ 8.33 (dd, *J* = 9.1, 2.3 Hz, 2H), 7.83 (dd, *J* = 5.2, 1.1 Hz, 2H), 7.52 – 7.45 (m, 4H), 7.40 – 7.36 (m, 2H), 7.34 (d, *J* = 9.1 Hz, 2H), 4.17 – 3.95 (m, 4H), 1.74 – 1.63 (m, 4H), 1.34 – 1.19 (m, 12H), 1.15 – 1.04 (m, 4H), 0.89 (t, *J* = 7.1 Hz, 6H).

^{13}C NMR (126 MHz, $CDCl_3$) δ 145.4, 140.4, 136.3, 134.9, 128.6, 128.4, 128.3, 125.1, 122.1, 121.6, 119.6, 112.5, 108.2, 45.0, 31.7, 29.4, 28.8, 26.7, 22.5, 14.0.

Anal. Calcd for $C_{40}H_{42}N_4O_4S_2$: C, 67.96; H, 5.99; N, 7.93. Found: C, 67.77; H, 5.88; N, 7.82.

*2.10. 2,8-Dinitro-5,11-dipentadecyl-6,12-di(thiophen-2-yl)-5,11-dihydroindolo[3,2-b]carbazole (**2j**)*

Orange crystals (1.34 g, 72% yield), mp 202–203 °C.

^{1}H NMR (400 MHz, $CDCl_3$) δ 8.33 (dd, *J* = 9.1, 2.3 Hz, 2H), 7.83 (dd, *J* = 5.2, 1.0 Hz, 2H), 7.51 – 7.46 (m, 4H), 7.41 – 7.36 (m, 2H), 7.34 (d, *J* = 9.1 Hz, 2H), 4.28 – 3.92 (m, 4H), 1.73 – 1.63 (m, 4H), 1.35 – 1.18 (m, 44H), 1.14 – 1.05 (m, 4H), 0.87 (t, *J* = 6.8 Hz, 6H).

^{13}C NMR (126 MHz, $CDCl_3$) δ 145.4, 140.4, 136.3, 134.9, 128.6, 128.4, 128.3, 125.1, 122.1, 121.6, 119.6, 112.5, 108.2, 45.0, 31.9, 29.7, 29.6, 29.6, 29.5, 29.5, 29.4, 29.3, 29.2, 26.8, 22.7, 14.1 (2 signals ($4C_{Alkyl}$) were not found due to overlapping peaks).

Anal. Calcd for $C_{56}H_{74}N_4S_2O_4$: C, 72.22; H, 8.01; N, 6.02. Found: C, 72.25; H, 8.17; N, 5.87.

*2.11. 5,11-Dihexyl-6,12-dinitro-5,11-dihydroindolo[3,2-b]carbazole (**9a**)*

Deep red needles (576 mg, 56% yield), mp 189–190 °C.

^{1}H NMR (500 MHz, $CDCl_3$) δ 7.92 (d, *J* = 8.0 Hz, 2H), 7.64 – 7.59 (m, 2H), 7.48 (d, *J* = 8.3 Hz, 2H), 7.35 – 7.29 (m, 2H), 4.27 – 4.20 (m, 4H), 1.83 – 1.73 (m, 4H), 1.47 – 1.28 (m, 12H), 0.92 (t, *J* = 6.8 Hz, 6H).

^{13}C NMR (126 MHz, $CDCl_3$) δ 142.8, 129.0, 128.5, 125.0, 121.2, 120.7, 117.7, 116.8, 109.6, 44.5, 31.3, 29.5, 26.6, 22.5, 13.9.

Anal. Calcd for $C_{30}H_{34}N_4O_4$: C, 70.02; H, 6.66; N, 10.89. Found: C, 70.09; H, 6.81; N, 10.69.

*2.12. 5,11-Diethyl-6,12-dinitro-5,11-dihydroindolo[3,2-b]carbazole (**9b**)*

Red crystals (483 mg, 60% yield), mp 318–319 °C.

^{1}H NMR (400 MHz, $CDCl_3$) δ 7.92 (d, *J* = 8.0 Hz, 2H), 7.67 – 7.60 (m, 2H), 7.50 (d, *J* = 8.4 Hz, 2H), 7.36 – 7.31 (m, 2H), 4.34 (q, *J* = 7.2 Hz, 4H), 1.43 (t, *J* = 7.2 Hz, 6H).

Anal. Calcd for $C_{22}H_{18}N_4O_4$: C, 65.66; H, 4.51; N, 13.92. Found: C, 65.25; H, 4.32; N, 13.93.

*2.13. 5,11-Dibutyl-6,12-dinitro-5,11-dihydroindolo[3,2-b]carbazole (**9c**)*

Red whiskers (540 mg, 59% yield), mp 228–229 °C.

^{1}H NMR (400 MHz, $CDCl_3$) δ 7.91 (d, *J* = 8.0 Hz, 2H), 7.64 – 7.59 (m, 2H), 7.49 (d, *J* = 8.3 Hz, 2H), 7.34 – 7.28 (m, 2H), 4.28 – 4.22 (m, 4H), 1.81 – 1.71 (m, 4H), 1.47 – 1.35 (m, 4H), 0.97 (t, *J* = 7.4 Hz, 6H).

Anal. Calcd for $C_{26}H_{26}N_4O_4$: C, 68.11; H, 5.72; N, 12.22. Found: C, 67.73; H, 5.48; N, 12.09.

*2.14. 5,11-Didodecyl-6,12-dinitro-5,11-dihydroindolo[3,2-b]carbazole (**9d**)*

Bright red whiskers (780 mg, 57% yield), mp 128–129 °C.

^{1}H NMR (500 MHz, $CDCl_3$) δ 7.91 (d, *J* = 8.0 Hz, 2H), 7.64 – 7.59 (m, 2H), 7.48 (d, *J* = 8.4 Hz, 2H), 7.34 – 7.29 (m, 2H), 4.27 – 4.20 (m, 4H), 1.81 – 1.72 (m, 4H), 1.37 – 1.23 (m, 36H), 0.87 (t, *J* = 7.0 Hz, 6H).

^{13}C NMR (126 MHz, $CDCl_3$) δ 142.8, 129.0, 128.5, 125.0, 121.2, 120.7, 117.7, 116.8, 109.6, 44.5, 31.9, 29.6, 29.54, 29.49, 29.4, 29.3, 29.23, 29.17, 26.9, 22.7, 14.1.

Anal. Calcd for $C_{42}H_{58}N_4O_4$: C, 73.87; H, 8.56; N, 8.20. Found: C, 73.97; H, 8.54; N, 8.12.

3. General procedure for synthesis of 2-nitro and 6-nitro ICZ derivatives 3a,b and 10a,b.

A solution of acetyl nitrate, prepared in situ from white fuming HNO_3 (0.14 ml, 3.25 mmol) and acetic anhydride (0.37 ml, 3.9 mmol), in dry CH_2Cl_2 (10 ml) was added dropwise to the stirring solution of indolo[3,2-*b*]carbazole **1a** (1.44 g, 2.5 mmol) or **1b** (1.59 g, 2.5 mmol) in dry CH_2Cl_2 (60 ml) at −20 °C. The resulting dark-red mixture was stirred at this temperature for 15 min and at room temperature for 1h. A saturated solution of $NaHCO_3$ (25 ml) was added to the reaction mixture and well stirred for 0.5 h. The organic layer was separated, washed with water (50 ml), dried with $MgSO_4$ and concentrated under vacuum. The residue was purified by column chromatography on silica gel (benzene–hexane, v/v 3:1) to afford target product **3a** or **3b**, while unreacted compound **1a** (810 mg, 1.4 mmol) or **1b** (770 mg, 1.21 mmol) was returned.

The synthesis of 6-nitro derivatives **10a**,**b** was carried out in accordance with the above procedure starting from indolo[3,2-*b*]carbazoles **8a** (1.7 g, 4 mmol) or **8b** (1.25 g, 4 mmol) in dry CH_2Cl_2 (100

ml), white fuming HNO_3 (0.23 ml, 5.3 mmol) and acetic anhydride (0.61 ml, 6.4 mmol) in dry CH_2Cl_2 (15 ml). Full conversion of compounds **8a,b** was observed in these experiments.

*3.1. 5,11-Dihexyl-2-nitro-6,12-diphenyl-5,11-dihydroindolo[3,2-b]carbazole (**3a**)*

Orange needles (370 mg, 54% yield, based on reacted **1a**), mp 207–208 °C.

^{1}H NMR (500 MHz, $CDCl_3$) δ 8.23 (dd, *J* = 9.0, 2.3 Hz, 1H), 7.77 – 7.63 (m, 10H), 7.39 – 7.35 (m, 2H), 7.30 (d, *J* = 8.2 Hz, 1H), 7.21 (d, *J* = 9.1 Hz, 1H), 6.87 – 6.82 (m, 1H), 6.50 (d, *J* = 7.9 Hz, 1H), 4.03 – 3.62 (m, 4H), 1.60 – 1.49 (m, 4H), 1.26 – 1.18 (m, 4H), 1.16 – 1.07 (m, 4H), 0.95 – 0.82 (m, 10H).

^{13}C NMR (126 MHz, $CDCl_3$) δ 145.3, 142.6, 139.6, 137.9, 137.6, 133.0, 132.8, 130.3, 129.9, 129.5, 129.1, 128.9, 128.5, 125.9, 123.8, 122.6, 122.6, 122.5, 122.2, 121.2, 119.3, 118.9, 118.6, 118.3, 108.5, 107.4, 44.9, 44.4, 31.38, 31.35, 28.80, 28.75, 26.29, 26.25, 22.51, 22.49, 13.97, 13.96.

Anal. Calcd for $C_{42}H_{43}N_3O_2$: C, 81.13; H, 6.97; N, 6.76 Found: C, 80.93; H, 6.96; N, 6.85.

*3.2. 5,11-Dhexyl-6,12-bis(4-methoxyphenyl)-2-nitro-5,11-dihydroindolo[3,2-b]carbazole (**3b**)*

Orange needles (440 mg, 50% yield, based on reacted **1b**), mp 193–194 °C.

^{1}H NMR (400 MHz, $CDCl_3$) δ 8.23 (dd, *J* = 9.1, 2.2 Hz, 1H), 7.57 – 7.52 (m, 4H), 7.45 (d, *J* = 2.2 Hz, 1H), 7.38 (t, *J* = 7.4 Hz, 1H), 7.30 (d, *J* = 8.2 Hz, 1H), 7.25 – 7.15 (m, 5H), 6.88 (t, *J* = 7.4 Hz, 1H), 6.63 (d, *J* = 7.9 Hz, 1H), 4.03 (s, 3H), 4.00 (s, 3H), 3.97 – 3.84 (m, 4H), 1.60 – 1.48 (m, 4H), 1.28 – 1.18 (m, 4H), 1.17 – 1.08 (m, 4H), 1.02 – 0.91 (m, 4H), 0.88 – 0.84 (m, 6H).

^{13}C NMR (126 MHz, $CDCl_3$) δ 160.2, 159.8, 145.3, 142.6, 139.5, 133.3, 133.2, 131.3, 130.9, 129.8, 129.5, 125.8, 124.2, 122.8, 122.7, 122.6, 122.6, 121.1, 119.3, 118.6, 118.3, 115.1, 114.5, 108.4, 107.4, 55.8, 55.5, 44.9, 44.4, 31.41, 31.38, 28.82, 28.76, 26.4, 22.55, 13.97, 13.95 (3 signals ($1C_{Ar}$ + $2C_{Aklyl}$) were not found due to overlapping peaks).

Anal. Calcd for $C_{44}H_{47}N_3O_4$: C, 77.50; H, 6.95; N, 6.16. Found: C, 77.54; H, 6.98; N, 6.21.

*3.3. 5,11-Dihexyl-6-nitro-5,11-dihydroindolo[3,2-b]carbazole (**10a**)*

Dark red plates (1.17 g, 62% yield), mp 82–83 °C.

^{1}H NMR (500 MHz, $CDCl_3$) δ 8.20 (d, *J* = 7.7 Hz, 1H), 8.15 (s, 1H), 8.02 (d, *J* = 8.0 Hz, 1H), 7.57 – 7.51 (m, 2H), 7.46 – 7.42 (m, 2H), 7.33 – 7.28 (m, 1H), 7.25 – 7.21 (m, 1H), 4.41 (t, *J* = 7.3 Hz, 2H), 4.26 – 4.21 (m, 2H), 1.96 – 1.89 (m, 2H), 1.77 – 1.71 (m, 2H), 1.48 – 1.41 (m, 2H), 1.37 – 1.26 (m, 10H), 0.89 – 0.84 (m, 6H).

^{13}C NMR (101 MHz, $CDCl_3$) δ 142.8, 141.7, 135.4, 128.36, 128.35, 127.0, 125.9, 125.7, 122.0, 121.7, 120.2, 119.5, 119.1, 118.6, 113.8, 109.3, 108.8, 102.0, 44.6, 43.2, 31.6, 31.4, 29.3, 28.8, 27.0, 26.7, 22.6, 22.5, 14.03, 13.98.

Anal. Calcd for $C_{30}H_{35}N_3O_2$: C, 76.73; H, 7.51; N, 8.95. Found: C, 77.05; H, 7.46; N, 9.09.

*5,11-Diethyl-6-nitro-5,11-dihydroindolo[3,2-b]carbazole (**10b**)*

Dark red crystals (985 mg, 56% yield), mp 179–180 °C.

^{1}H NMR (400 MHz, DMSO-d_6) δ 8.85 (s, 1H), 8.43 (d, J = 7.7 Hz, 1H), 7.83 (d, J = 8.1 Hz, 1H), 7.75 (d, J = 8.3 Hz, 1H), 7.72 (d, J = 8.3 Hz, 1H), 7.62 – 7.57 (m, 2H), 7.39 – 7.33 (m, 1H), 7.29 – 7.23 (m, 1H), 4.65 (q, J = 7.0 Hz, 2H), 4.31 (q, J = 7.1 Hz, 2H), 1.41 (t, J = 7.1 Hz, 3H), 1.26 (t, J = 7.1 Hz, 3H).

^{13}C NMR (126 MHz, $CDCl_3$) δ 142.2, 141.1, 134.7, 128.2, 127.0, 126.9, 125.8, 125.4, 122.1, 121.6, 120.1, 119.5, 119.0, 118.6, 113.7, 109.0, 108.5, 101.8, 39.1, 37.5, 14.3, 13.5.

Anal. Calcd for $C_{22}H_{19}N_3O_2$: C, 73.93; H, 5.36; N, 11.76. Found: C, 73.94; H, 5.44; N, 11.81.

4. General procedure for reduction of 2,8-dinitro and 2-nitro ICZ derivatives 2a–f and 3a,b.

Zinc powder (1.3 g, 20 mmol) was added to the stirring solution of 2,8-dinitro derivative **2** (1 mmol) in THF (40 ml) at room temperature. Concentrated HCl (5.2 ml of 12 M) was added dropwise to this suspension and the resulting mixture was stirred under reflux for 1 h. The reaction mixture was neutralized with a 5% solution of NaOH (100 ml) and extracted with CH_2Cl_2 (3 × 20 ml). The extract was dried with Na_2SO_4 and then evaporated under vacuum to afford crude 2,8-diamino derivative **4**. Reduction of 2-nitro derivative **3a** (310 mg, 0.5 mmol) or **3b** (340 mg, 0.5 mmol) was carried out in accordance with the above procedure using zinc powder (330 mg, 5 mmol) and concentrated HCl (1.3 ml of 12 M) in THF (10 ml) to give crude 2-amino derivative **5a** or **5b**, respectively. Amino derivatives **4** and **5** were immediately converted into corresponding phtalimides **6** and **7** without any purification.

5. General procedure for synthesis of phtalimides 6a–f and 7a,b.

Crude diamine **4** and phthalic anhydride (450 mg, 3 mmol) were dissolved in dry DMF (10 ml) and the solution was heated at 100 °C for 10 min. Acetyl chloride (0.44 ml, 6 mmol) was added to this hot solution and then it was cooled to room temperature. The analytically pure form of product **6** was separated by filtration, washed with warm EtOH (2 × 5 ml) and dried at 120 °C. Crude amines **5a,b** were converted into derivatives **7a**,**b** in accordance with the above procedure using phthalic anhydride (110 mg, 0.75 mmol) in dry DMF (3 ml) and acetyl chloride (0.11 ml, 1.5 mmol).

*5.1. 2,2'-(5,11-Dihexyl-6,12-diphenyl-5,11-dihydroindolo[3,2-b]carbazole-2,8-diyl)bis(isoindoline-1,3-dione) (**6a**)*

Yellow crystals (540 mg, 62% yield), mp 298–299 °C.

^{1}H NMR (400 MHz, $CDCl_3$) δ 7.96 – 7.90 (m, 4H), 7.81 – 7.74 (m, 4H), 7.73 – 7.66 (m, 4H), 7.64 – 7.58 (m, 4H), 7.55 – 7.48 (m, 2H), 7.37 – 7.29 (m, 4H), 6.46 (d, *J* = 1.4 Hz, 2H), 3.96 – 3.65 (m, 4H), 1.59 – 1.48 (m, 4H), 1.25 – 1.17 (m, 4H), 1.15 – 1.06 (m, 4H), 0.95 – 0.81 (m, 10H).

^{13}C NMR (101 MHz, $CDCl_3$) δ 167.6, 141.9, 138.1, 134.0, 132.9, 132.0, 130.3, 129.1, 128.2, 124.0, 123.5, 123.1, 122.8, 121.6, 121.4, 118.4, 108.5, 44.6, 31.4, 28.7, 26.3, 22.5, 14.0.

HRMS (+ESI): m/z calculated for $C_{58}H_{50}N_4O_4$: 866.3827 $[M]^+$, found 866.3829 $[M]^+$.

*5.2. 2,2'-(5,11-Dihexyl-6,12-bis(4-methoxyphenyl)-5,11-dihydroindolo[3,2-b]carbazole-2,8-diyl)bis(isoindoline-1,3-dione) (**6b**)*

Yellow powder (585 mg, 63% yield), mp 340–341 °C.

^{1}H NMR (400 MHz, $CDCl_3$) δ 7.95 – 7.89 (m, 4H), 7.81 – 7.73 (m, 4H), 7.59 (d, *J* = 8.5 Hz, 4H), 7.35 (d, *J* = 0.9 Hz, 4H), 7.16 (d, *J* = 8.6 Hz, 4H), 6.55 (s, 2H), 3.93 – 3.85 (m, 10H), 1.61 – 1.49 (m, 4H), 1.27 – 1.18 (m, 4H), 1.17 – 1.07 (m, 4H), 1.01 – 0.91 (m, 4H), 0.85 (t, *J* = 7.2 Hz, 6H).

^{13}C NMR (101 MHz, $CDCl_3$) δ 167.6, 159.6, 141.9, 134.1, 133.2, 132.1, 131.4, 130.2, 123.8, 123.4, 123.3, 123.3, 121.7, 121.3, 118.0, 114.8, 108.4, 55.5, 44.6, 31.5, 28.8, 26.5, 22.6, 14.0.

HRMS (+ESI): m/z calculated for $C_{60}H_{54}N_4O_6$: 926.4038 $[M]^+$, found 926.4051 $[M]^+$.

*5.3. 2,2'-(5,11-Dihexyl-6,12-bis(4-isopropylphenyl)-5,11-dihydroindolo[3,2-b]carbazole-2,8-diyl)bis(isoindoline-1,3-dione) (**6c**)*

Yellow powder (610 mg, 64% yield), mp 315–316 °C.

^{1}H NMR (400 MHz, $CDCl_3$) δ 7.93 – 7.88 (m, 4H), 7.80 – 7.74 (m, 4H), 7.61 (d, *J* = 8.0 Hz, 4H), 7.48 (d, *J* = 8.0 Hz, 4H), 7.38 – 7.33 (m, 4H), 6.32 (s, 2H), 3.99 – 3.89 (m, 4H), 3.08 – 2.93 (m, 2H), 1.70 – 1.50 (m, 4H), 1.29 – 1.19 (m, 16H), 1.17 – 1.09 (m, 4H), 0.98 – 0.90 (m, 4H), 0.86 (t, *J* = 7.2 Hz, 6H).

^{13}C NMR (101 MHz, $CDCl_3$) δ 167.6, 149.0, 141.7, 135.5, 134.0, 132.7, 131.9, 130.2, 127.1, 123.6, 123.3, 123.2, 123.1, 121.5, 121.4, 118.3, 108.2, 44.7, 34.2, 31.4, 28.9, 26.4, 24.1, 22.6, 14.0.

HRMS (+ESI): m/z calculated for $C_{64}H_{62}N_4NaO_4$: 973.4670 $[M+Na]^+$, found 973.4663 $[M+Na]^+$.

*5.4. 2,2'-(6,12-Bis(4-bromophenyl)-5,11-dihexyl-5,11-dihydroindolo[3,2-b]carbazole-2,8-diyl)bis(isoindoline-1,3-dione) (**6d**)*

Dark yellow powder (625 mg, 61% yield), mp > 360 °C.

^{1}H NMR (400 MHz, $CDCl_3$) δ 8.01 – 7.94 (m, 4H), 7.88 – 7.75 (m, 8H), 7.59 (d, *J* = 8.3 Hz, 4H), 7.42 (dd, *J* = 8.7, 1.9 Hz, 2H), 7.38 (d, *J* = 8.7 Hz, 2H), 6.51 (d, *J* = 1.8 Hz, 2H), 3.90 – 3.85 (m, 4H), 1.61 – 1.51 (m, 4H), 1.32 – 1.21 (m, 4H), 1.19 – 1.09 (m, 4H), 1.04 – 0.94 (m, 4H), 0.90 (t, *J* = 7.3 Hz, 6H).

^{13}C NMR (101 MHz, $CDCl_3$) δ 167.6, 146.6, 141.7, 137.1, 134.2, 132.8, 132.5, 132.1, 132.0, 124.0, 123.6, 122.7, 122.5, 122.1, 120.9, 117.2, 108.6, 44.7, 31.4, 28.8, 26.4, 22.6, 14.0.

HRMS (+ESI): m/z calculated for $C_{58}H_{48}N_4Br_2O_4$: 1022.2037 $[M]^+$, found 1022.2048 $[M]^+$.

*5.5. 2,2'-(6,12-Bis(benzo[b]thiophen-2-yl)-5,11-dihexyl-5,11-dihydroindolo[3,2-b]carbazole-2,8-diyl)bis(isoindoline-1,3-dione) (**6e**)*

Yellow powder (520 mg, 53% yield), mp > 360 °C.

^{1}H NMR (400 MHz, $CDCl_3$) δ 7.98 – 7.92 (m, 4H), 7.83 – 7.76 (m, 4H), 7.62 (dd, *J* = 5.2, 1.0 Hz, 2H), 7.40 (d, *J* = 1.0 Hz, 4H), 7.38 – 7.33 (m, 2H), 7.29 (dd, *J* = 5.1, 3.4 Hz, 2H), 6.61 (s, 2H), 4.12 – 3.73 (m, 4H), 1.82 – 1.59 (m, 4H), 1.36 – 1.15 (m, 8H), 1.13 – 1.03 (m, 4H), 0.87 (t, *J* = 7.1 Hz, 6H).

^{13}C NMR (101 MHz, $CDCl_3$) δ 167.5, 141.9, 141.0, 140.0, 138.7, 134.1, 134.0, 131.9, 127.5, 125.6, 124.52, 124.48, 124.1, 123.7, 123.4, 122.6, 122.4, 122.4, 121.4, 111.2, 108.9, 44.9, 31.3, 29.4, 26.5, 22.5, 13.9.

HRMS (+ESI): m/z calculated for $C_{62}H_{50}N_4S_2O_4$: 978.3268 $[M]^+$, found 978.3264 $[M]^+$.

*5.6. 2,2'-(5,11-Dihexyl-6,12-di(thiophen-2-yl)-5,11-dihydroindolo[3,2-b]carbazole-2,8-diyl)bis(isoindoline-1,3-dione) (**6f**)*

Yellow powder (660 mg, 75% yield), mp 329–330 °C.

^{1}H NMR (400 MHz, $CDCl_3$) δ 7.96 (d, *J* = 7.7 Hz, 2H), 7.92 (d, *J* = 7.2 Hz, 2H), 7.84 – 7.78 (m, 4H), 7.74 – 7.68 (m, 4H), 7.67 – 7.62 (m, 2H), 7.43 – 7.33 (m, 8H), 6.89 (s, 2H), 4.10 – 3.91 (m, 4H), 1.82 – 1.64 (m, 4H), 1.16 – 0.79 (m, 12H), 0.74 (t, *J* = 7.1 Hz, 6H).

^{13}C NMR (101 MHz, $CDCl_3$) δ 167.6, 141.9, 138.2, 135.5, 134.2, 134.0, 132.1, 128.4, 127.7, 127.4, 124.3, 123.5, 122.6, 122.3, 121.3, 111.1, 108.7, 44.7, 31.4, 29.1, 26.5, 22.5, 13.9.

HRMS (+ESI): m/z calculated for $C_{54}H_{47}N_4S_2O_4$: 879.3044 $[M+H]^+$, found 879.3033 $[M+H]^+$.

*5.7. 2-(5,11-Dihexyl-6,12-diphenyl-5,11-dihydroindolo[3,2-b]carbazol-2-yl)isoindoline-1,3-dione (**7a**)*

Yellow crystals (320 mg, 89% yield), mp 203–204 °C.

^{1}H NMR (500 MHz, $CDCl_3$) δ 7.95 – 7.91 (m, 2H), 7.79 – 7.75 (m, 2H), 7.63 (ddd, *J* = 27.3, 17.8, 7.4 Hz, 10H), 7.50 (t, *J* = 7.4 Hz, 1H), 7.36 – 7.29 (m, 3H), 6.82 (t, *J* = 5.9 Hz, 1H), 6.53 (d, *J* = 8.1 Hz,

1H), 6.45 (s, 1H), 3.92 – 3.68 (m, 4H), 1.60 – 1.47 (m, 4H), 1.25 – 1.18 (m, 4H), 1.15 – 1.06 (m, 4H), 0.92 – 0.82 (m, 10H).

^{13}C NMR (101 MHz, C_6D_6) δ 167.1, 143.2, 142.0, 139.3, 138.9, 133.22, 133.17, 133.0, 132.2, 130.8, 130.6, 129.2, 129.0, 125.7, 124.4, 123.7, 123.6, 122.9, 121.5, 118.7, 118.5, 118.4, 108.7, 108.5, 44.6, 44.4, 31.5, 31.5, 28.7, 28.6, 26.4, 26.4, 22.7, 22.6, 14.02, 13.95 (6 signals ($6C_{Ar}$) were not found due to overlapping peaks).

Anal. Calcd for $C_{50}H_{47}N_3O_2$: C, 83.19; H, 6.56; N, 5.82. Found: C, 83.33; H, 6.51; N, 5.88.

*5.8. 2-(5,11-Dihexyl-6,12-bis(4-methoxyphenyl)-5,11-dihydroindolo[3,2-b]carbazol-2-yl)isoindoline-1,3-dione (**7b**)*

Yellow powder (292 mg, 75% yield), mp 227–228 °C.

^{1}H NMR (500 MHz, C_6D_6) δ 7.63 – 7.55 (m, 5H), 7.48 – 7.43 (m, 2H), 7.38 – 7.34 (m, 1H), 7.32 (d, *J* = 2.0 Hz, 1H), 7.22 (d, *J* = 8.6 Hz, 2H), 7.19 (d, *J* = 8.7 Hz, 1H), 7.09 – 7.05 (m, 2H), 7.05 – 7.02 (m, 1H), 7.01 – 6.98 (m, 2H), 6.92 – 6.87 (m, 2H), 3.86 – 3.73 (m, 4H), 3.47 (s, 3H), 3.43 (s, 3H), 1.57 – 1.39 (m, 4H), 1.22 – 0.91 (m, 8H), 0.91 – 0.77 (m, 10H).

^{13}C NMR (101 MHz, C_6D_6) δ 167.1, 159.9, 159.88, 143.2, 142.0, 133.7, 133.4, 133.1, 132.2, 131.8, 131.6, 131.2, 130.8, 125.8, 125.6, 124.1, 123.9, 123.8, 123.5, 123.0, 122.9, 122.8, 121.3, 118.43, 118.40, 118.3, 115.0, 114.5, 108.6, 108.4, 54.8, 54.7, 44.64, 44.56, 31.6, 31.5, 28.8, 28.7, 26.61, 26.55, 22.8, 22.7, 14.00, 13.95.

Anal. Calcd for $C_{52}H_{51}N_3O_4$: C, 79.87; H, 6.57; N, 5.37. Found: C, 79.97; H, 6.52; N, 5.40.

6. General procedure for formylation of 6,12-dinitro ICZ derivatives 9a,b.

$SnCl_4$ (780 mg, 0.35 ml, 3 mmol) and dichloromethyl methyl ether (340 mg, 0.27 ml, 3 mmol) was successively added dropwise to the stirring solution of compound **9a** (515 mg, 1 mmol) or **9b** (400 mg, 1 mmol) in dry CH_2Cl_2 (30 ml) at 0 °C. The reaction mixture was stirred for 1h at 0 °C and another 1 h at room temperature. After that, it was poured into ice water (50 ml) and stirring was continued for 1 h. The organic layer was separated, washed successively with 5% solution of NaOH (15 ml) and water (30 ml), dried with $MgSO_4$ and evaporated under reduced pressure to give crude material as reddish residue, that was crystallized from DMF (15 ml) to give target product **12**. It was filtered, washed with EtOH (10 ml) and dried at 100 °C.

*6.1. 5,11-Dihexyl-6,12-dinitro-5,11-dihydroindolo[3,2-b]carbazole-2-carbaldehyde (**12a**)*

Dark orange crystals (288 mg, 53% yield), mp 158–159 °C.

^{1}H NMR (400 MHz, $CDCl_3$) δ 10.10 (s, 1H), 8.38 (d, *J* = 0.9 Hz, 1H), 8.19 (dd, *J* = 8.7, 1.3 Hz, 1H), 7.91 (d, *J* = 8.0 Hz, 1H), 7.69 – 7.63 (m, 1H), 7.58 (d, *J* = 8.7 Hz, 1H), 7.52 (d, *J* = 8.4 Hz, 1H), 7.37 – 7.32 (m, 1H), 4.36 – 4.20 (m, 4H), 1.87 – 1.71 (m, 4H), 1.46 – 1.27 (m, 13H), 0.94 – 0.83 (m, 6H).
^{13}C NMR (101 MHz, $CDCl_3$) δ 190.9, 146.0, 143.1, 130.0, 129.4, 129.1, 128.9, 128.2, 126.1, 125.9, 125.08, 121.3, 121.1, 117.84, 117.78, 117.5, 116.5, 110.2, 109.9, 45.0, 44.7, 31.29, 31.26, 29.6, 29.4, 26.6, 26.54, 22.46, 22.4, 13.91, 13.90.
Anal. Calcd for $C_{31}H_{34}N_4O_5$: C, 68.62; H, 6.32; N, 10.33. Found: C, 68.77; H, 6.38; N, 10.47.

*6.2. 5,11-Diethyl-6,12-dinitro-5,11-dihydroindolo[3,2-b]carbazole-2-carbaldehyde (**12b**)*
Red crystals (383 mg, 89% yield), mp 303–304 °C dec.
^{1}H NMR (400 MHz, $CDCl_3$) δ 10.12 (s, 1H), 8.40 (d, *J* = 0.8 Hz, 1H), 8.21 (dd, *J* = 8.6, 1.3 Hz, 1H), 7.92 (d, *J* = 8.2 Hz, 1H), 7.71 – 7.65 (m, 1H), 7.60 (d, *J* = 8.7 Hz, 1H), 7.54 (d, *J* = 8.4 Hz, 1H), 7.39 – 7.34 (m, 1H), 4.45 – 4.31 (m, 4H), 1.51 – 1.43 (m, 6H).
Anal. Calcd for $C_{23}H_{18}N_4O_5$: C, 64.18; H, 4.22; N, 13.02. Found: C, 63.86; H, 4.12; N, 12.91.

7. General procedure for bromination of 6,12-dinitro ICZ derivatives 9a,b.
Bromine (0.16 ml, 500 mg, 3.1 mmol) was added in one portion to compound **9a** (515 mg, 1 mmol) or **9b** (400 mg, 1 mmol) in dry CH_2Cl_2 (30 ml) and the solution was stirred at room temperature for 0.5h. The precipitate of product **13** was formed during this time. The reaction mixture was worked with 1% solution of Na_2SO_3 (30 ml) and intensively shaken, then CH_2Cl_2 was removed from the mixture in a rotary evaporator under reduced pressure. The solid material was filtered, washed with water and crystallized from DMF (15 ml). The analytically pure product **13** was filtered, washed with EtOH (10 ml) and dried at 100 °C.

*7.1. 2,8-Dibromo-5,11-dihexyl-6,12-dinitro-5,11-dihydroindolo[3,2-b]carbazole (**13a**)*
Dark red needles (620 mg, 92% yield), mp 209–210 °C.
^{1}H NMR (400 MHz, $CDCl_3$) δ 8.00 (d, *J* = 1.7 Hz, 2H), 7.72 (dd, *J* = 8.8, 1.8 Hz, 2H), 7.38 (d, *J* = 8.8 Hz, 2H), 4.28 – 4.14 (m, 4H), 1.85 – 1.65 (m, 4H), 1.58 – 1.26 (m, 12H), 0.89 (t, *J* = 6.9 Hz, 6H).
Anal. Calcd for $C_{30}H_{32}Br_2N_4O_4$: C, 53.59; H, 4.80; N, 8.33. Found: C, 53.62; H, 4.80; N, 8.25.

*7.2. 2,8-Dibromo-5,11-diethyl-6,12-dinitro-5,11-dihydroindolo[3,2-b]carbazole (**13b**)*
Red crystals (493 mg, 88% yield), mp 354–355 °C dec.

^{1}H NMR (400 MHz, $CDCl_3$) δ 8.01 (d, *J* = 1.7 Hz, 2H), 7.74 (dd, *J* = 8.8, 1.7 Hz, 2H), 7.39 (d, *J* = 8.9 Hz, 2H), 4.31 (q, *J* = 7.2 Hz, 4H), 1.42 (t, *J* = 7.2 Hz, 6H).

Anal. Calcd for $C_{22}H_{16}Br_2N_4O_4$: C, 47.17; H, 2.88; N, 10.00. Found: C, 47.18; H, 2.74; N, 10.06.

8. Procedure for synthesis of 2,8-diphenyl ICZ derivative 14a.

Compound **13a** (680 mg, 1 mmol), phenylboronic acid (370 mg, 3 mmol), solution of K_2CO_3 (3 ml of 2 M, 6 mmol) and a mixture of ethanol (15 ml) and toluene (15 ml) were placed to a two-necked round-bottomed flask, equipped with a condenser and magnetic stir bar. The apparatus was evacuated and filled with argon in several cycles and $Pd(PPh_3)_4$ (116 mg, 0.1 mmol) was added to the obtained mixture under an argon atmosphere. The reaction mixture was stirred under reflux for 3 h and then evaporated under vacuum. The residue was extracted with $CHCl_3$ (40 ml), then the extract was evaporated under vacuum and the solid was purified by flash column chromatography on silica gel ($CHCl_3$–hexane, v/v 5:1) to give desired product **14a**.

*8.1. 5,11-Dihexyl-6,12-dinitro-2,8-diphenyl-5,11-dihydroindolo[3,2-b]carbazole (**14a**)*

Orange crystals (505 mg, 75% yield), mp 247–248 °C.

^{1}H NMR (500 MHz, $CDCl_3$) δ 8.10 (d, *J* = 1.5 Hz, 2H), 7.87 (dd, *J* = 8.6, 1.7 Hz, 2H), 7.70 – 7.65 (m, 4H), 7.55 (d, *J* = 8.6 Hz, 2H), 7.53 – 7.47 (m, 4H), 7.41 – 7.36 (m, 2H), 4.35 – 4.21 (m, 4H), 1.88 – 1.74 (m, 4H), 1.44 – 1.28 (m, 12H), 0.90 (t, *J* = 7.0 Hz, 6H).

^{13}C NMR (126 MHz, $CDCl_3$) δ 142.3, 141.0, 134.3, 129.1, 128.9, 128.1, 127.4, 127.0, 125.4, 119.5, 118.3, 117.0, 109.9, 44.8, 31.3, 29.6, 26.6, 22.5, 14.0.

Anal. Calcd for $C_{42}H_{42}N_4O_4$: C, 75.65; H, 6.35; N, 8.40. Found: C, 75.45; H, 6.12; N, 8.43.

9. General procedure for denitrohydrogenation of 6,12-dinitro ICZ derivatives 9a and 13a.

Zinc powder (330 mg, 5 mmol) was added to the stirring solution of 6,12-dinitro derivative **9a** (130 mg, 0.25 mmol) or **13a** (170 mg, 0.25 mmol) in THF (10 ml) at room temperature. Concentrated HCl (1.3 ml of 12 M) was added dropwise to this suspension and the resulting mixture was stirred under reflux for 1 h. The reaction mixture was diluted with water (30 ml) and the formed precipitate was collected by filtration and then crystallized from DMF (3 ml) to afford target product **8a** or **8e**, that was filtered, washed with EtOH (5 ml) and dried at 100 °C.

*9.1. 5,11-Dihexyl-5,11-dihydroindolo[3,2-b]carbazole (**8a**).*

Yellowish crystals (70 mg, 66% yield), mp 150–151 °C.

^{1}H NMR (500 MHz, $CDCl_3$) δ 8.21 (d, *J* = 7.7 Hz, 2H), 8.01 (s, 2H), 7.51 – 7.45 (m, 2H), 7.41 (d, *J* = 8.1 Hz, 2H), 7.24 – 7.20 (m, 2H), 4.40 (t, *J* = 7.3 Hz, 4H), 1.99 – 1.90 (m, 4H), 1.50 – 1.43 (m, 4H), 1.38 – 1.27 (m, 8H), 0.87 (d, *J* = 7.1 Hz, 6H).

Anal. Calcd for $C_{30}H_{36}N_2$: C, 84.86; H, 8.55; N, 6.60. Found: C, 84.73; H, 8.85; N, 6.32.

Analytical data of compound **8a** are identical to the reported data [1].

*9.2. 2,8-Dibromo-5,11-dihexyl-5,11-dihydroindolo[3,2-b]carbazole (**8e**).*

Yellowish crystals (86 mg, 59% yield), mp 233–234 °C.

^{1}H NMR (400 MHz, $CDCl_3$) δ 8.31 (d, *J* = 1.8 Hz, 2H), 7.95 (s, 2H), 7.56 (dd, *J* = 8.7, 1.8 Hz, 2H), 7.29 (d, *J* = 8.7 Hz, 2H), 4.36 (t, *J* = 7.3 Hz, 4H), 1.97 – 1.86 (m, 4H), 1.45 – 1.27 (m, 12H), 0.87 (t, *J* = 7.0 Hz, 6H).

Anal. Calcd for $C_{30}H_{34}N_2Br_2$: C, 61.87; H, 5.88; N, 4.81. Found: C, 61.77; H, 5.91; N, 4.88.

Analytical data of compound **8e** are identical to the reported data [4].

10. General procedure for synthesis of alkyl(aryl)thyo ICZ derivatives 15a–h and 16a,b.

6,12-Dinitro derivative **9a** / **13a** / **14a** (0.3 mmol) or 6-nitro derivative **10b** (0.6 mmol) was added to the stirring solution of potassium thiolate, prepared *in situ* from potassium *tert*-butoxide (205 mg, 1.8 mmol) and an appropriate mercaptane (1.8 mmol), in dry DMF (10 ml) at 100 °C. The reaction mixture was stirred and heated at 100 °C for 0.5 h, then it was diluted with 5% solution of NaOH (10 ml). The formed precipitate was filtered and purified by crystallization from DMF (3 ml) to afford desired product **15** or **16**. It was filtered, washed with EtOH (5 ml) and dried under reduced pressure at 50 °C.

*10.1. 5,11-Dihexyl-6,12-bis(methylthio)-5,11-dihydroindolo[3,2-b]carbazole (**15a**)*

Yellow crystals (107 mg, 69% yield), mp 142–143 °C.

^{1}H NMR (500 MHz, C_6D_6) δ 9.61 (d, *J* = 8.0 Hz, 2H), 7.57 – 7.50 (m, 2H), 7.44 – 7.37 (m, 4H), 5.37 – 4.45 (m, 4H), 2.15 (s, 6H), 1.98 – 1.61 (m, 4H), 1.38 – 1.11 (m, 12H), 0.84 (t, *J* = 6.9 Hz, 6H).

^{13}C NMR (126 MHz, $CDCl_3$) δ 143.1, 136.9, 126.3, 126.1, 124.4, 122.9, 118.9, 112.5, 108.7, 45.5, 31.6, 29.4, 26.6, 22.6, 19.9, 14.0.

Anal. Calcd for $C_{32}H_{40}N_2S_2$: C, 74.37; H, 7.80; N, 5.42. Found: C, 74.35; H, 7.84; N, 5.56.

*10.2. 5,11-Dihexyl-6,12-bis(propylthio)-5,11-dihydroindolo[3,2-b]carbazole (**15b**)*

Yellow crystals (112 mg, 65% yield), mp 91–92 °C.

^{1}H NMR (500 MHz, C_6D_6) δ 9.71 (d, *J* = 7.9 Hz, 2H), 7.55 – 7.48 (m, 2H), 7.45 – 7.36 (m, 4H), 5.55 – 5.22 (m, 2H), 4.75 – 4.35 (m, 2H), 3.00 – 2.49 (m, 4H), 2.03 – 1.65 (m, 4H), 1.58 – 1.11 (m, 16H), 0.83 (t, *J* = 6.7 Hz, 6H), 0.66 (br s, 6H).

^{13}C NMR (101 MHz, $CDCl_3$) δ 143.3, 137.8, 126.7, 126.0, 124.6, 123.4, 118.8, 111.5, 108.9, 45.4, 38.9, 31.7, 29.1, 26.6, 23.0, 22.7, 14.1, 13.7.

Anal. Calcd for $C_{36}H_{48}N_2S_2$: C, 75.47; H, 8.45; N, 4.89. Found: C, 75.57; H, 8.24; N, 4.94.

*10.3. 5,11-Dihexyl-6,12-bis(isopropylthio)-5,11-dihydroindolo[3,2-b]carbazole (**15c**)*

Yellow crystals (120 mg, 70% yield), mp 117–118 °C.

^{1}H NMR (500 MHz, C_6D_6) δ 9.82 – 9.62 (m, 2H), 7.54 – 7.45 (m, 2H), 7.44 – 7.35 (m, 4H), 5.69 – 5.32 (m, 2H), 4.59 – 4.29 (m, 2H), 3.59 – 3.12 (m, 2H), 2.00 – 1.66 (m, 4H), 1.41 – 1.06 (m, 18H), 1.04 – 0.90 (m, 6H), 0.87 – 0.76 (m, 6H).

^{13}C NMR (101 MHz, $CDCl_3$) δ 138.3, 133.3, 121.8, 120.8, 119.5, 118.6, 113.6, 106.3, 103.8, 40.3, 34.8, 26.5, 23.6, 21.4, 17.9, 17.5, 17.3, 8.9. The methyl groups of isopropyl moieties are not equivalent.

Anal. Calcd for $C_{36}H_{48}N_2S_2$: C, 75.47; H, 8.45; N, 4.89. Found: C, 75.46; H, 8.35; N, 4.87.

*10.4. 5,11-Dihexyl-6,12-bis(octadecylthio)-5,11-dihydroindolo[3,2-b]carbazole (**15d**)*

Yellowish needles (215 mg, 72% yield), mp 73–74 °C.

^{1}H NMR (500 MHz, C_6D_6) δ 9.75 (d, *J* = 7.9 Hz, 2H), 7.56 – 7.50 (m, 2H), 7.48 – 7.38 (m, 4H), 5.58 – 5.28 (m, 2H), 4.79 – 4.44 (m, 2H), 3.11 – 2.67 (m, 4H), 2.05 – 1.70 (m, 4H), 1.48 – 1.08 (m, 76H), 0.92 (t, *J* = 7.0 Hz, 6H), 0.85 (s, 6H).

^{13}C NMR (101 MHz, $CDCl_3$) δ 143.4, 137.6, 126.7, 125.9, 124.6, 123.5, 118.7, 111.6, 108.8, 45.4, 36.9, 32.3, 31.9, 31.7, 29.8, 29.69, 29.65, 29.6, 29.5, 29.44, 29.42, 29.34, 29.27, 29.2, 29.1, 29.0, 26.6, 22.7, 22.6, 14.05, 13.97 (2 signal ($4C_{Alkyl}$) was not found due to overlapping peaks).

Anal. Calcd for $C_{66}H_{108}N_2S_2$: C, 79.77; H, 10.96; N, 2.82. Found: C, 79.75; H, 10.74; N, 2.83.

*10.5. 5,11-Dihexyl-6,12-bis(phenylthio)-5,11-dihydroindolo[3,2-b]carbazole (**15e**)*

Yellow crystals (177 mg, 92% yield), mp 182–183 °C.

^{1}H NMR (500 MHz, $CDCl_3$) δ 9.06 (d, *J* = 8.1 Hz, 2H), 7.49 – 7.44 (m, 2H), 7.40 (d, *J* = 8.2 Hz, 2H), 7.18 – 7.10 (m, 6H), 7.08 – 6.99 (m, 6H), 5.18 – 4.52 (m, 4H), 2.12 – 1.85 (m, 2H), 1.80 – 1.62 (m, 2H), 1.48 – 1.15 (m, 12H), 0.87 (t, *J* = 6.7 Hz, 6H).

^{13}C NMR (126 MHz, $CDCl_3$) δ 143.0, 138.3, 137.5, 129.3, 128.0, 126.5, 125.4, 125.0, 124.8, 122.6, 119.0, 108.7, 107.3, 45.4, 31.5, 29.8, 26.5, 22.6, 14.0.

Anal. Calcd for $C_{42}H_{44}N_2S_2$: C, 78.71; H, 6.92; N, 4.37. Found: C, 78.73; H, 7.06; N, 4.41.

*10.6. 2,8-Dibromo-5,11-dihexyl-6,12-bis(phenylthio)-5,11-dihydroindolo[3,2-b]carbazole (**15f**)*

Yellow crystals (178 mg, 74% yield), mp 232–233 °C.

^{1}H NMR (400 MHz, $CDCl_3$) δ 9.20 (s, 2H), 7.54 (dd, J = 8.7, 2.0 Hz, 2H), 7.28 (br s, 2H), 7.19 – 7.13 (m, 4H), 7.09 – 7.04 (m, 2H), 7.03 – 6.99 (m, 4H), 5.11 – 4.51 (m, 4H), 2.04 – 1.60 (m, 4H), 1.39 – 1.22 (m, 12H), 0.86 (t, J = 6.6 Hz, 6H).

^{13}C NMR (126 MHz, $CDCl_3$) δ 141.8, 138.9, 137.8, 137.6, 129.4, 129.4, 127.4, 127.3, 125.6, 125.5, 124.1, 111.8, 110.2, 108.2, 45.5, 31.5, 29.7, 26.5, 22.6, 14.0.

Anal. Calcd for $C_{42}H_{42}Br_2N_2S_2$: C, 63.16; H, 5.30; N, 3.51. Found: C, 62.90; H, 5.18; N, 3.57.

*10.7. 2,8-Dibromo-5,11-dihexyl-6,12-bis(propylthio)-5,11-dihydroindolo[3,2-b]carbazole (**15g**)*

Yellow crystals (150 mg, 68% yield), mp 159–160 °C.

^{1}H NMR (500 MHz, C_6D_6) δ 9.93 (br s, 2H), 7.63 (dd, J = 8.8, 2.0 Hz, 2H), 7.03 (d, J = 8.8 Hz, 2H), 5.23 (t, J = 18.0 Hz, 2H), 4.53 – 4.19 (m, 2H), 2.80 – 2.41 (m, 4H), 1.85 – 1.56 (m, 4H), 1.45 – 1.16 (m, 16H), 0.84 (t, J = 6.6 Hz, 6H), 0.75 – 0.59 (m, 6H).

^{13}C NMR (101 MHz, $CDCl_3$) δ 144.0, 137.7, 128.9, 127.1, 126.1, 124.8, 112.0, 111.6, 110.3, 45.5, 39.0, 31.6, 29.1, 26.5, 22.9, 22.7, 14.0, 13.6.

Anal. Calcd for $C_{36}H_{46}Br_2N_2S_2$: C, 59.18; H, 6.35; N, 3.83. Found: C, 58.89; H, 6.38; N, 3.53.

*10.8. 5,11-Dihexyl-2,8-diphenyl-6,12-bis(propylthio)-5,11-dihydroindolo[3,2-b]carbazole (**15h**)*

Yellow crystals (193 mg, 90% yield), mp 169–170 °C.

^{1}H NMR (500 MHz, C_6D_6) δ 10.08 (br s, 2H), 7.96 – 7.92 (m, 4H), 7.87 (dd, J = 8.5, 1.8 Hz, 2H), 7.44 (d, J = 8.5 Hz, 2H), 7.42 – 7.37 (m, 4H), 7.25 – 7.20 (m, 2H), 5.58 – 5.33 (m, 2H), 4.71 – 4.43 (m, 2H), 2.95 – 2.60 (m, 4H), 2.09 – 1.69 (m, 4H), 1.61 – 1.15 (m, 16H), 0.98 – 0.76 (m, 6H), 0.72 – 0.55 (m, 6H).

^{13}C NMR (101 MHz, $CDCl_3$) δ 142.9, 142.6, 138.2, 132.3, 128.9, 127.4, 126.9, 126.4, 125.6, 123.9, 123.2, 111.9, 109.2, 45.6, 39.0, 31.8, 29.3, 26.7, 23.1, 22.8, 14.1, 13.7.

Anal. Calcd for $C_{48}H_{56}N_2S_2$: C, 79.51; H, 7.78; N, 3.86. Found: C, 79.58; H, 7.96; N, 3.84.

*10.9. 5,11-Diethyl-6-(isopropylthio)-5,11-dihydroindolo[3,2-b]carbazole (**16a**)*

Bright yellow crystals (130 mg, 56% yield), mp 189–190 °C.

^{1}H NMR (500 MHz, C_6D_6) δ 9.63 (d, *J* = 7.9 Hz, 1H), 8.18 (d, *J* = 7.6 Hz, 1H), 7.98 (s, 1H), 7.52 – 7.44 (m, 2H), 7.42 – 7.38 (m, 1H), 7.35 – 7.24 (m, 2H), 7.17 (s, 1H), 5.46 (dq, *J* = 13.8, 6.8 Hz, 1H), 4.50 (dq, *J* = 14.1, 6.9 Hz, 1H), 3.88 (q, *J* = 7.2 Hz, 2H), 3.39 (hept, *J* = 6.7 Hz, 1H), 1.25 (t, *J* = 7.0 Hz, 3H), 1.16 (d, *J* = 6.7 Hz, 3H), 1.05 – 0.98 (m, 6H).

^{13}C NMR (101 MHz, $CDCl_3$) δ 142.8, 141.3, 137.4, 135.8, 126.1, 125.9, 125.7, 124.5, 124.37, 123.8, 123.1, 120.0, 118.7, 118.2, 110.1, 109.1, 107.9, 99.5, 40.2, 39.8, 37.6, 23.5, 22.6, 14.0, 13.7. The methyl groups of isopropyl moiety are not equivalent.

Anal. Calcd for $C_{25}H_{26}N_2S$: C, 77.68; H, 6.78; N, 7.25. Found: C, 77.83; H, 6.82; N, 7.47.

*10.10. 5,11-Diethyl-6-(propylthio)-5,11-dihydroindolo[3,2-b]carbazole (**16b**)*

Bright yellow crystals (158 mg, 68% yield), mp 170–171 °C.

^{1}H NMR (500 MHz, C_6D_6) δ 9.64 – 9.61 (m, 1H), 8.21 – 8.16 (m, 1H), 7.98 (s, 1H), 7.53 – 7.46 (m, 2H), 7.41 (ddd, *J* = 8.1, 7.1, 1.0 Hz, 1H), 7.33 – 7.26 (m, 2H), 7.18 (br s, 1H), 5.47 – 5.17 (m, 1H), 4.78 – 4.48 (m, 1H), 3.88 (q, *J* = 7.2 Hz, 2H), 2.82 – 2.68 (m, 2H), 1.55 – 1.37 (m, 2H), 1.27 (t, *J* = 7.0 Hz, 3H), 1.02 (t, *J* = 7.2 Hz, 3H), 0.65 (t, *J* = 7.4 Hz, 3H).

^{13}C NMR (101 MHz, $CDCl_3$) δ 142.7, 141.3, 136.9, 135.8, 126.1, 125.7, 125.6, 124.5, 124.4, 123.5, 123.1, 119.9, 118.7, 118.3, 110.3, 109.0, 107.9, 99.4, 39.8, 39.0, 37.6, 23.2, 14.4, 13.8, 13.7.

Anal. Calcd for $C_{25}H_{26}N_2S$: C, 77.68; H, 6.78; N, 7.25. Found: C, 77.42; H, 6.84; N, 7.54.

11. General procedure for synthesis of ICZ compounds 17a,b.

6,12-Dinitro derivative **9a** (412 mg, 0.8 mmol) was added to the stirring solution of indole (190 mg, 1.6 mmol) or carbazole (270 mg, 1.6 mmol) and potassium *tert*-butoxide (180 mg, 1.6 mmol) in dry DMF (10 ml) at 100 °C. The reaction mixture was stirred and heated at 100 °C for 1 h, then it was diluted with water (10 ml). The formed precipitate was filtered and crystallized from DMF (5 ml) to give desired product **17**, which was collected by filtration, washed with EtOH (5 ml) and dried at 100 °C.

*11.1. 5,11-Dihexyl-6-(1H-indol-1-yl)-12-nitro-5,11-dihydroindolo[3,2-b]carbazole (**17a**)*

Orange flakes (250 mg, 53% yield), mp 147–148 °C.

^{1}H NMR (400 MHz, $CDCl_3$) δ 7.99 (d, *J* = 8.1 Hz, 1H), 7.85 (d, *J* = 7.9 Hz, 1H), 7.58 – 7.49 (m, 1H), 7.45 – 7.27 (m, 5H), 7.24 – 7.20 (m, 1H), 7.13 – 7.07 (m, 1H), 6.98 (d, *J* = 3.1 Hz, 1H), 6.91 (d, *J* = 8.2 Hz, 1H), 6.88 – 6.82 (m, 1H), 6.15 (d, *J* = 8.0 Hz, 1H), 4.43 – 4.10 (m, 2H), 3.57 – 3.27 (m, 2H), 1.88 – 1.75 (m, 2H), 1.47 – 1.29 (m, 7H), 1.26 – 1.14 (m, 1H), 1.12 – 1.02 (m, 2H), 0.94 – 0.83 (m, 5H), 0.82 – 0.70 (m, 4H), 0.58 – 0.46 (m, 1H).

^{13}C NMR (126 MHz, $CDCl_3$) δ 142.2, 142.1, 136.8, 131.5, 128.0, 127.89, 127.85, 127.2, 126.9, 125.0, 124.6, 122.6, 121.7, 120.84, 120.75, 120.4, 119.7, 119.6, 119.5, 118.0, 116.5, 115.3, 110.4, 109.1, 108.5, 104.0, 44.1, 43.9, 30.9, 30.7, 29.2, 29.0, 26.2, 25.8, 22.1, 22.0, 13.52, 13.47.

Anal. Calcd for $C_{38}H_{40}N_4O_2$: C, 78.05; H, 6.90; N, 9.58. Found: C, 77.91; H, 7.20; N, 9.60.

*11.2. 6-(9H-Carbazol-9-yl)-5,11-dihexyl-12-nitro-5,11-dihydroindolo[3,2-b]carbazole (**17b**)*

Orange plates (430 mg, 85% yield), mp 211–212 °C.

^{1}H NMR (500 MHz, $CDCl_3$) δ 8.31 (d, J = 7.6 Hz, 2H), 8.02 (d, J = 8.2 Hz, 1H), 7.57 – 7.48 (m, 1H), 7.41 – 7.26 (m, 8H), 6.96 (d, J = 8.0 Hz, 2H), 6.72 – 6.59 (m, 1H), 6.05 (d, J = 8.0 Hz, 1H), 4.37 – 4.21 (m, 2H), 3.43 – 3.29 (m, 2H), 1.93 – 1.79 (m, 2H), 1.49 – 1.29 (m, 6H), 1.17 – 1.04 (m, 2H), 0.96 – 0.82 (m, 5H), 0.68 (t, J = 7.3 Hz, 3H), 0.62 – 0.52 (m, 2H), 0.37 – 0.26 (m, 2H).

^{13}C NMR (126 MHz, $CDCl_3$) δ 142.7, 142.6, 140.8, 132.4, 128.6, 127.6, 127.2, 126.6, 125.8, 125.1, 123.3, 122.2, 121.2, 120.8, 120.6, 120.1, 119.93, 119.89, 118.6, 116.0, 114.6, 110.4, 109.5, 108.8, 44.6, 44.4, 31.4, 31.0, 29.5, 29.4, 26.7, 26.1, 22.5, 22.4, 14.0, 13.9.

Anal. Calcd for $C_{42}H_{42}N_4O_2$: C, 79.46; H, 6.67; N, 8.83. Found: C, 79.25; H, 6.70; N, 8.83.

12. General procedure for synthesis of ICZ compounds 18a,b.

Compound **17b** (385 mg, 0.6 mmol) was added in one portion to the stirring solution of potassium thiolate, prepared from in situ potassium *tert*-butoxide (205 mg, 1.8 mmol) and an appropriate mercaptane (1.8 mmol), in dry DMF (10 ml) at 100 °C. The reaction mixture was stirred and heated at 100 °C for 0.5 h, then it was diluted with 5% solution of NaOH (10 ml). The formed precipitate was filtered and crystallized from DMF (5 ml) to afford desired product **18**. The latter was filtered, washed with EtOH (5 ml) and dried at 100 °C.

*12.1. 6-(9H-Carbazol-9-yl)-5,11-dihexyl-12-(isopropylthio)-5,11-dihydroindolo[3,2-b]carbazole (**18a**)*

Yellow crystals (280 mg, 70% yield), mp 182–183 °C.

^{1}H NMR (500 MHz, C_6D_6) δ 9.99 – 9.59 (m, 1H), 8.21 (d, J = 7.7 Hz, 2H), 7.47 – 7.38 (m, 2H), 7.27 – 7.19 (m, 3H), 7.14 – 7.04 (m, 4H), 7.00 (d, J = 8.0 Hz, 1H), 6.95 (d, J = 8.1 Hz, 1H), 6.49 – 6.40 (m, 2H), 5.65 – 5.56 (m, 1H), 4.66 – 4.15 (m, 1H), 3.55 – 3.46 (m, 1H), 3.39 – 3.23 (m, 2H), 2.07 – 1.94 (m, 1H), 1.86 – 1.74 (m, 1H), 1.45 – 1.30 (m, 2H), 1.29 – 1.19 (m, 7H), 1.17 – 1.02 (m, 5H), 0.95 – 0.82 (m, 5H), 0.73 (t, J = 7.3 Hz, 3H), 0.60 – 0.49 (m, 2H), 0.30 – 0.15 (m, 2H).

^{13}C NMR (101 MHz, $CDCl_3$) δ 143.1, 142.6, 141.2, 138.1, 132.9, 128.0, 126.5, 126.4, 126.2, 126.1, 124.6, 123.8, 123.3, 123.2, 122.2 120.7, 120.6, 120.3, 119.2, 118.9, 112.9, 111.5, 110.8, 110.6, 108.7,

45.4, 44.4, 40.3, 31.7, 31.2, 29.4, 28.9, 26.6, 26.2, 23.5, 22.7, 22.6, 22.5, 14.1, 14.0. The CH_3 groups of isopropyl moiety are not equivalent.

Anal. Calcd for $C_{45}H_{49}N_3S$: C, 81.40; H, 7.44; N, 6.33. Found: C, 81.44; H, 7.65; N, 6.35.

*12.2. 6-(9H-Carbazol-9-yl)-5,11-dihexyl-12-(propylthio)-5,11-dihydroindolo[3,2-b]carbazole (**18b**)*

Yellow crystals (315 mg, 79% yield), mp 177–178 °C.

^{1}H NMR (500 MHz, C_6D_6) δ 9.80 – 9.75 (m, 1H), 8.20 (d, *J* = 7.8 Hz, 2H), 7.49 – 7.39 (m, 2H), 7.26 – 7.19 (m, 3H), 7.13 – 7.06 (m, 4H), 7.01 – 6.90 (m, 2H), 6.51 – 6.42 (m, 2H), 5.61 – 5.34 (m, 1H), 4.69 – 4.50 (m, 1H), 3.42 – 3.21 (m, 2H), 3.03 – 2.80 (m, 2H), 2.09 – 1.94 (m, 1H), 1.85 – 1.73 (m, 1H), 1.70 – 1.54 (m, 2H), 1.47 – 1.32 (m, 2H), 1.30 – 1.17 (m, 4H), 1.14 – 1.02 (m, 2H), 0.92 – 0.85 (m, 5H), 0.79 (t, *J* = 7.4 Hz, 3H), 0.73 (t, *J* = 7.3 Hz, 3H), 0.58 – 0.49 (m, 2H), 0.30 – 0.17 (m, 2H).

^{13}C NMR (101 MHz, $CDCl_3$) δ 143.0, 142.6, 141.2, 137.5, 132.9, 127.7, 126.4, 126.3, 126.2, 124.5, 123.6, 123.2, 122.1, 120.7, 120.6, 120.2, 119.2, 119.0, 112.9, 111.6, 110.7, 108.7, 45.4, 44.4, 39.2, 31.8, 31.1, 29.42, 29.38, 26.7, 26.2, 23.2, 22.7, 22.5, 14.1, 14.0, 13.9.

Anal. Calcd for $C_{45}H_{49}N_3S$: C, 81.40; H, 7.44; N, 6.33. Found: C, 81.14; H, 7.74; N, 6.42.

13. Crystallographic data and results of refinement for the structure 2a in the XRD experiment.

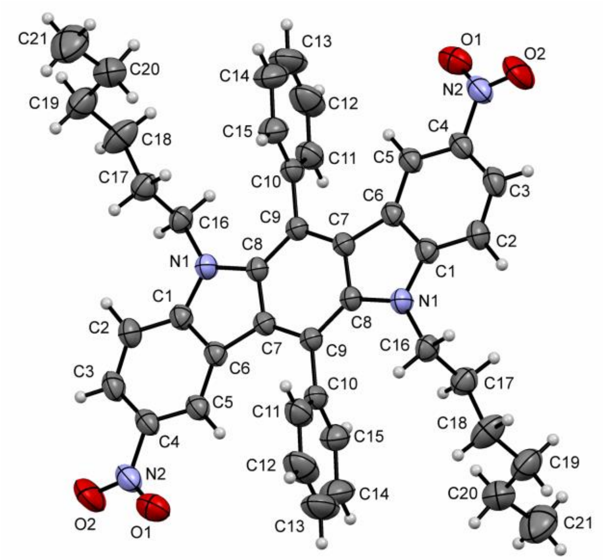

Compound **2a** in according XRD data. Thermal ellipsoids are shown at 50% probability level.

Deposition number CCDC 1537015 contains the supplementary crystallographic data for this structure. These data can be obtained free of charge from the Cambridge Crystallographic Data Centre via www.ccdc.cam.ac.uk/data_request/cif.

Compound **2a**	
Empirical formula	$C_{42}H_{42}N_4O_4$
Formula weight	666.80
Temperature/K	295(2)
Crystal system	monoclinic
Space group	$P2_1/c$
a/Å	14.8902(6)
b/Å	7.4148(4)
c/Å	15.9282(6)
α/°	90.00
β/°	94.855(3)
γ/°	90.00
Volume/Å^3	1752.31(13)
Z	2
ρ_{calc} mg/mm^3	1.264
m/mm^{-1}	0.082
F(000)	708
Crystal size/mm^3	0.57 × 0.49 × 0.11
2Θ range for data collection	2.7430 < Θ < 29.7160°
Index ranges	-21 < h < 18,<br>-10 < k < 9,<br>-22 < l < 19
Reflections collected	9644
Independent reflections	4798 [R(int) = 0.0196]
Data/restraints/parameters	4798 / 0 / 239
Goodness-of-fit on F^2	1.007
Final R indexes [I>2σ (I)]	R_1 = 0.0508,<br>wR_2 = 0.1475
Final R indexes [all data]	R_1 = 0.0863,<br>wR_2 = 0.1775
Largest diff. peak/hole / e Å^{-3}	0.230/-0.172

14. Crystallographic data and results of refinement for the structure 9b in the XRD experiment.

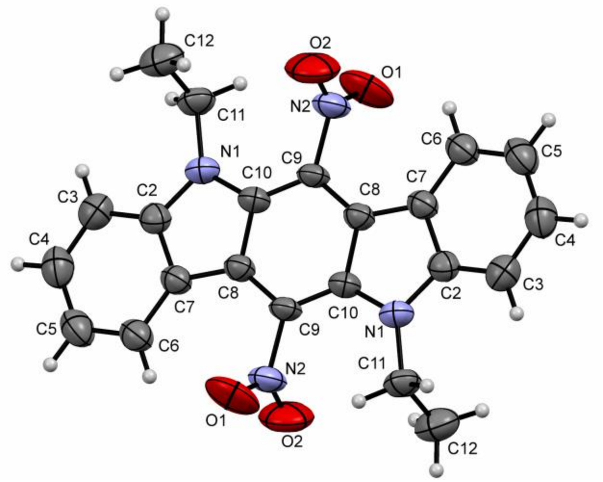

Compound **9b** in according XRD data. Thermal ellipsoids are shown at 50% probability level.

Deposition number CCDC 1537016 contains the supplementary crystallographic data for this structure. These data can be obtained free of charge from the Cambridge Crystallographic Data Centre via www.ccdc.cam.ac.uk/data_request/cif.

Compound **9b**	
Empirical formula	$C_{22}H_{18}N_4O_4$
Formula weight	402.40
Temperature/K	293(2)
Crystal system	monoclinic
Space group	$P2_1/n$
a/Å	10.0099(16)
b/Å	7.2367(8)
c/Å	13.2682(12)
α/°	90.00
β/°	101.894(12)
γ/°	90.00
Volume/Å^3	940.5(2)
Z	2
ρ_{calc} mg/mm^3	1.421
m/mm^{-1}	0.101
F(000)	420
Crystal size/mm^3	0.25 × 0.2 × 0.15
2Θ range for data collection	2.4050 < Θ < 28.1830°
Index ranges	-13 < h < 6,<br>-8 < k < 9,<br>-17 < l < 15
Reflections collected	4350
Independent reflections	2334 [R(int) = 0.0490]
Data/restraints/parameters	2334 / 0 / 138
Goodness-of-fit on F^2	1.114
Final R indexes [I>2σ (I)]	R_1 = 0.0679,<br>wR_2 = 0.1758
Final R indexes [all data]	R_1 = 0.1120,<br>wR_2 = 0.2164
Largest diff. peak/hole / e Å^{-3}	0.323/-0.220

15. Crystallographic data and results of refinement for the structure 10b in the XRD experiment.

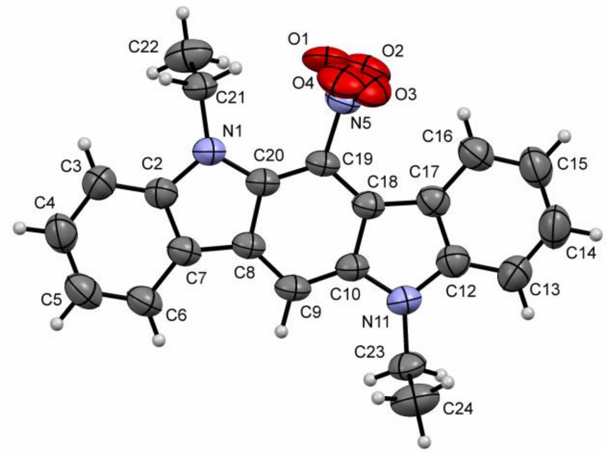

Compound **10b** in according XRD data. Thermal ellipsoids are shown at 50% probability level.

Deposition number CCDC 1537017 contains the supplementary crystallographic data for this structure. These data can be obtained free of charge from the Cambridge Crystallographic Data Centre via www.ccdc.cam.ac.uk/data_request/cif.

Compound **10b**	
Empirical formula	$C_{22}H_{19}N_3O_2$
Formula weight	357.40
Temperature/K	295(2)
Crystal system	monoclinic
Space group	$P2_1$
a/Å	8.2838(8)
b/Å	12.4443(10)
c/Å	8.7429(5)
α/°	90.00
β/°	97.939(6)
γ/°	90.00
Volume/Å^3	892.63(12)
Z	2
ρ_{calc} mg/mm^3	1.330
m/mm^{-1}	0.087
F(000)	376
Crystal size/mm^3	0.25 × 0.2 × 0.15
2Θ range for data collection	2.5070 < Θ < 29.4080°
Index ranges	-11 < h < 10,<br>-13 < k < 17,<br>-12 < l < 11
Reflections collected	4331
Independent reflections	3164 [R(int) = 0.0209]
Data/restraints/parameters	3164 / 1 / 268
Goodness-of-fit on F^2	1.005
Final R indexes [I>2σ (I)]	R_1 = 0.0483,<br>wR_2 = 0.1193
Final R indexes [all data]	R_1 = 0.0701,<br>wR_2 = 0.1385
Largest diff. peak/hole / e Å^{-3}	0.235/-0.185

16. Crystallographic data and results of refinement for the structure 12b in the XRD experiment.

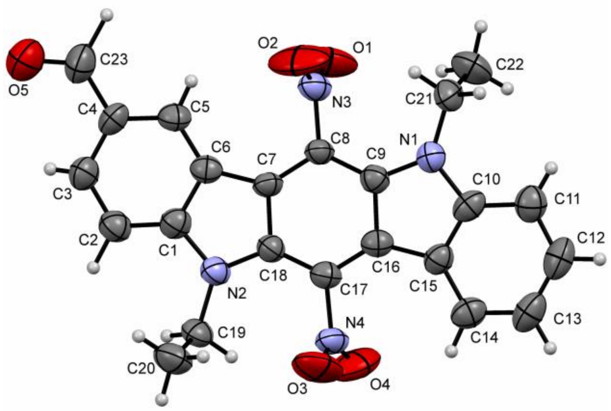

Compound **12b** in according XRD data. Thermal ellipsoids are shown at 50% probability level.

Deposition number CCDC 1537018 contains the supplementary crystallographic data for this structure. These data can be obtained free of charge from the Cambridge Crystallographic Data Centre via www.ccdc.cam.ac.uk/data_request/cif.

Compound **12b**	
Empirical formula	$C_{23}H_{18}N_4O_5$
Formula weight	430.41
Temperature/K	295(2)
Crystal system	monoclinic
Space group	$P2_1/c$
a/Å	16.122(8)
b/Å	6.715(4)
c/Å	18.426(13)
α/°	90.00
β/°	104.13(5)
γ/°	90.00
Volume/Å^3	21934(2)
Z	4
ρ_{calc} mg/mm^3	1.478
m/mm^{-1}	0.887
F(000)	896
Crystal size/mm^3	0.3 × 0.2 × 0.1
2Θ range for data collection	2.8337 < Θ < 65.5884°
Index ranges	-18 < h < 16,<br>-6 < k < 7,<br>-21 < l < 21
Reflections collected	15828
Independent reflections	3291 [R(int) = 0.0969]
Data/restraints/parameters	3291 / 0 / 311
Goodness-of-fit on F^2	1.000
Final R indexes [I>2σ (I)]	R_1 = 0.0582,<br>wR_2 = 0.1100
Final R indexes [all data]	R_1 = 0.1373,<br>wR_2 = 0.1181
Largest diff. peak/hole / e Å^{-3}	0.270/-0.211

17. Crystallographic data and results of refinement for the structure 13b in the XRD experiment.

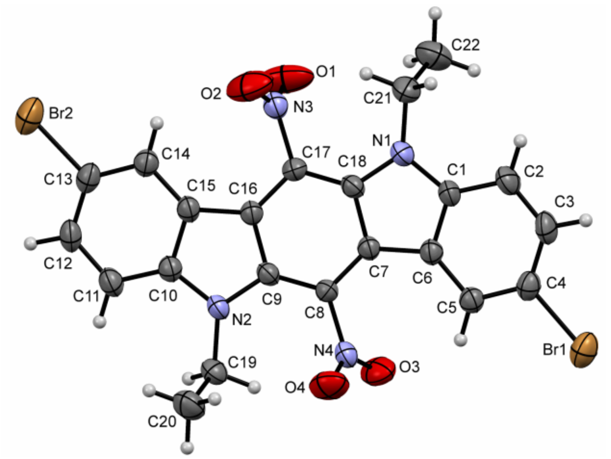

Compound **13b** in according XRD data. Thermal ellipsoids are shown at 50% probability level.

Deposition number CCDC 1537019 contains the supplementary crystallographic data for this structure. These data can be obtained free of charge from the Cambridge Crystallographic Data Centre via www.ccdc.cam.ac.uk/data_request/cif.

Compound **13b**	
Empirical formula	$C_{22}H_{16}N_4O_4Br_2$
Formula weight	560.21
Temperature/K	295(2)
Crystal system	monoclinic
Space group	$P2_1/c$
a/Å	10.920(9)
b/Å	11.628(5)
c/Å	16.567(19)
α/°	90.00
β/°	106.81(9)
γ/°	90.00
Volume/Å^3	2014(3)
Z	4
ρ_{calc} mg/mm^3	1.848
m/mm^{-1}	5.462
F(000)	1112
Crystal size/mm^3	0.2 × 0.15 × 0.1
2Θ range for data collection	4.23 < Θ < 66.85°
Index ranges	-12 < h < 12,<br>-13 < k < 13,<br>-19 < l < 19
Reflections collected	21664
Independent reflections	3535 [R(int) = 0.0548]
Data/restraints/parameters	3535 / 0 / 289
Goodness-of-fit on F^2	1.005
Final R indexes [I>2σ (I)]	R_1 = 0.0462,<br>wR_2 = 0.1175
Final R indexes [all data]	R_1 = 0.0580,<br>wR_2 = 0.1207
Largest diff. peak/hole / e Å^{-3}	0.731/-0.433

18. References.

1. Yudina, L. N.; Bergman, J. *Tetrahedron* **2003**, *59*, 1265–1275.

2. Van Snick, S.; Dehaen, W. *Org. Biomol. Chem.* **2012**, *10*, 79–82.

3. Irgashev, R. A.; Teslenko, A. Y.; Zhilina, E. F.; Schepochkin, A. V.; El'tsov, O. S.; Rusinov, G. L.; Charushin, V. N. *Tetrahedron* **2014**, *70*, 4685–4696.

4. Shi, H.; Yuan, J.; Dong, X.; Cheng, F. *Spectrochim. Acta - Part A Mol. Biomol. Spectrosc.* **2014**, *133*, 501–508.

5. Petrikyte, I.; Zimmermann, I.; Rakstys, K.; Daskeviciene, M.; Malinauskas, T.; Jankauskas, V.; Getautis, V.; Nazeeruddin, M. K. *Nanoscale* **2016**, *8*, 8530–8535.

19. Copies of ^{1}H and ^{13}C NMR spectra of new compounds.

^{1}H NMR (solvent: $CDCl_3$)

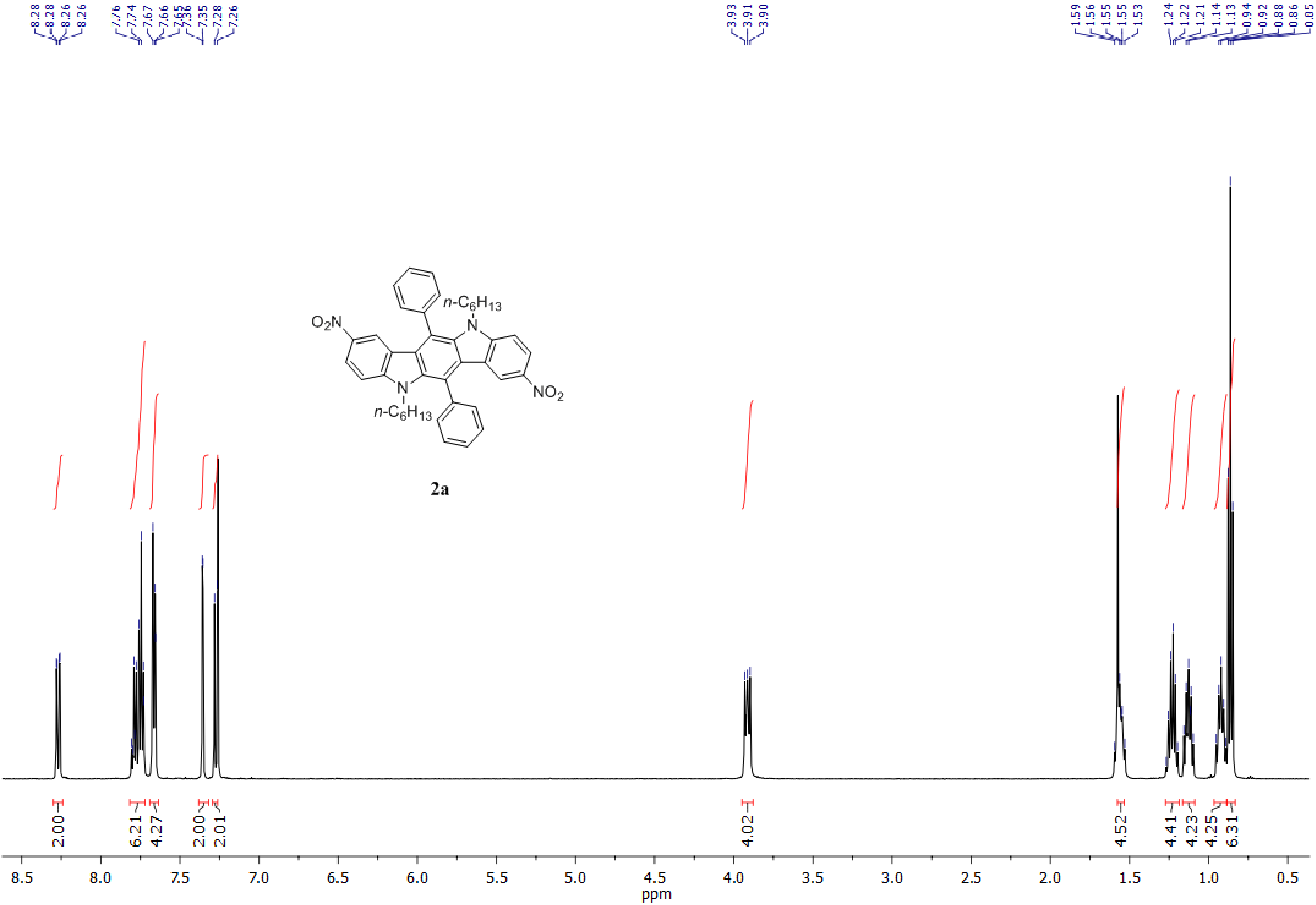

^{13}C NMR (solvent: $CDCl_3$)

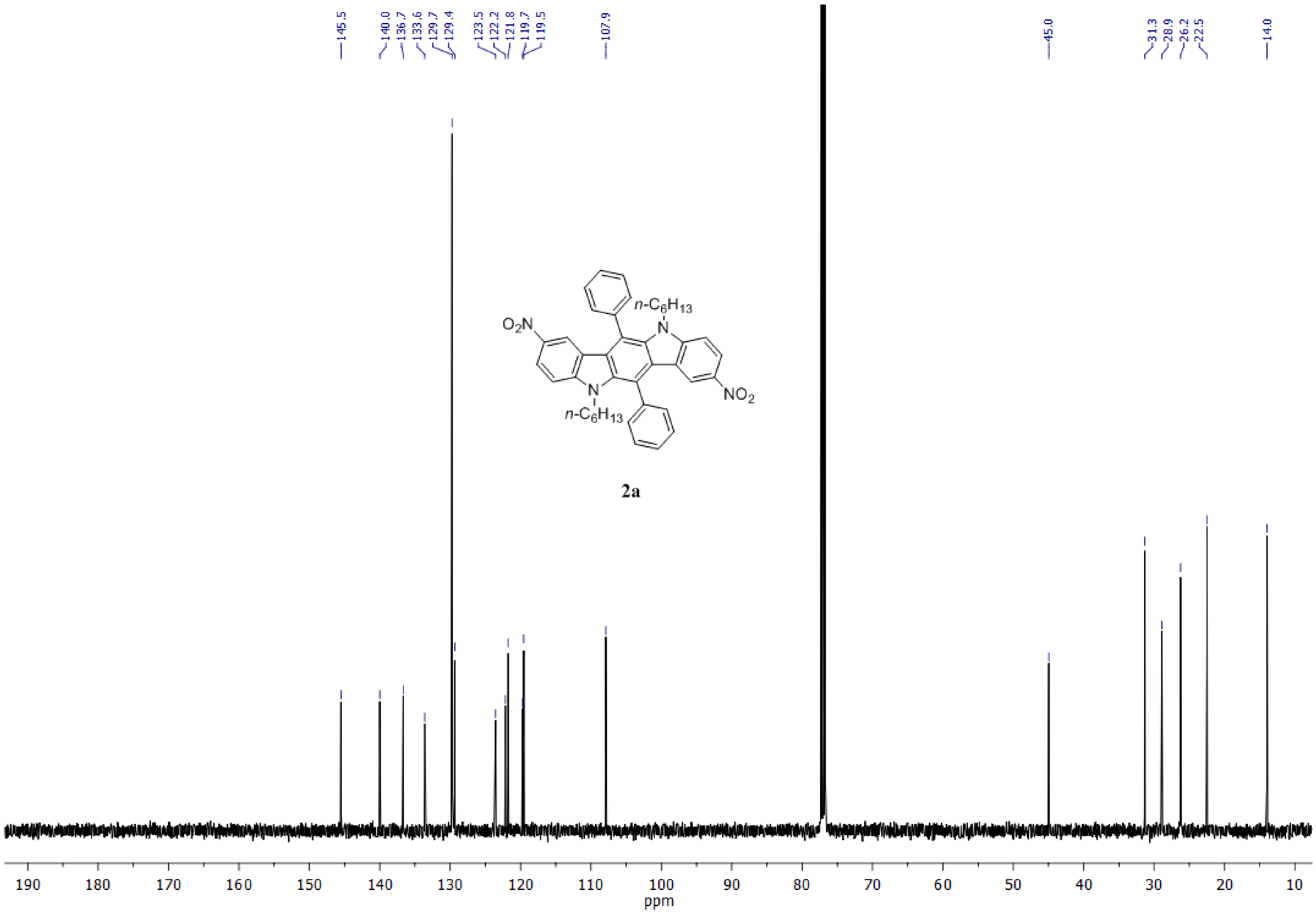

^{1}H NMR (solvent: $CDCl_3$)

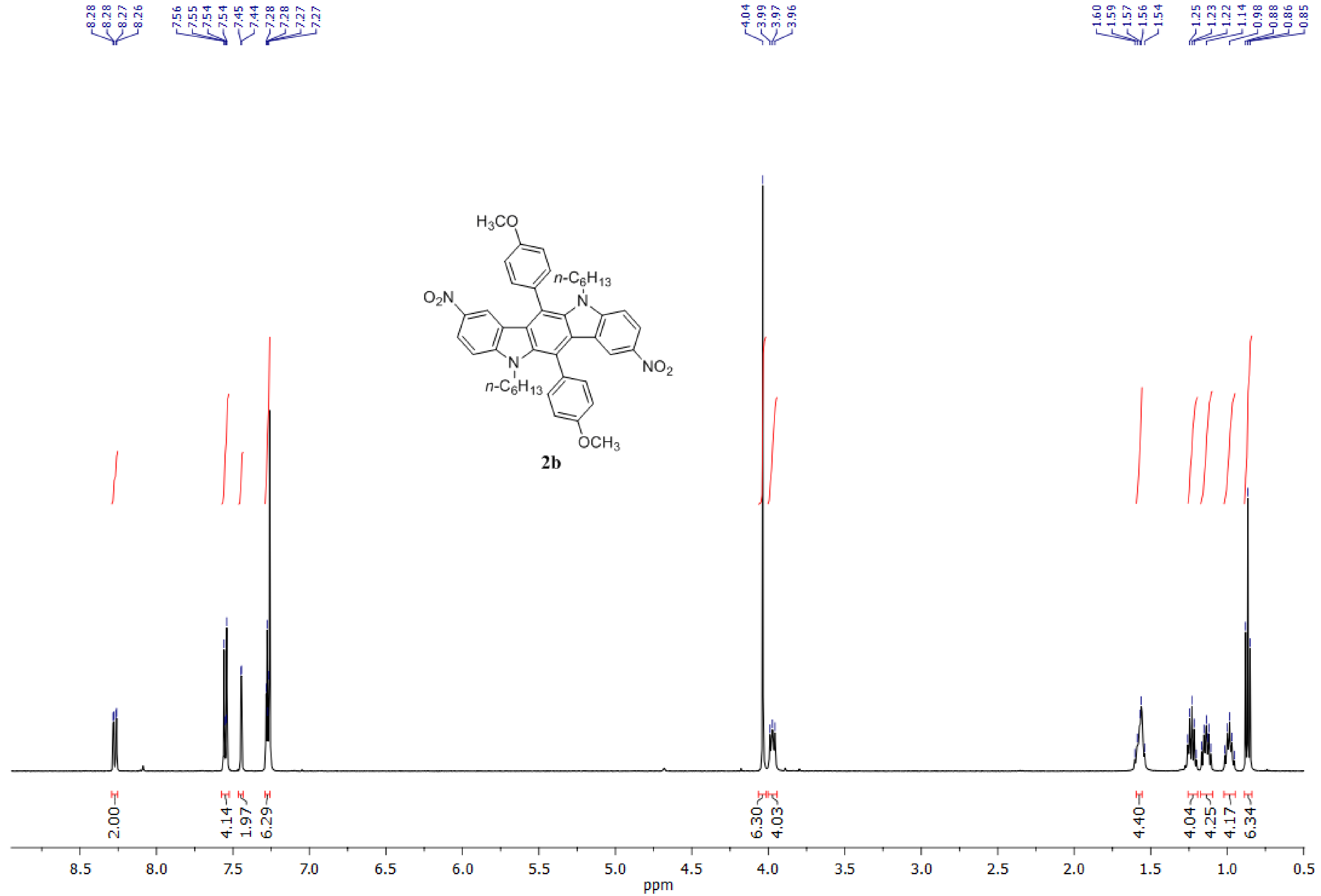

^{13}C NMR (solvent: $CDCl_3$)

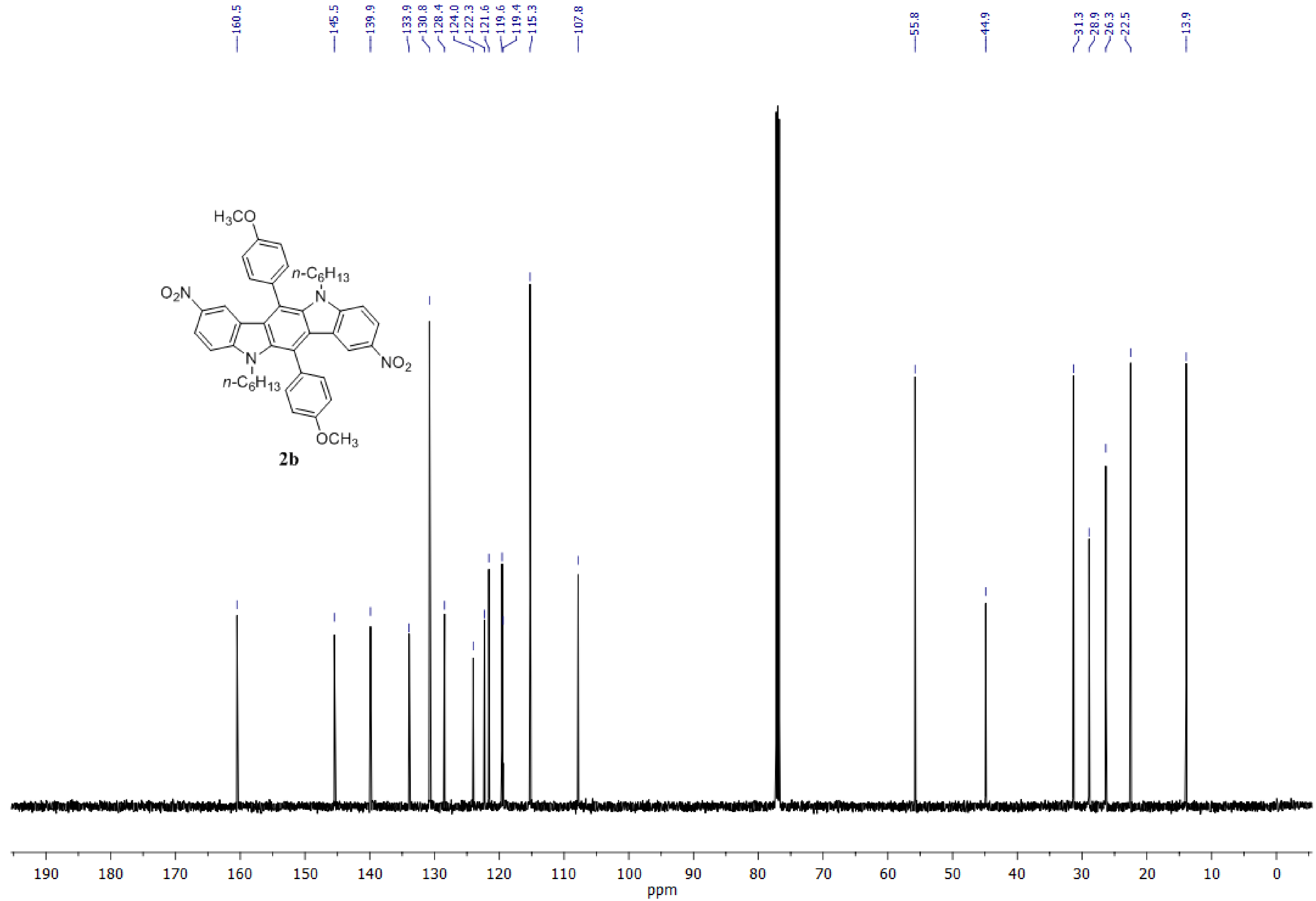

^{1}**H NMR** (solvent: $CDCl_3$)

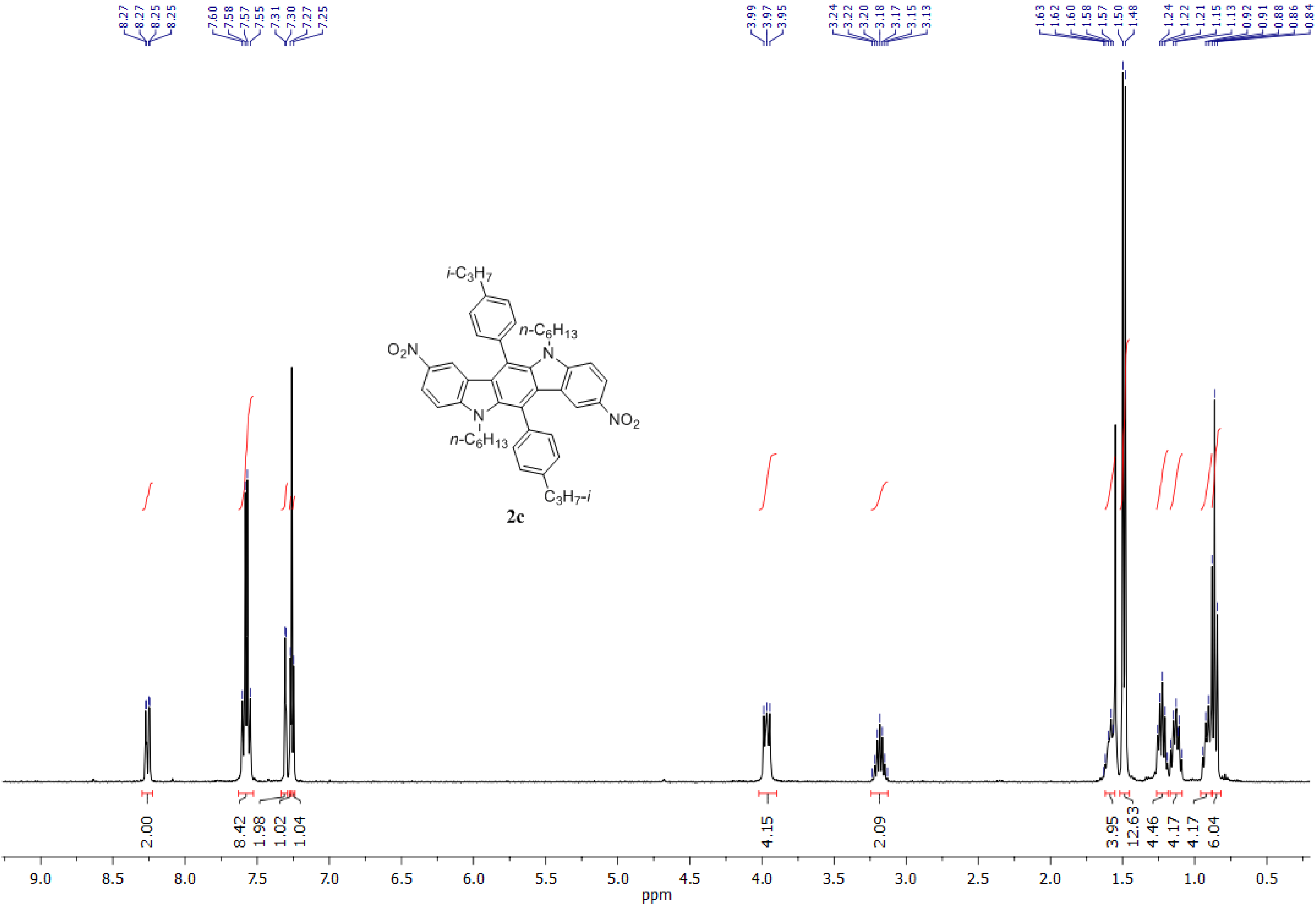

^{13}C NMR (solvent: $CDCl_3$)

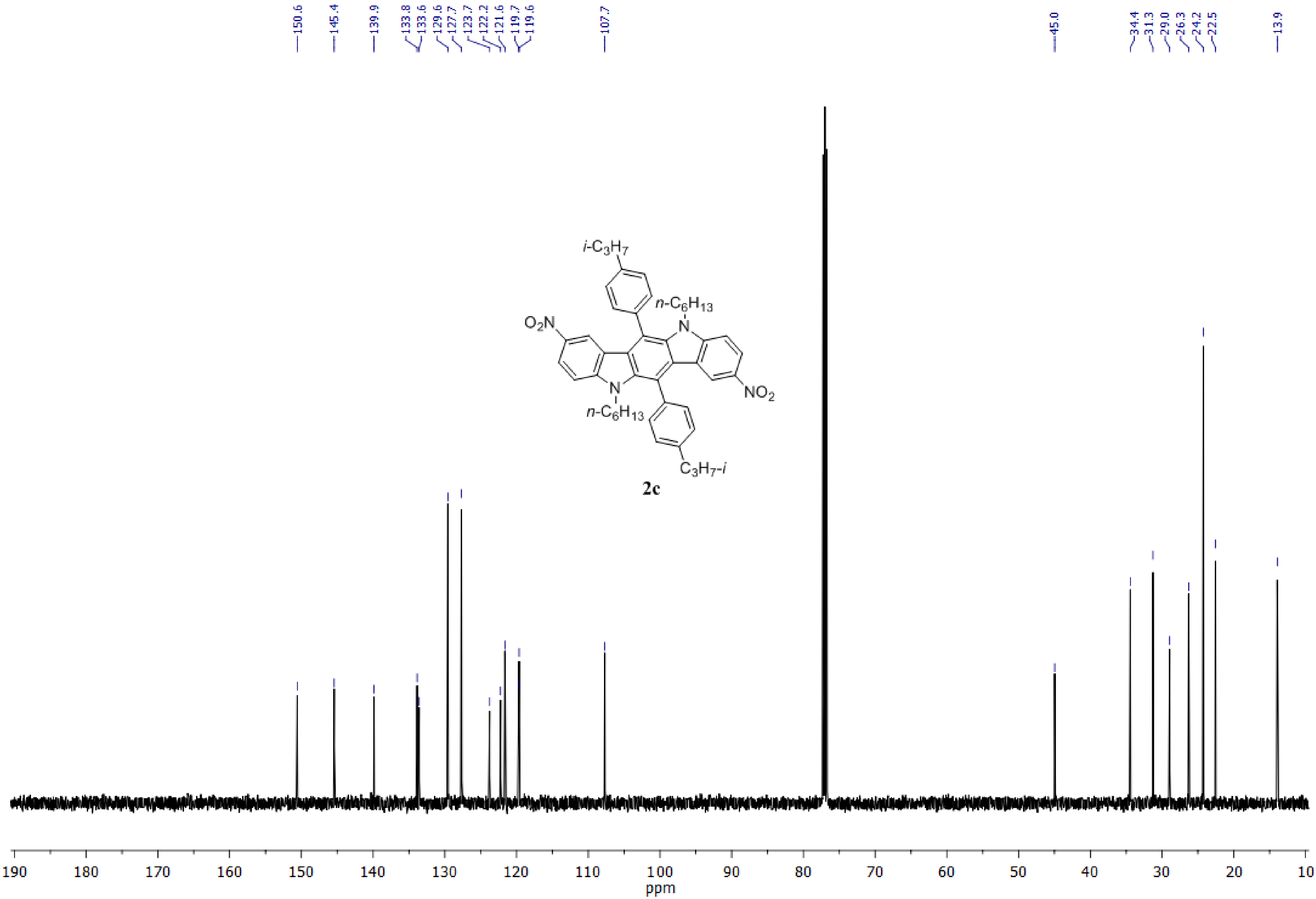

[1]**H NMR** (solvent: $CDCl_3$)

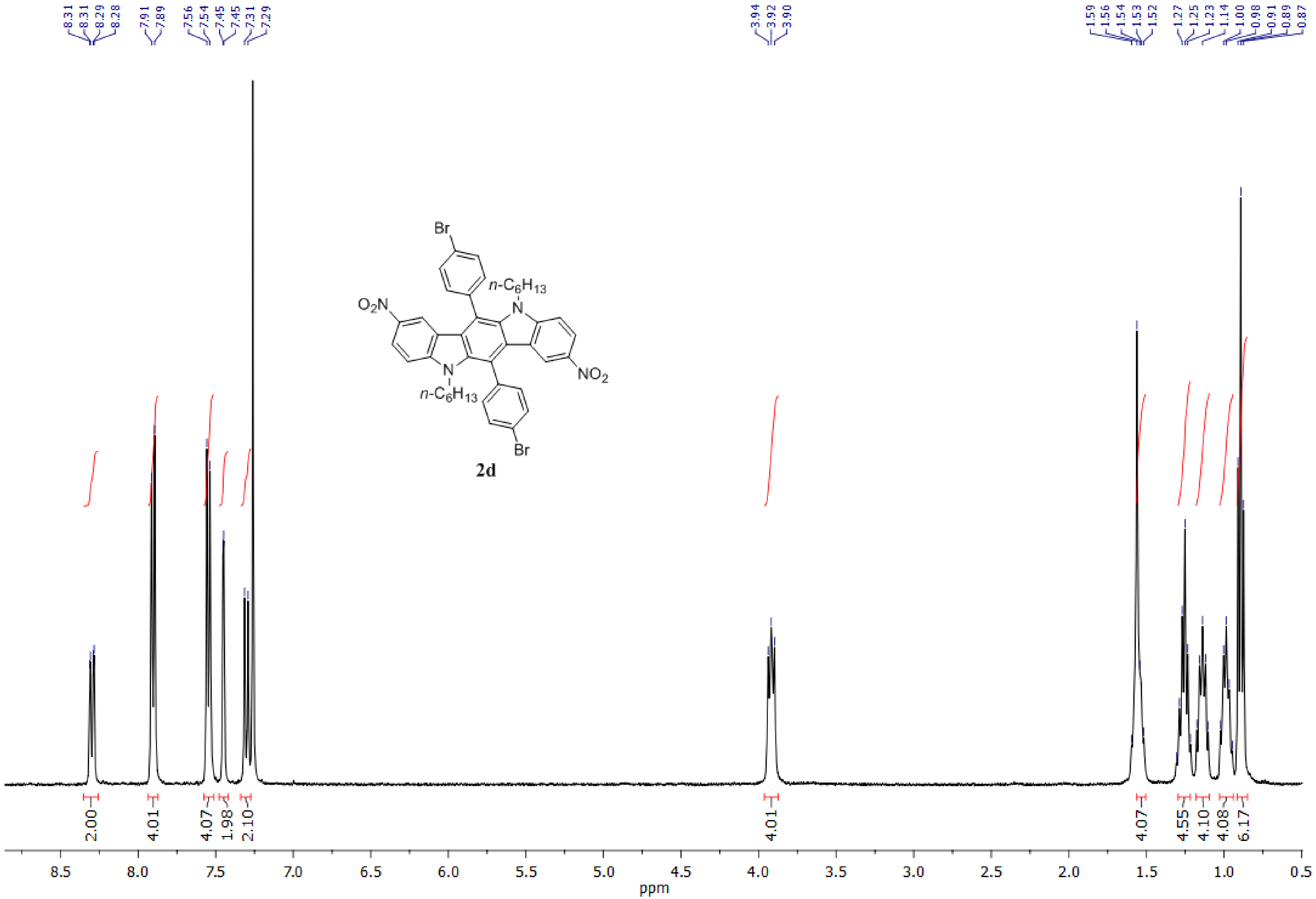

^{13}C NMR (solvent: $CDCl_3$)

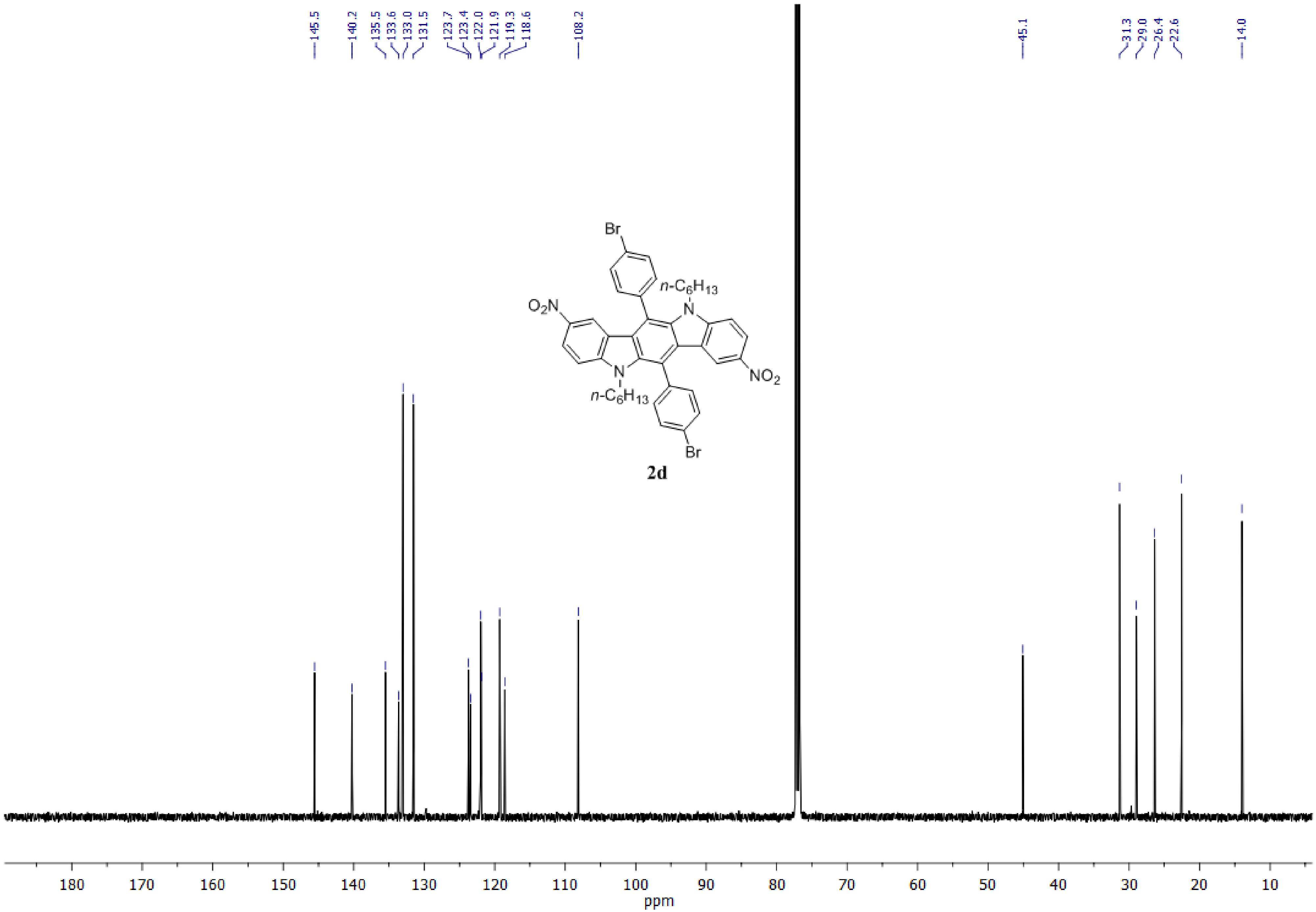

2d

^{1}H NMR (solvent: $CDCl_3$)

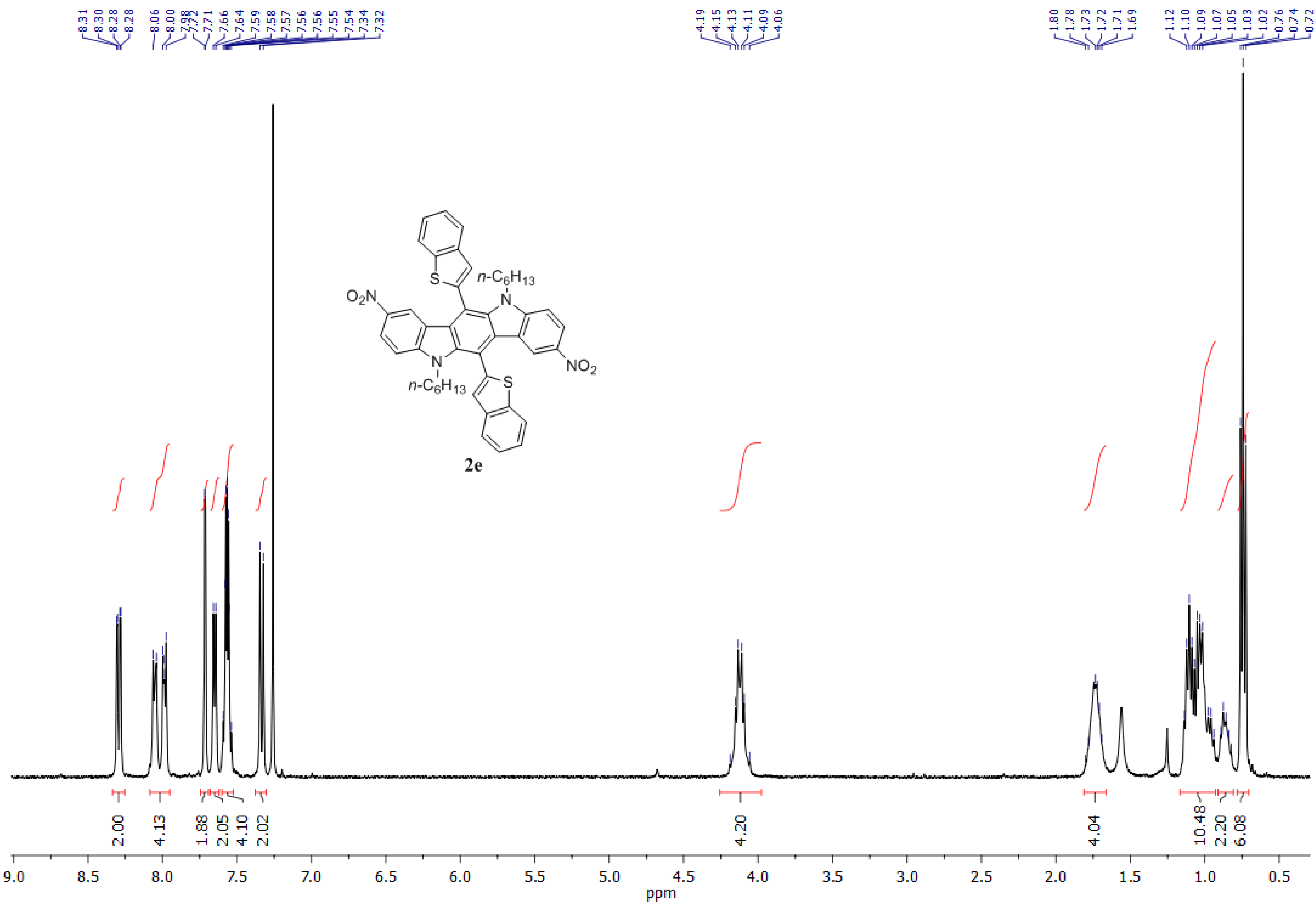

^{13}C NMR (solvent: $CDCl_3$)

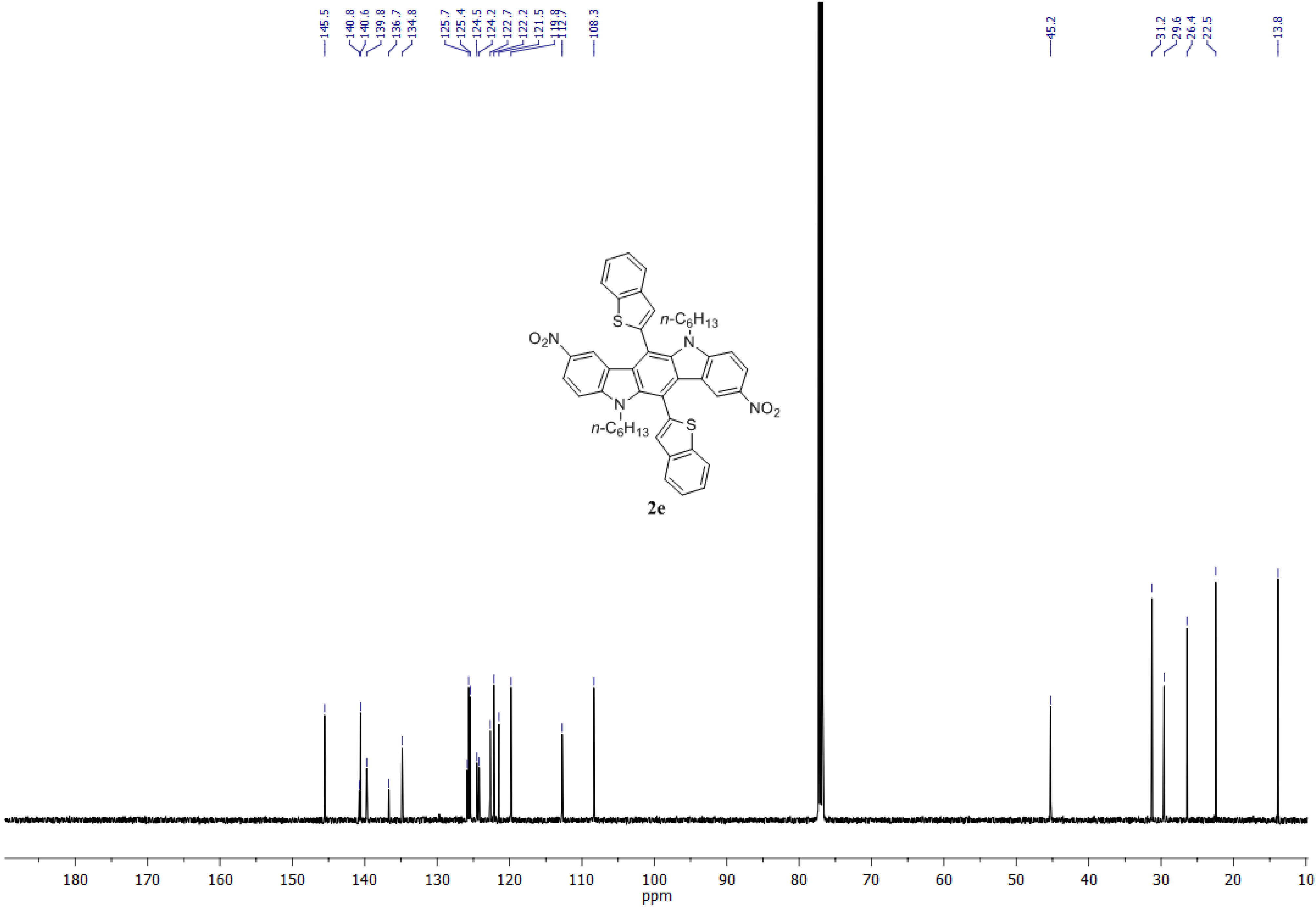

^{1}**H NMR** (solvent: $CDCl_3$)

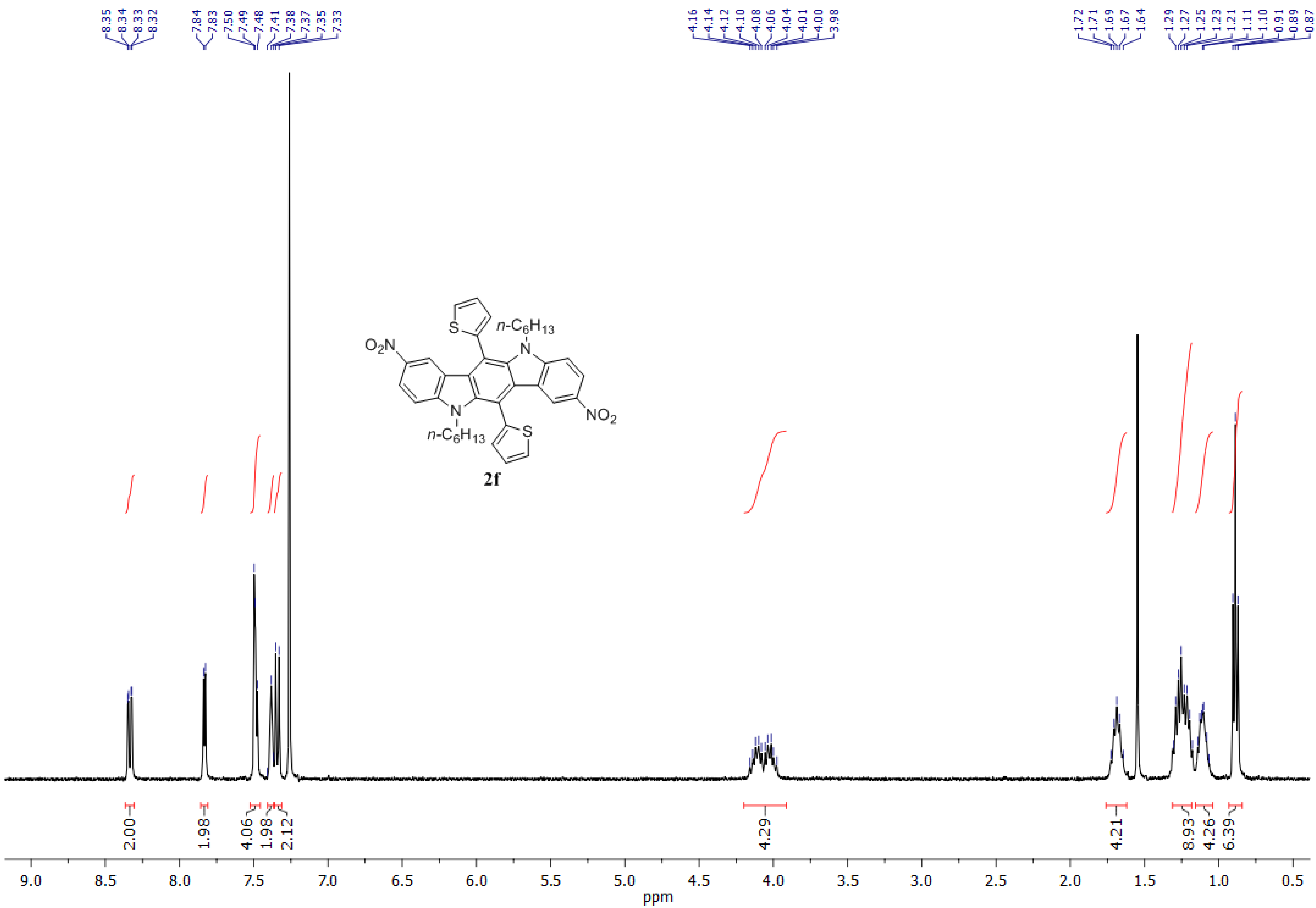

^{13}C NMR (solvent: $CDCl_3$)

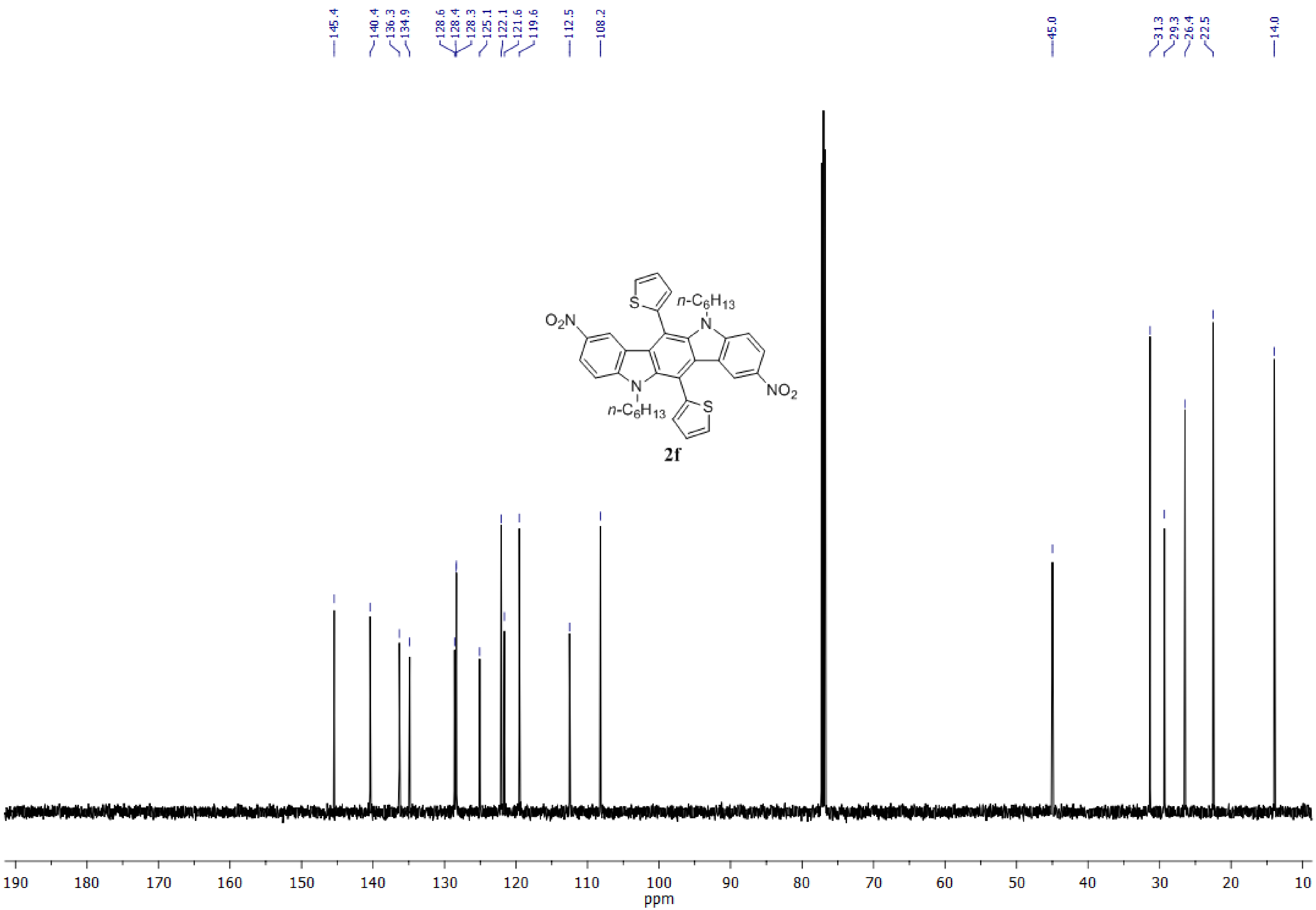

2f

^{1}H NMR (solvent: $CDCl_3$)

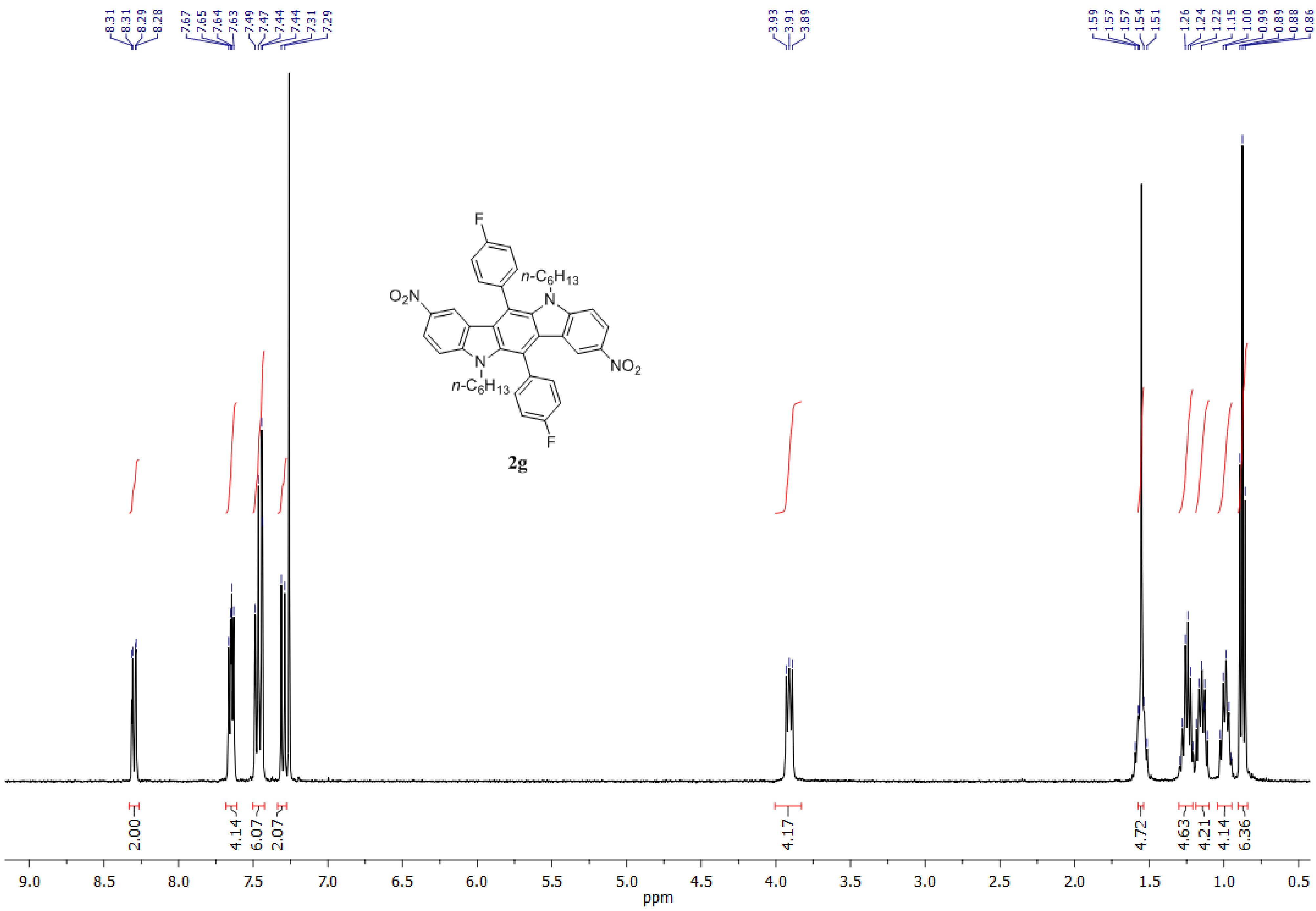

^{13}**C NMR** (solvent: $CDCl_3$)

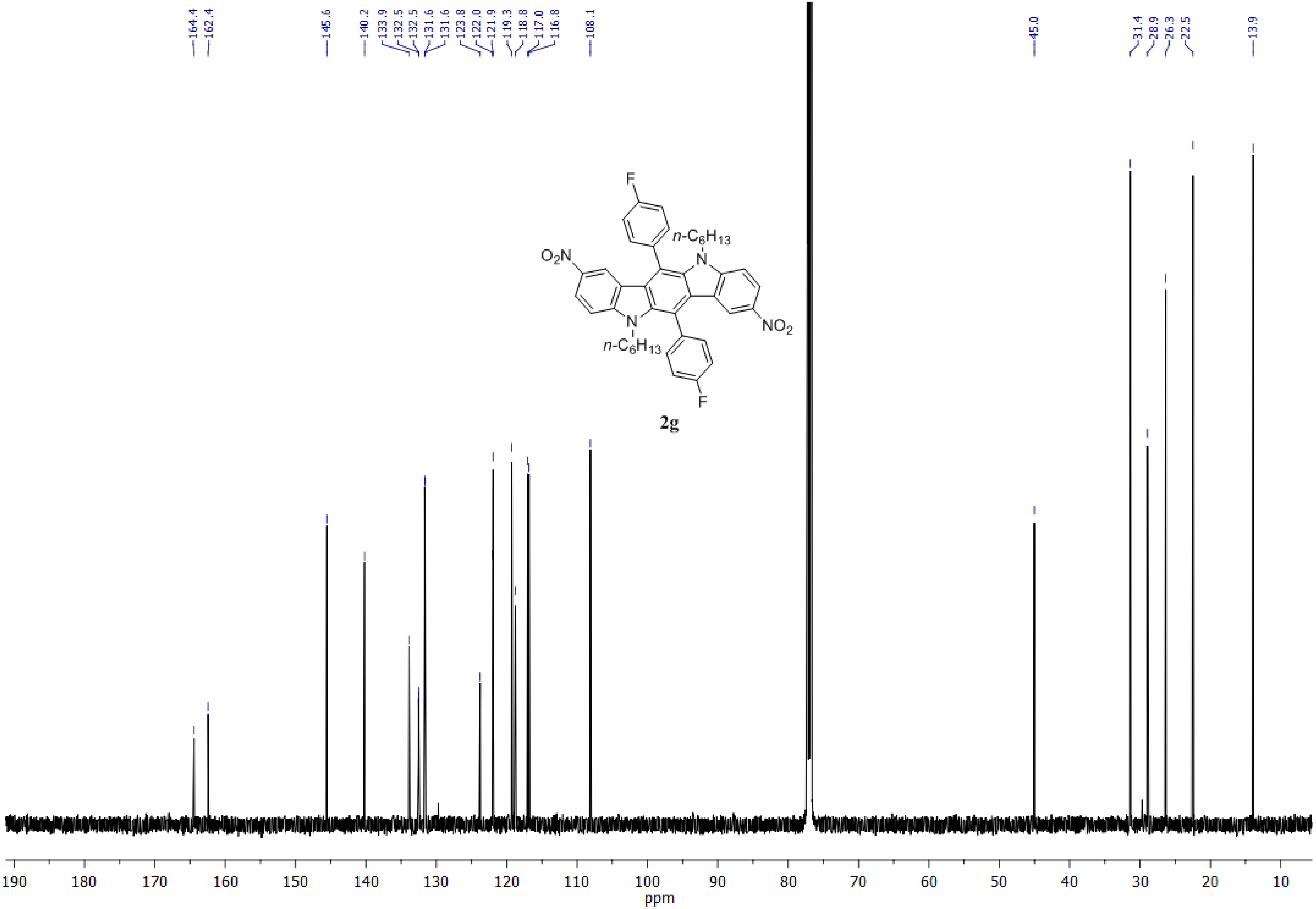

^{1}**H NMR** (solvent: $CDCl_3$)

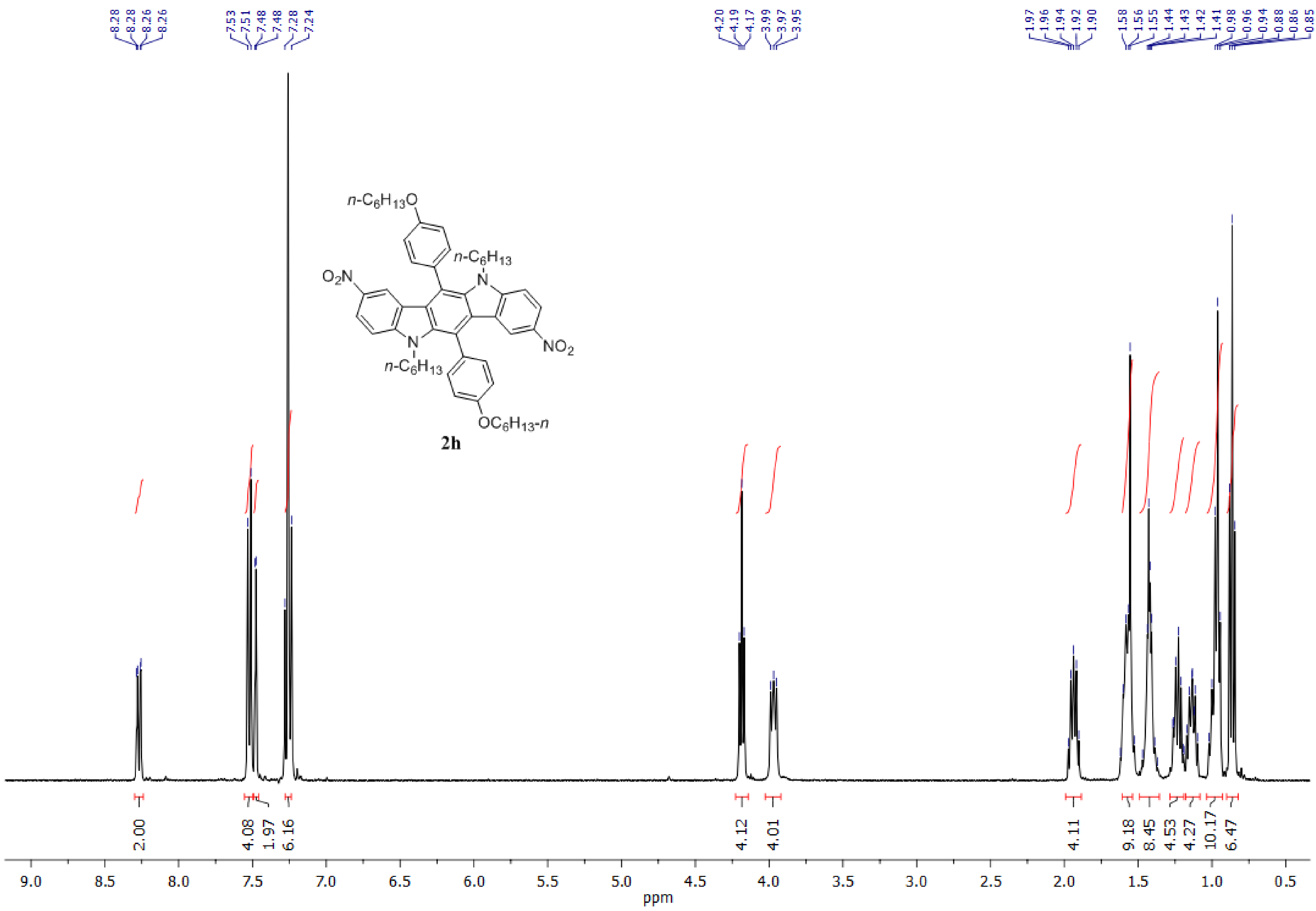

^{13}C NMR (solvent: $CDCl_3$)

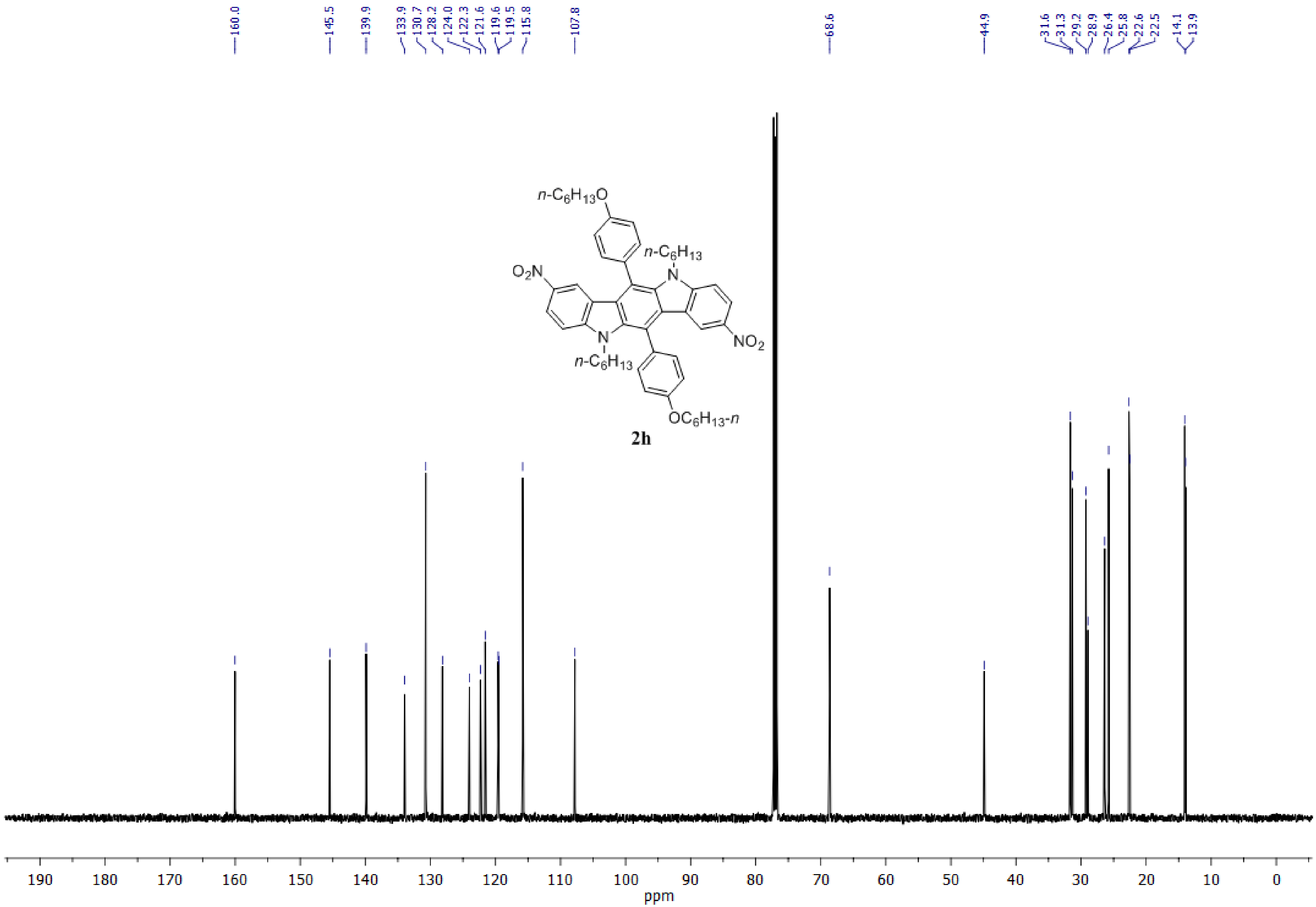

^{1}H NMR (solvent: $CDCl_3$)

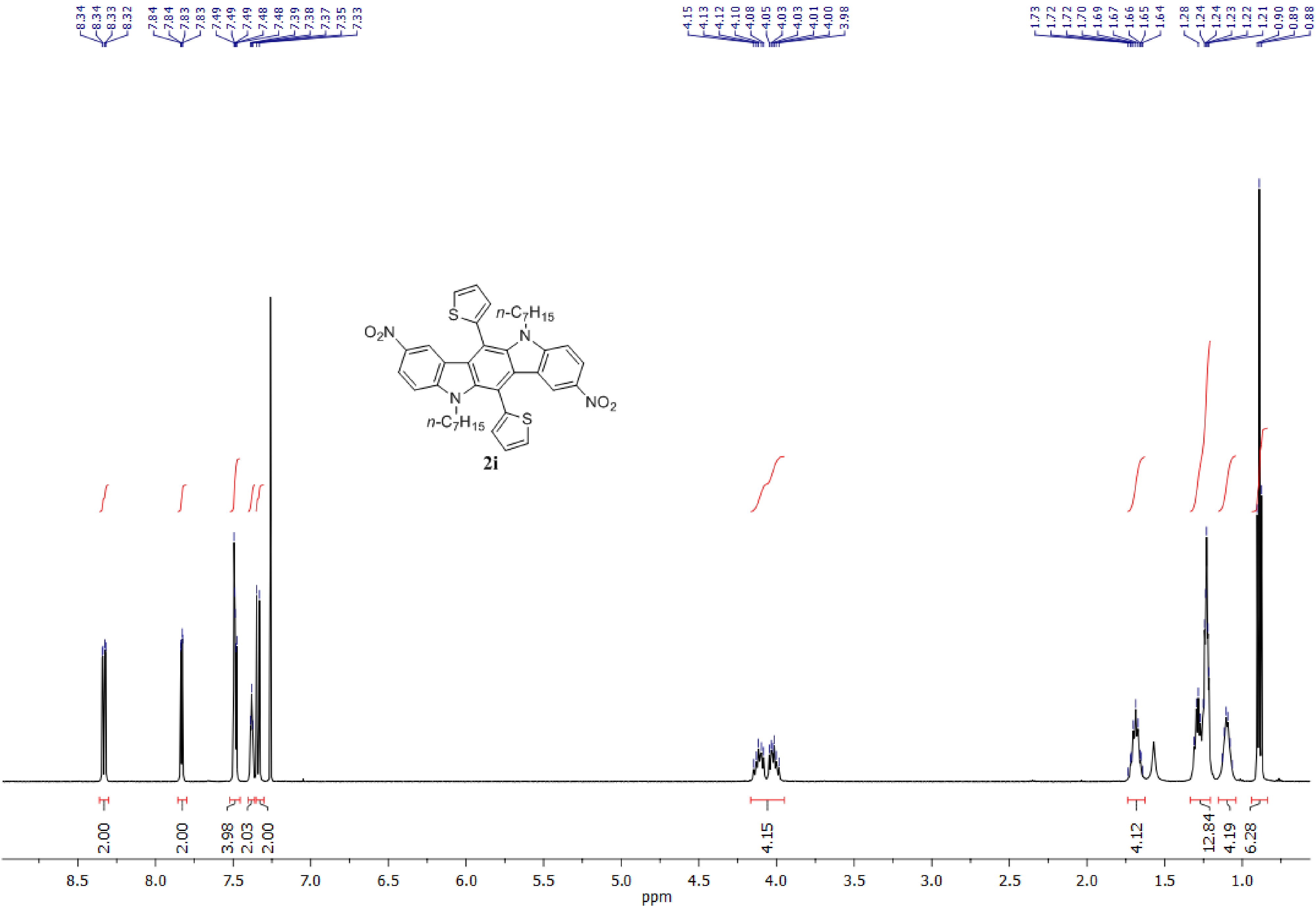

^{13}C NMR (solvent: $CDCl_3$)

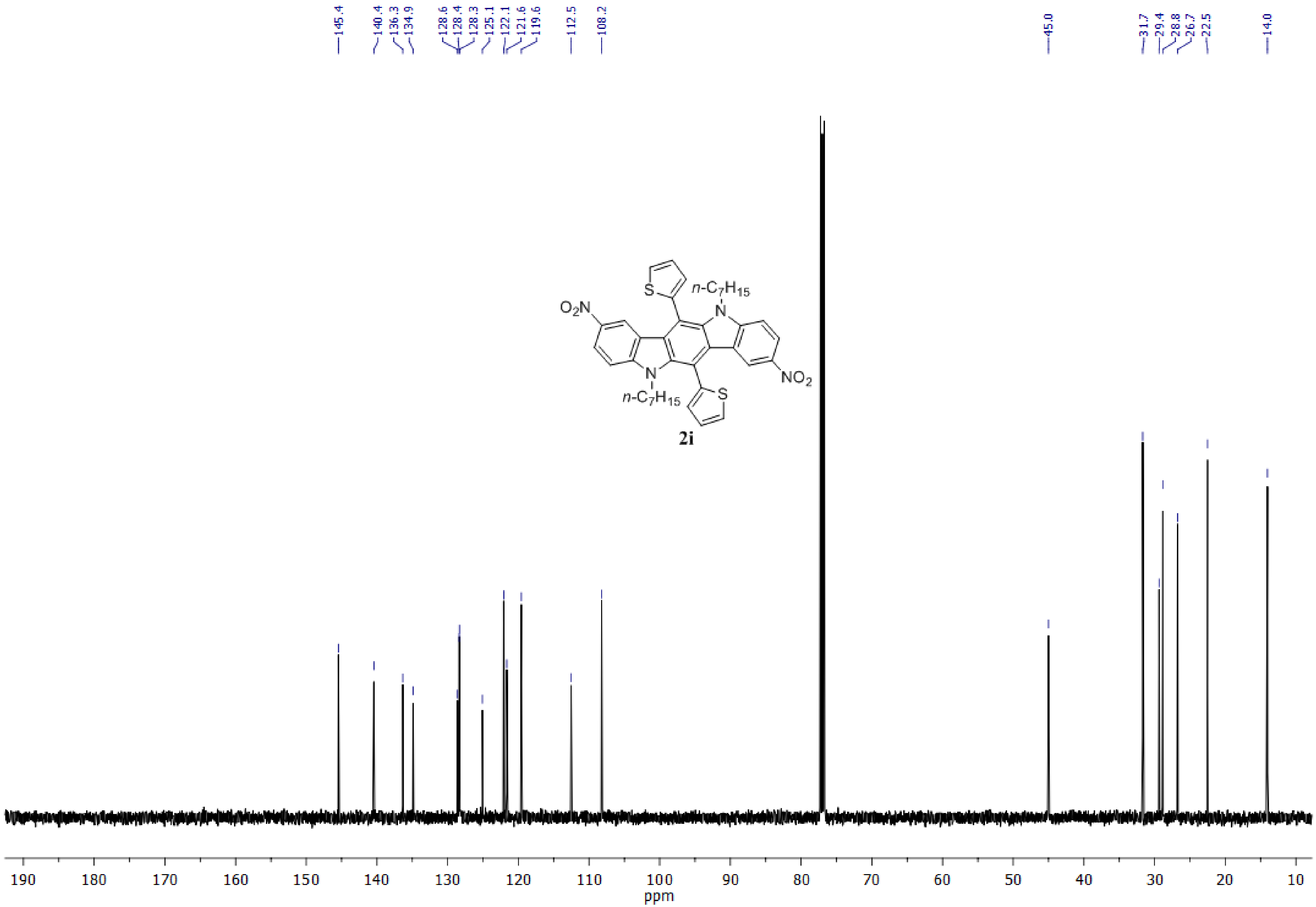

^{1}H NMR (solvent: $CDCl_3$)

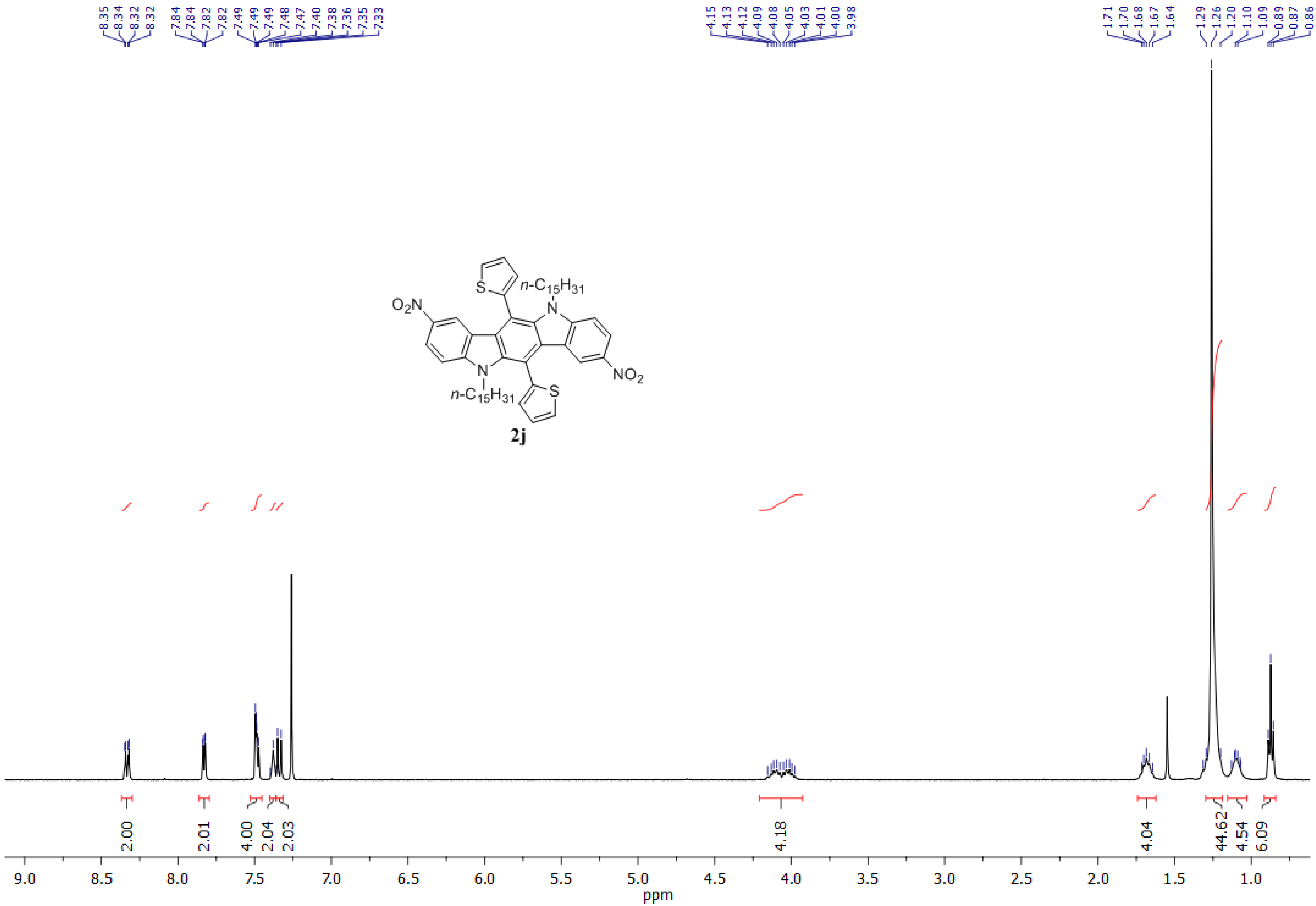

^{13}C NMR (solvent: $CDCl_3$)

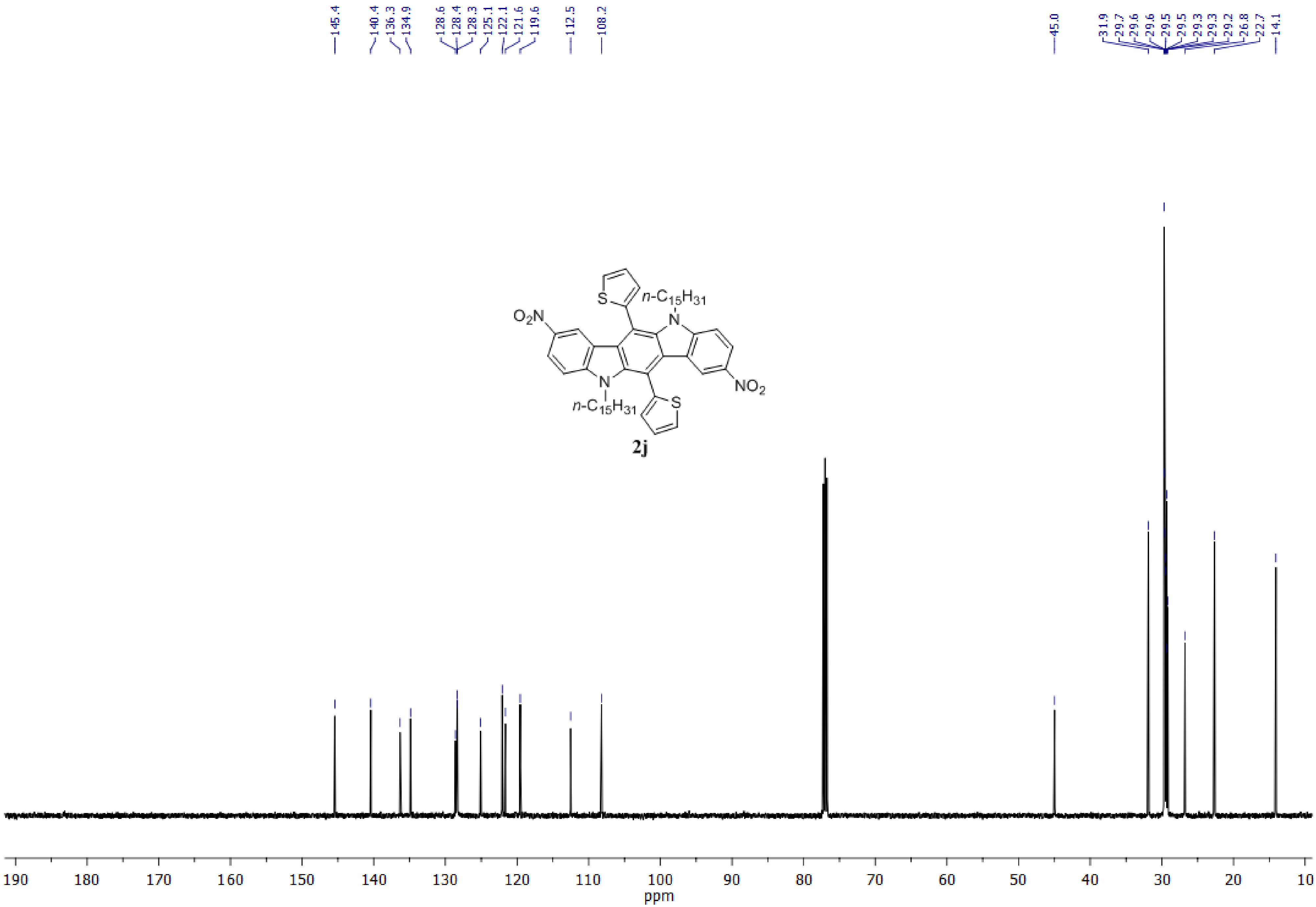

2j

^{1}**H NMR** (solvent: $CDCl_3$)

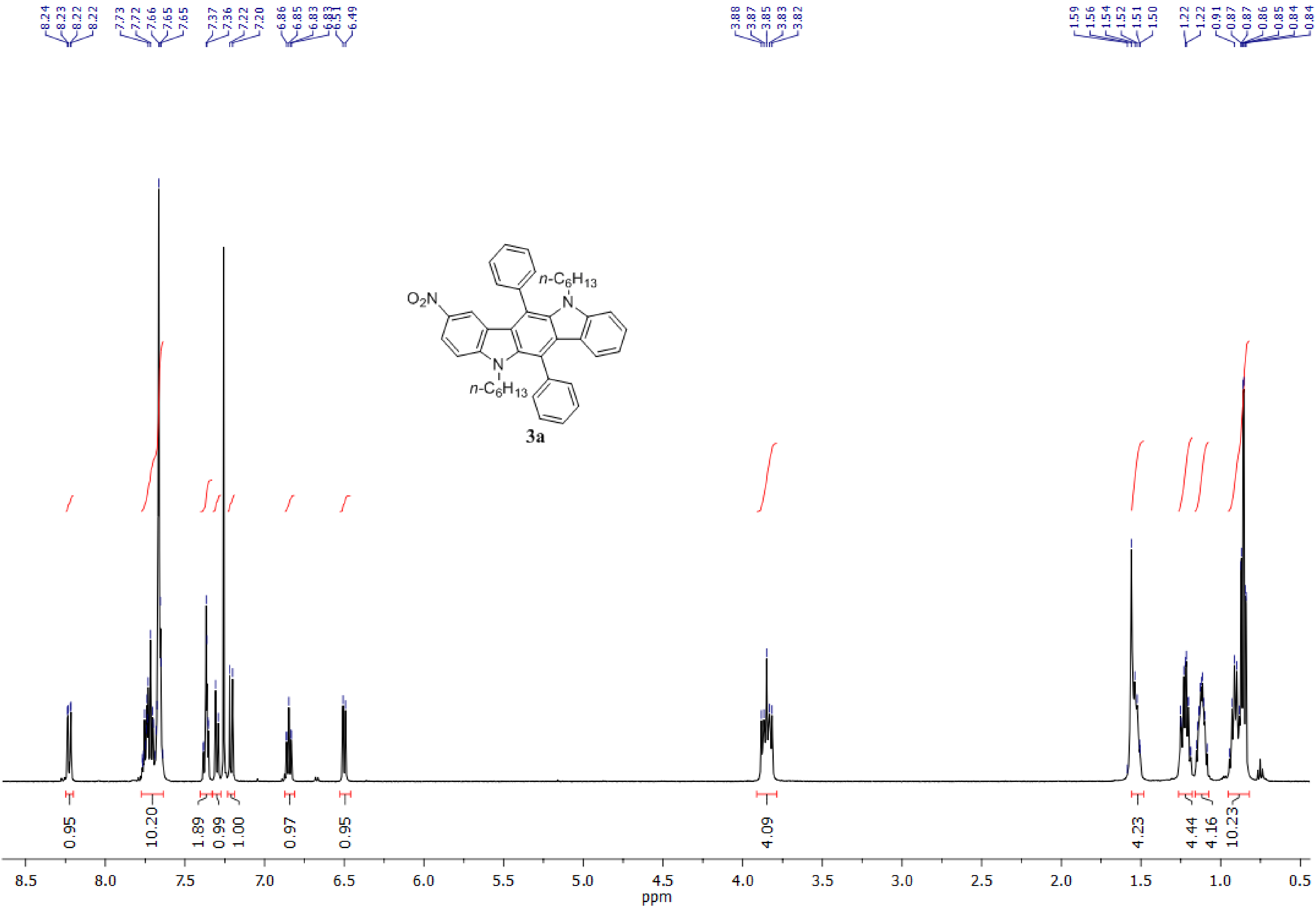

^{13}**C NMR** (solvent: $CDCl_3$)

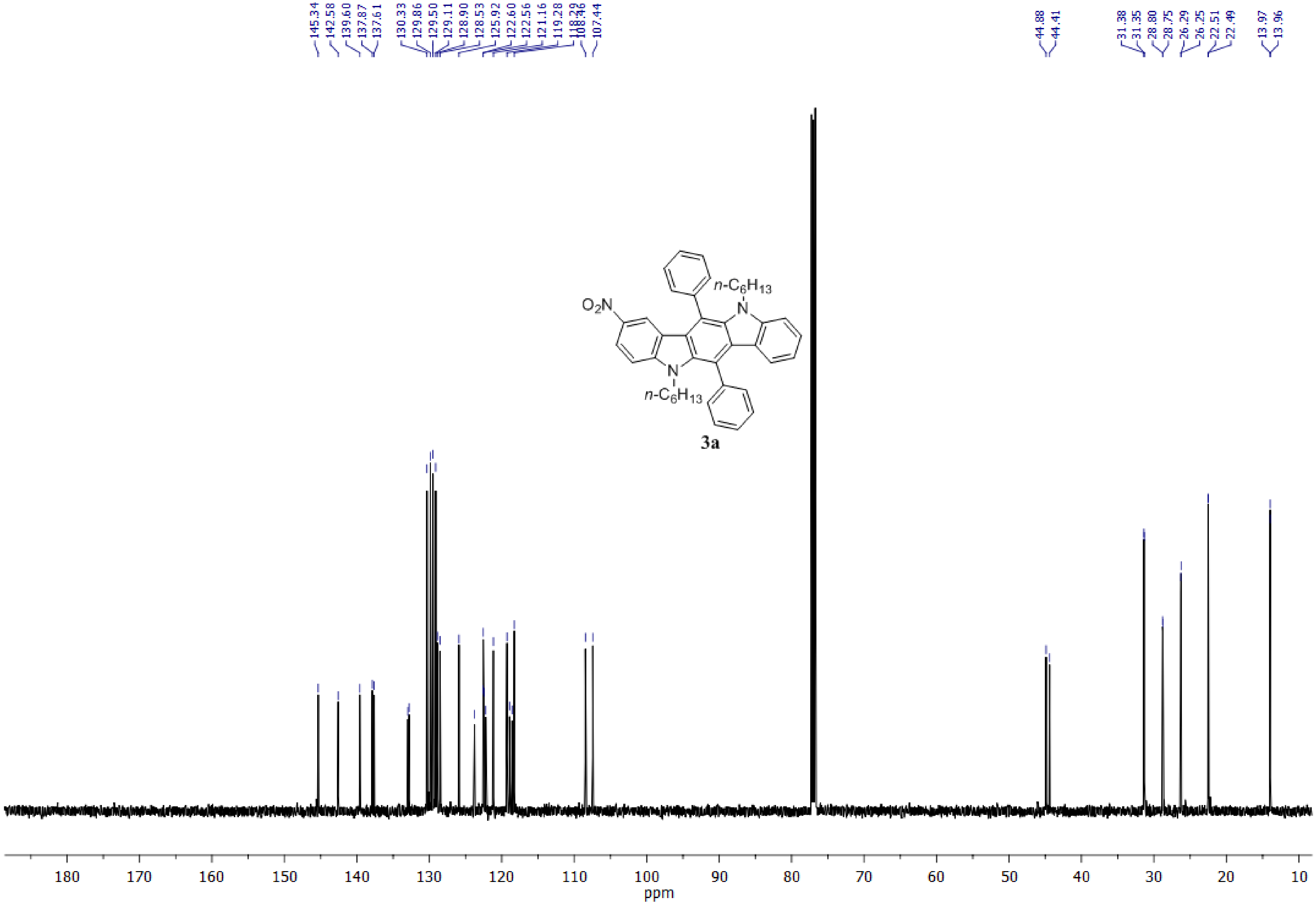

^{1}**H NMR** (solvent: $CDCl_3$)

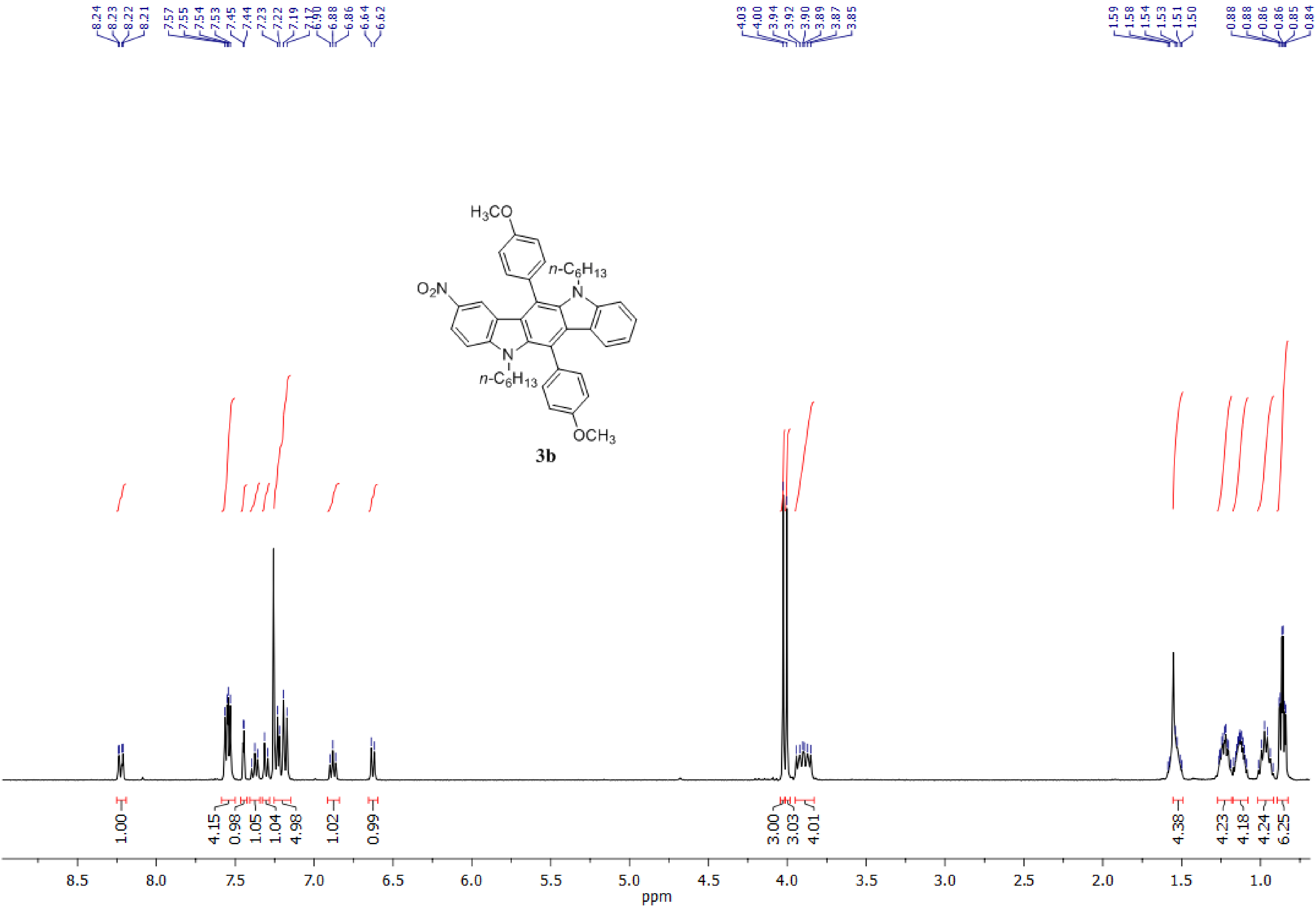

S48

^{13}C NMR (solvent: $CDCl_3$)

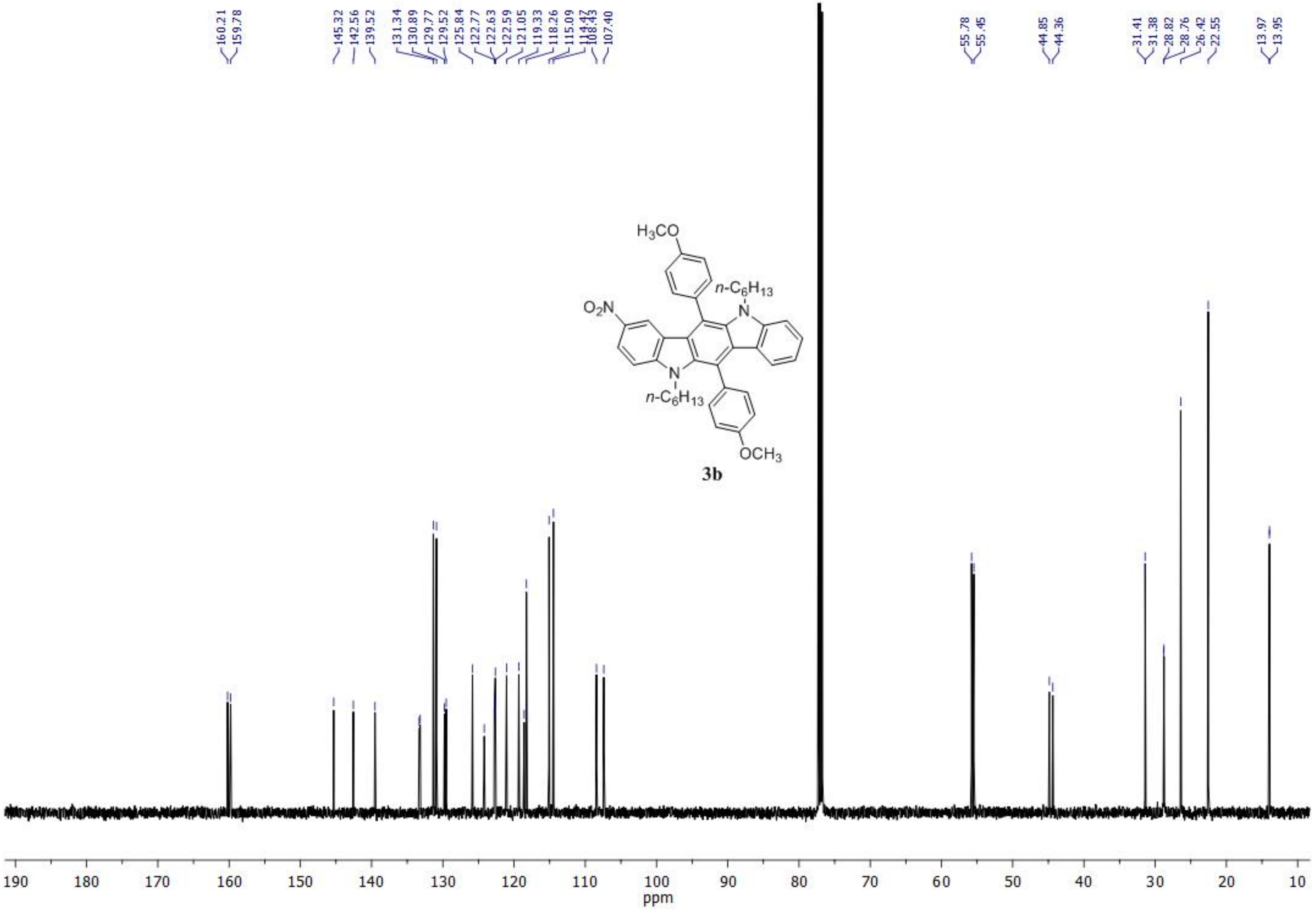

^{1}H NMR (solvent: $CDCl_3$)

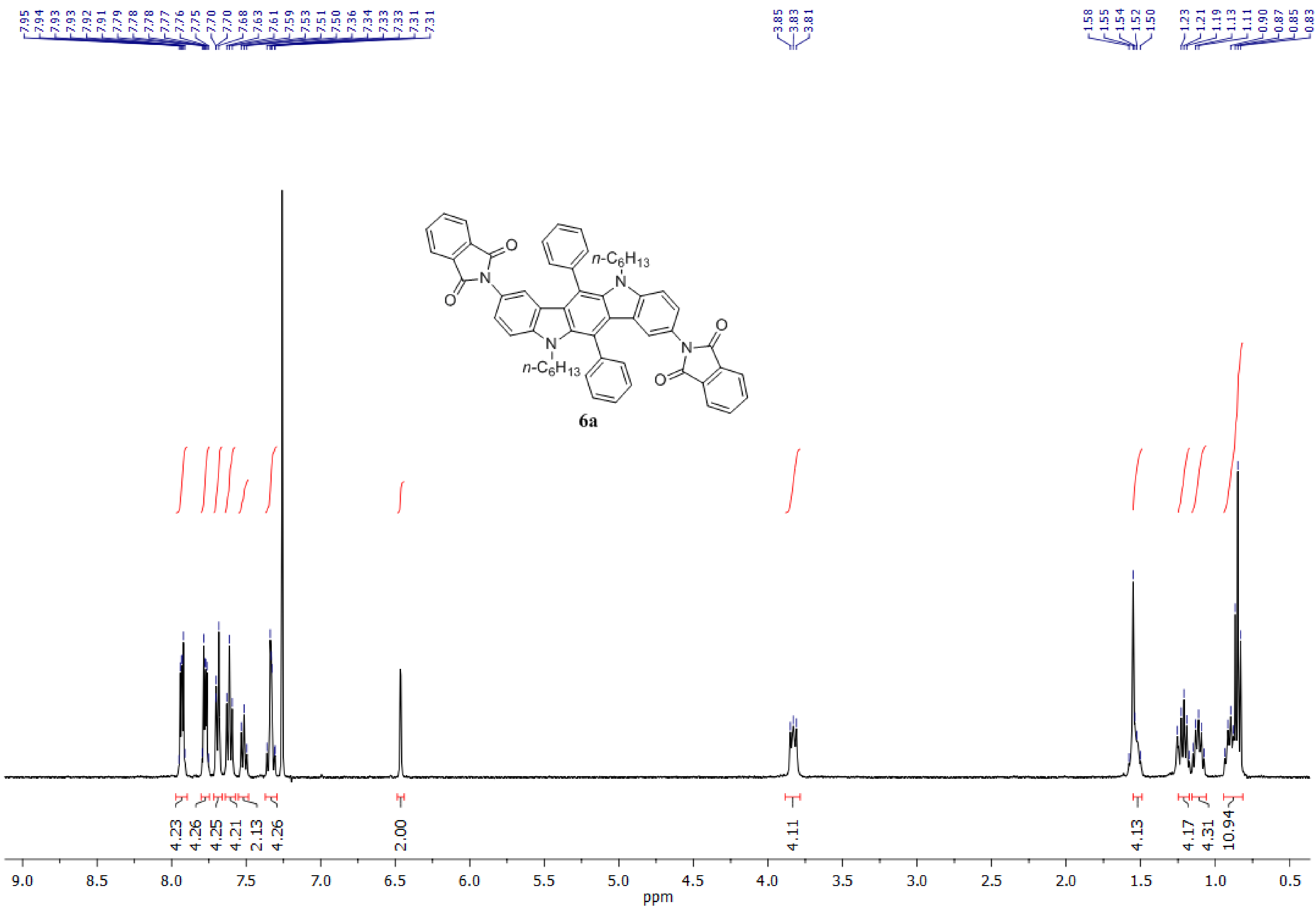

^{13}C NMR (solvent: $CDCl_3$)

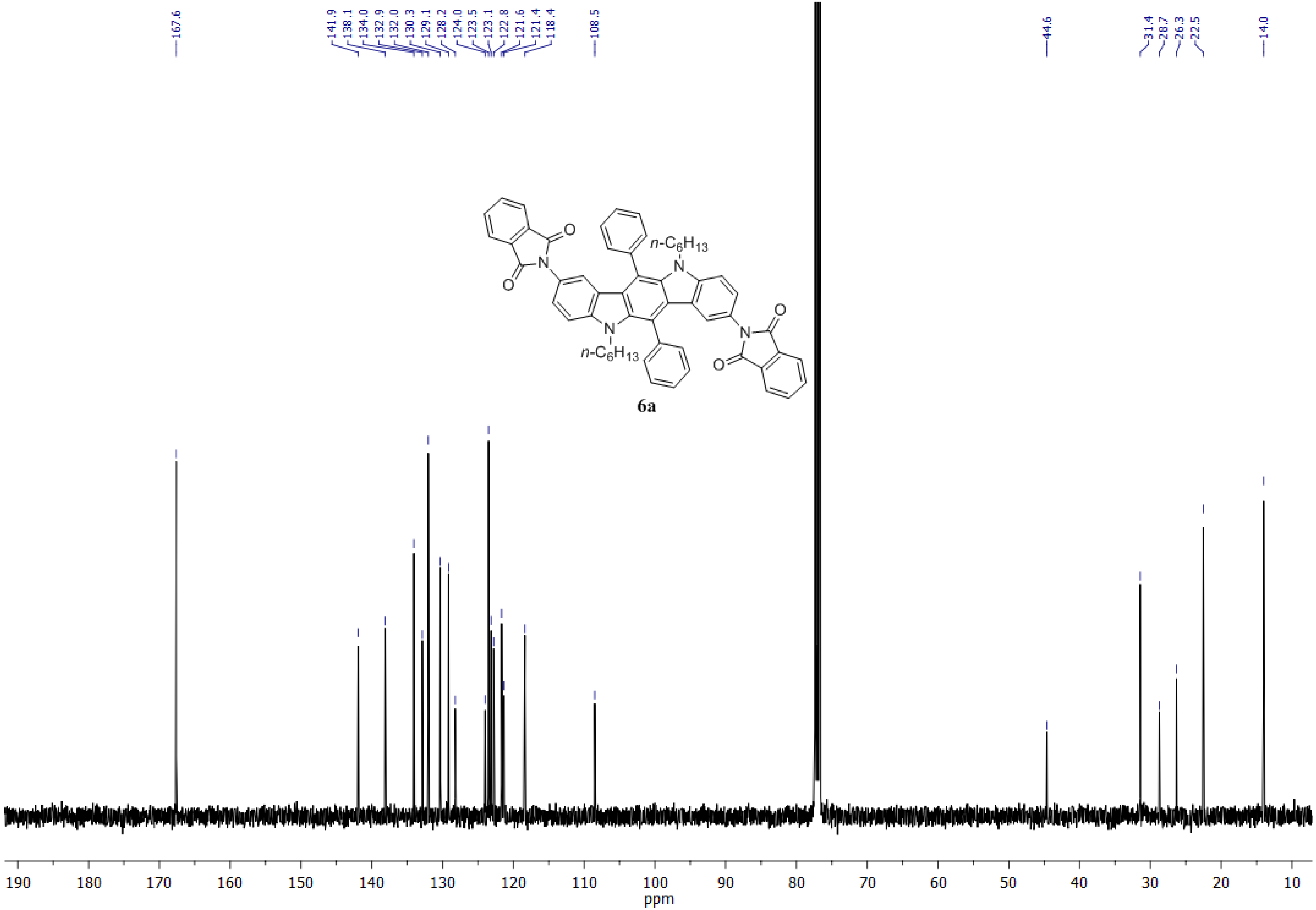

[1]**H NMR** (solvent: $CDCl_3$)

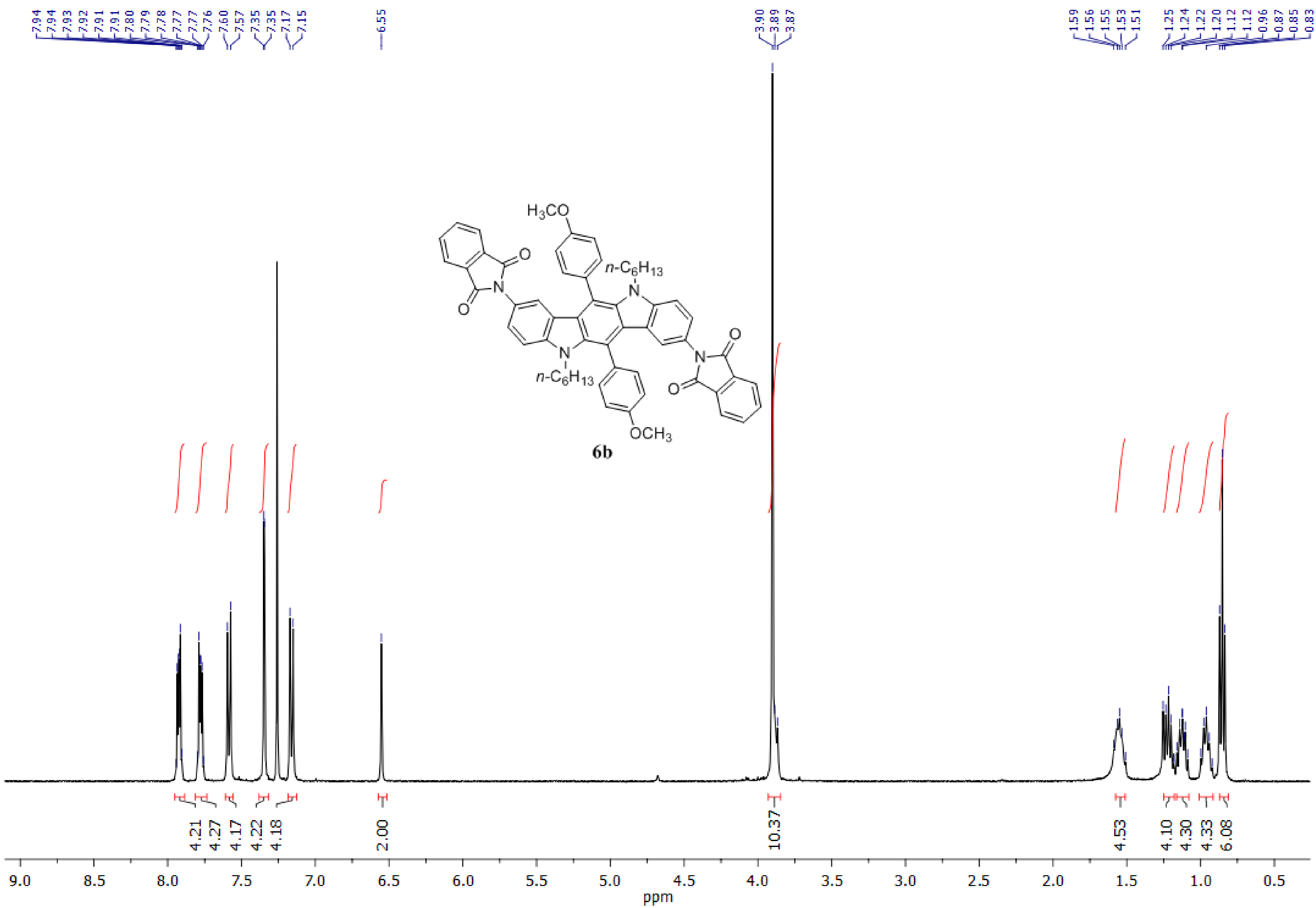

^{13}**C NMR** (solvent: $CDCl_3$)

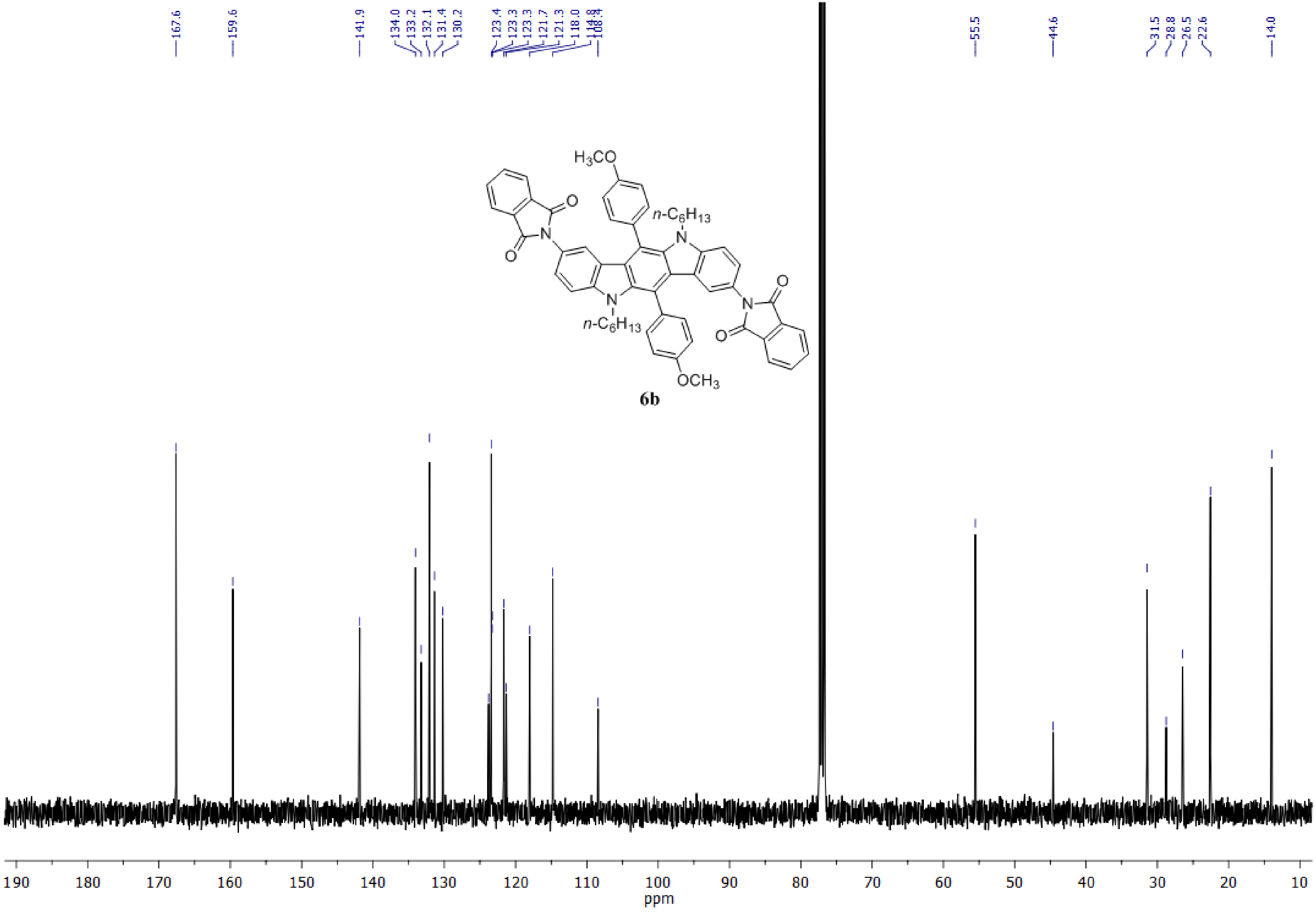

^{1}**H NMR** (solvent: $CDCl_3$)

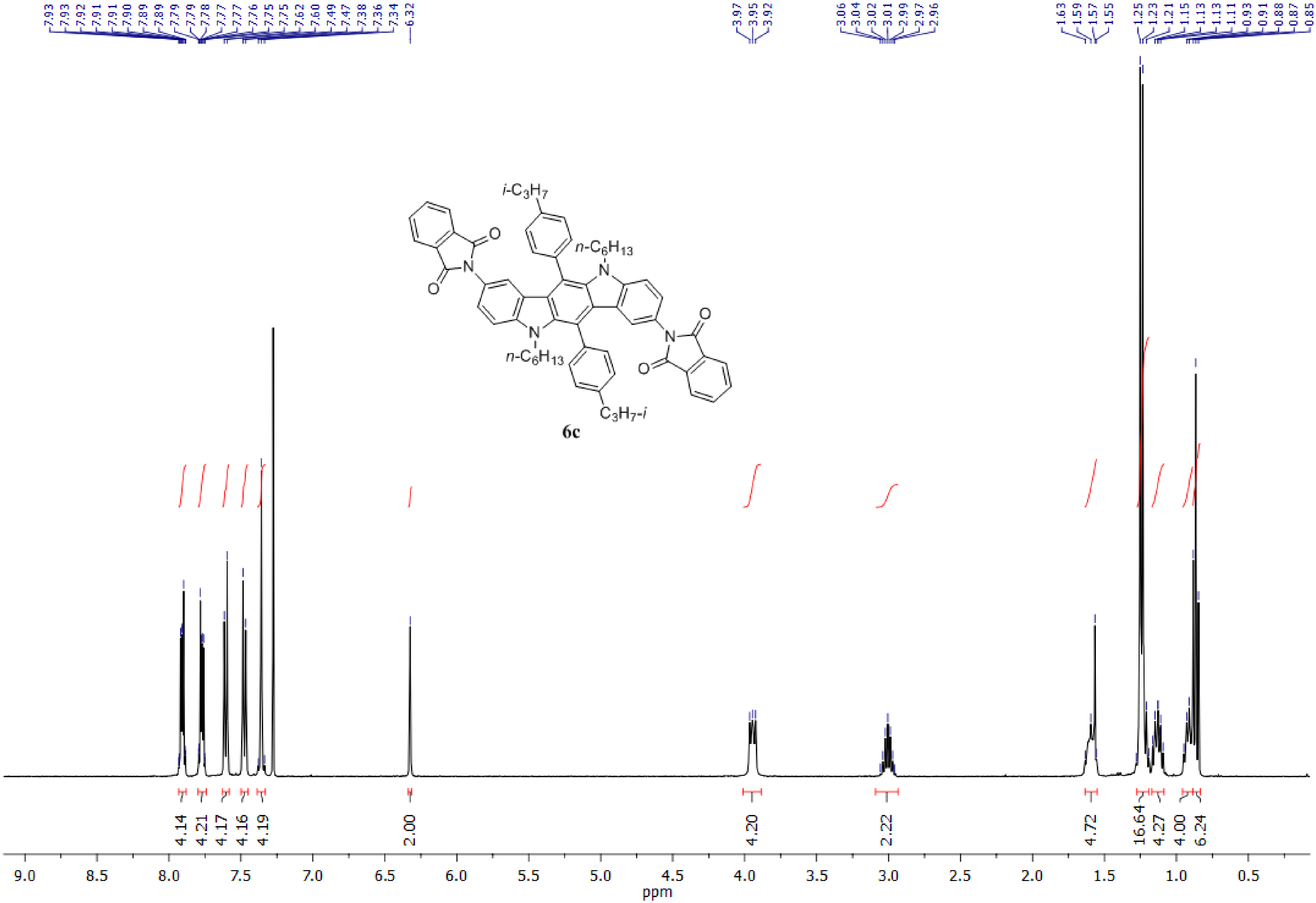

^{13}C NMR (solvent: $CDCl_3$)

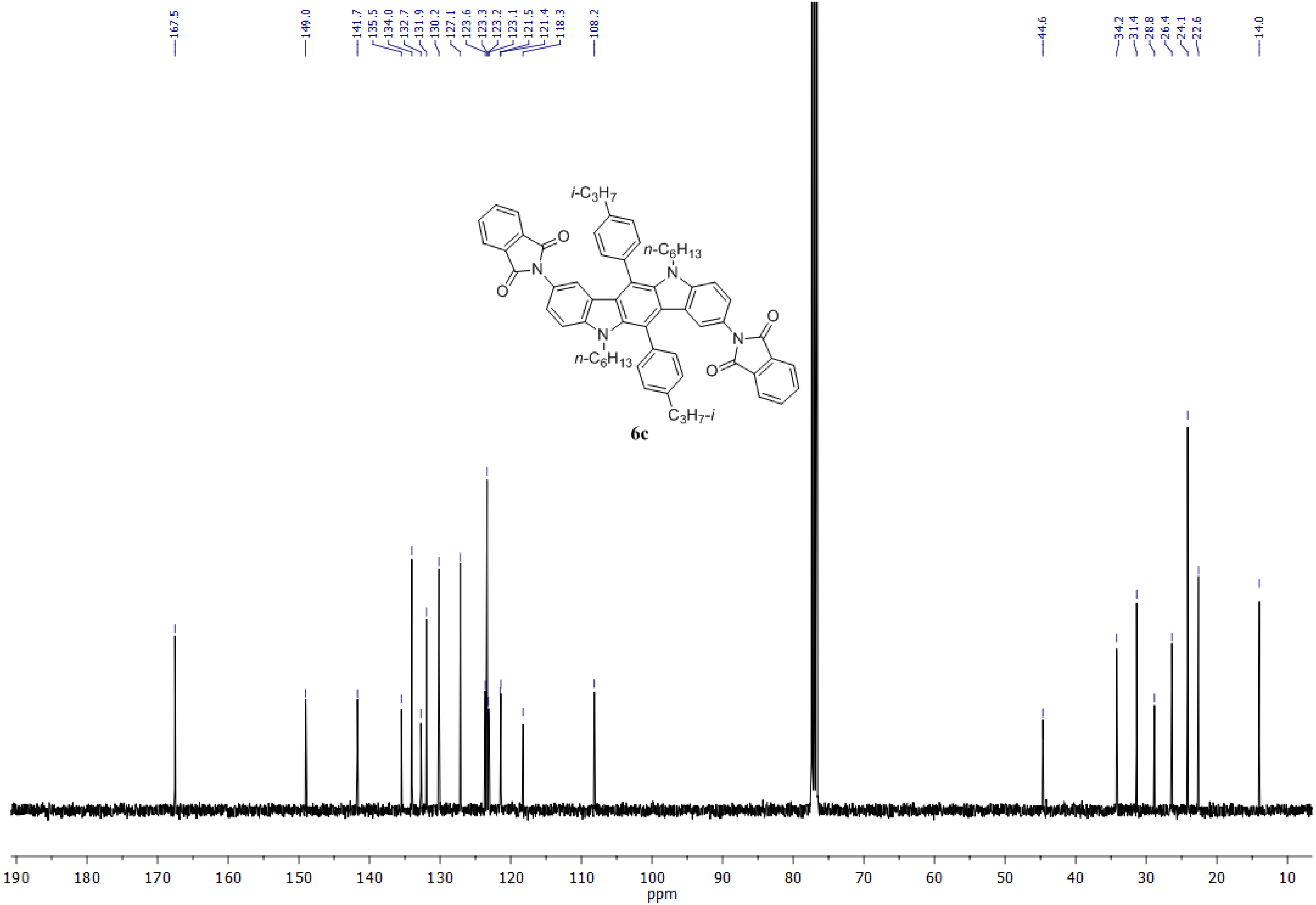

^{1}**H NMR** (solvent: $CDCl_3$)

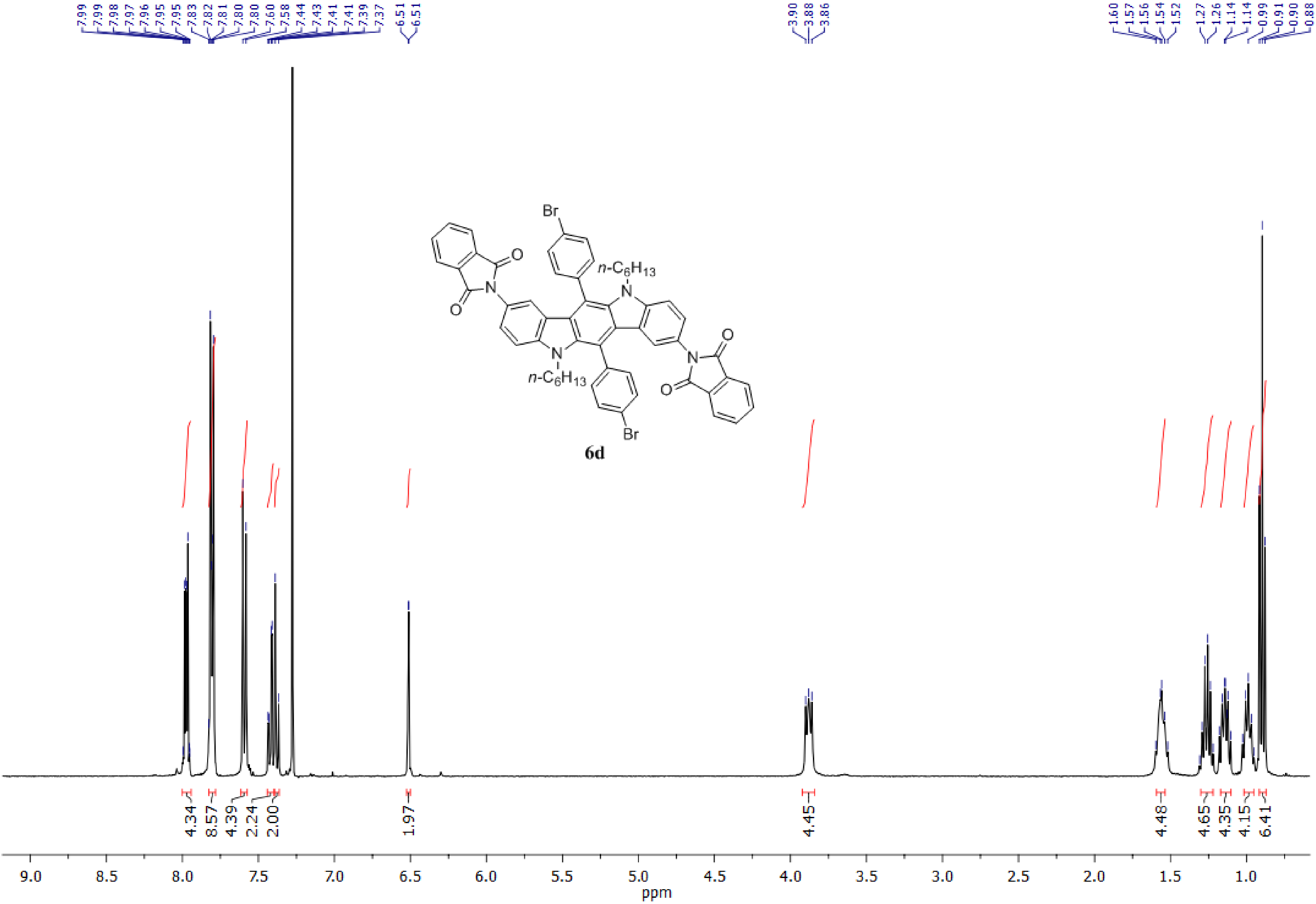

^{13}**C NMR** (solvent: $CDCl_3$)

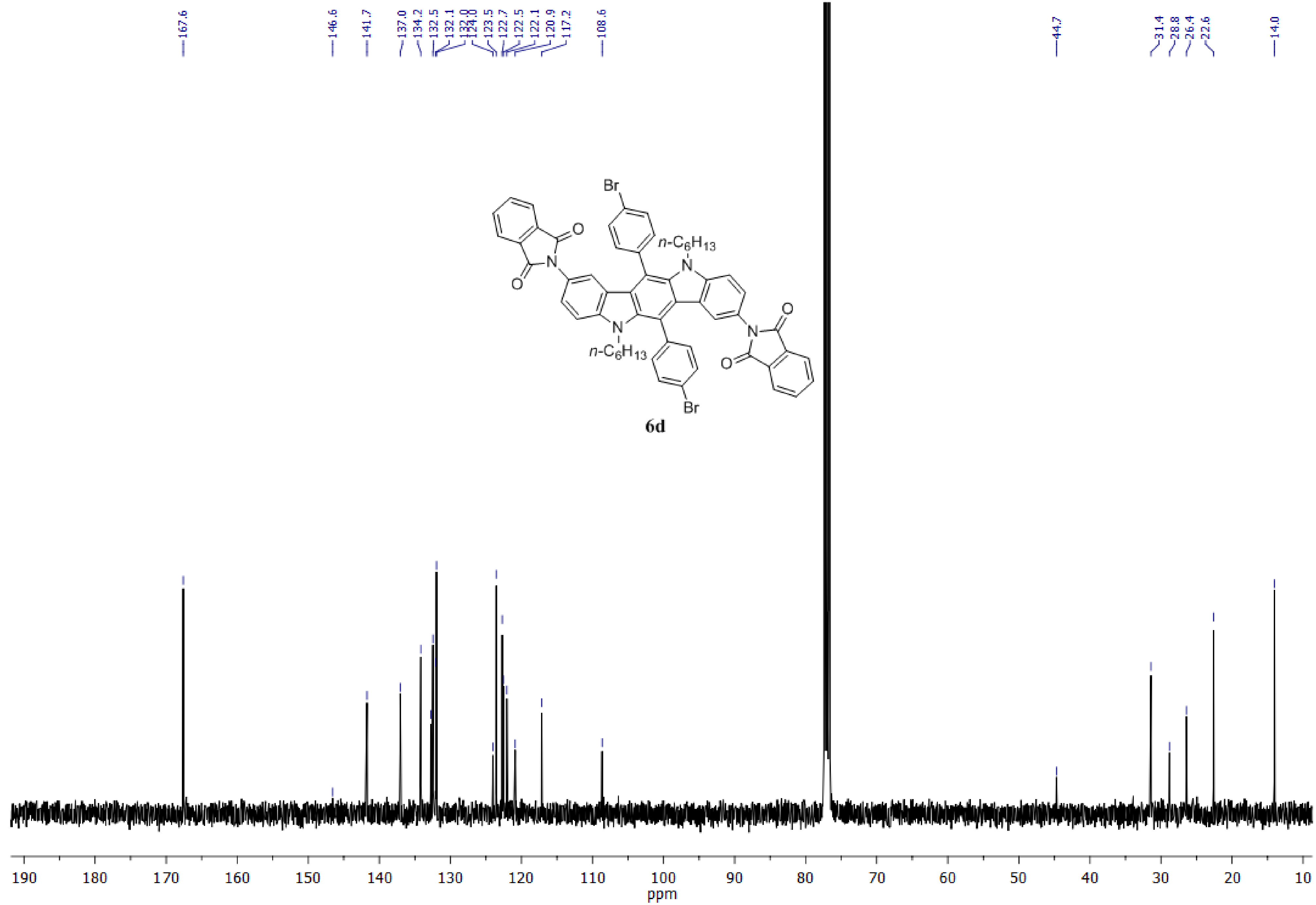

6d

^{1}H NMR (solvent: $CDCl_3$)

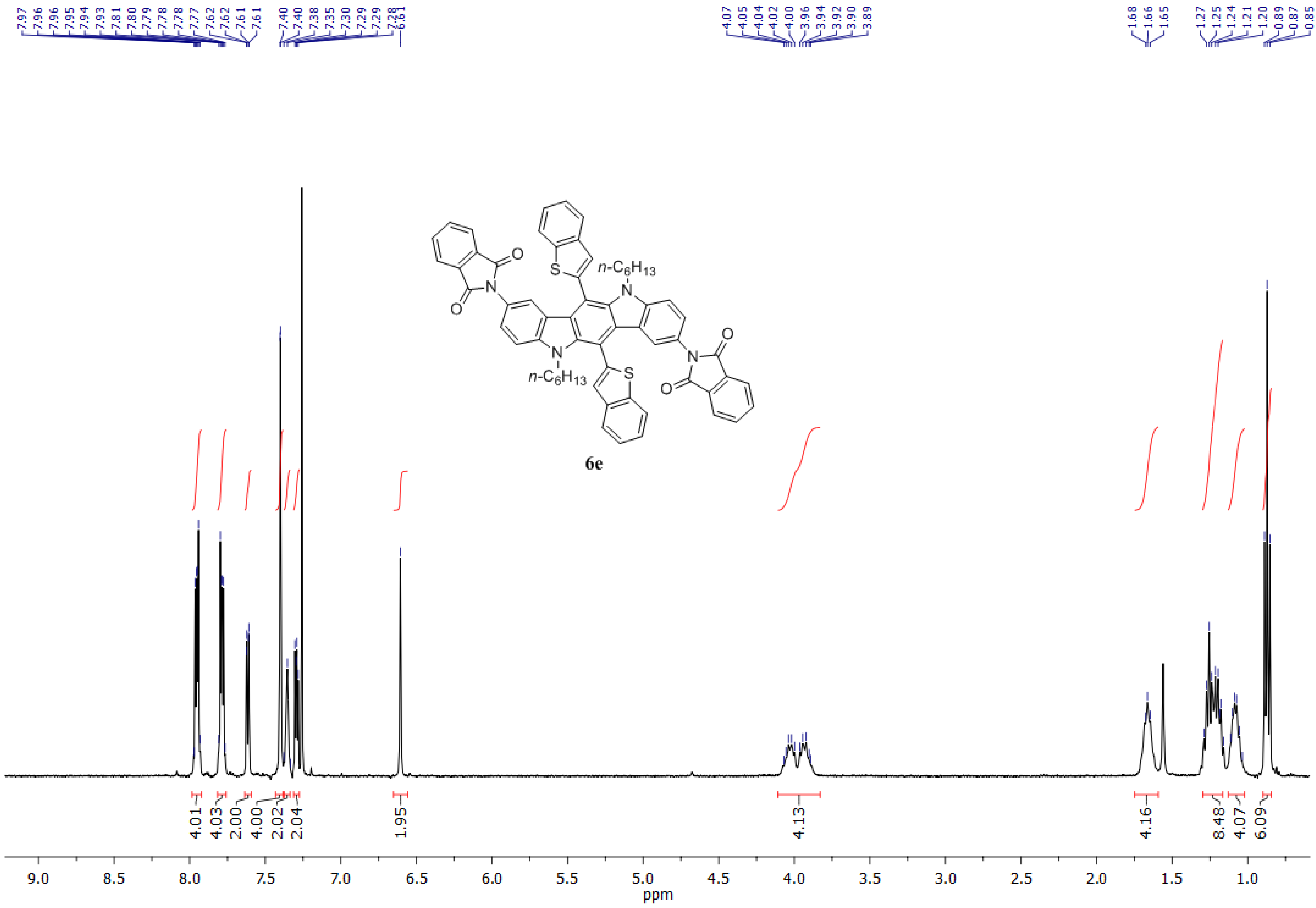

^{13}C NMR (solvent: $CDCl_3$)

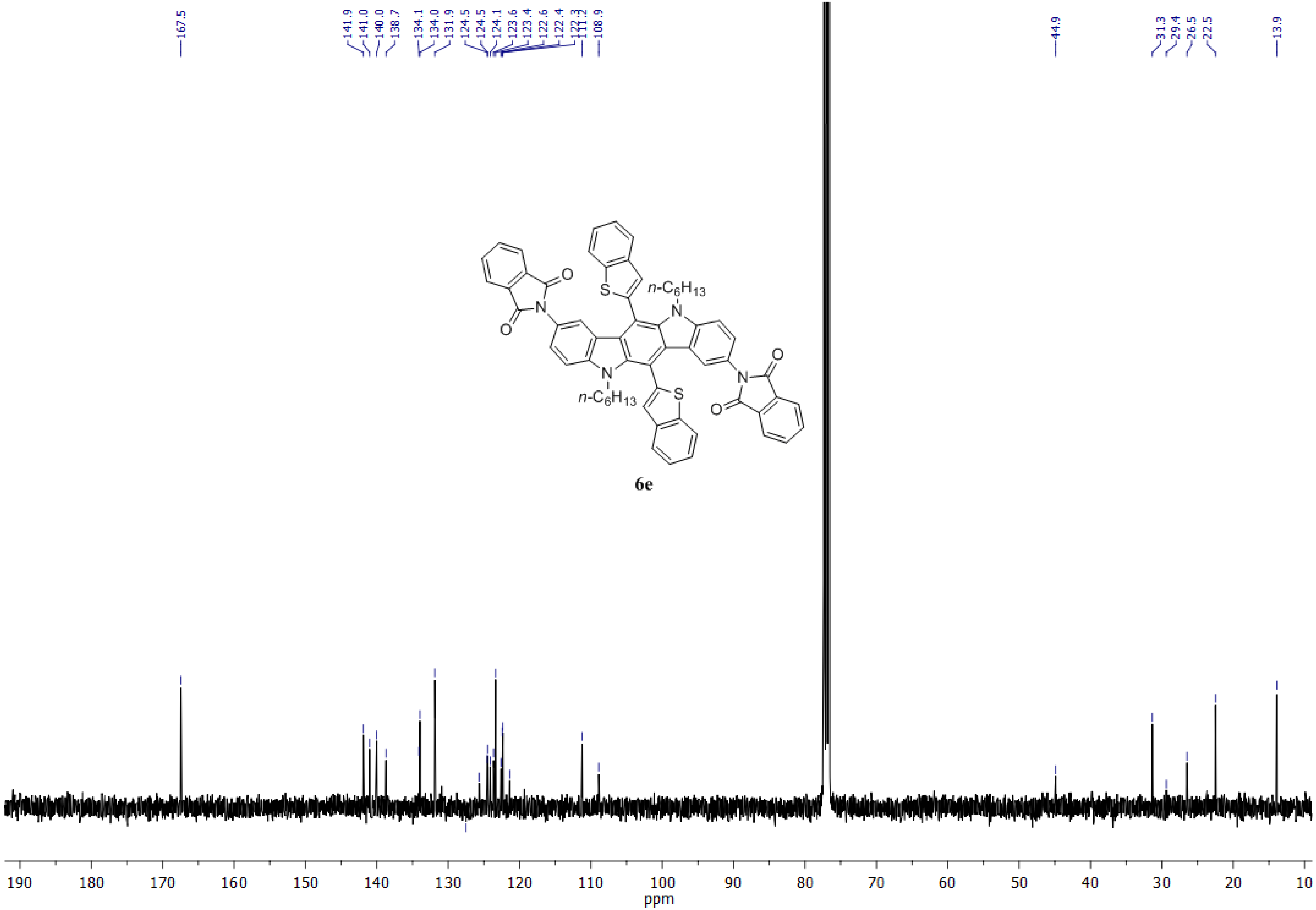

^{1}H NMR (solvent: $CDCl_3$)

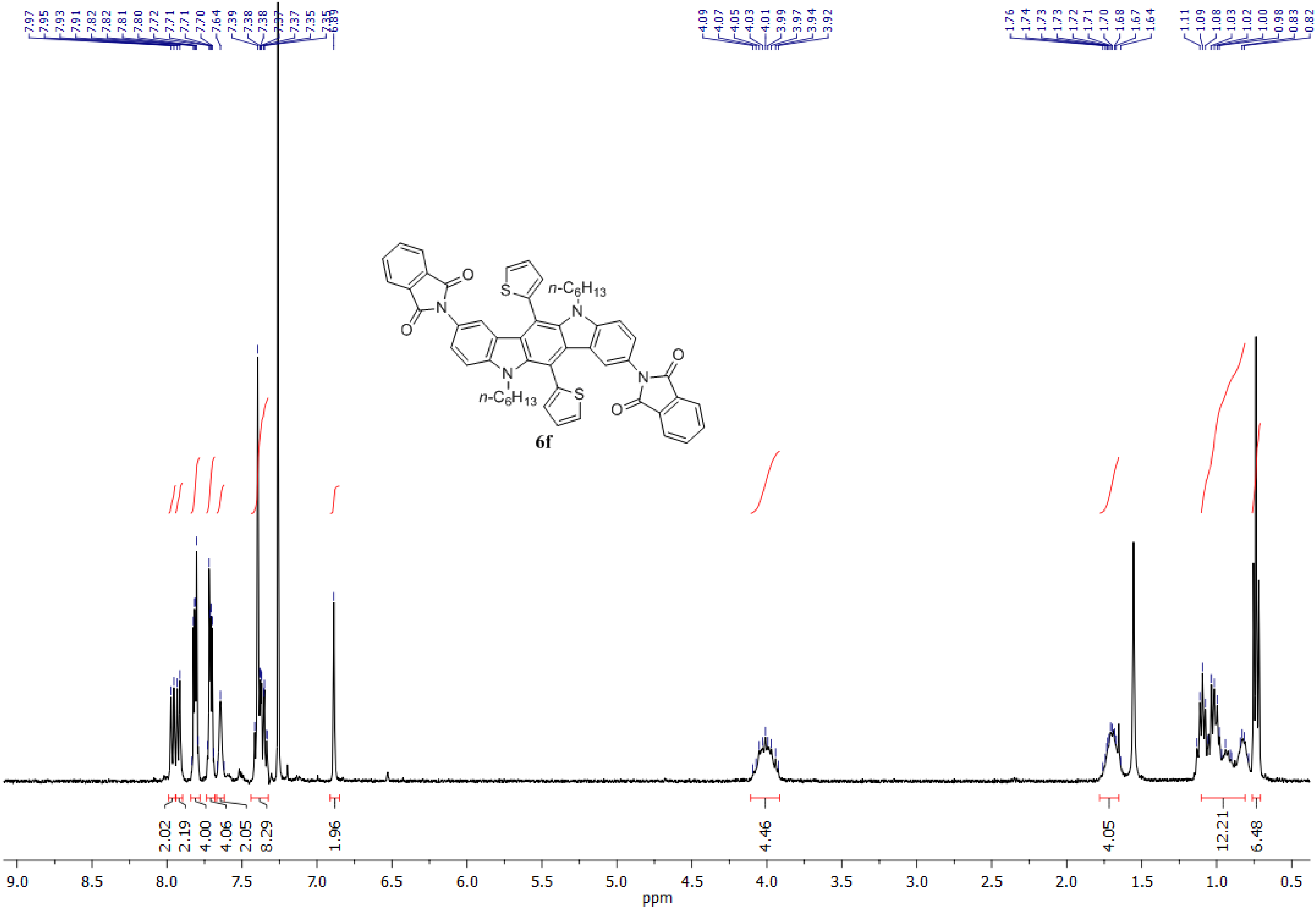

^{13}**C NMR** (solvent: $CDCl_3$)

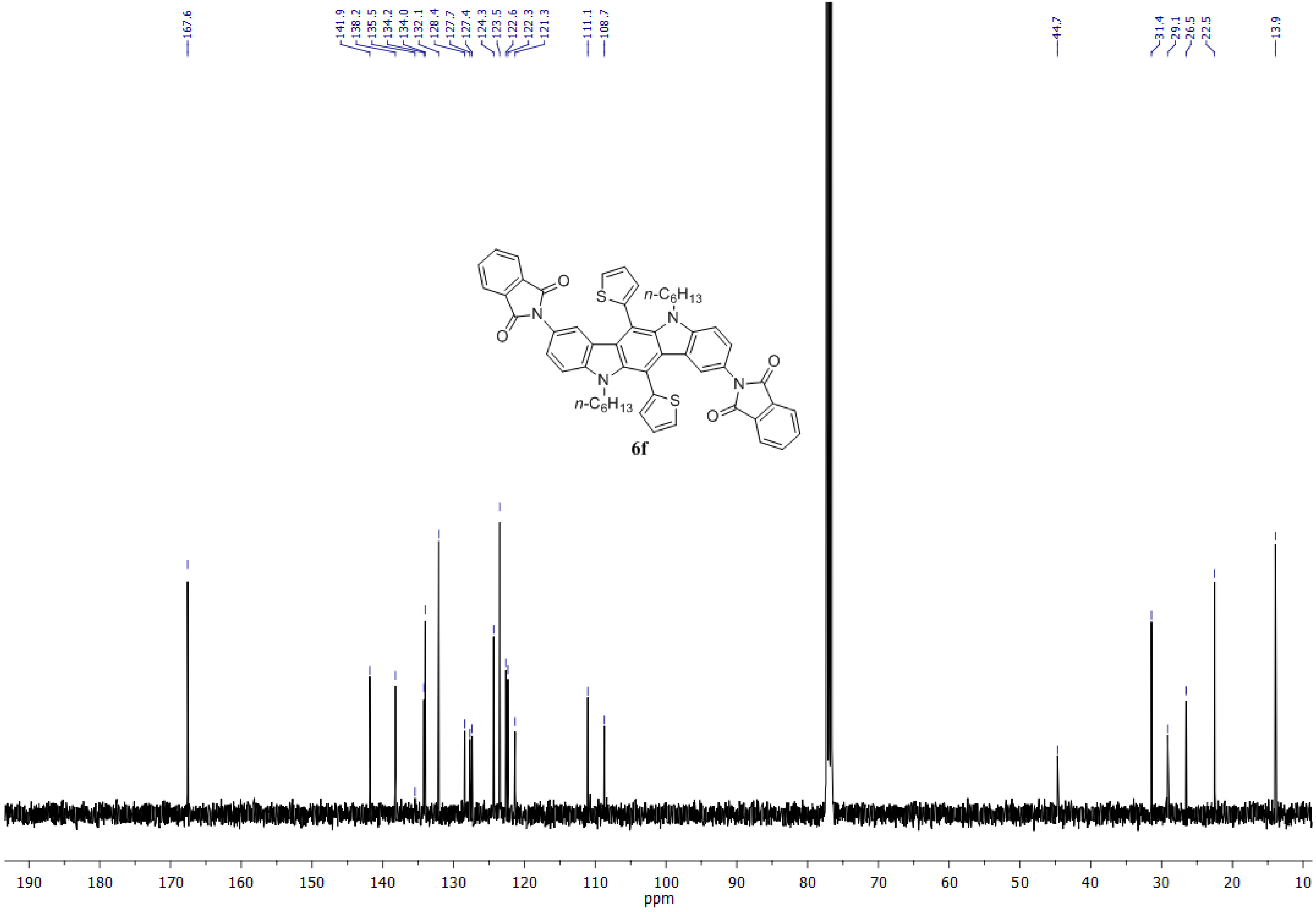

^{1}**H NMR** (solvent: $CDCl_3$)

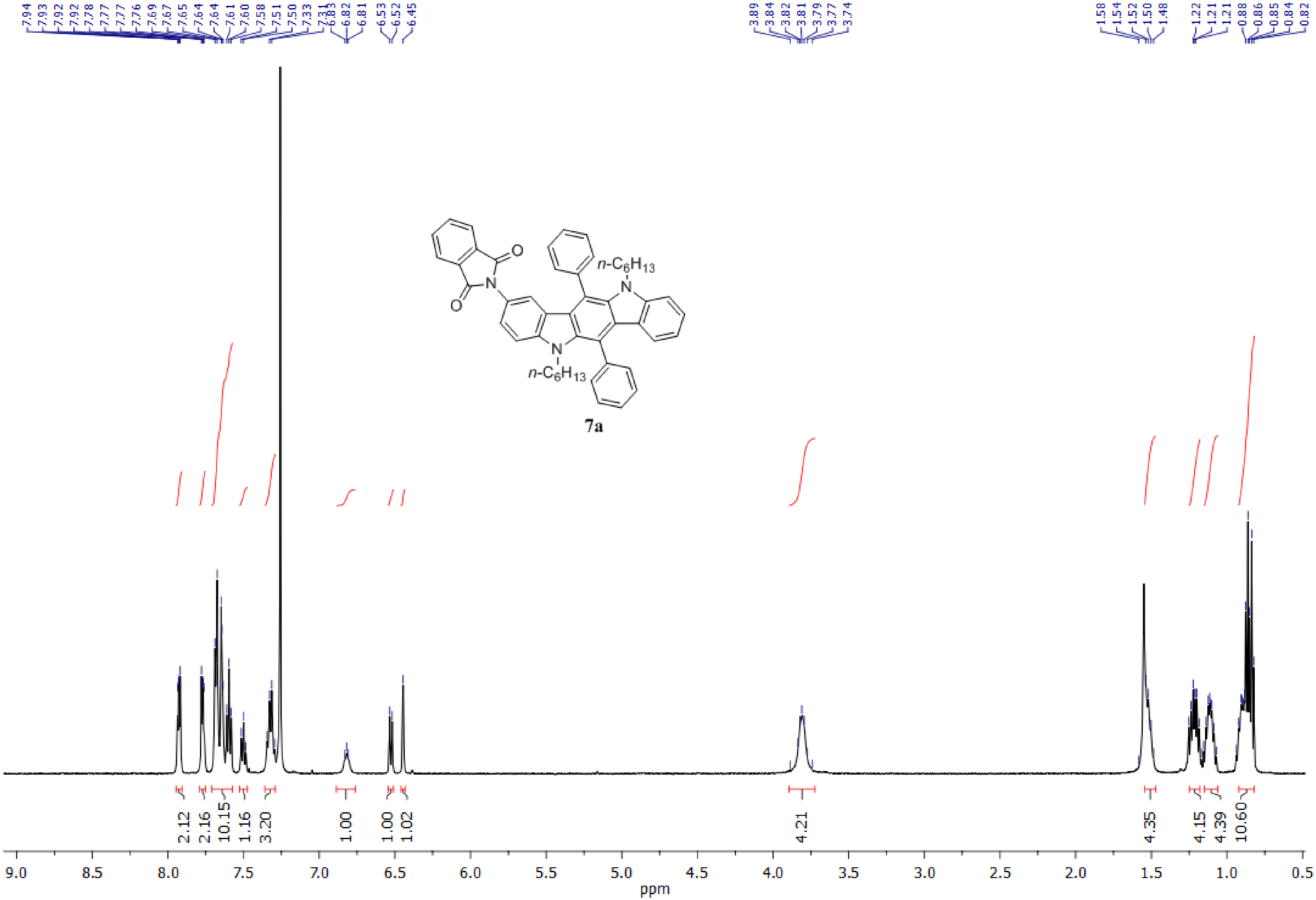

^{13}C NMR (solvent: C_6D_6)

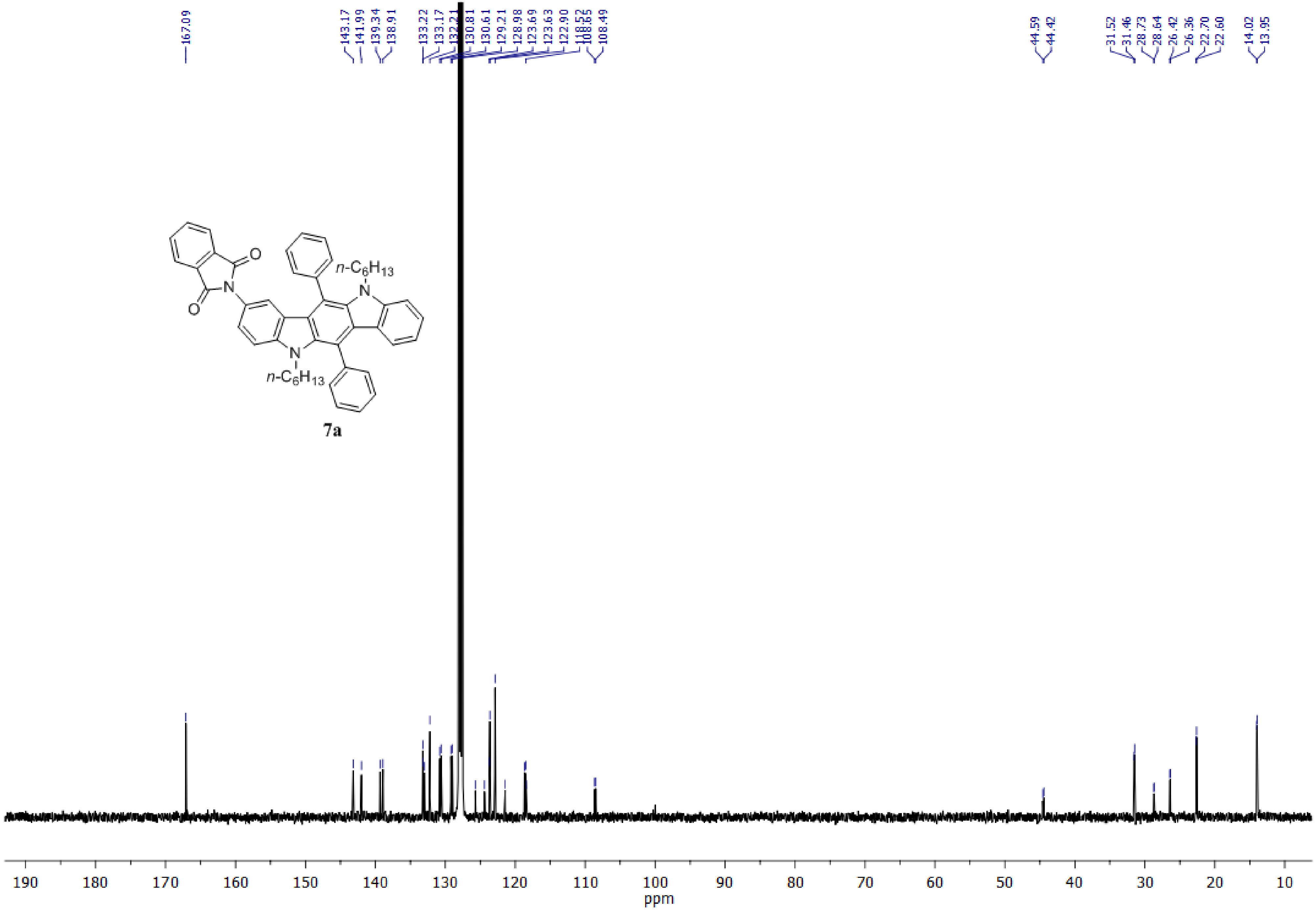

^{1}**H NMR** (solvent: C_6D_6)

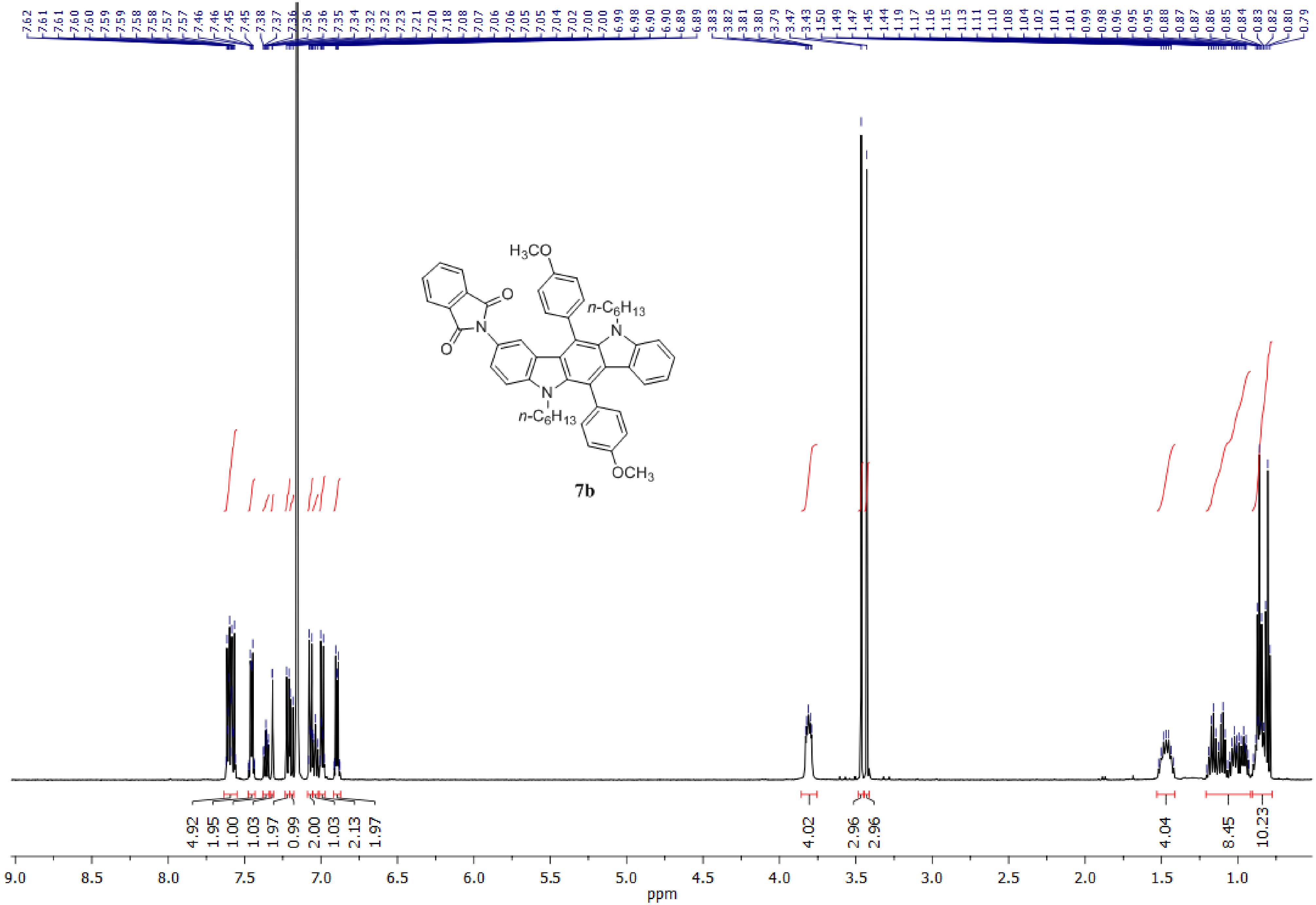

7b

^{13}**C NMR** (solvent: C_6D_6)

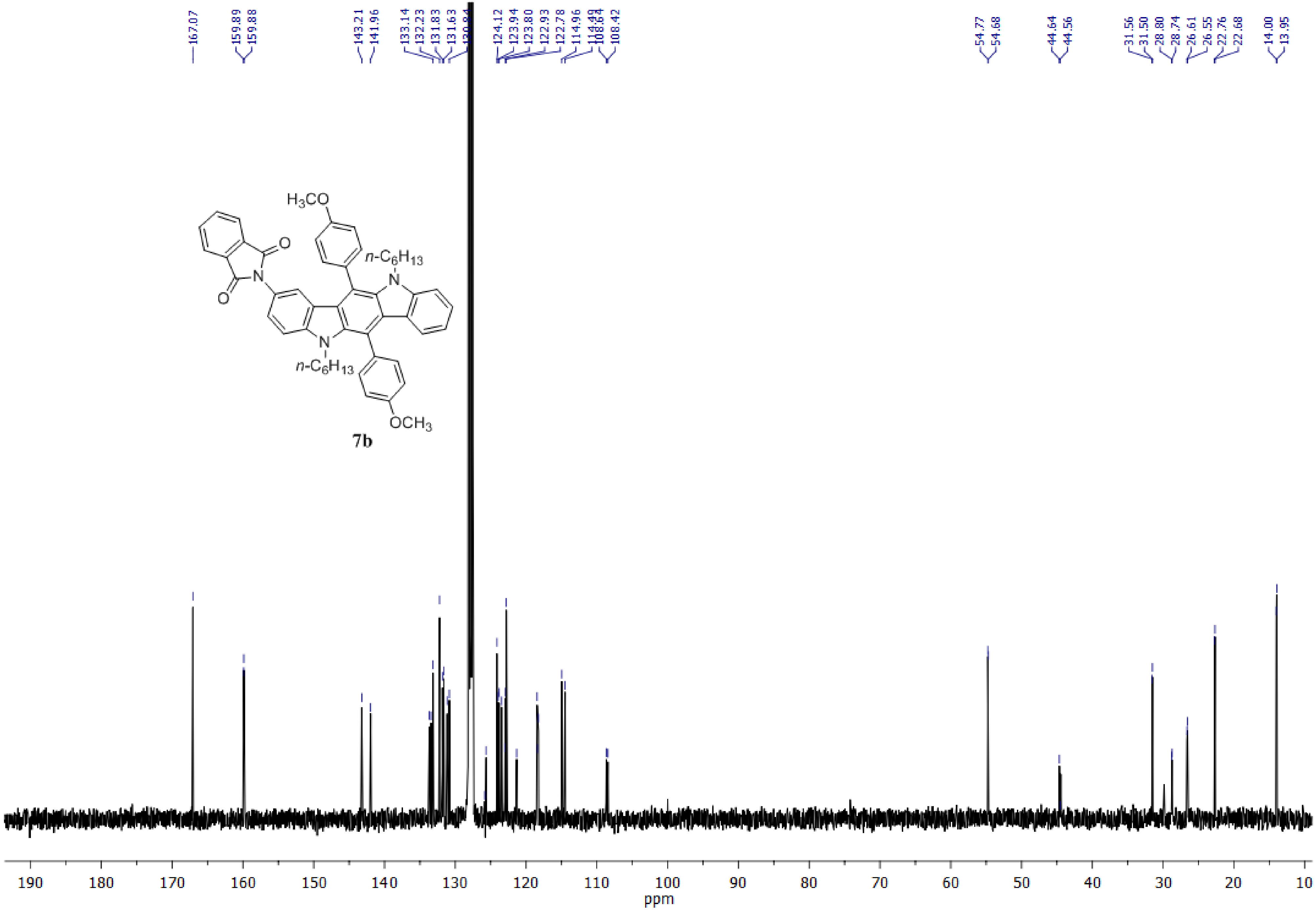

^{1}**H NMR** (solvent: $CDCl_3$)

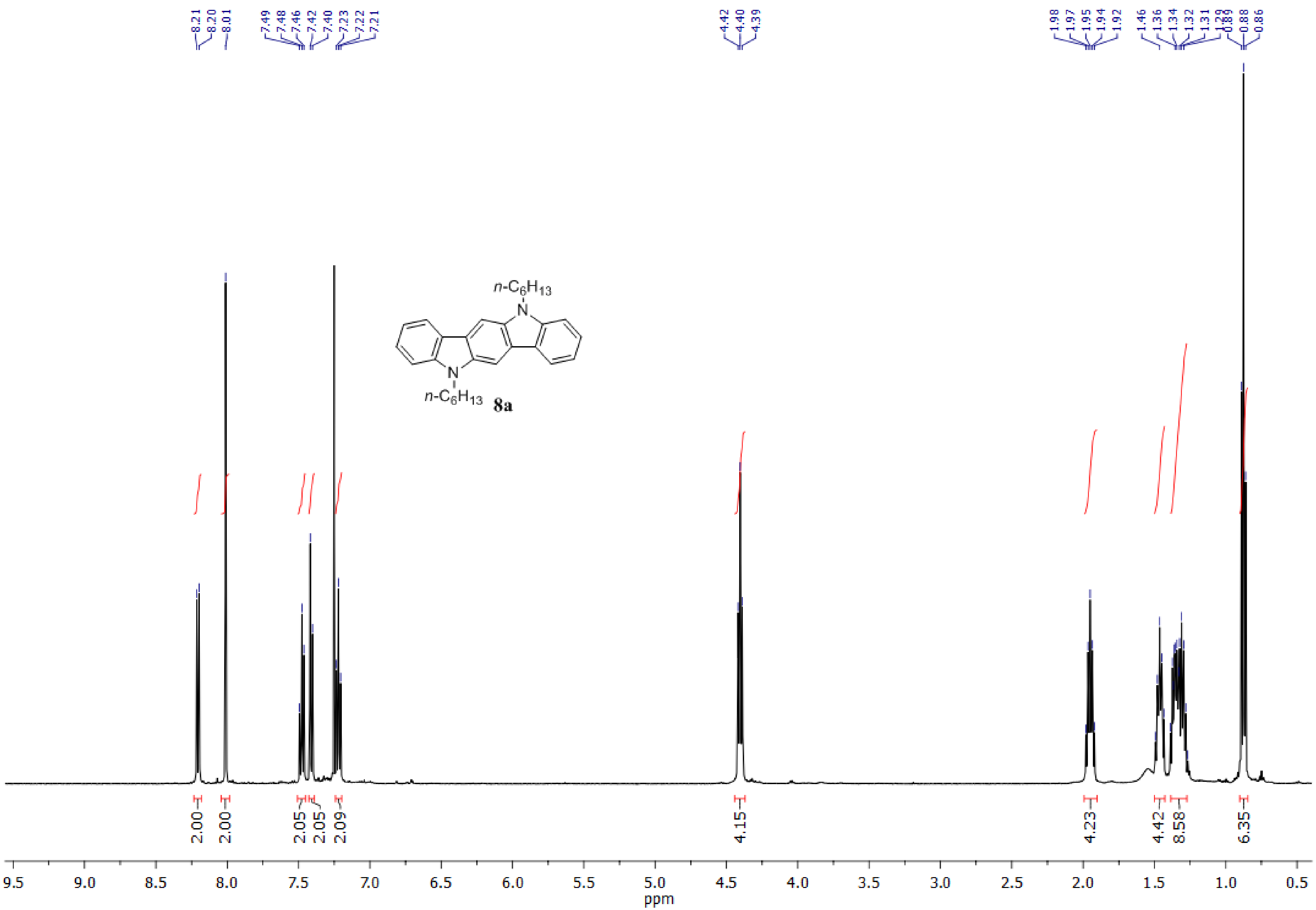

[1]**H NMR** (solvent: $CDCl_3$)

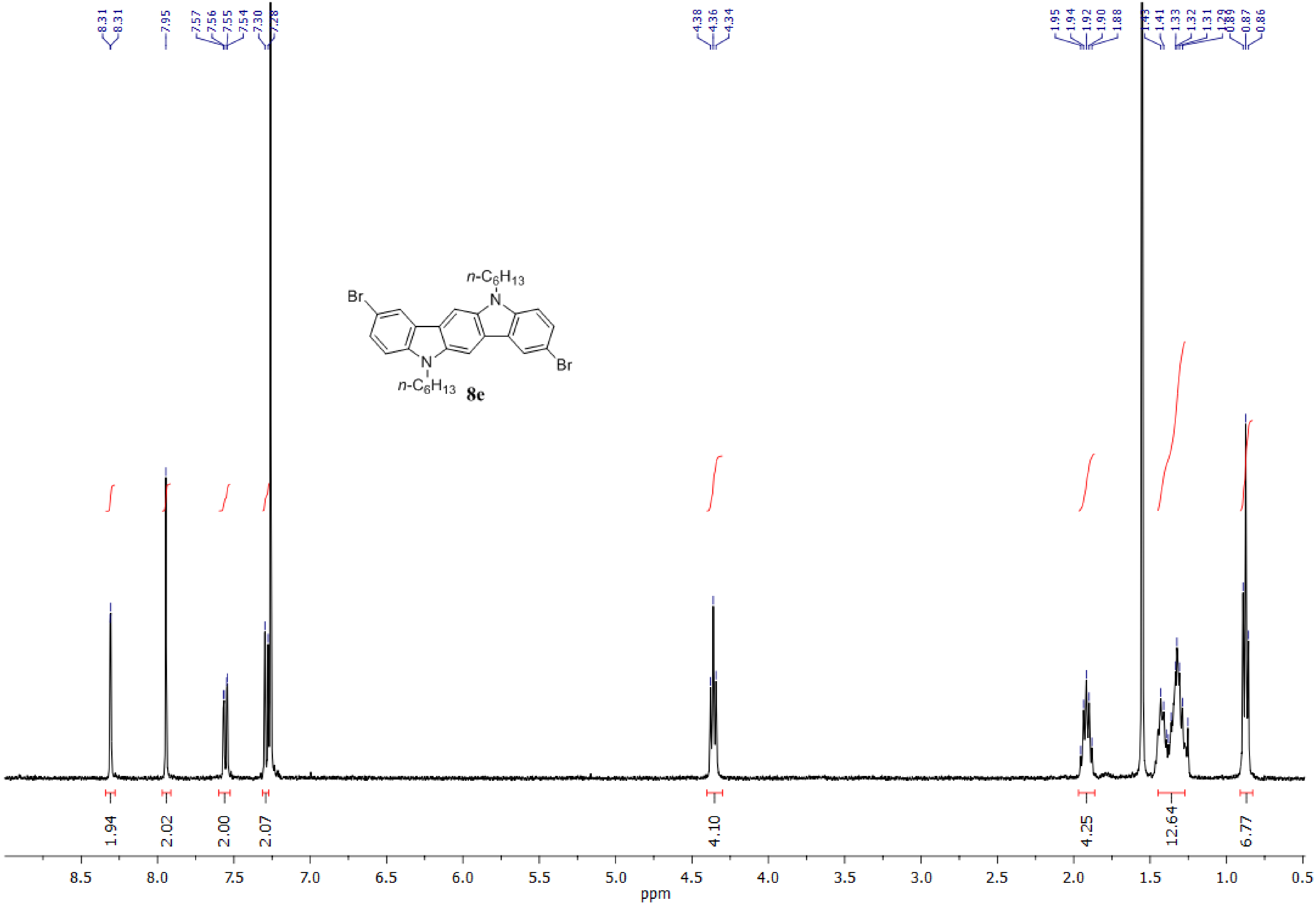

^{1}H NMR (solvent: $CDCl_3$)

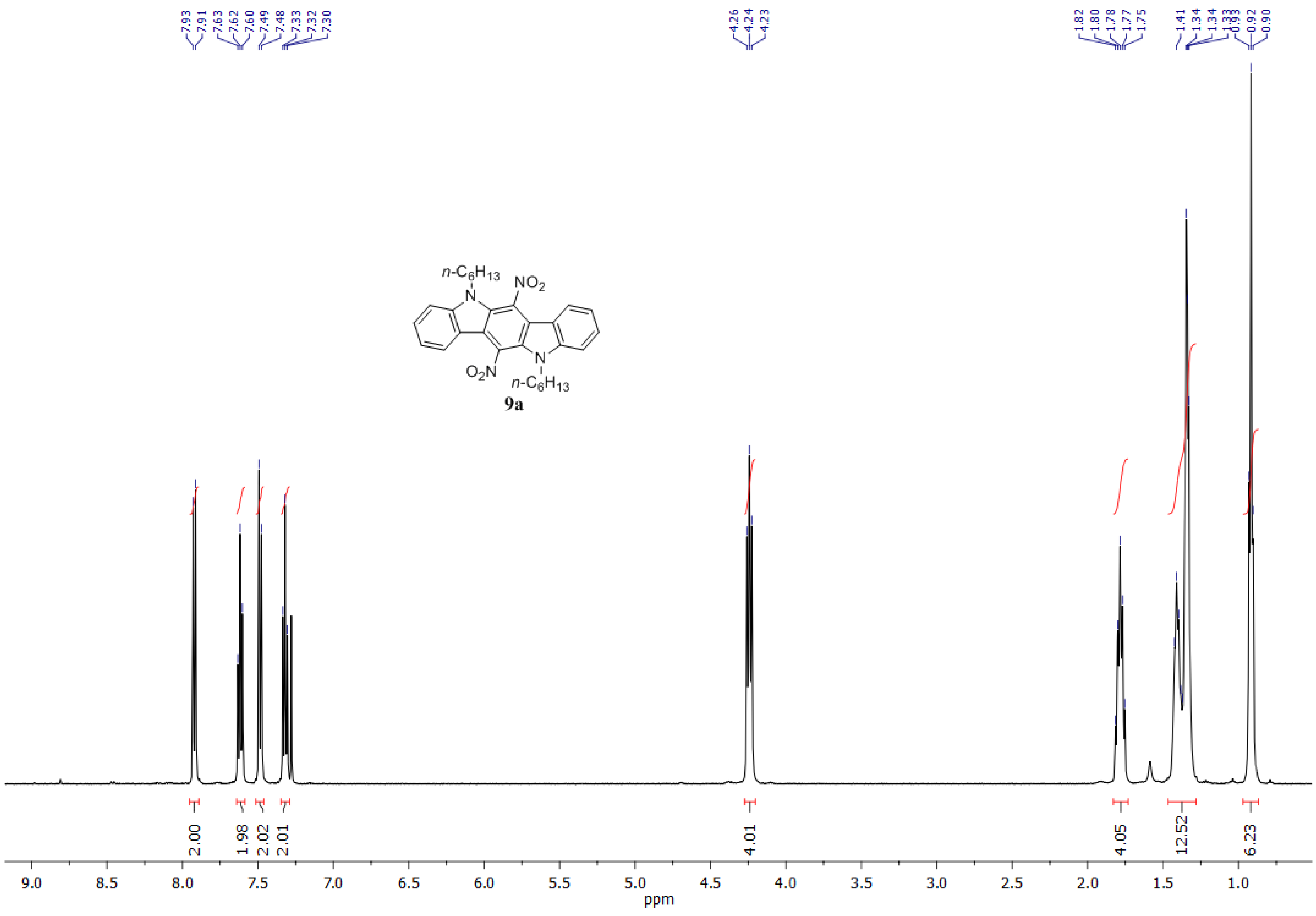

^{13}**C NMR** (solvent: $CDCl_3$)

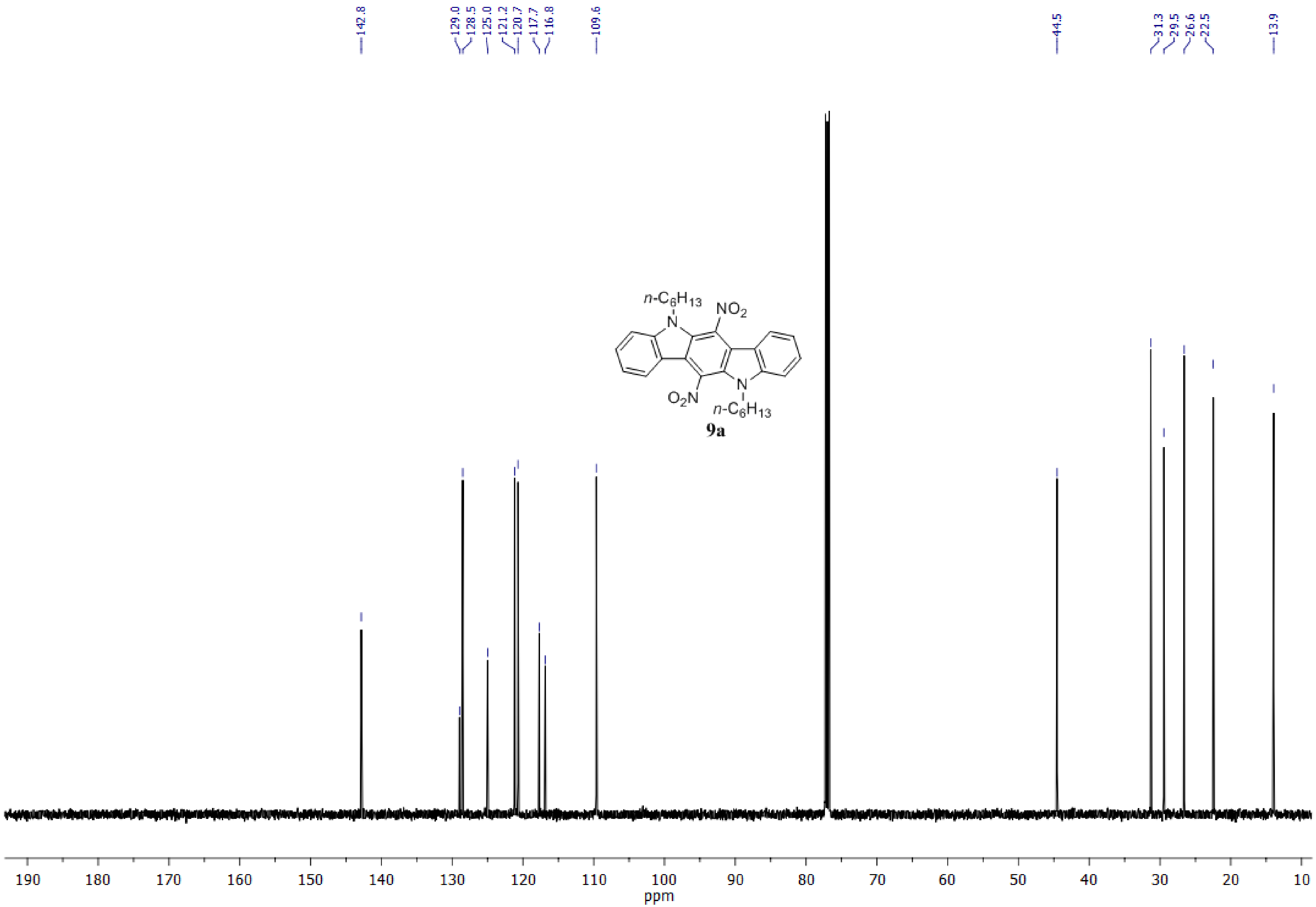

[1]**H NMR** (solvent: $CDCl_3$)

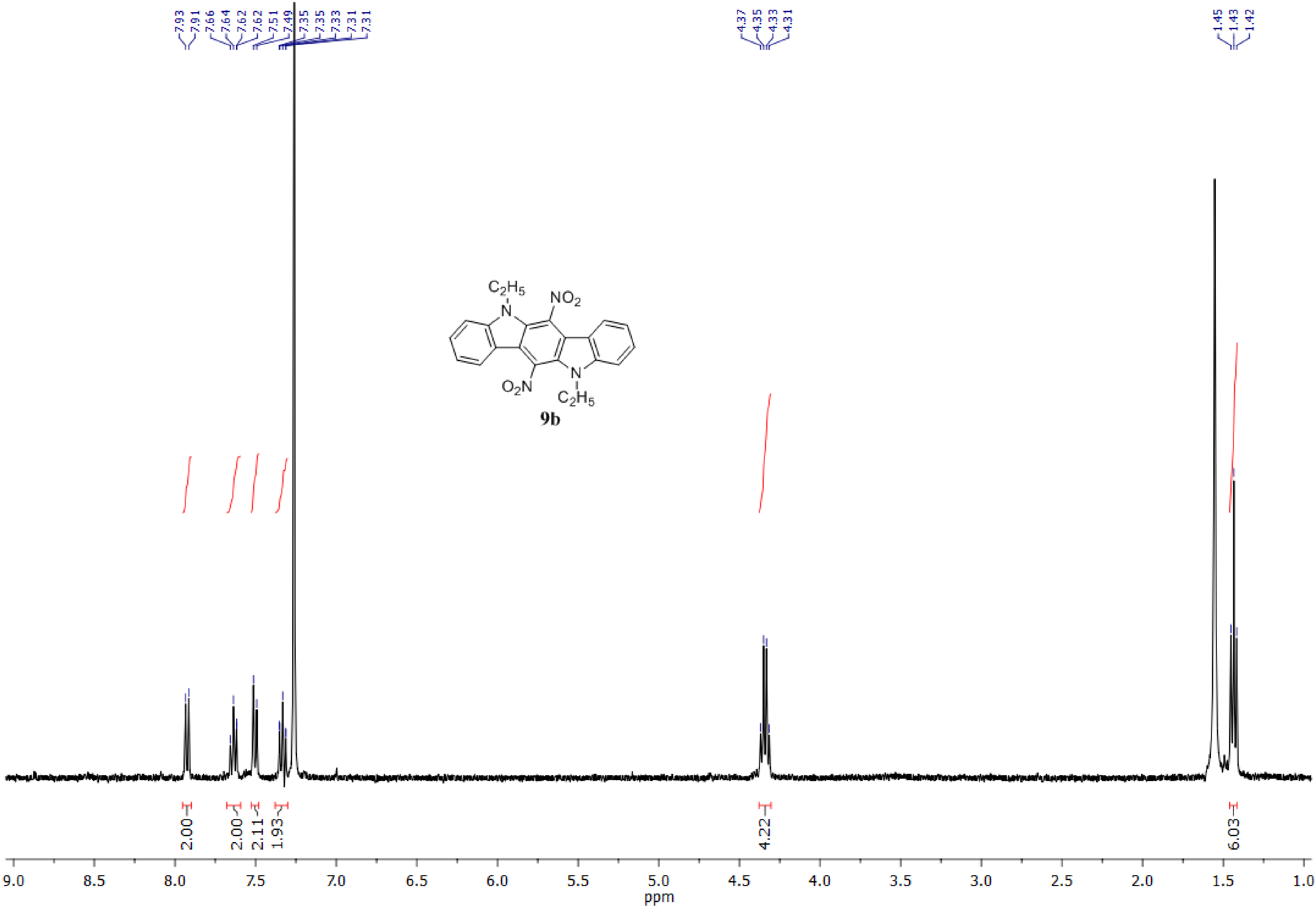

[1]**H NMR** (solvent: $CDCl_3$)

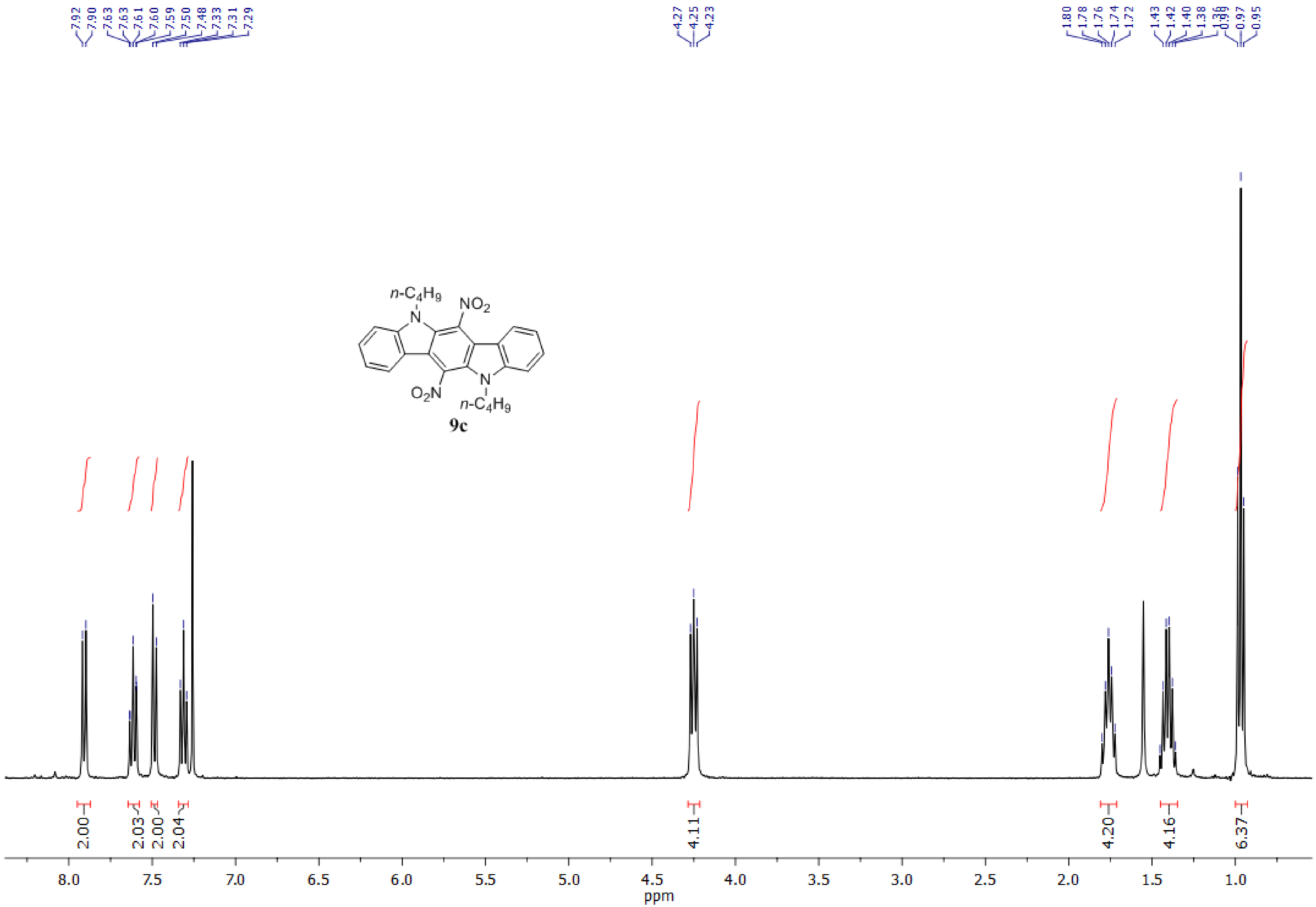

^{1}H NMR (solvent: $CDCl_3$)

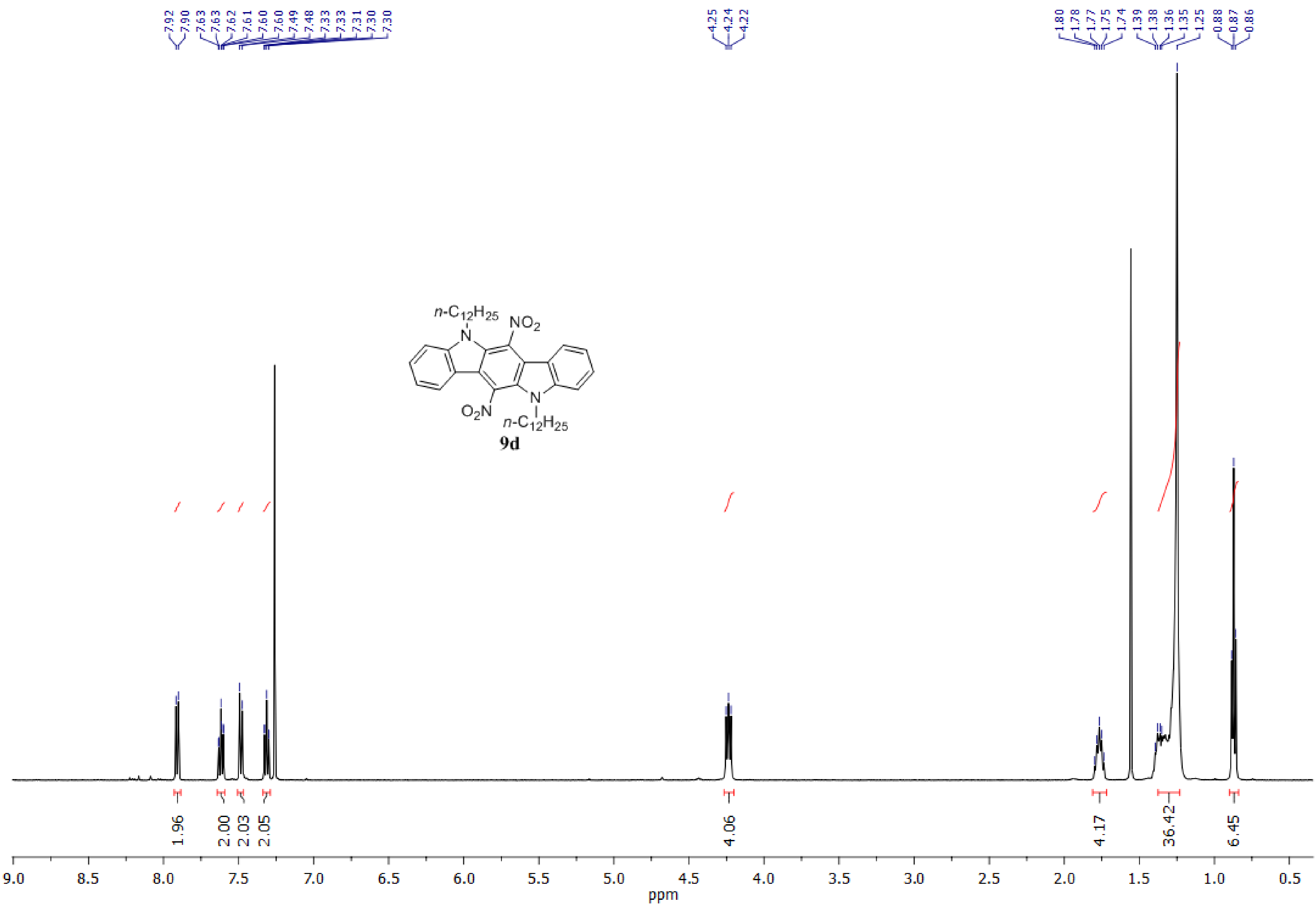

^{13}C NMR (solvent: $CDCl_3$)

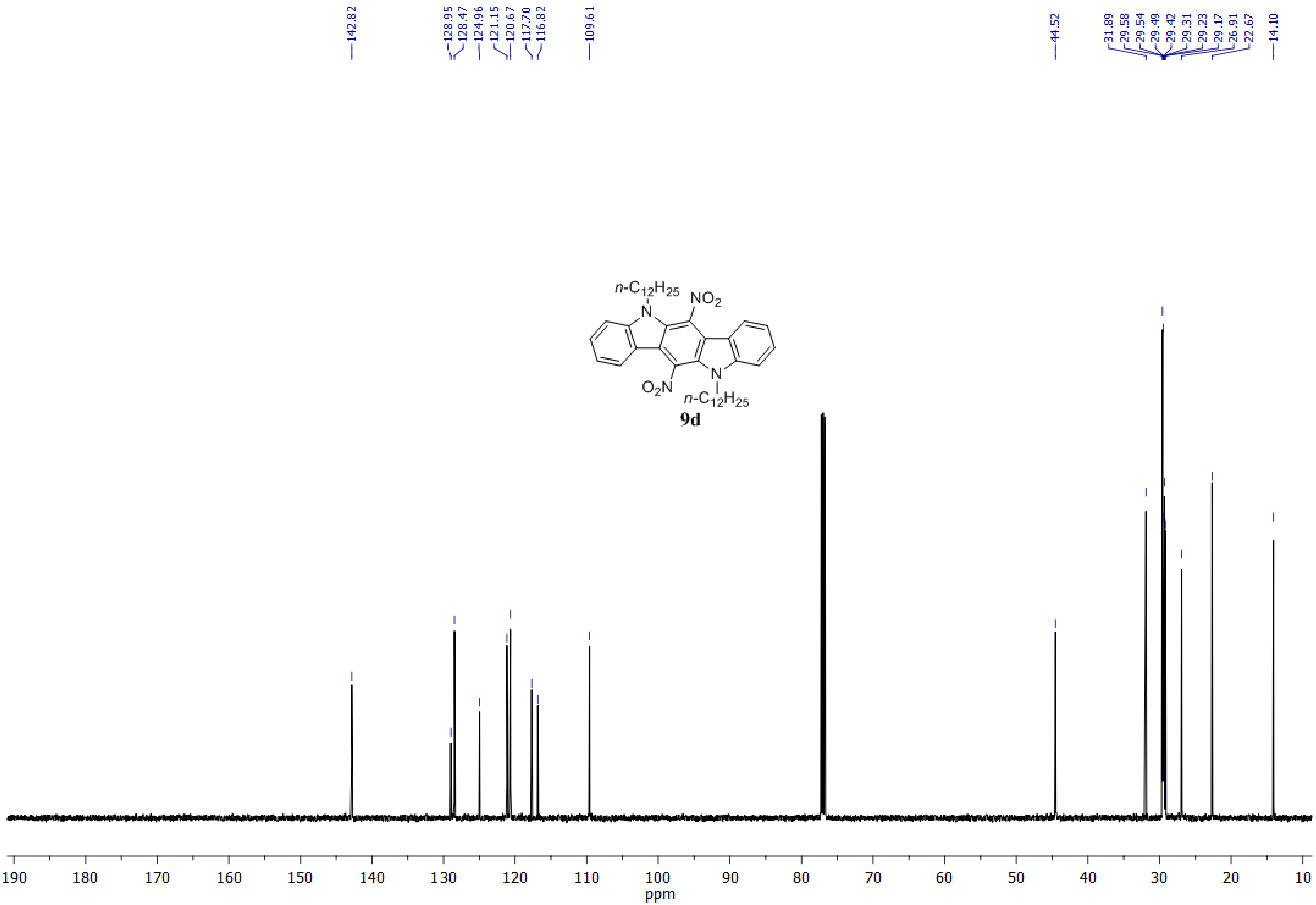

[1]**H NMR** (solvent: $CDCl_3$)

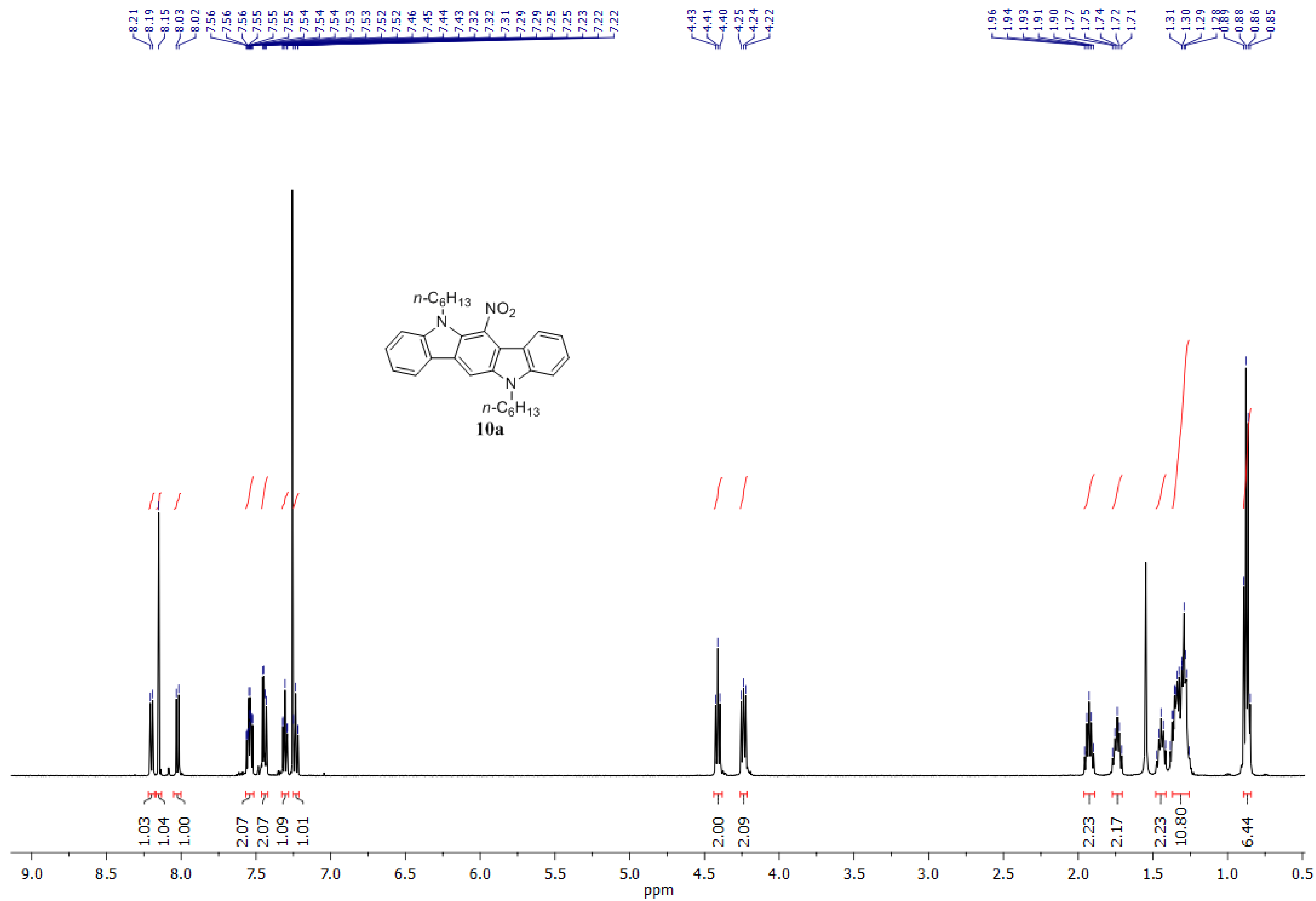

^{13}C NMR (solvent: $CDCl_3$)

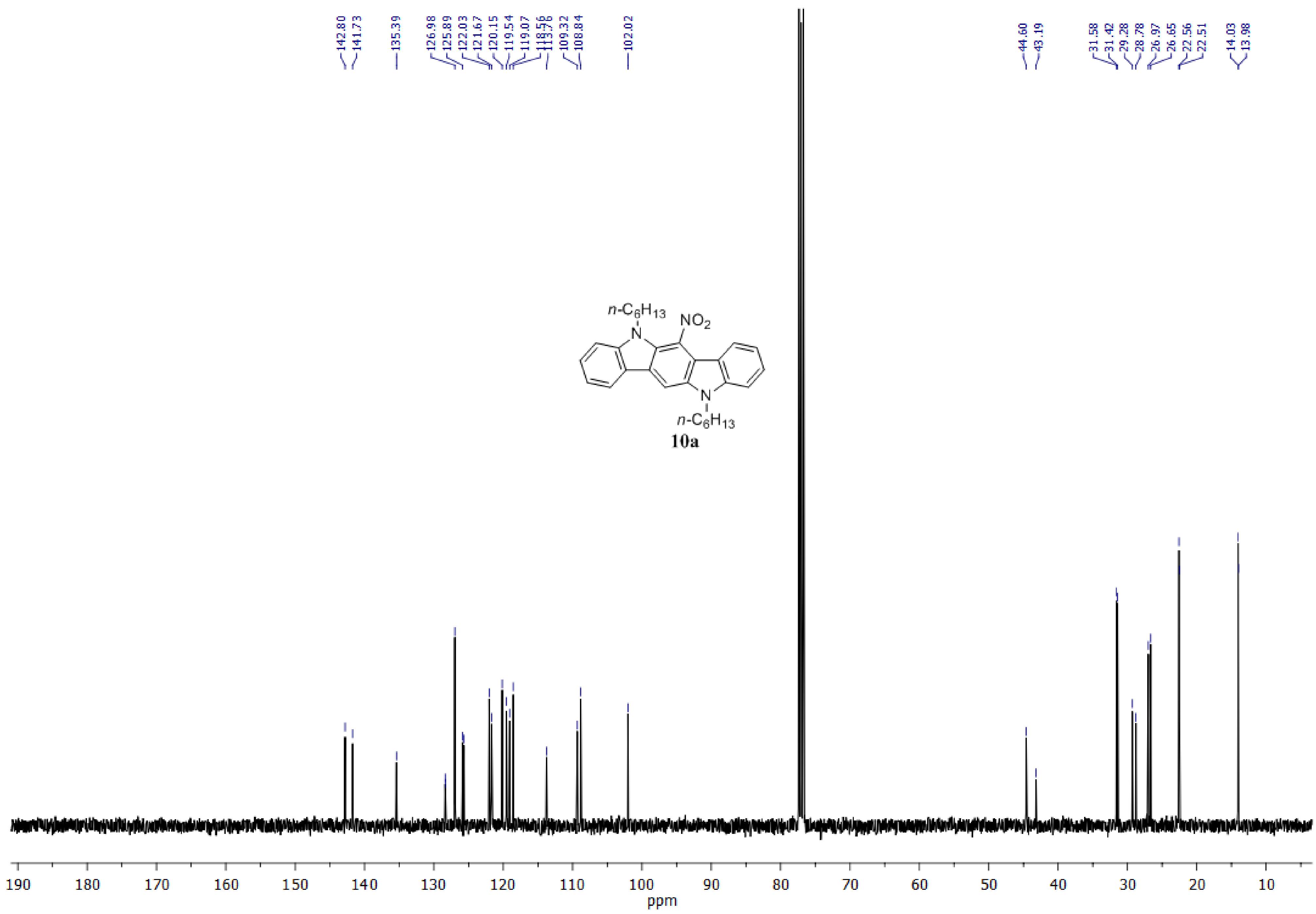

^{1}**H NMR** (solvent: DMSO-d_6)

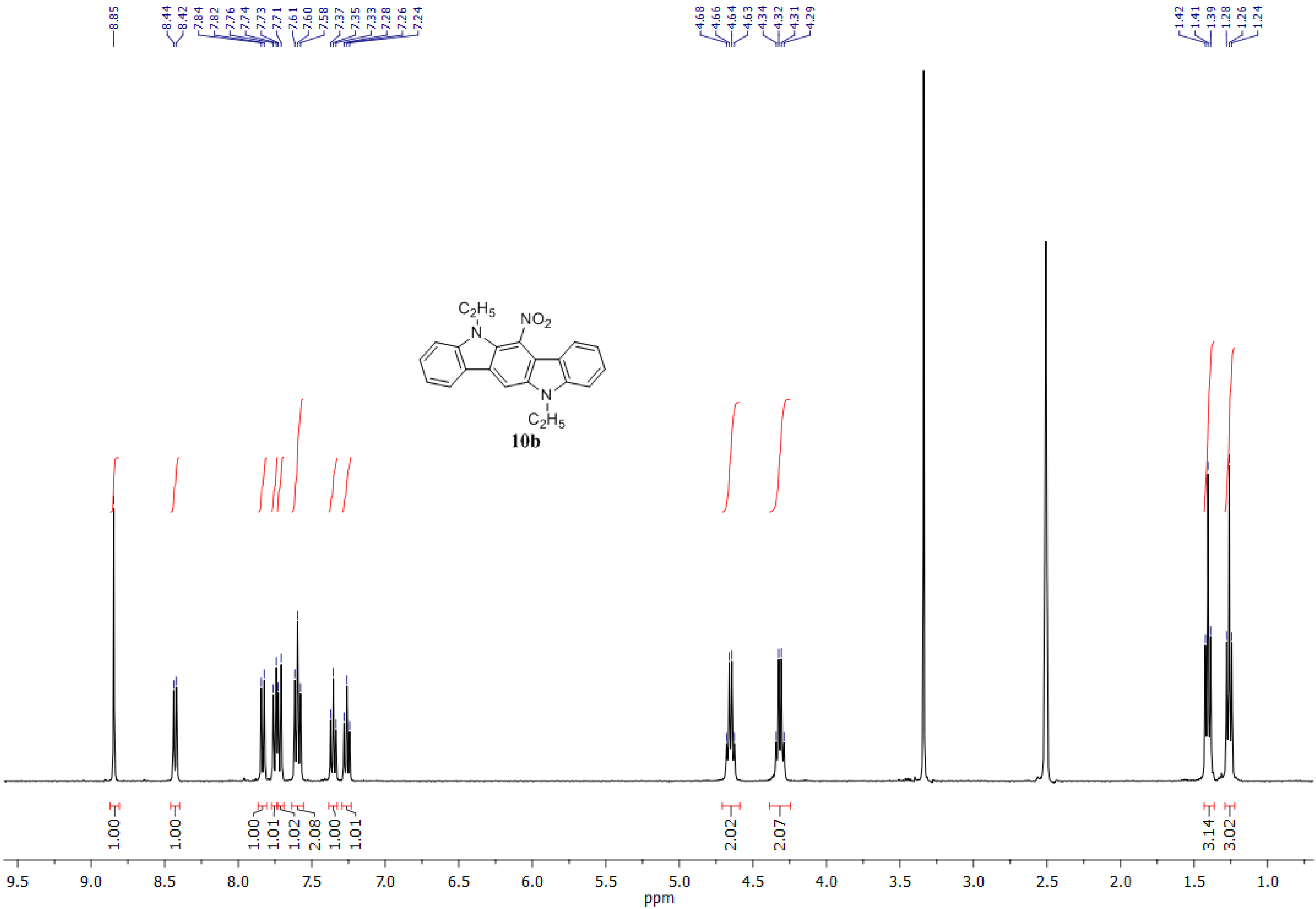

^{13}C NMR (solvent: $CDCl_3$)

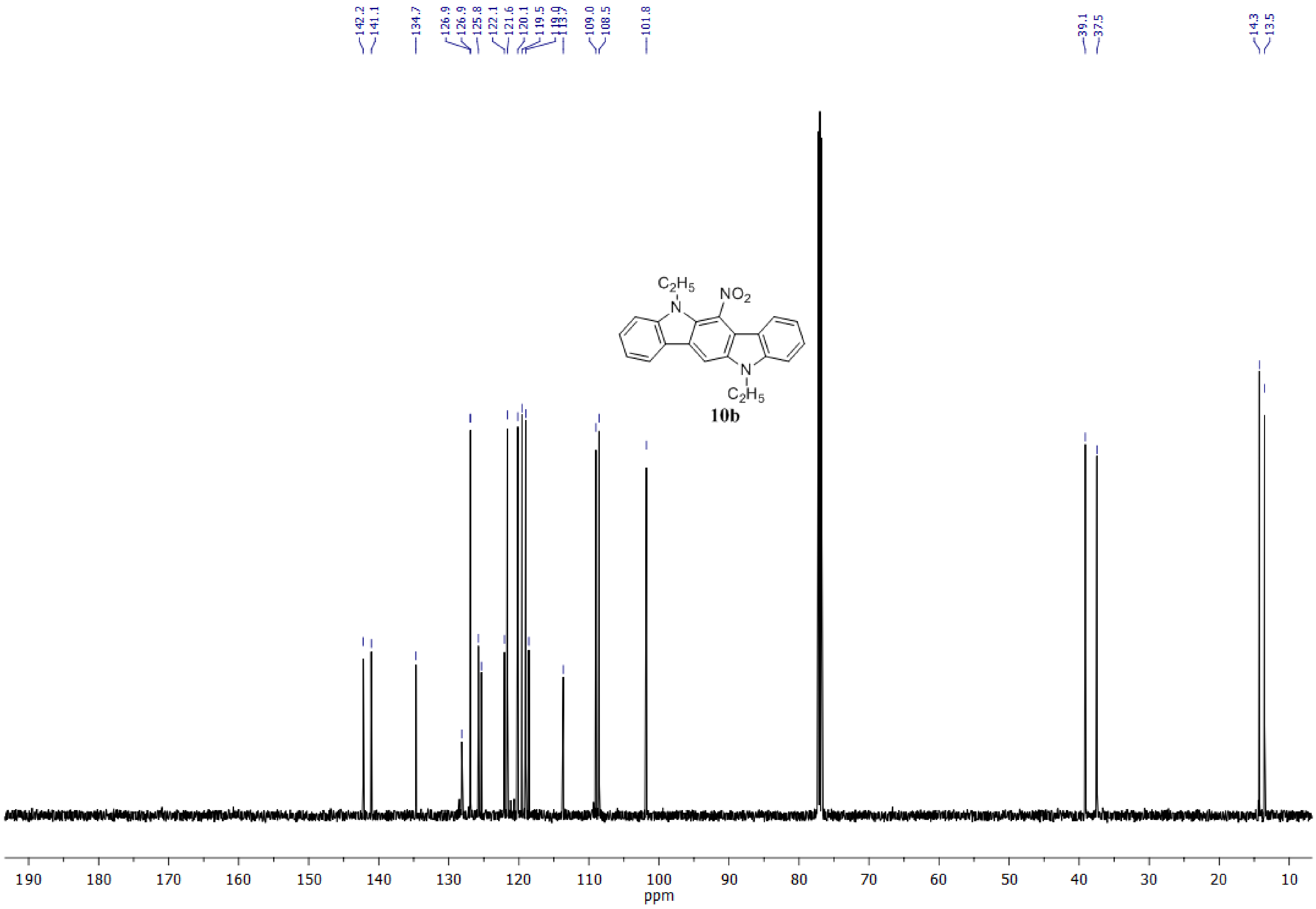

^{1}**H NMR** (solvent: $CDCl_3$)

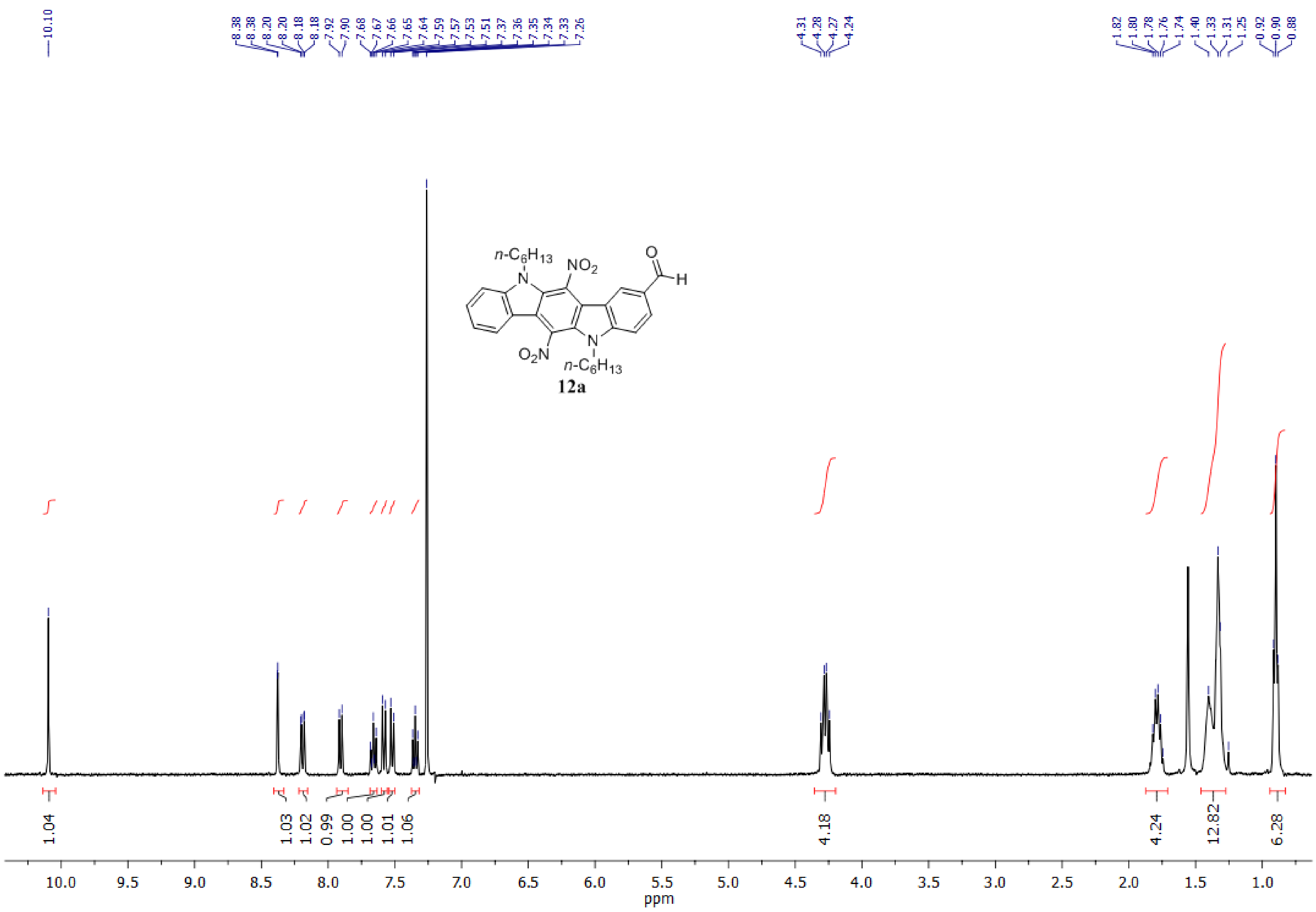

^{13}**C NMR** (solvent: $CDCl_3$)

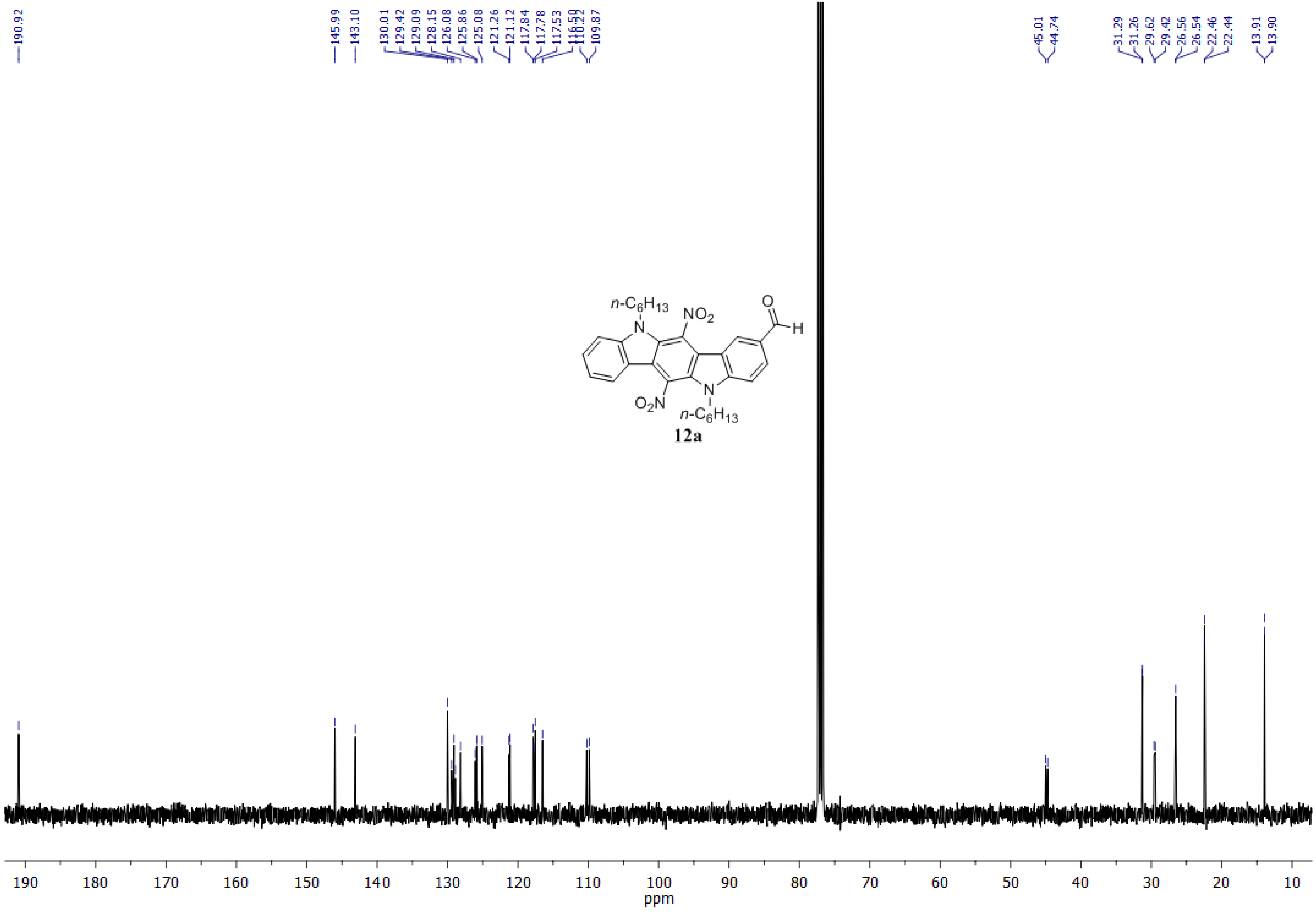

^{1}H NMR (solvent: $CDCl_3$)

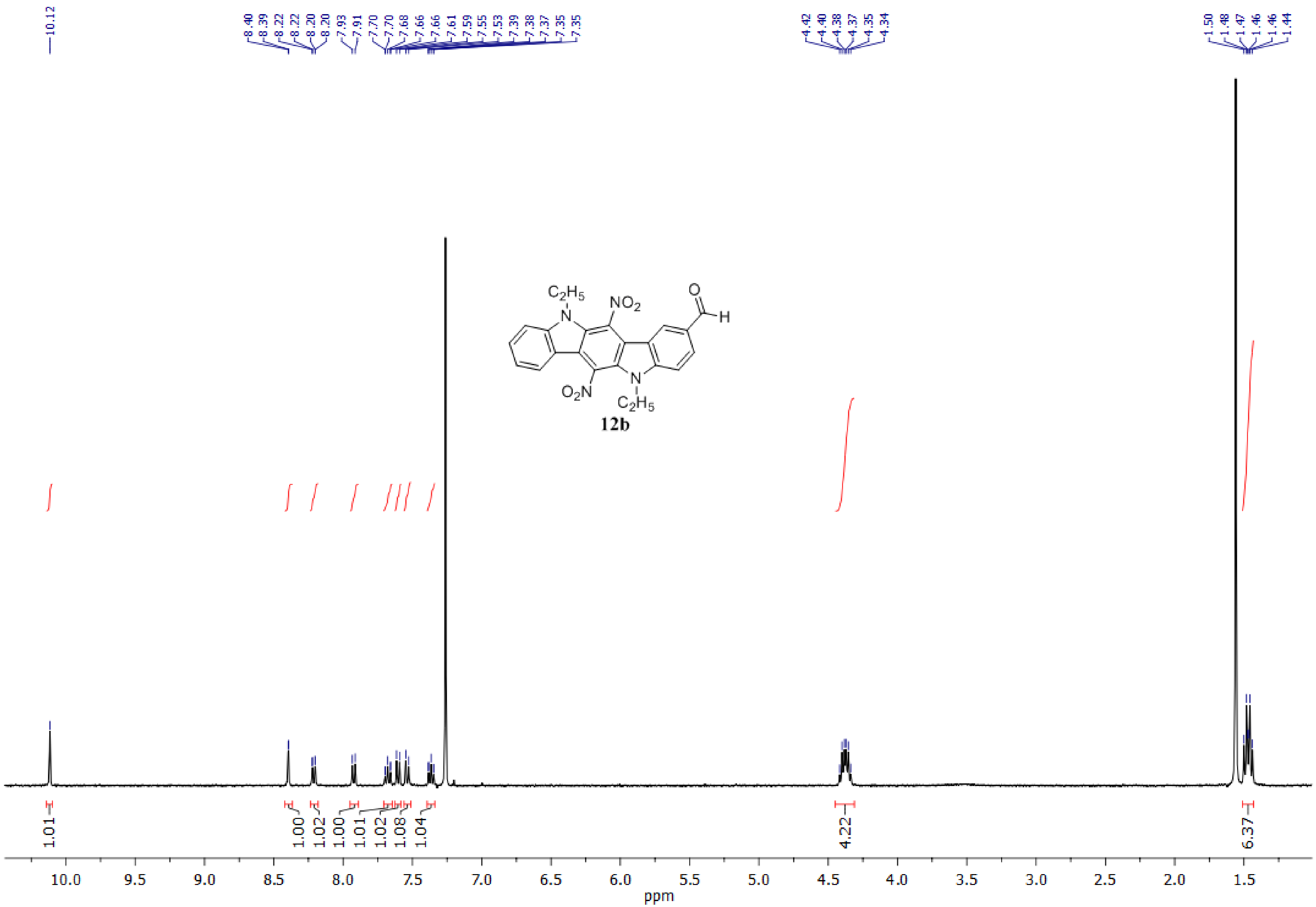

^{1}H NMR (solvent: $CDCl_3$)

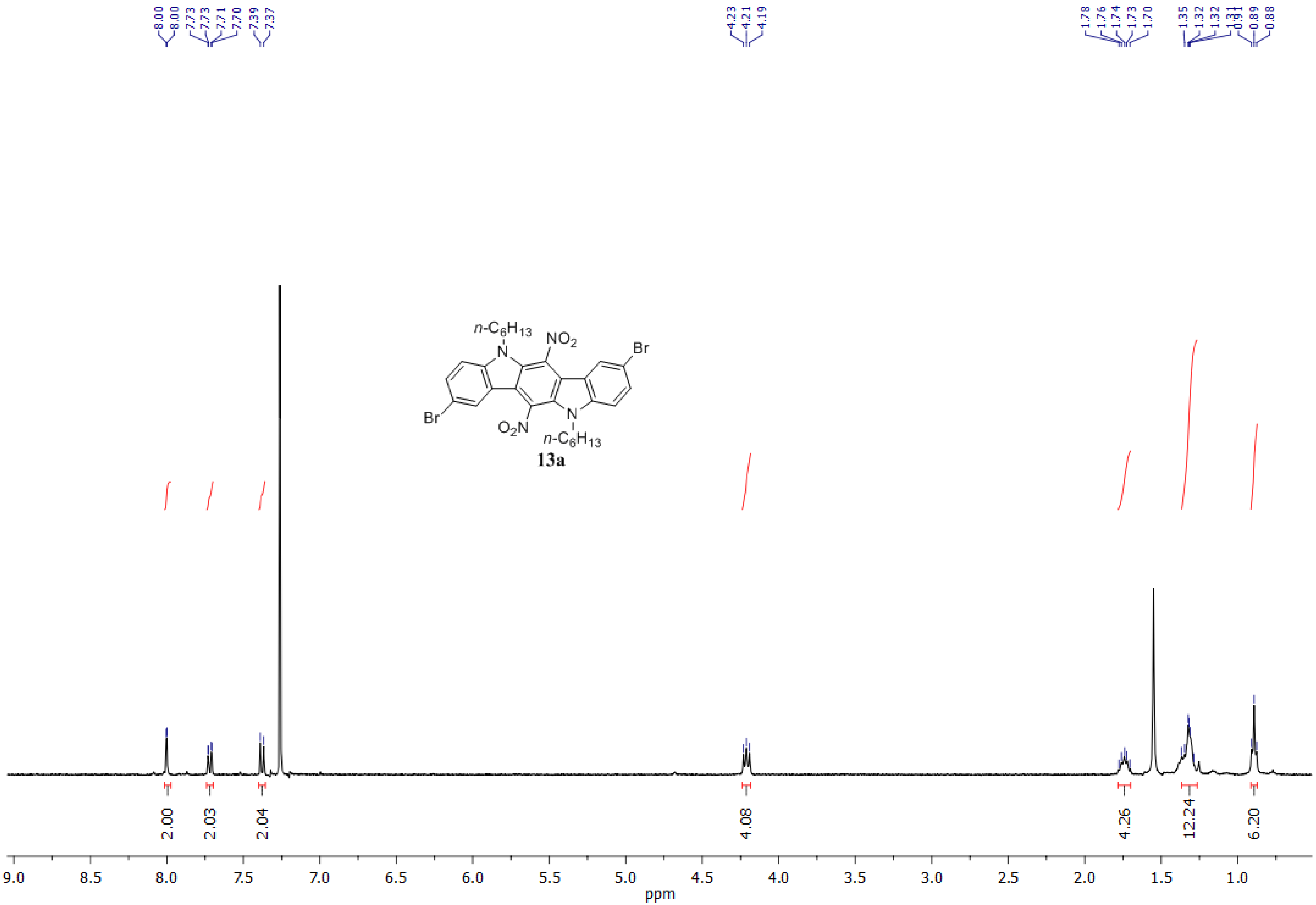

[1]**H NMR** (solvent: $CDCl_3$)

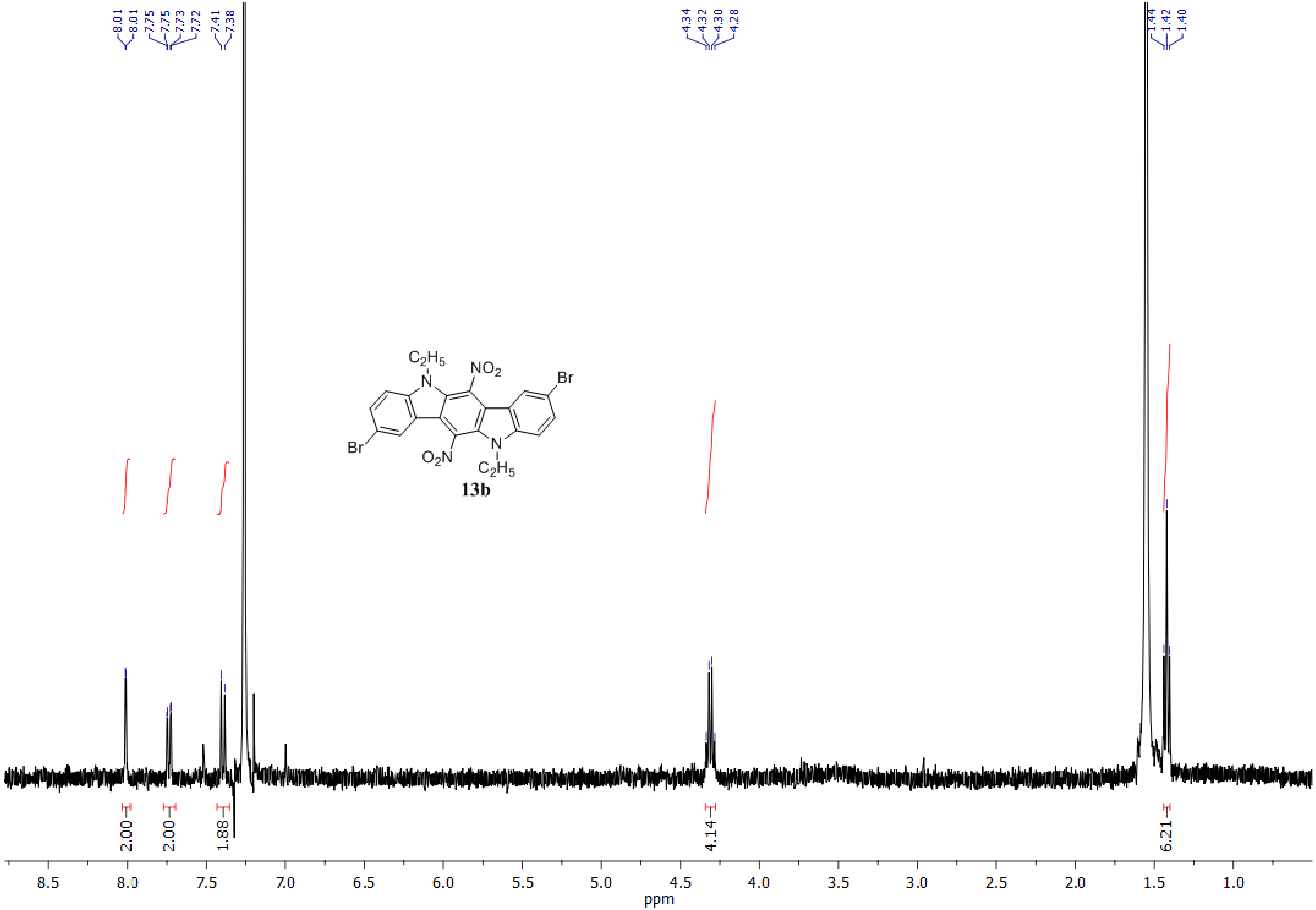

^{1}**H NMR** (solvent: $CDCl_3$)

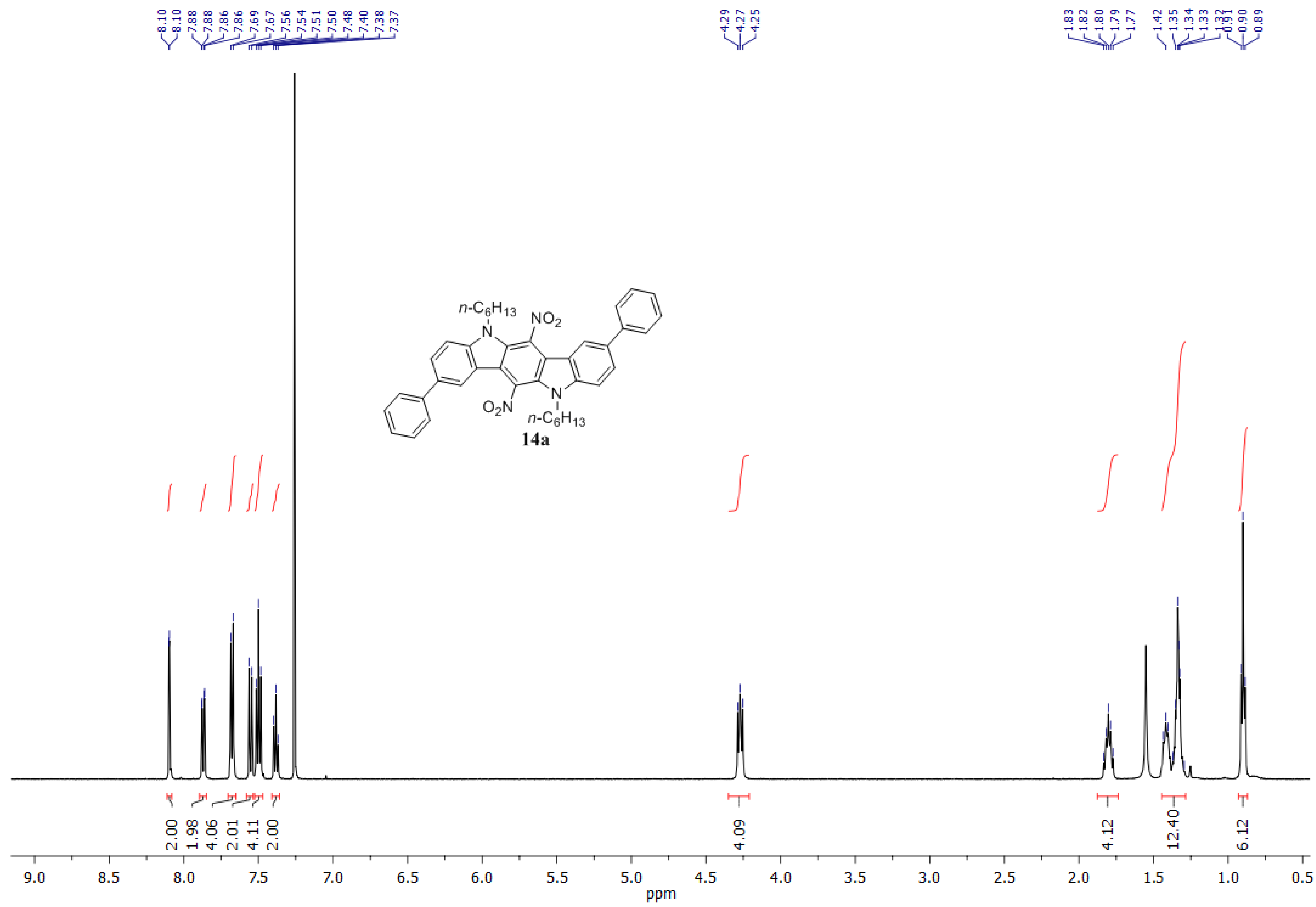

^{13}**C NMR** (solvent: $CDCl_3$)

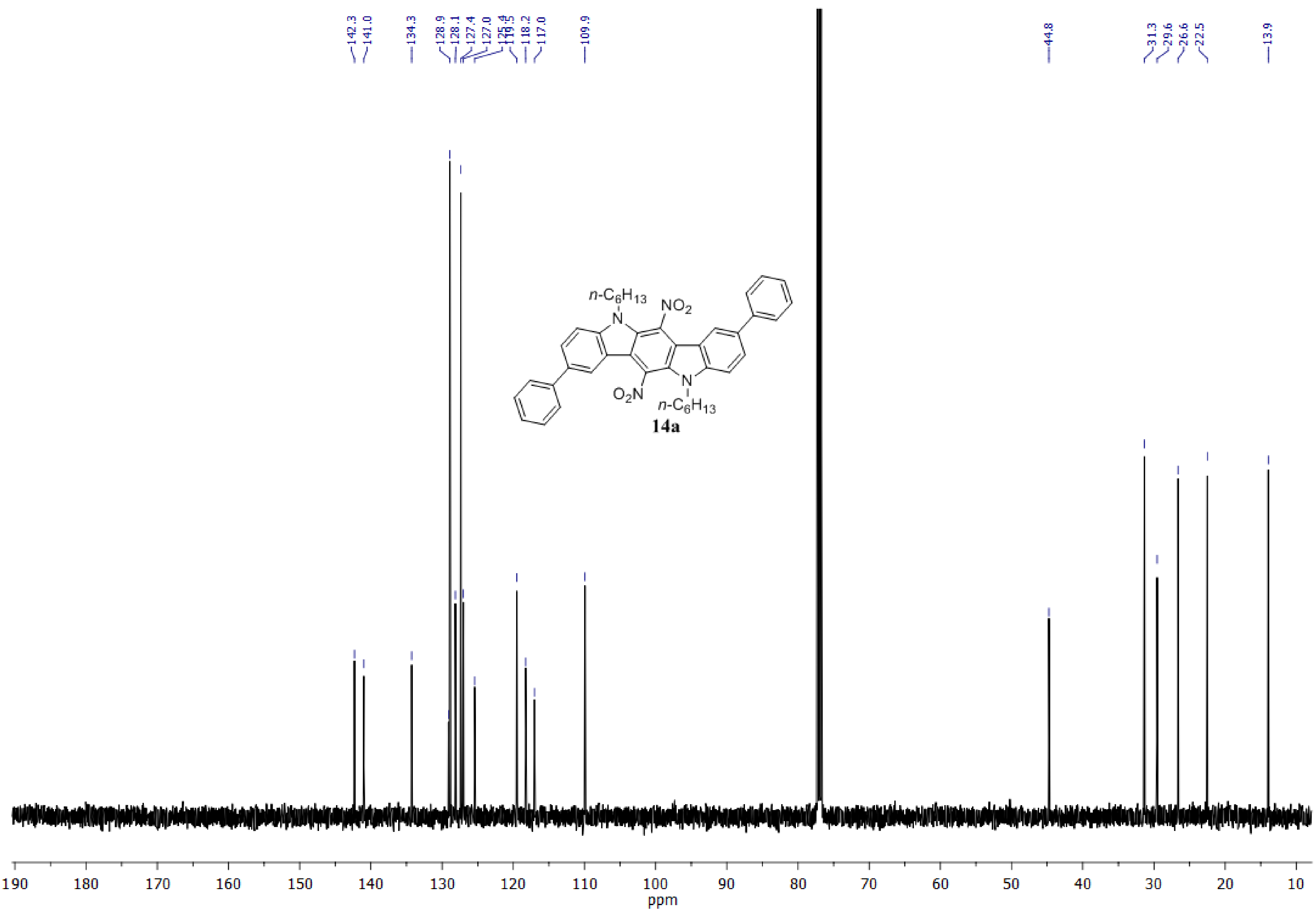

^{1}**H NMR** (solvent: C_6D_6)

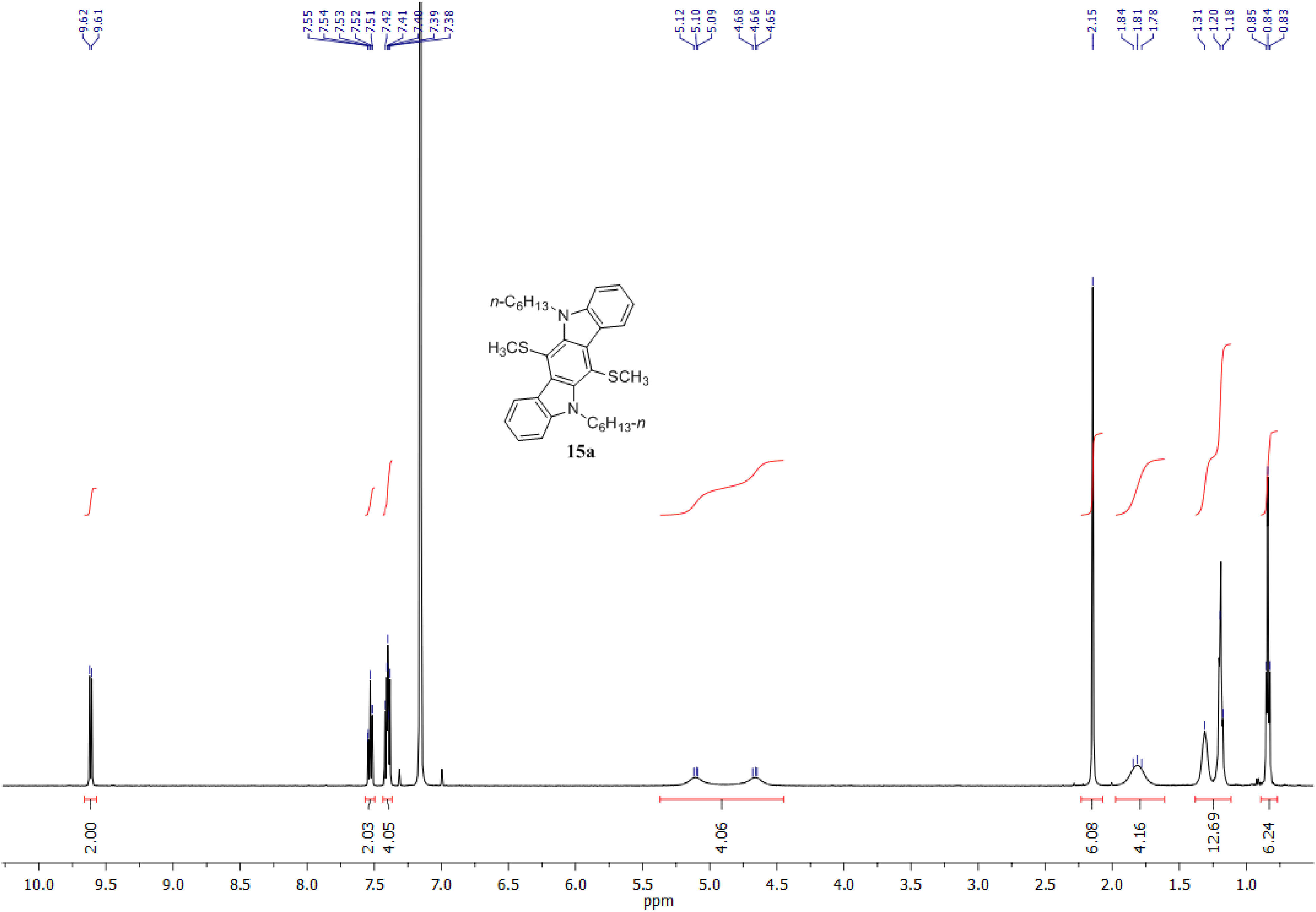

^{13}**C NMR** (solvent: $CDCl_3$)

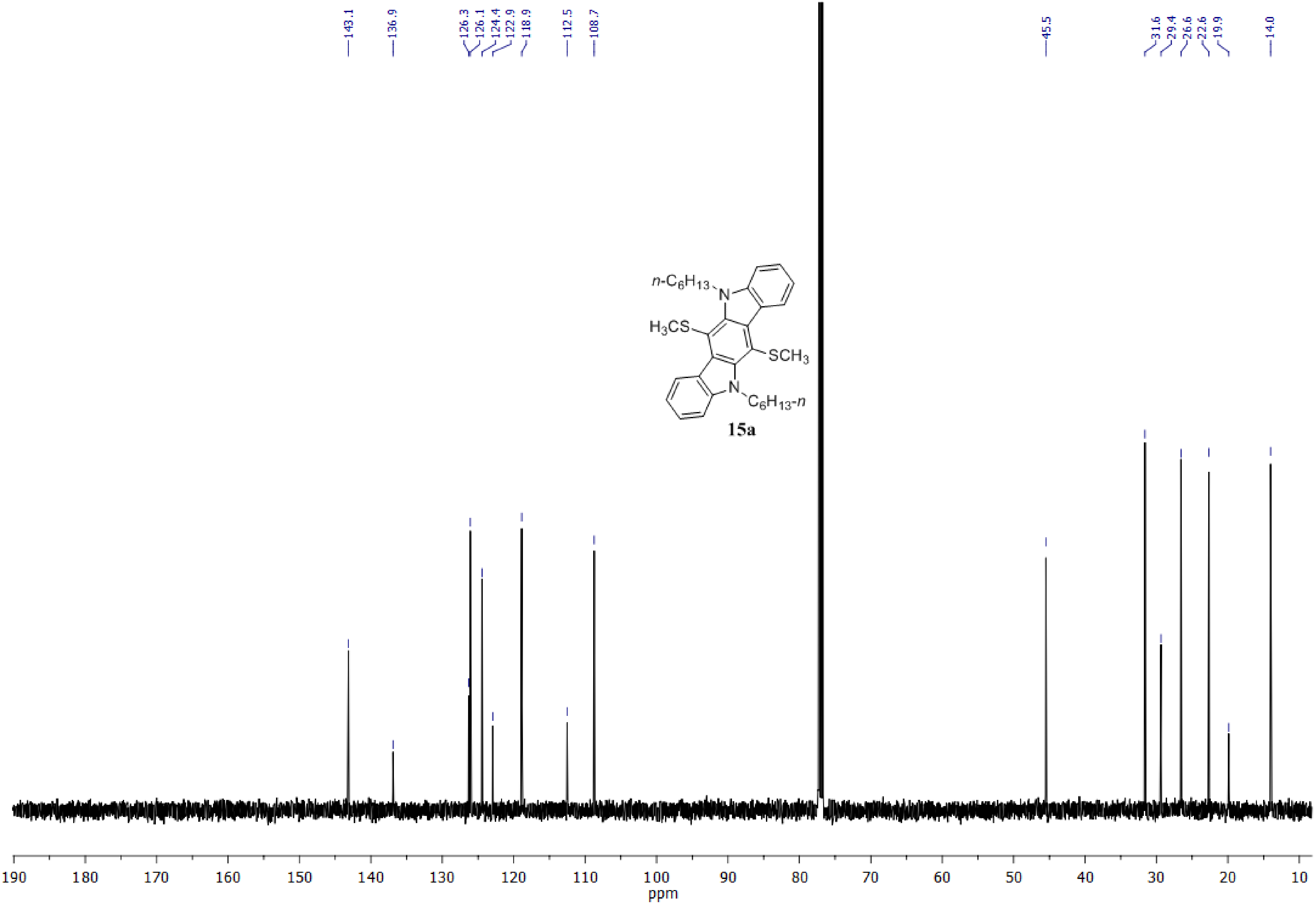

15a

[1]**H NMR** (solvent: C_6D_6)

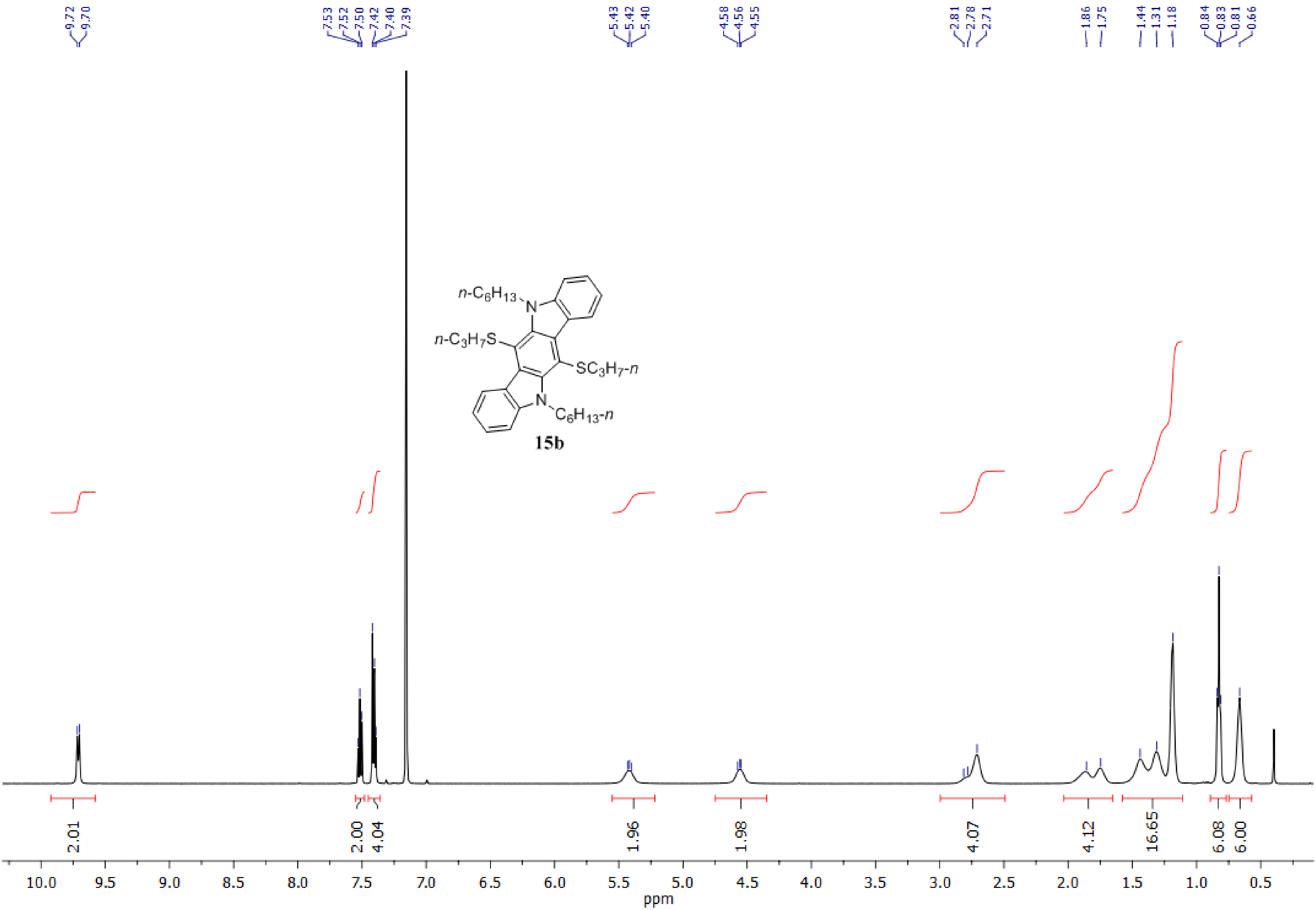

^{13}**C NMR** (solvent: $CDCl_3$)

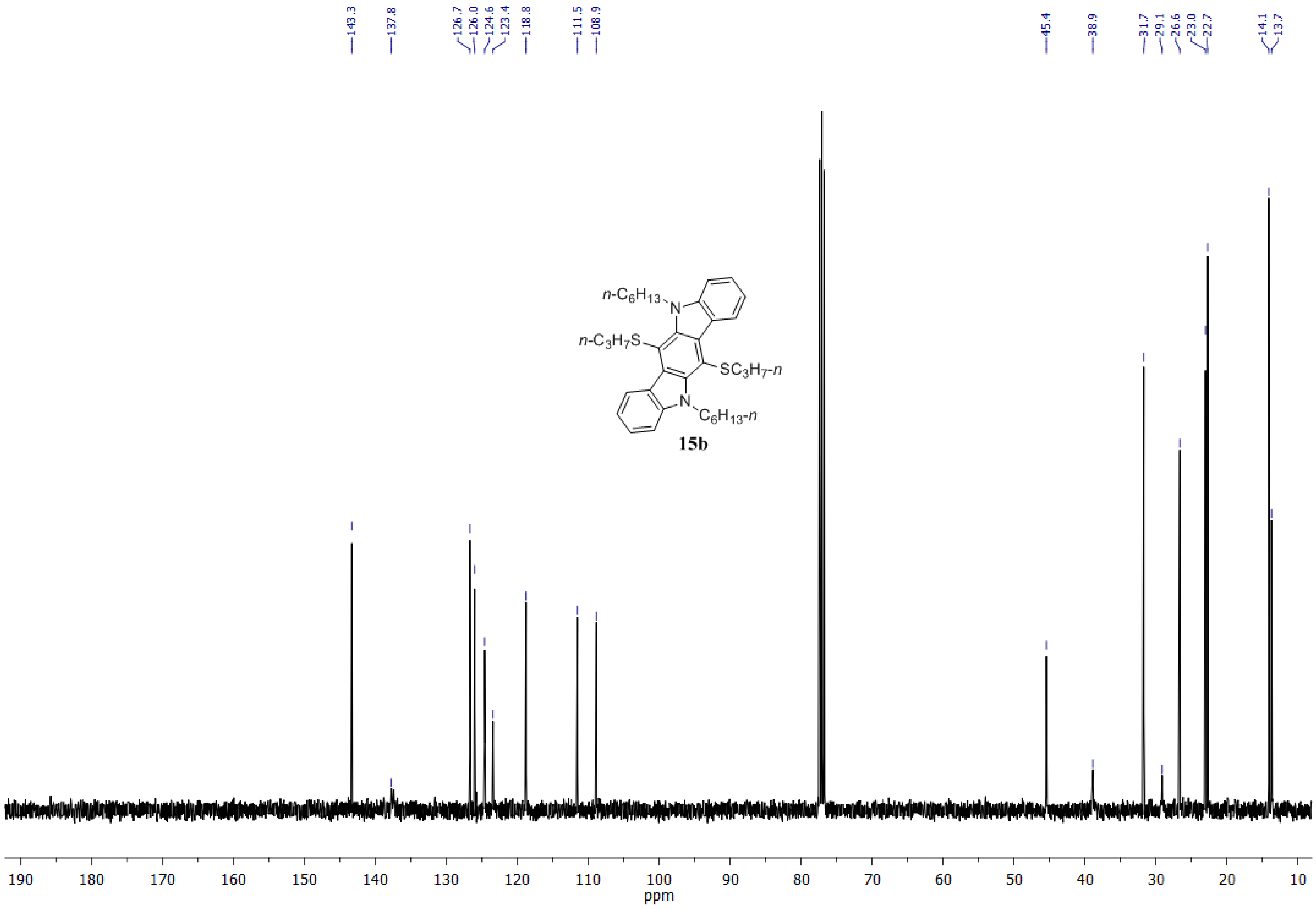

^{1}**H NMR** (solvent: $CDCl_3$)

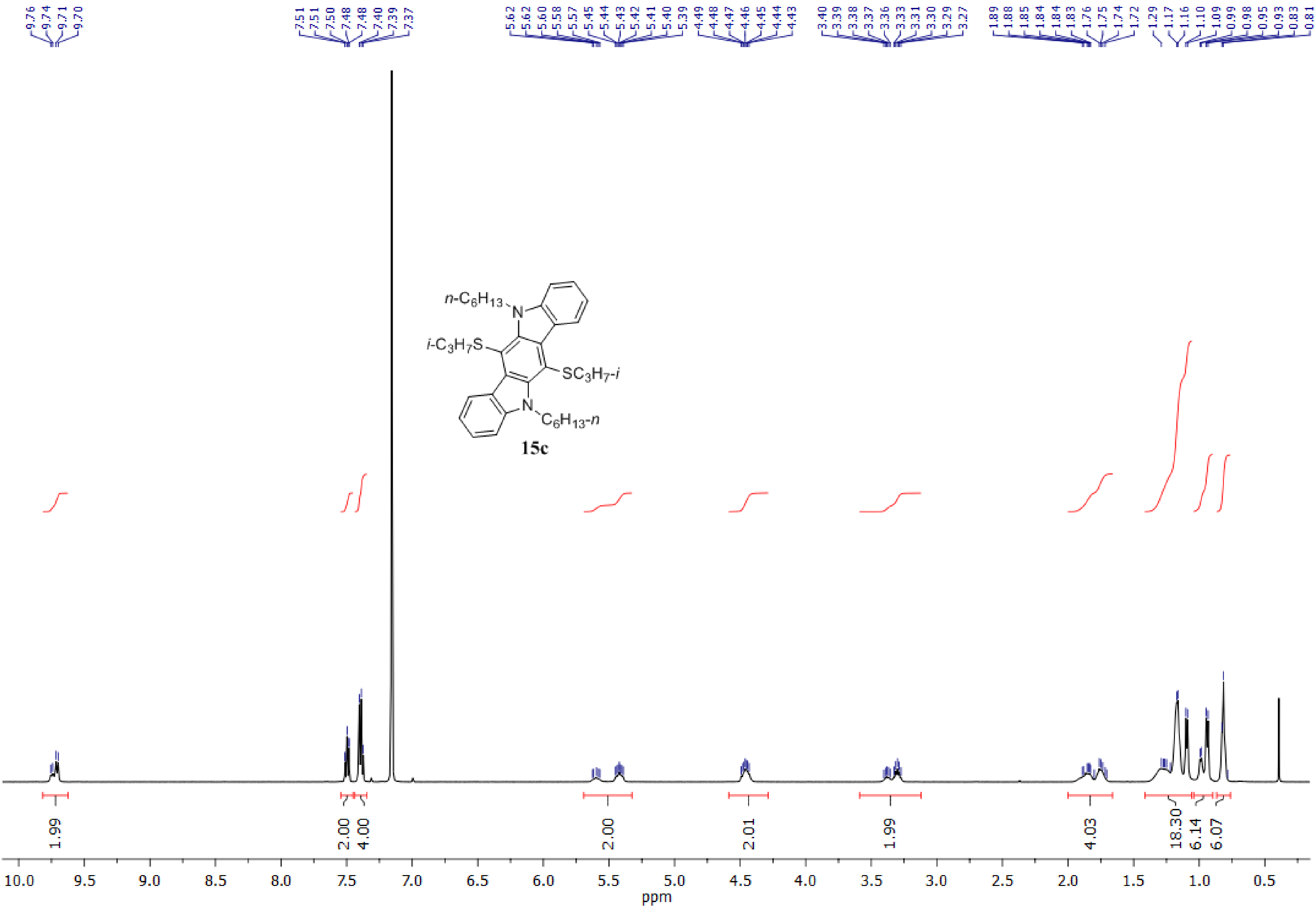

^{13}C NMR (solvent: $CDCl_3$)

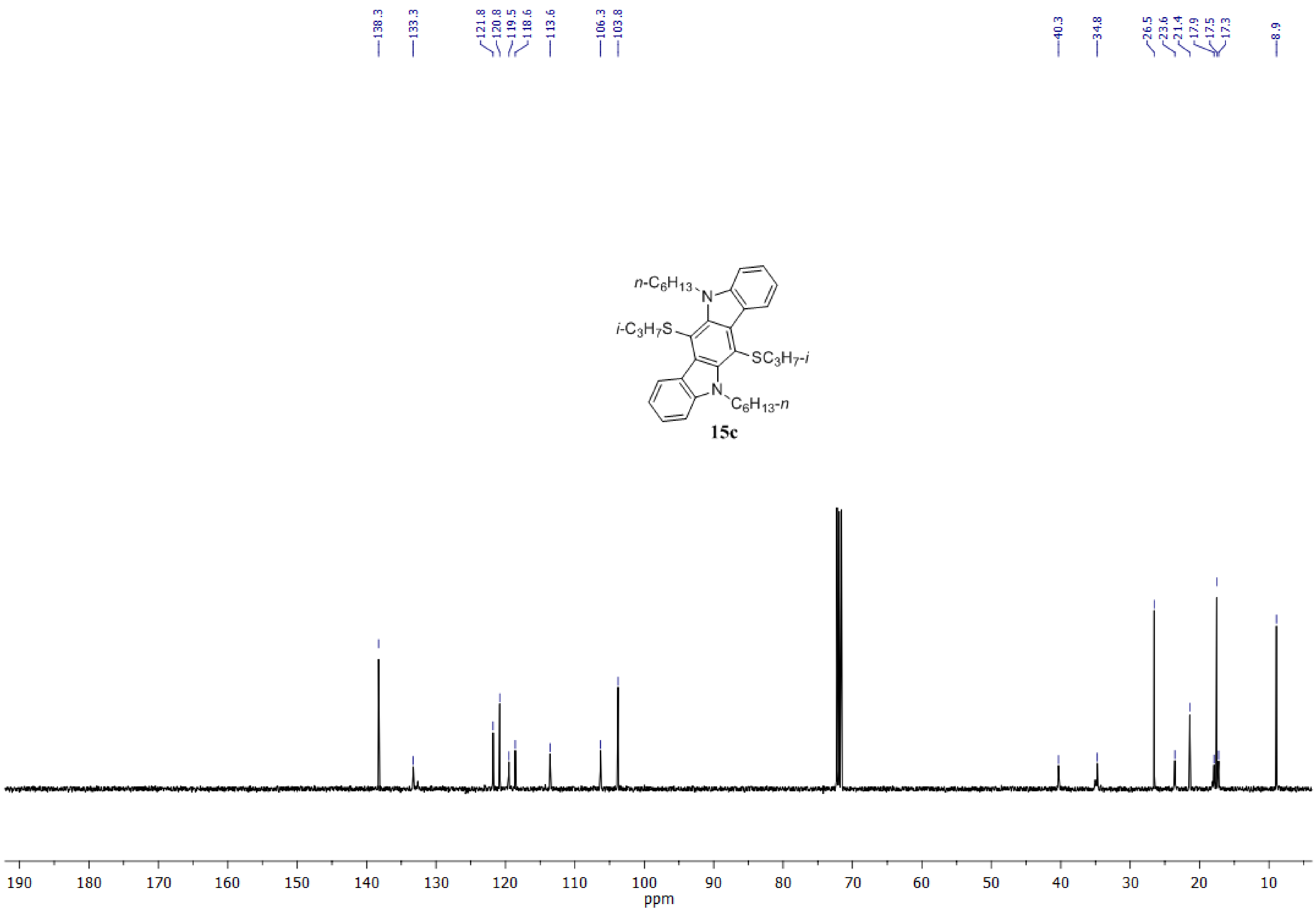

^{1}H NMR (solvent: C_6D_6)

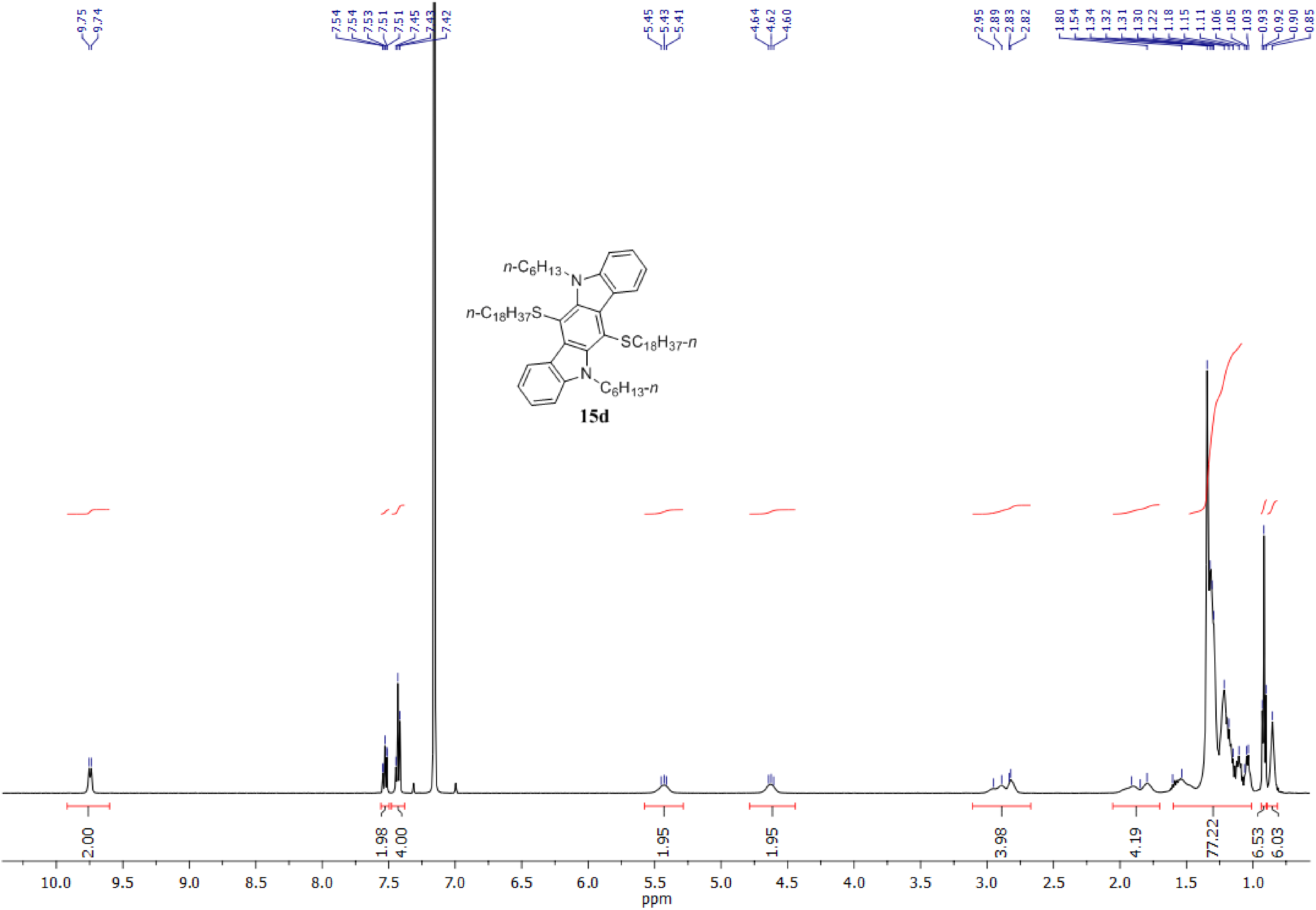

^{13}C NMR (solvent: $CDCl_3$)

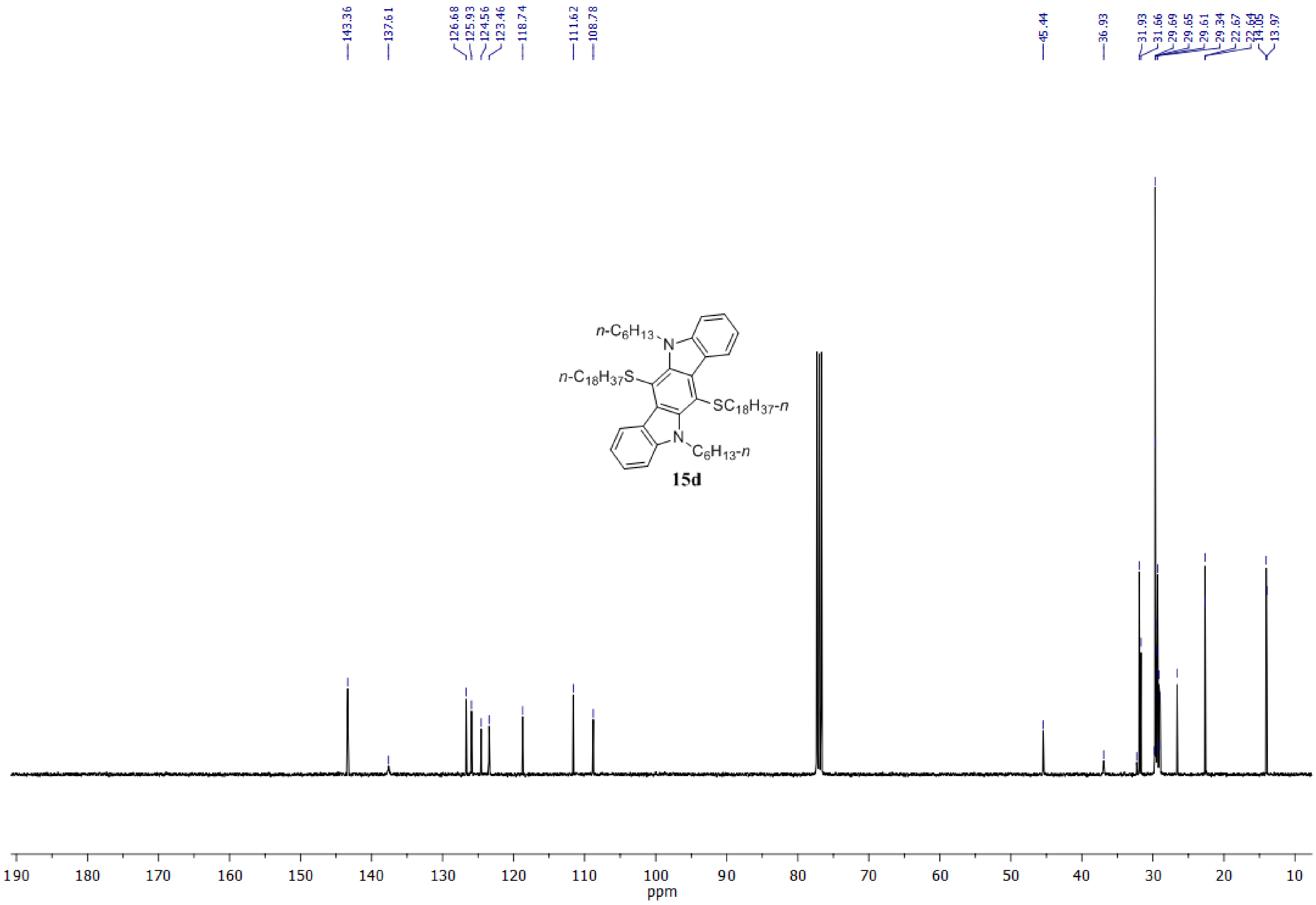

[1]**H NMR** (solvent: $CDCl_3$)

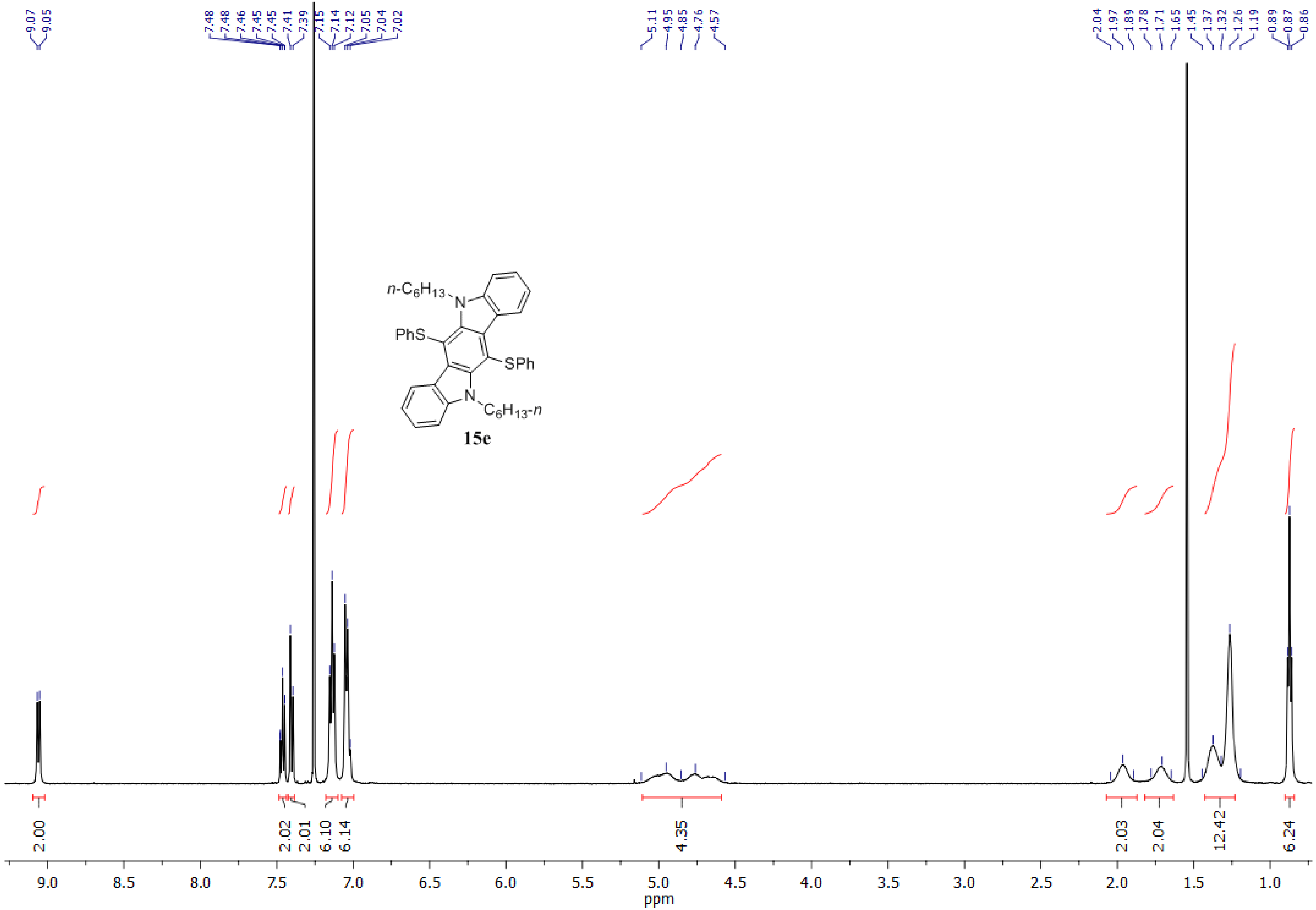

^{13}C NMR (solvent: $CDCl_3$)

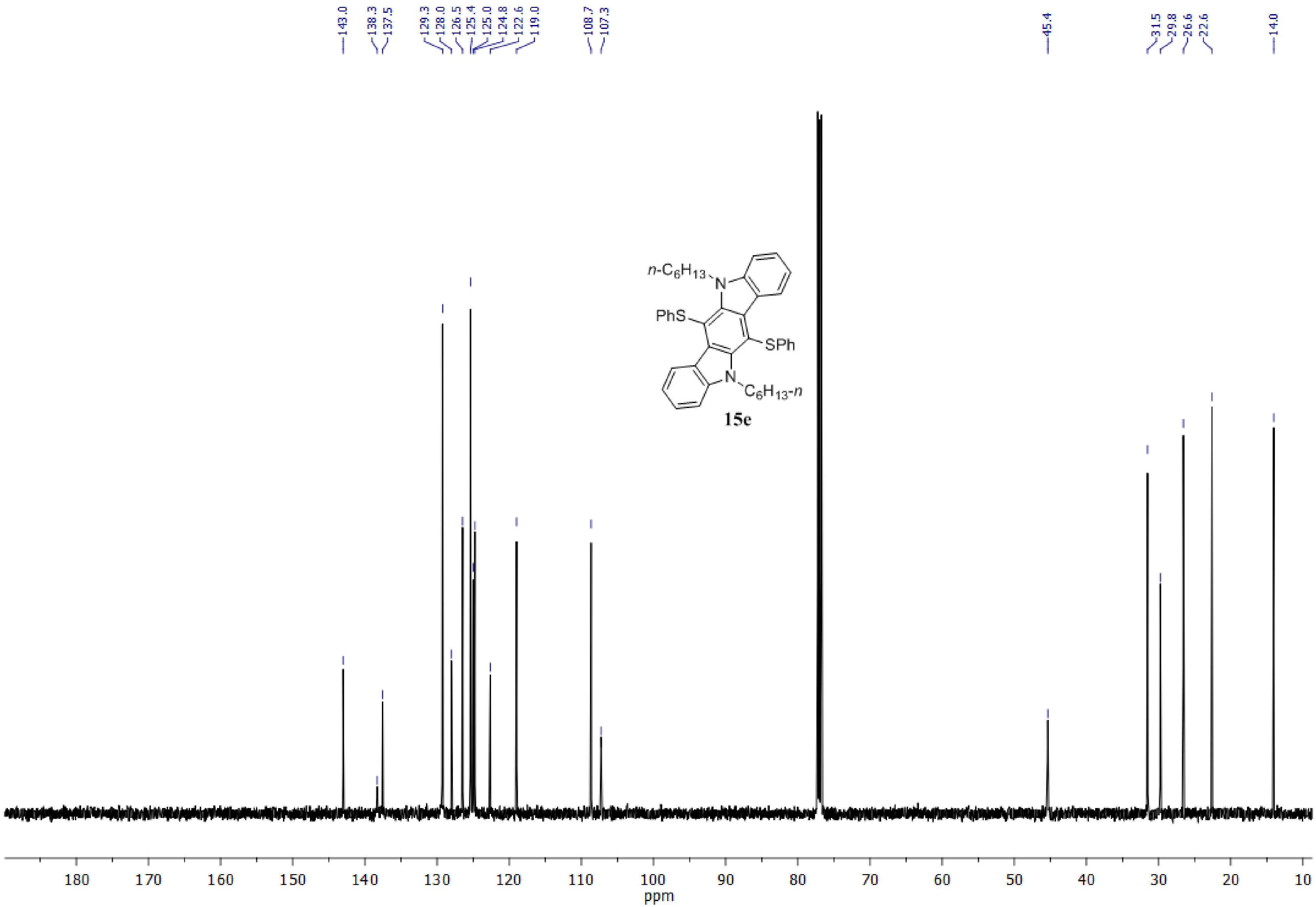

^{1}**H NMR** (solvent: $CDCl_3$)

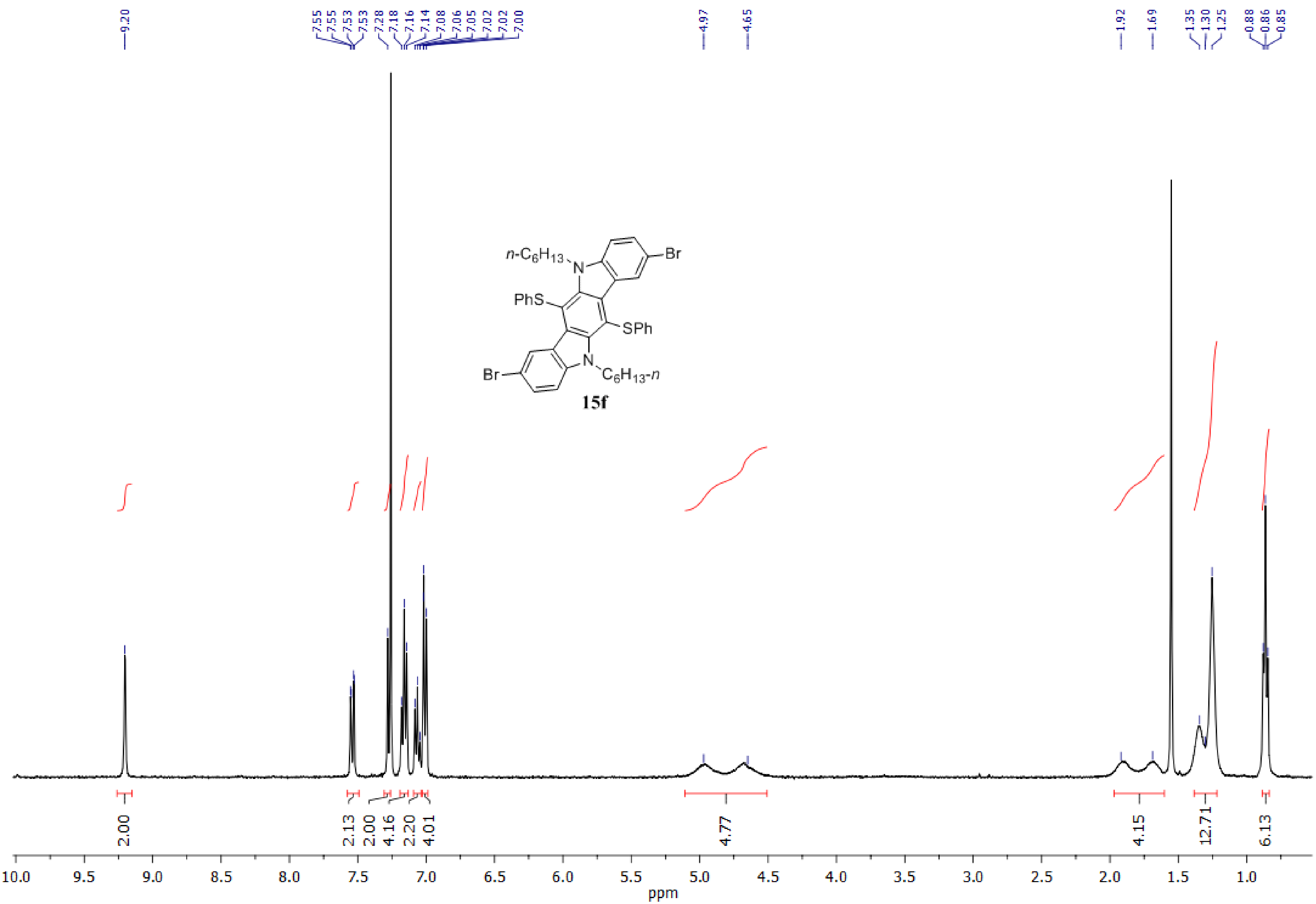

^{13}C NMR (solvent: $CDCl_3$)

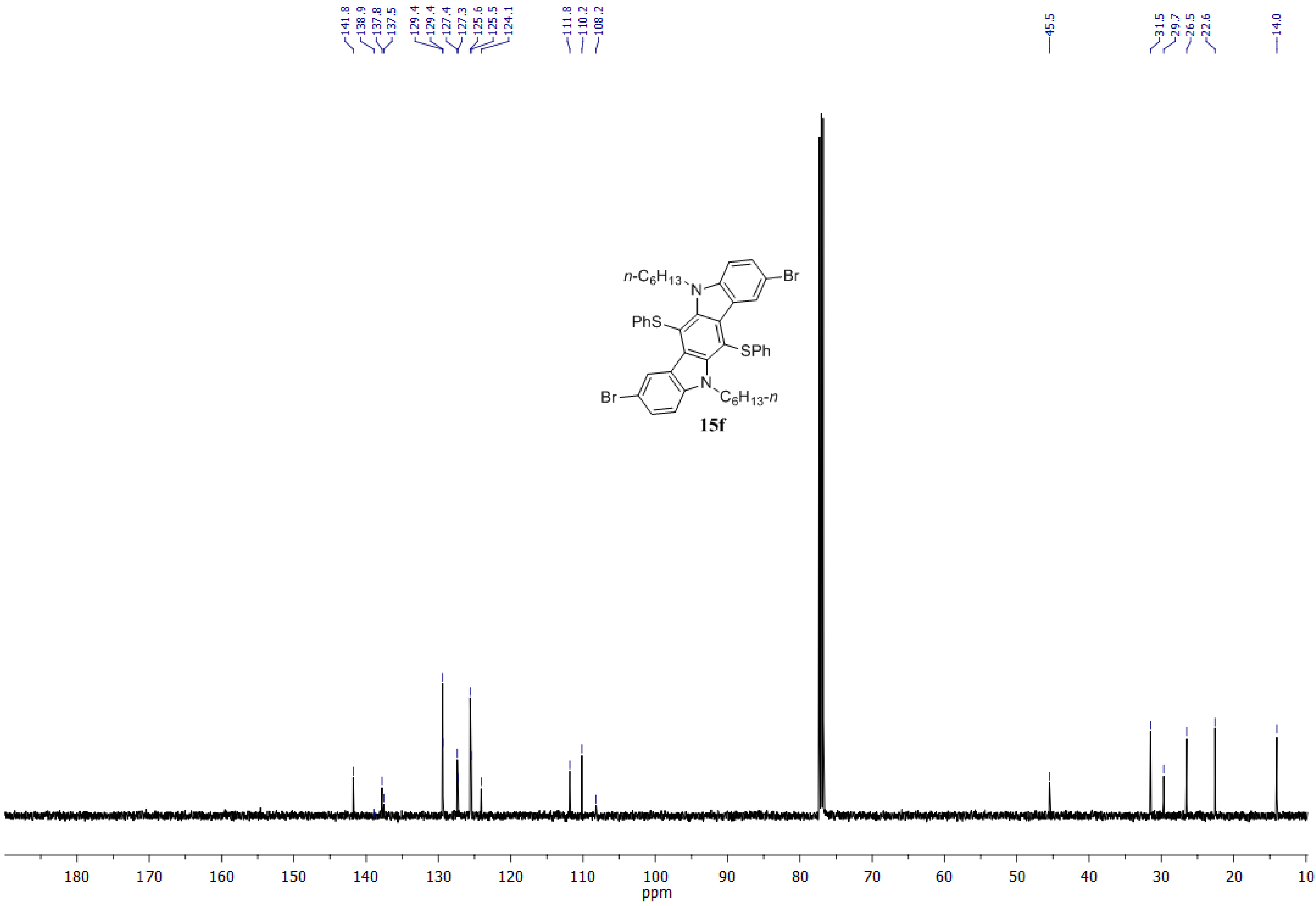

^{1}**H NMR** (solvent: C_6D_6)

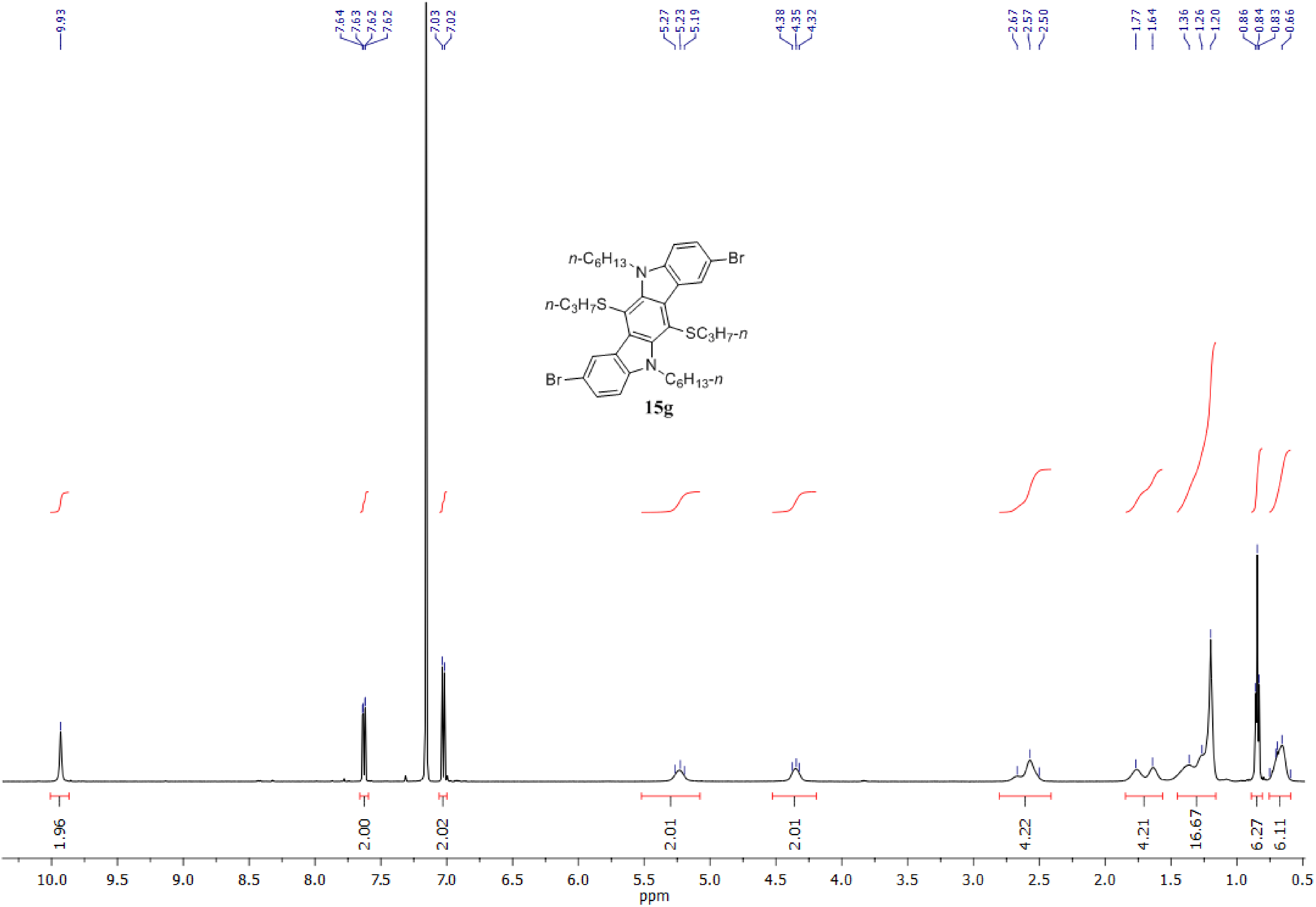

^{13}C NMR (solvent: $CDCl_3$)

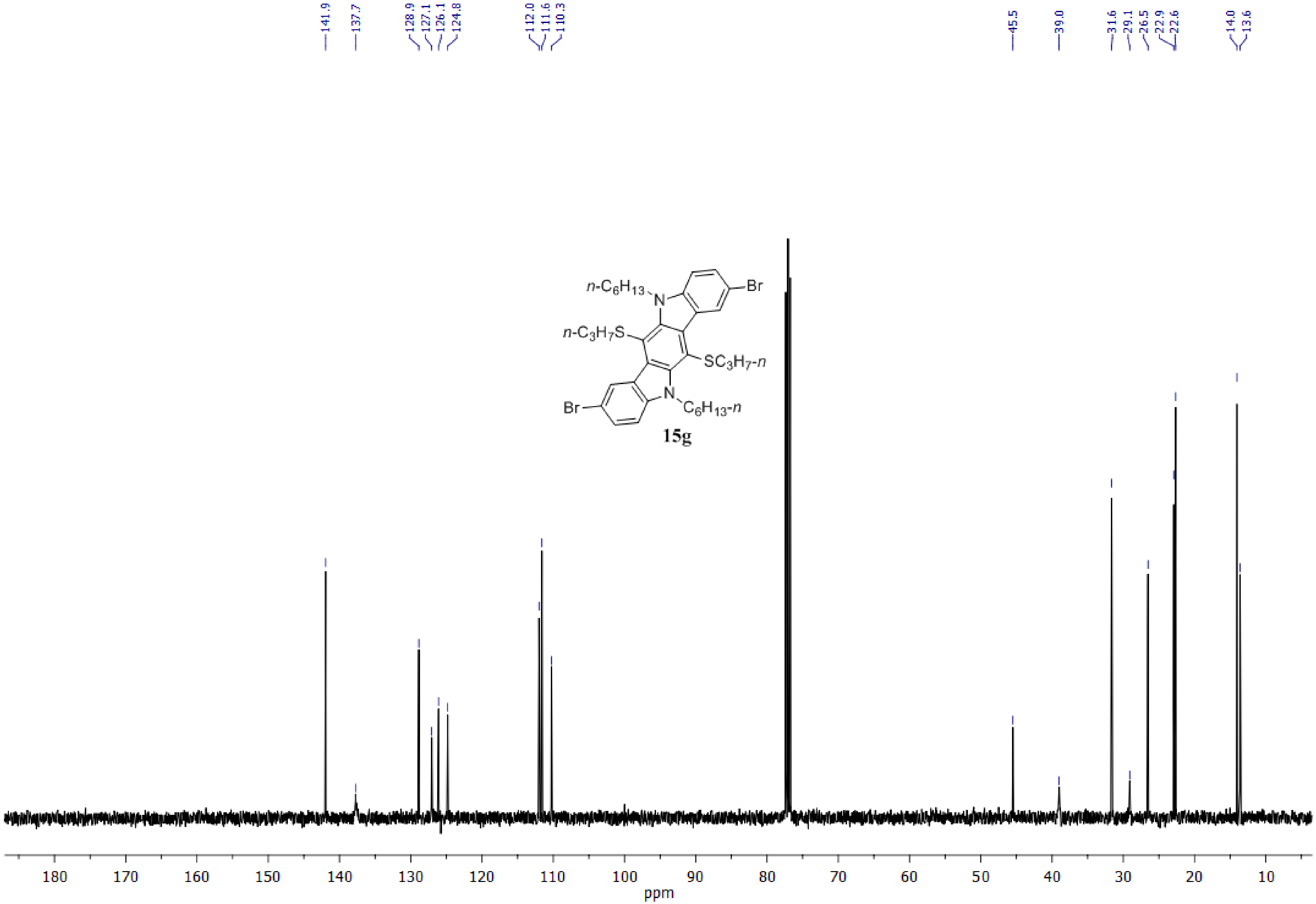

^{1}H NMR (solvent: C_6D_6)

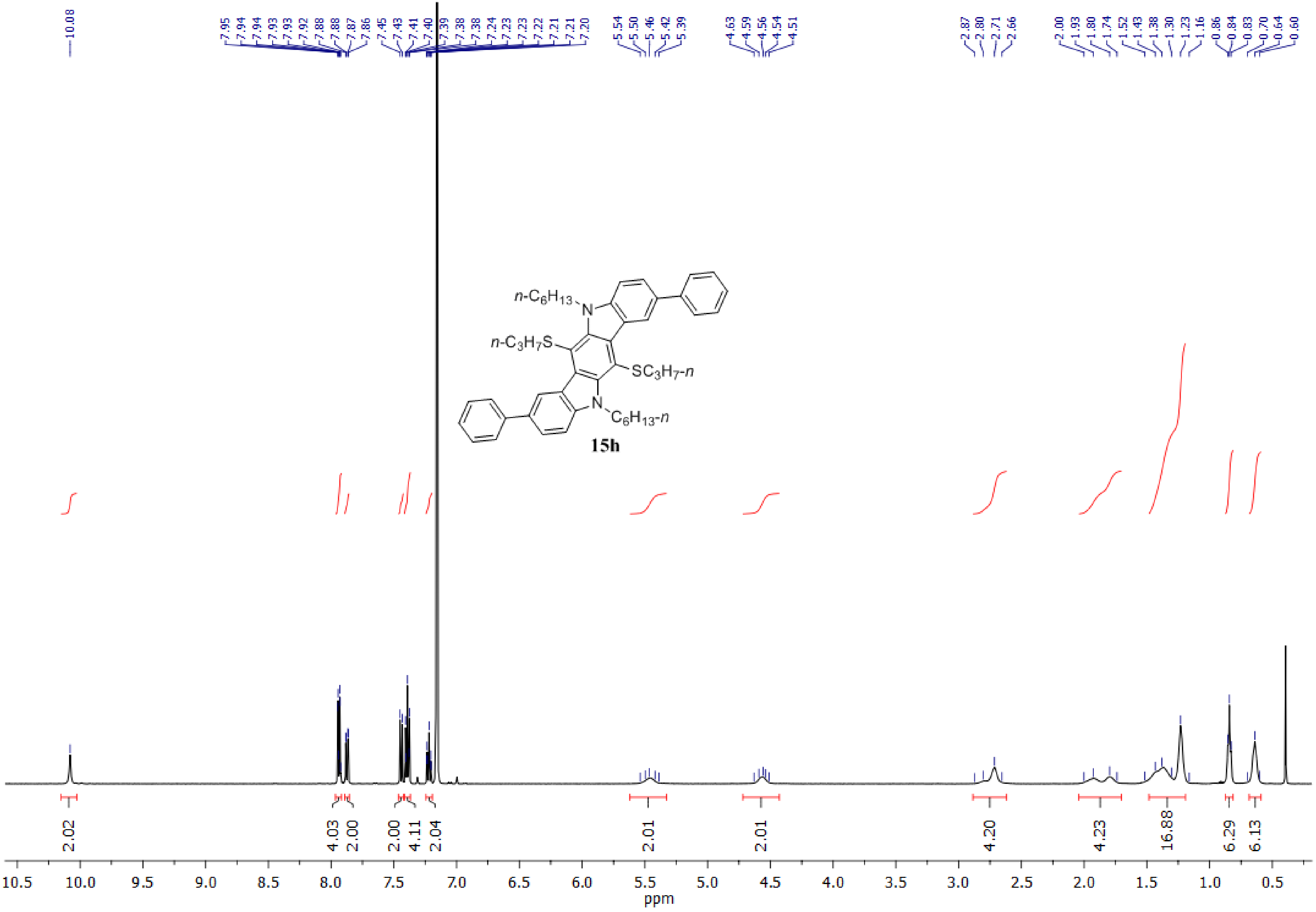

15h

^{13}**C NMR** (solvent: $CDCl_3$)

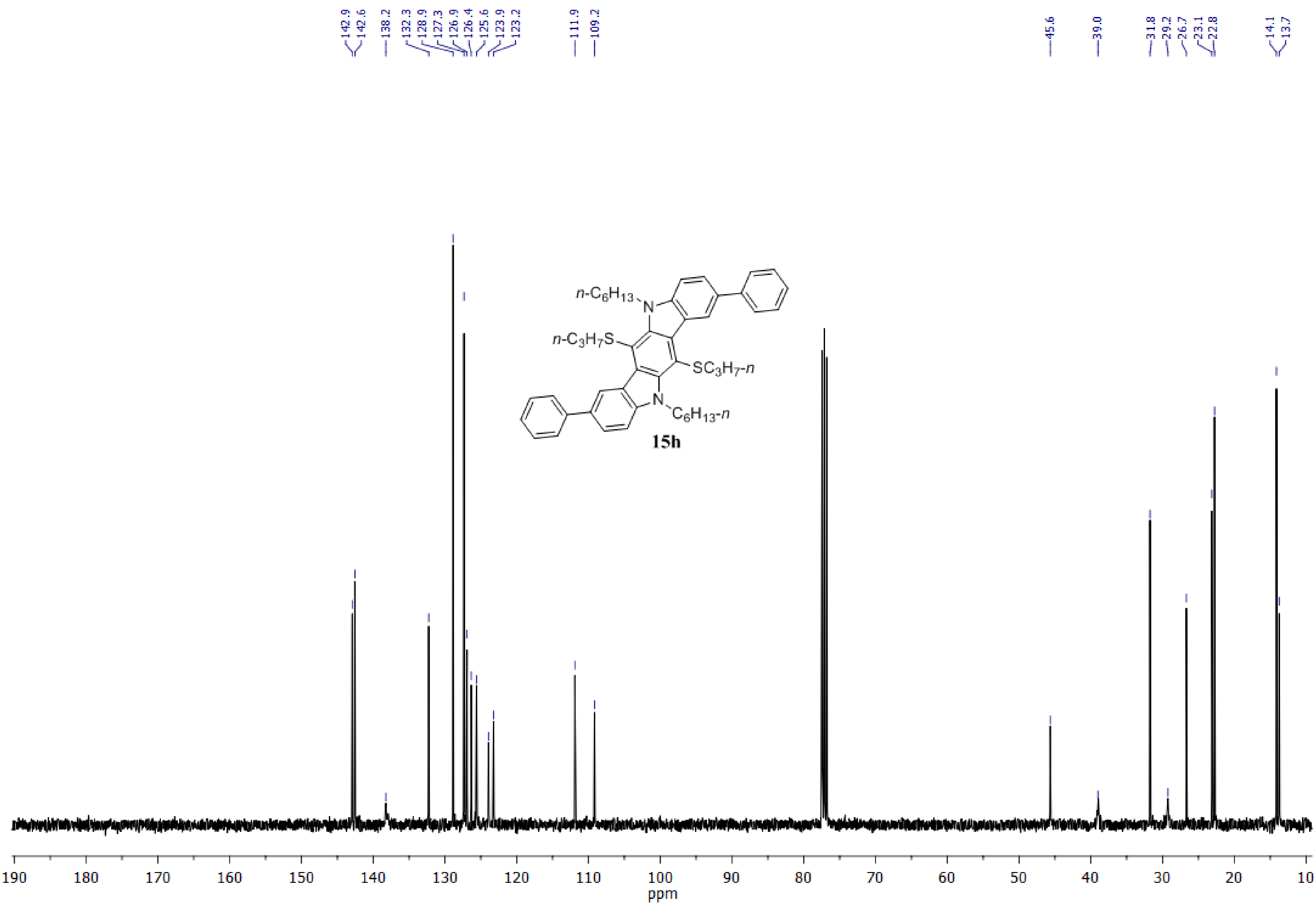

^{1}**H NMR** (solvent: C_6D_6)

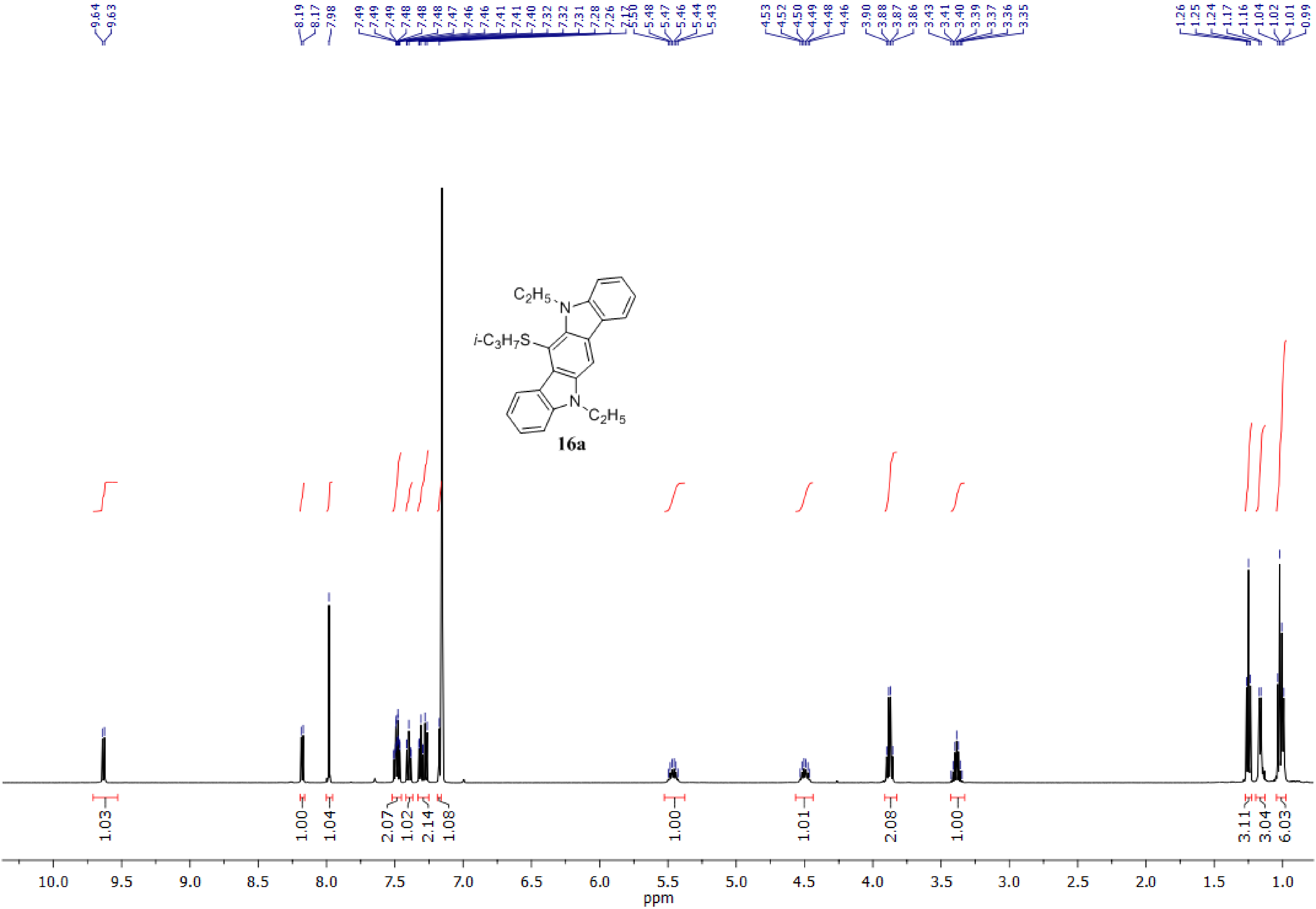

^{13}**C NMR** (solvent: $CDCl_3$)

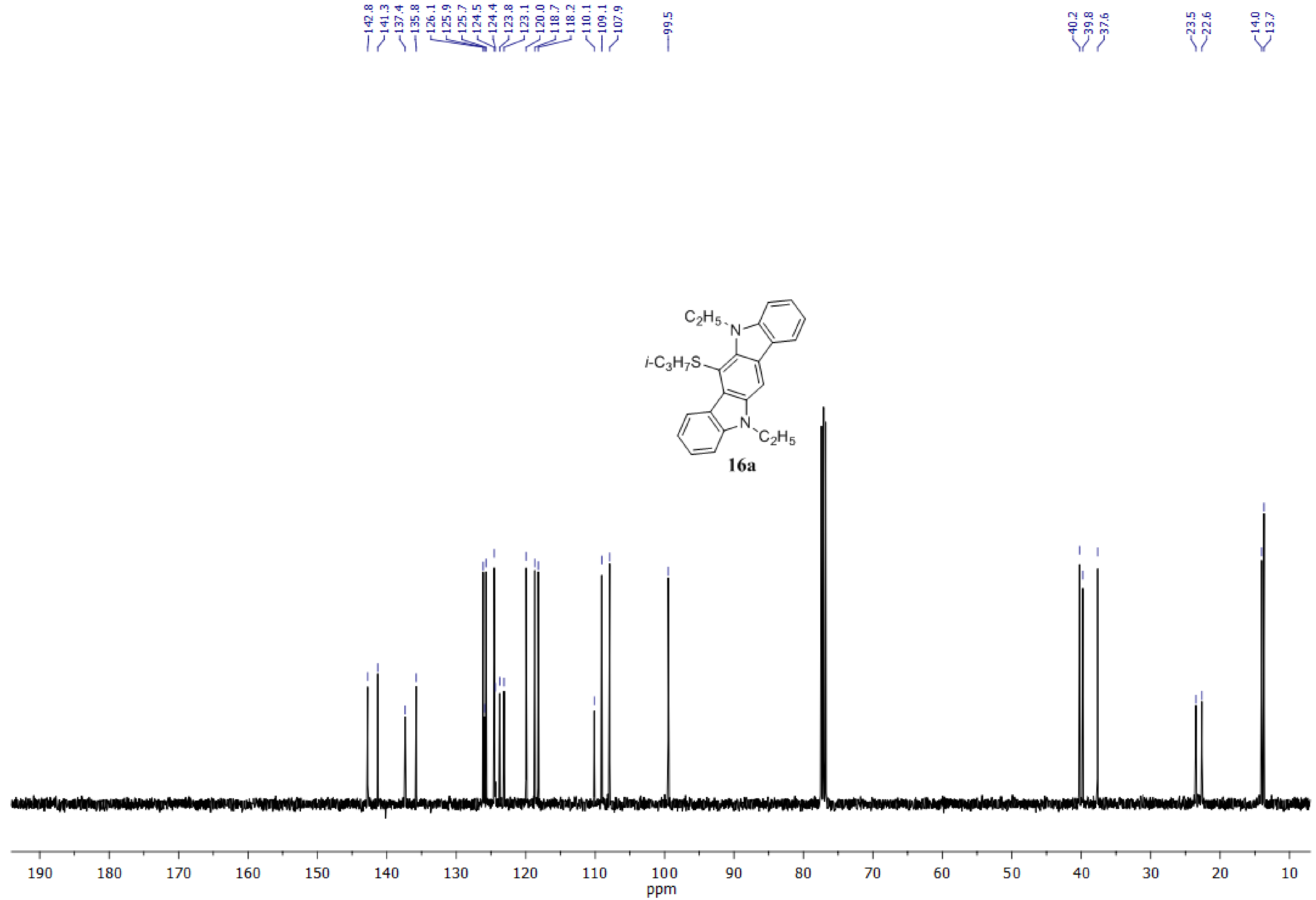

16a

^{1}H NMR (solvent: C_6D_6)

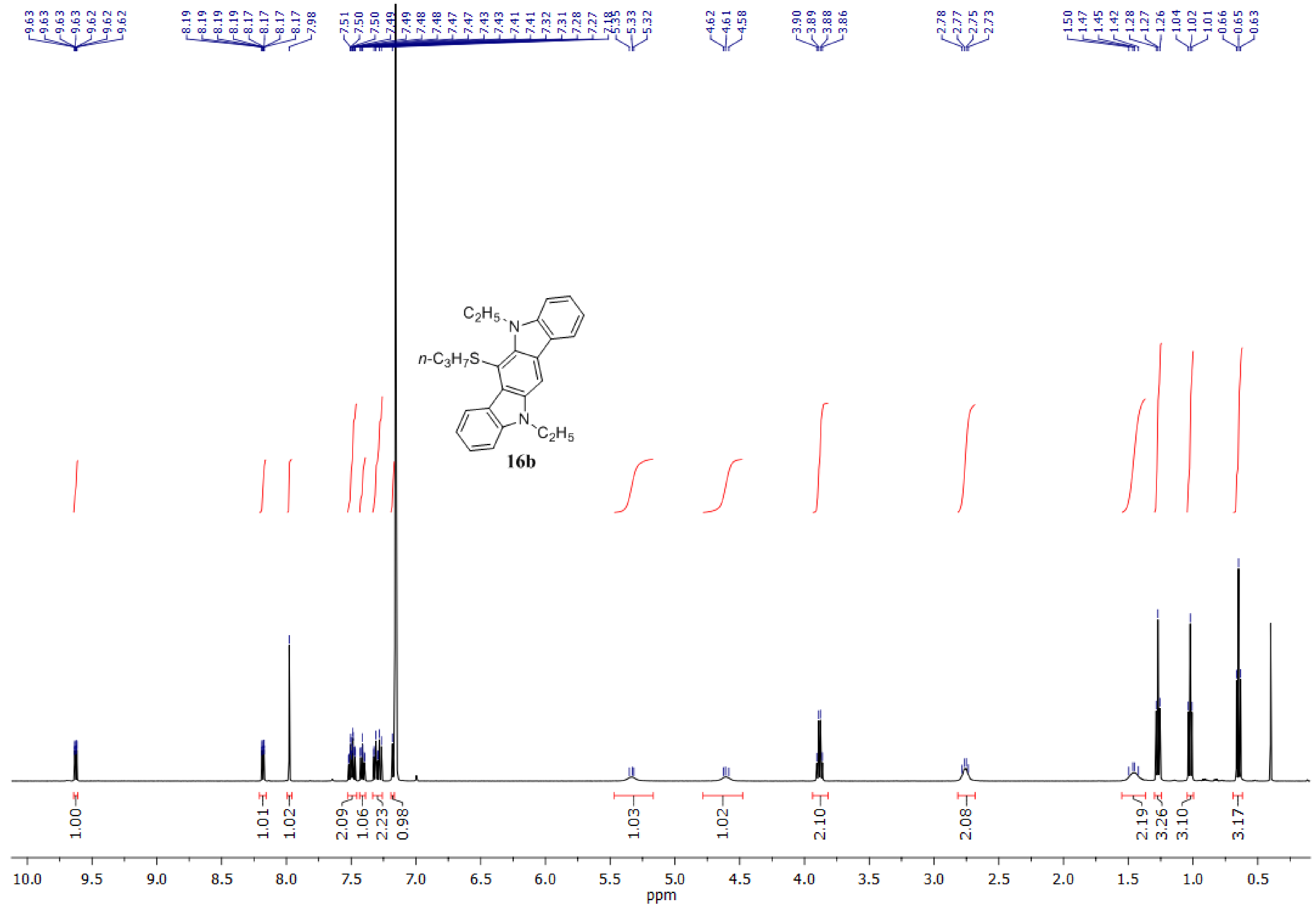

^{13}C NMR (solvent: $CDCl_3$)

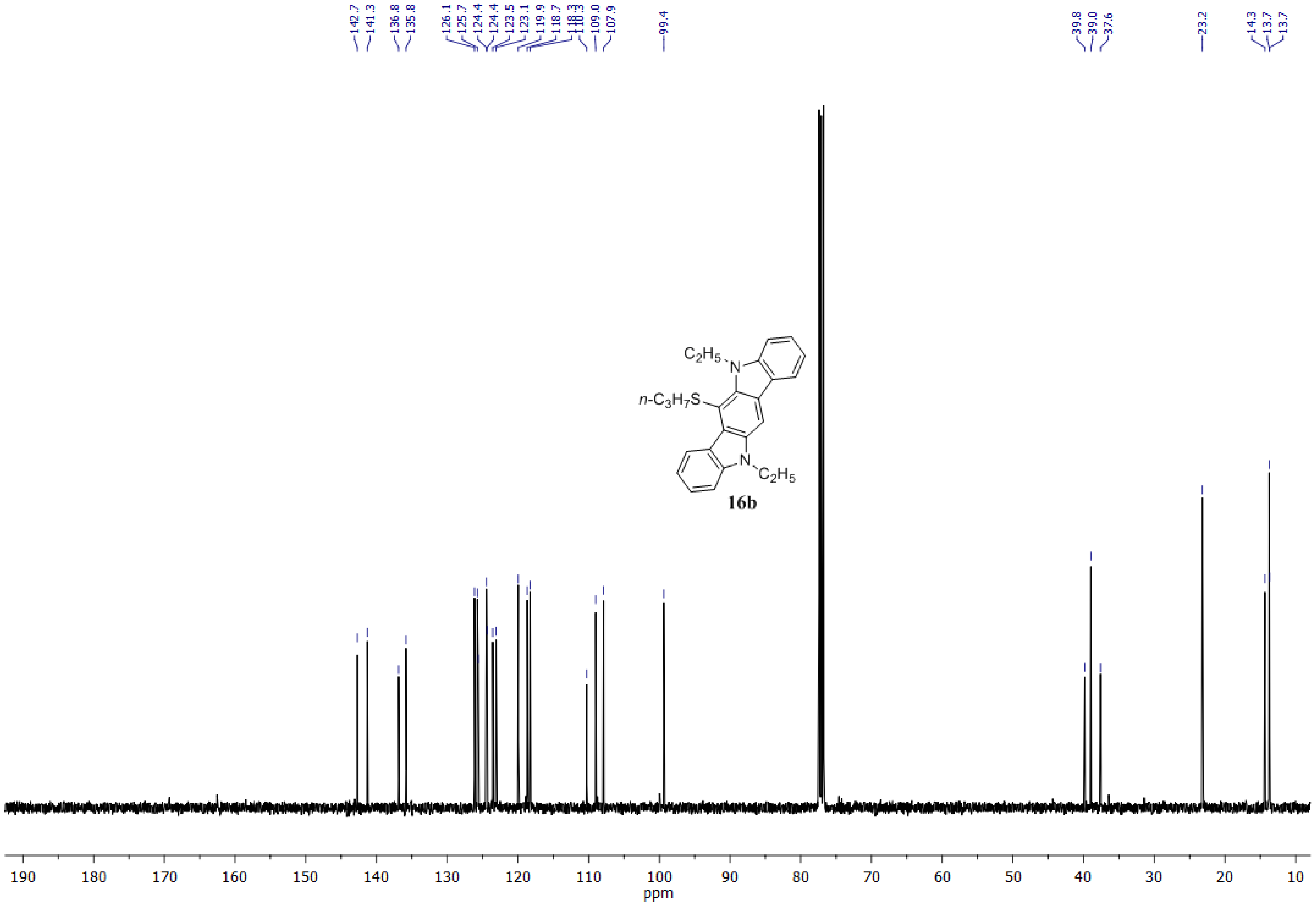

[1]**H NMR** (solvent: $CDCl_3$)

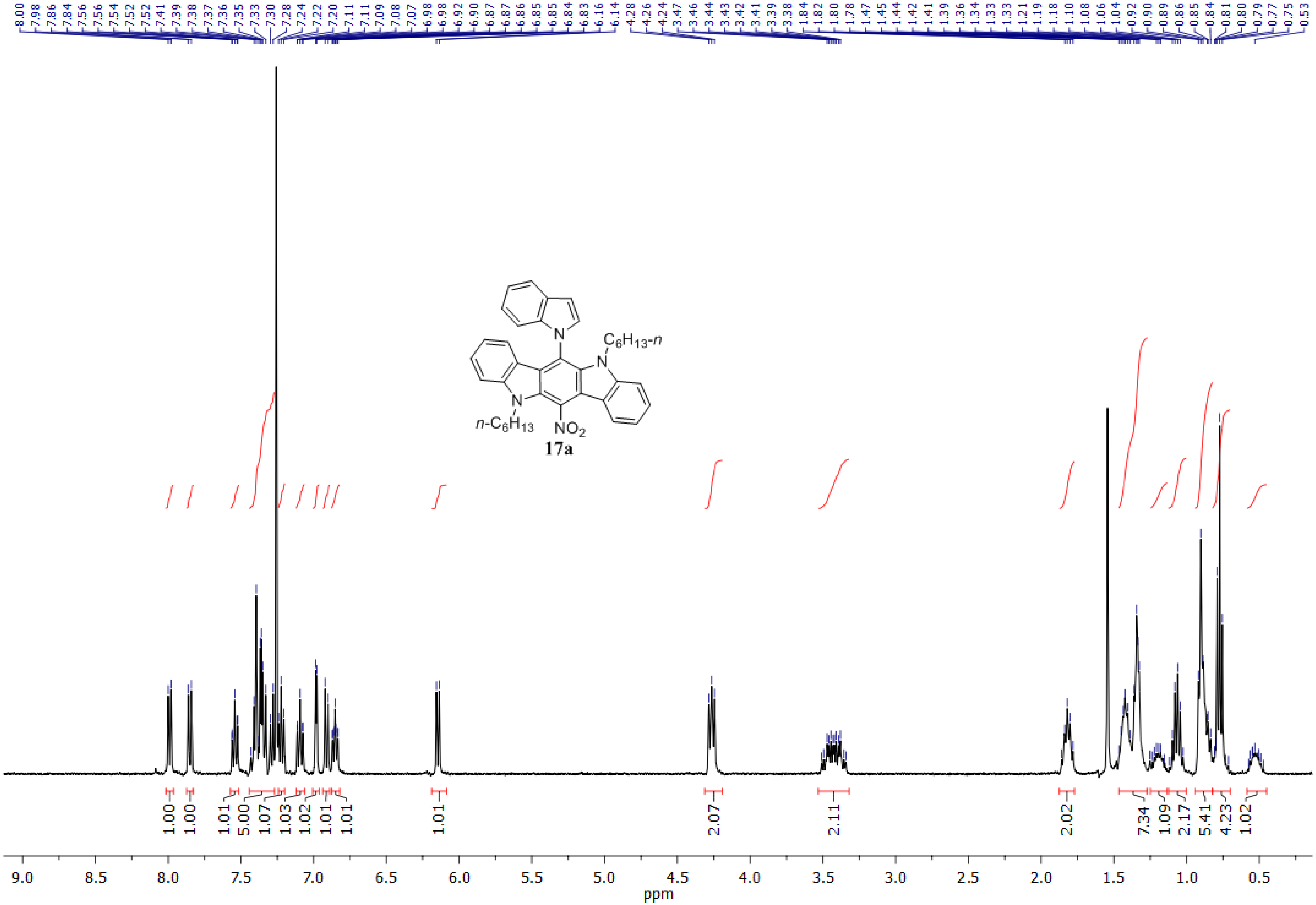

^{13}C NMR (solvent: $CDCl_3$)

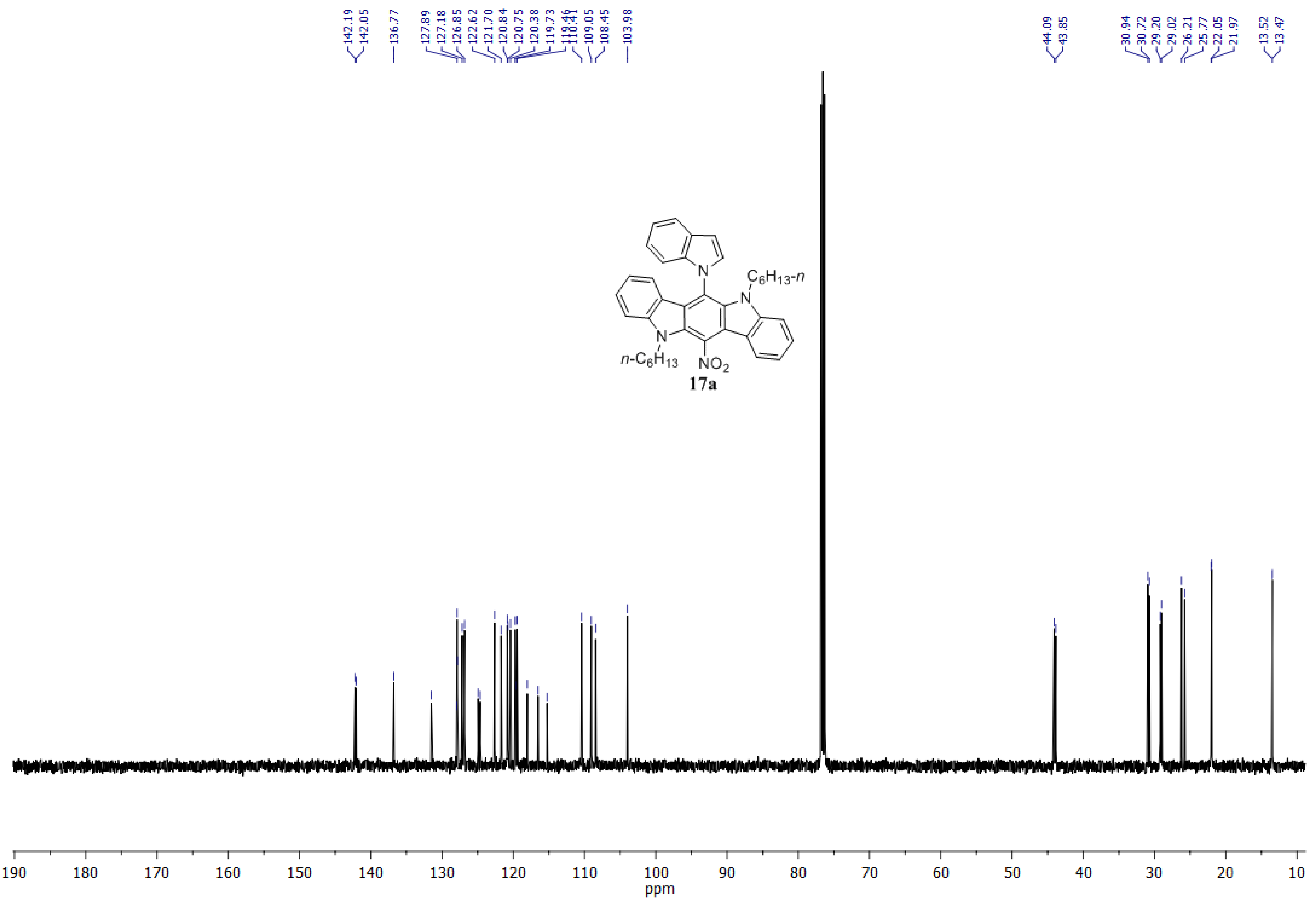

^{1}**H NMR** (solvent: $CDCl_3$)

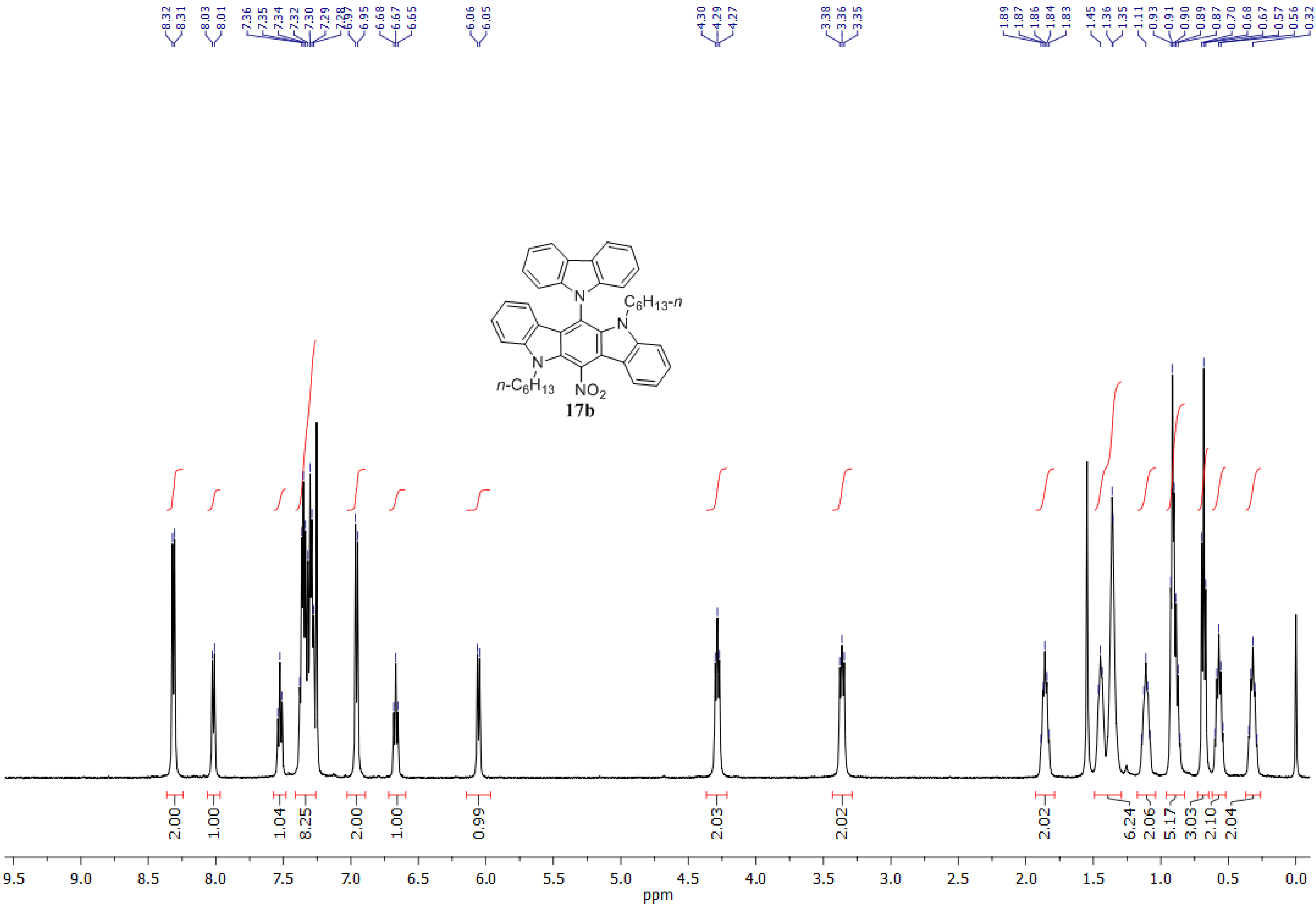

^{13}C NMR (solvent: $CDCl_3$)

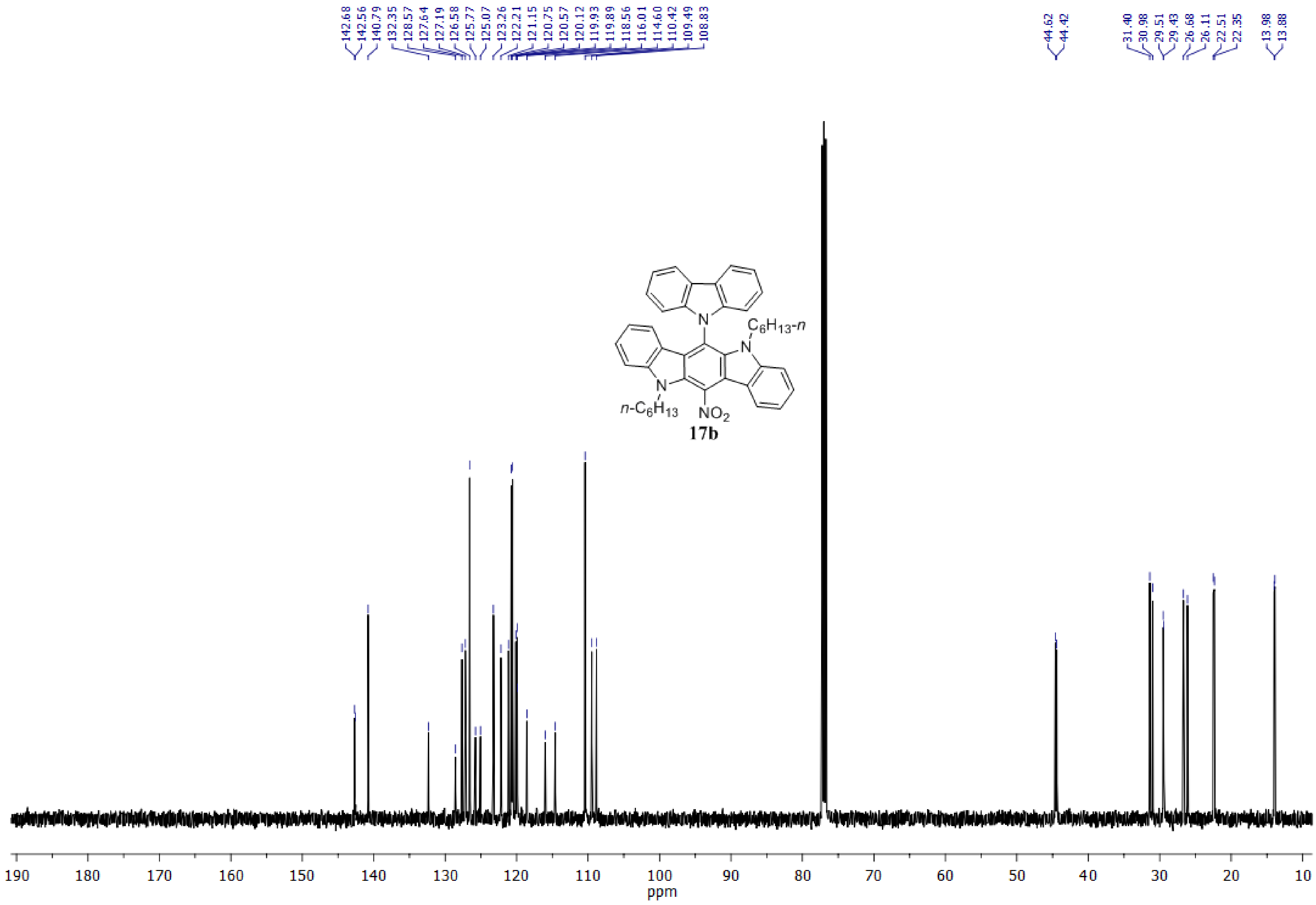

^{1}**H NMR** (solvent: C_6D_6)

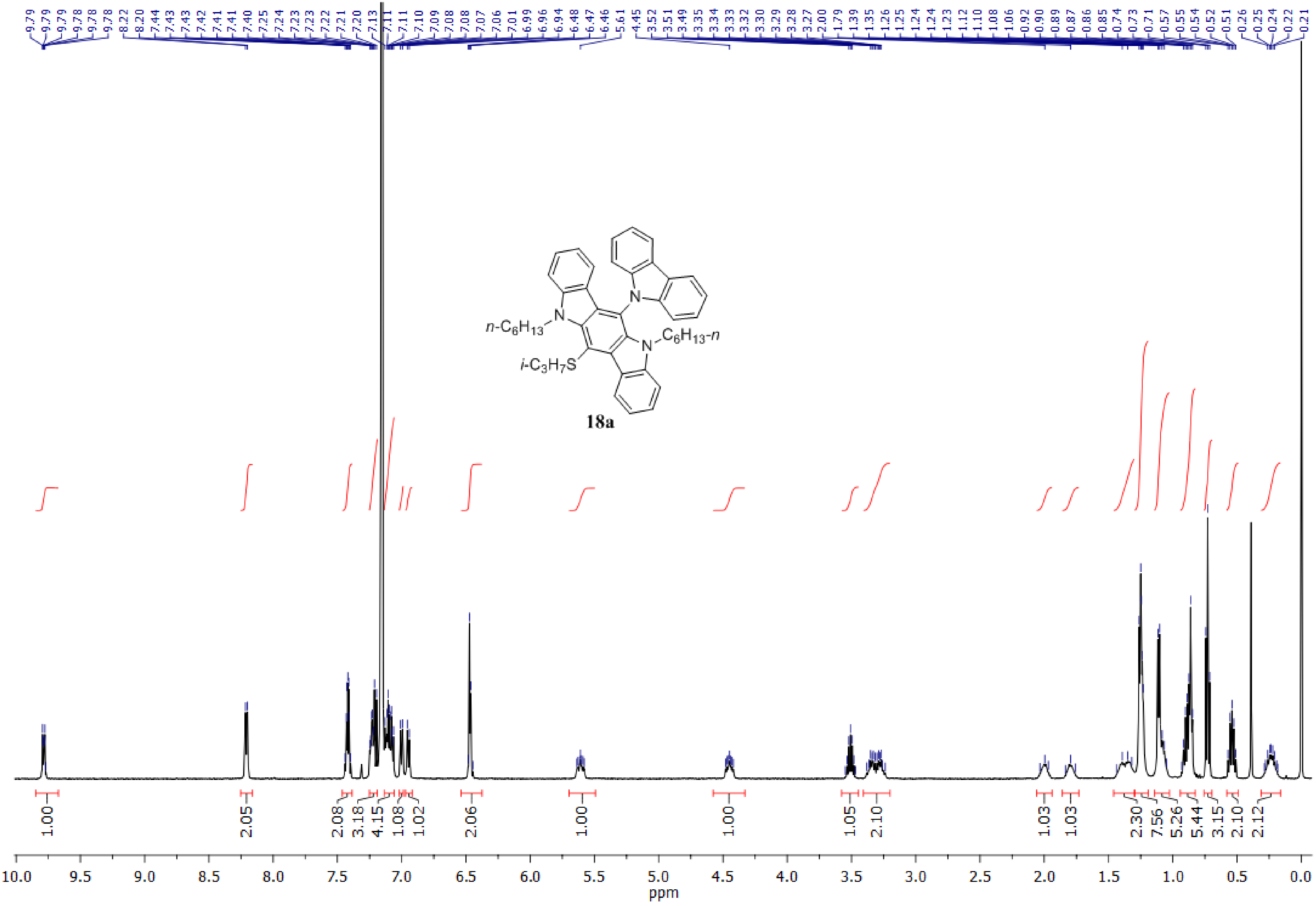

^{13}C NMR (solvent: $CDCl_3$)

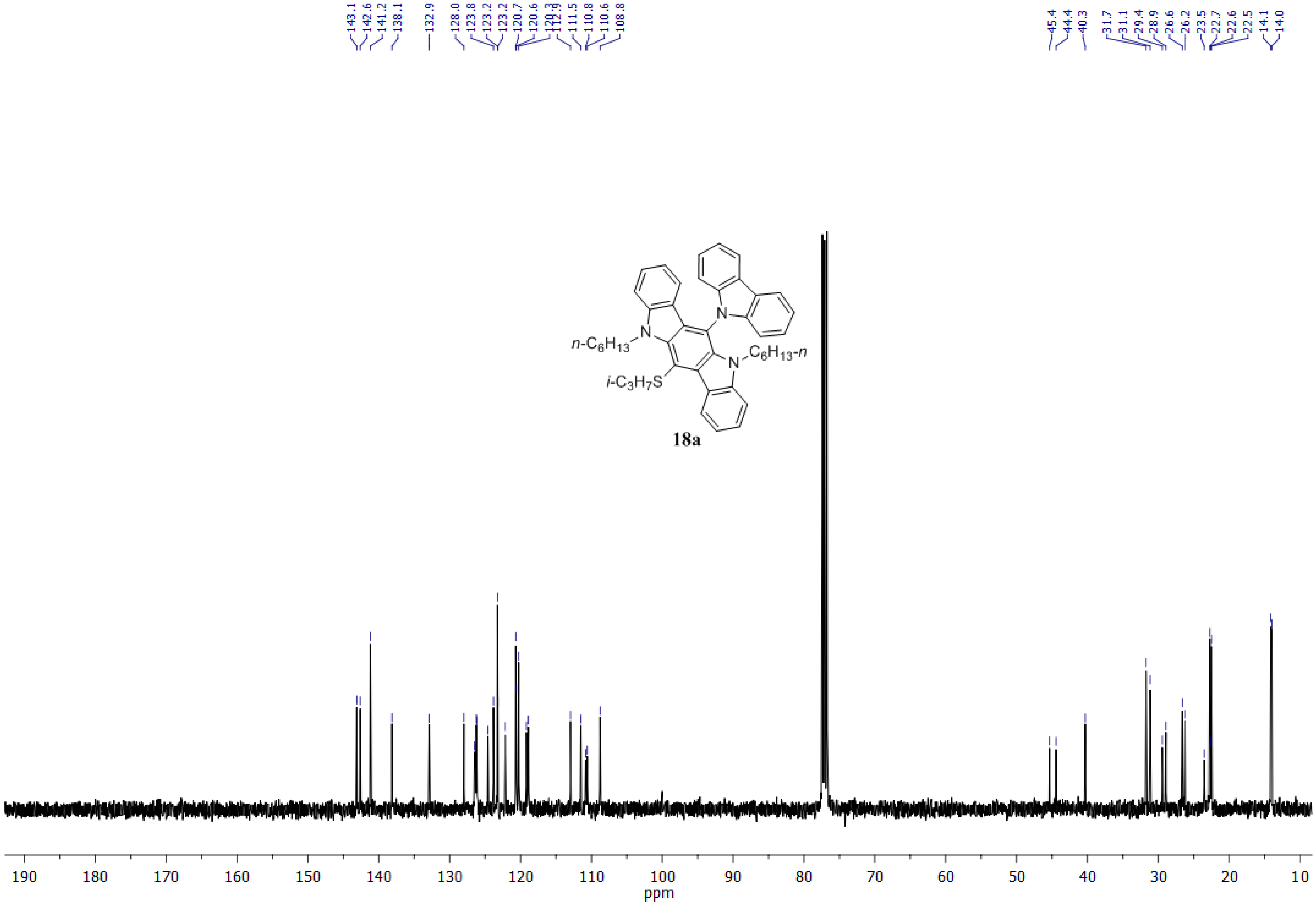

18a

^{1}H NMR (solvent: C_6D_6)

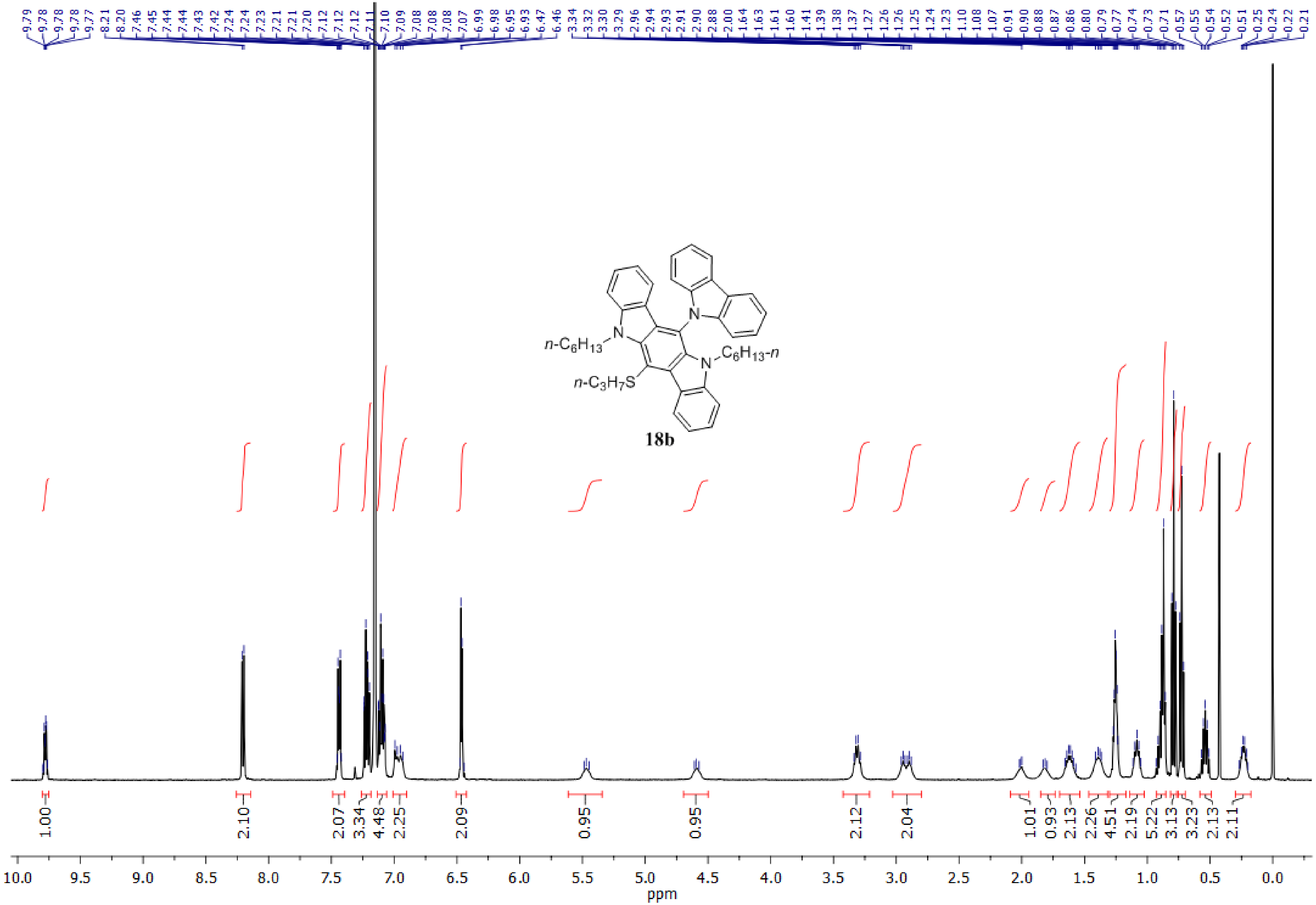

^{13}C NMR (solvent: $CDCl_3$)

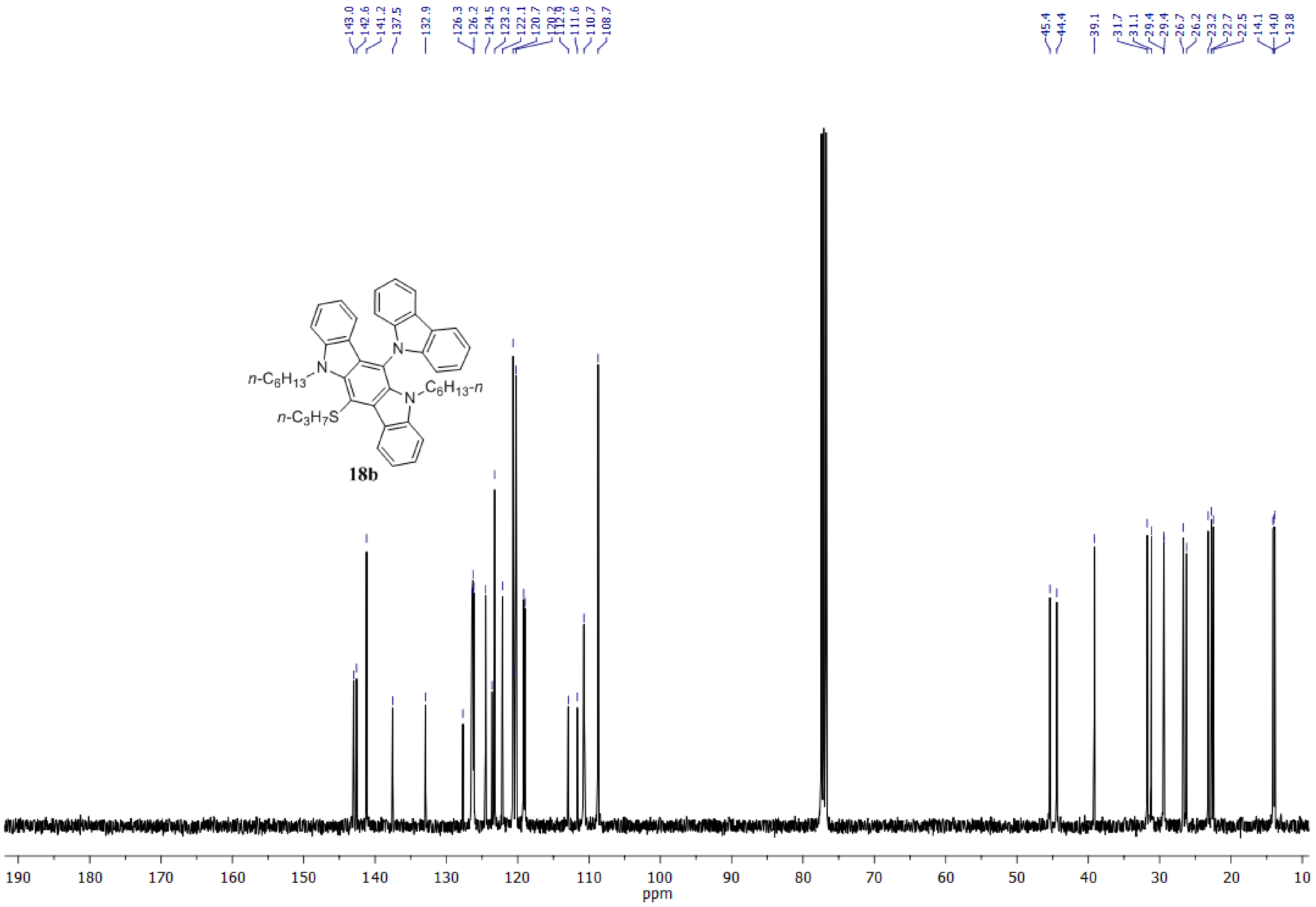

Supplement: File 1 — Experimental procedures, characterization data for new compounds and copies of 1H and 13C NMR spectra. [file Beilstein_J_Org_Chem-13-1396-s001.pdf]
